# Facile access to nitroarenes and nitroheteroarenes using N-nitrosaccharin

**DOI:** 10.1038/s41467-019-11419-y

**Published:** 2019-07-30

**Authors:** Roxan Calvo, Kun Zhang, Alessandro Passera, Dmitry Katayev

**Affiliations:** Department of Chemistry and Applied Biosciences, ETH Zürich, Vladimir-Prelog-Weg 2, 8093 Zürich, Switzerland

**Keywords:** Homogeneous catalysis, Synthetic chemistry methodology, Computational chemistry

## Abstract

Nitroaromatics and nitroheteroaromatics serve as key building blocks and intermediates in synthesis, and form the core scaffold of a vast number of materials, dyes, explosives, agrochemicals and pharmaceuticals. However, their synthesis relies on harsh methodologies involving excess mineral acids, which present a number of critical drawbacks in terms of functional group compatibility and environmental impact. Modern, alternative strategies still suffer from significant limitations in terms of practicality, and a general protocol amenable to the direct C-H functionalization of a broad range of aromatics has remained elusive. Herein we introduce a bench-stable, inexpensive, easy to synthesize and recyclable nitrating reagent based on saccharin. This reagent acts as a controllable source of the nitronium ion, allowing mild and practical nitration of both arenes and heteroarenes displaying an exceptional functional group tolerance.

## Abstract (web summary)

Nitro(hetero) arenes are valuable intermediates in organic synthesis, therefore mild and safe methods to nitrate C-H bonds remain desirable. Here, the authors report a bench-stable, inexpensive, easy to synthesize and recyclable nitrating reagent based on saccharin, allowing for nitration of (hetero)arenes with broad scope.

## Introduction

Electrophilic aromatic nitration is arguably one of the most extensively studied transformations in organic synthesis. The reaction has played a crucial role in our understanding of fundamental concepts underlying the reactivity of aromatics and has become an integral part of any undergraduate curriculum^1^. Furthermore, nitroaromatics have become an essential class of compounds serving as key building blocks and versatile intermediates in organic synthesis and in the preparation of various industrial products^2^. These compounds serve as precursors to amines, hydroxylamines, aldehydes, carboxylic acids, isocyanates and various heterocycles, and are starting materials in the nitro-aldol and Michael reactions, as well as in various cycloadditions^3^. In an industrial context, nitrobenzene is an essential intermediate in the synthesis of aniline^4^, while nitro(hetero)aromatic compounds are important precursors in the synthesis of azo dyes, explosives, and materials^2,4^.

**Fig. 1:**
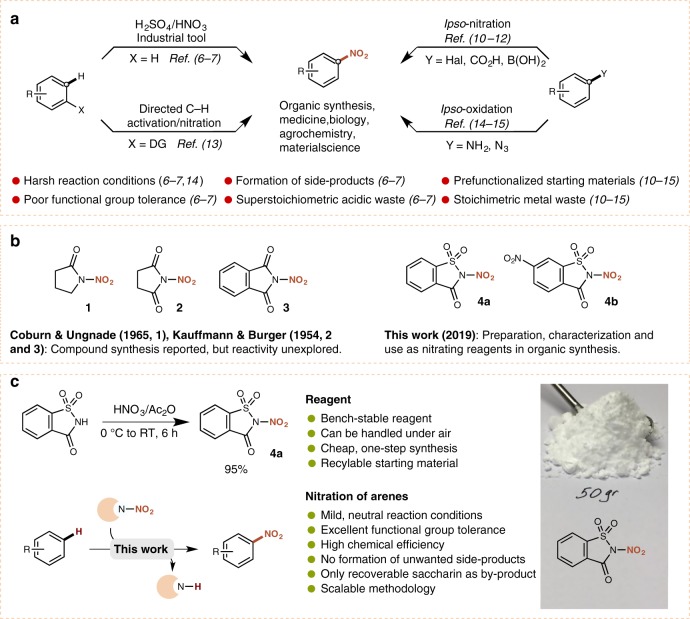
Development of nitrating reagents. **a** Current methods to access nitroarenes. **b** Synthesis of a series of N–NO_2_ heterocyclic compounds based on pyrrolidinone (**1**), succinimide (**2**), phthalimide (**3**), and saccharin (**4**) structures. **c** Our synthesis of reagent **4** and its application in the nitration of arenes

Despite the significance of electrophilic aromatic nitration and the value of nitroaromatic compounds, a mild and practical approach towards electrophilic aromatic nitration remains a challenge for synthetic chemists. To this day, the so called ‘mixed acid’ approach remains the fundamental process for the production of nitroaromatics on both laboratory and industrial scales^5,6^. This methodology utilizes a mixture of nitric acid (HNO_3_) and sulfuric acid (H_2_SO_4_) as a solvent and source of the active electrophilic nitronium (NO_2_^+^) species^7–9^, and it is not surprising that this harsh methodology suffers from several critical drawbacks (Fig. 1a). Crucial issues include the poor regioselectivity and functional group tolerance of the protocol, as it is clearly unamenable to acid-sensitive functional groups, and side products resulting from oxidation or hydrolysis are often obtained. Furthermore, nitrogen oxides (NO_*x*_) are generated along with superstoichiometric amounts of acidic waste, which both presents an environmental hazard and complicates isolation of the desired products^9^.

While other classical electrophilic nitrating reagents, including acyl nitrates, nitryl halides, or nitronium salts circumvent the use of excess quantities of mineral acid, these reactions typically require harsh, and in many cases strictly anhydrous conditions, while some of these reagents are further susceptible to thermal decomposition^9^ (Fig. 1a). Various nitrate salts have been investigated in recent years as alternative nitrating agents, however generate stoichiometric amounts of metal waste^10^. Modern regiospecific nitration methodologies include *ipso*-nitration of prefunctionalized arenes^10–12^ and chelation-assisted nitration using transition metal catalysis^13^ and alternatively, aryl azides^14^, and primary amines^15^ can be oxidized to the corresponding nitro compounds (Fig. 1a). While *tert*-butyl nitrite has been investigated as an organic NO_2_ source in recent years, a general protocol for the nitration of broad classes of arenes using this reagent is still lacking^16,17^.

A mild and practical approach towards the nitration of a diverse range of arenes that does not require prefunctionalized starting materials remains in high demand at both an academic and industrial level. We speculated that the development of a nitrating reagent based on a tunable organic scaffold could offer a versatile approach towards the realization of such a process.

While the application of various *N*-nitramine-based reagents in electrophilic nitration of arenes has been investigated in the last century, these protocols suffer from high-moisture sensitivity, and the use of air-sensitive Lewis acid catalysts^9,18^. Nevertheless, these studies demonstrate the potential of such R_2_N–NO_2_ reagents to act as viable nitrating agents. Herein we present a mild and acid-free methodology for the electrophilic nitration of a diverse range of arenes and heteroarenes displaying an exceptional functional group tolerance. The reaction is enabled by **4**, an easily accessible, inexpensive and recyclable reagent that is a bench-stable and convenient source of the NO_2_ group (Fig. 1c).

## Results

### Reaction development

We commenced by evaluating the reactivity of a series of R_2_N–NO_2_ heterocyclic compounds based on pyrrolidinone (**1**)^19^, succinimide (**2**)^20^, phthalimide (**3**)^20^, and saccharin (**4**) core structures. While the application of **2** in aromatic nitration under photolysis has been investigated, the reagent demonstrated poor reactivity^21^ and likewise, we did not observe nitration of benzene using **2** during our initial reaction screening (Supplementary Table 2). However, we were pleased to observe that the nitration of benzene proceeded in 75% yield using reagent **4a** in slight excess in acetonitrile at 85 °C (Supplementary Table 3). Although the synthesis of **4a** from saccharin has been reported using either unstable NO_2_BF_4_ or gaseous N_2_O_5_, with rudimentary characterization, its application as a nitrating reagent has to the best of our knowledge never been explored^22,23^. We pursued a practical approach towards **4a**, and achieved its synthesis on a 50 g scale in 95% yield from saccharin using a combination of fuming nitric acid and acetic anhydride (Fig. 1c). Structures of both reagents **4a** and **4b** have been confirmed by single crystal X-ray diffraction (Supplementary Tables 5 and 6). Characterization by differential scanning calorimetry and thermal gravimetric analysis revealed **4a** to be stable until 173 °C whereby a rapid exothermal decomposition was observed, accompanied by a mass loss of 87% (Supplementary Fig. 2).

**Fig. 2:**
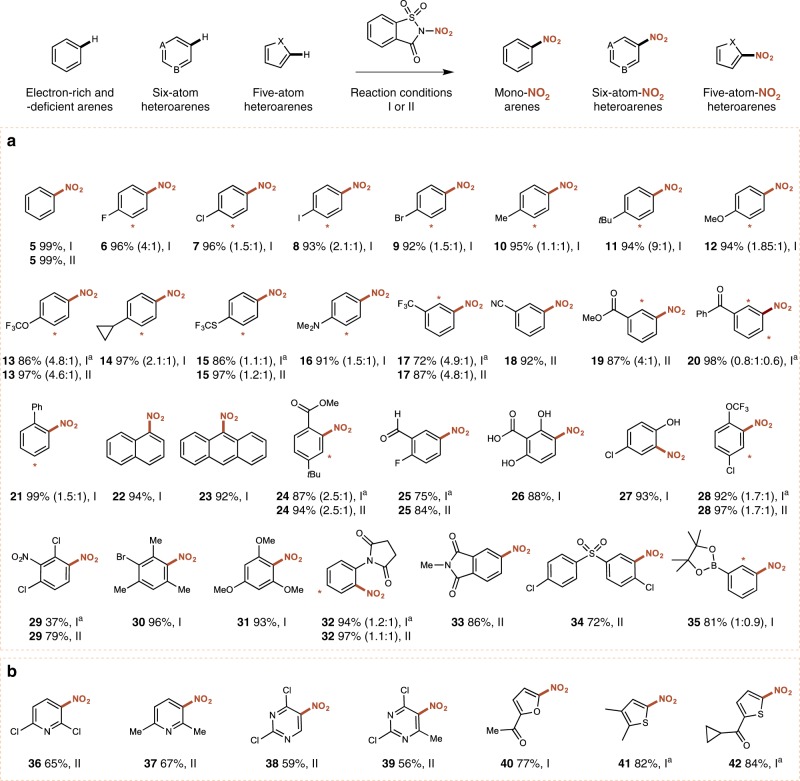
Electrophilic nitration of arenes and five- and six-membered heteroarenes using **4a**. Standard procedures: **I** (Hetero)arene (0.5 mmol), **4a** (0.65 mmol), HFIP (0.5 M), 55 °C, 3 h ([a], 19 h); **II** (Hetero)arene (0.5 mmol), **4a** (0.65 mmol), [Mg(ClO_4_)_2_] (10 mol%), CH_3_CN (0.5 M), 85 °C, 5 h ([a], 19 h). Yields refer to the isolated product of a single regioisomer or a sum of separately isolated regioisomers. [*] The minor regioisomeric position is labeled. Scale up synthesis of **5** under conditions **I** (reaction run on 84.0 mmol of benzene; yield of **5** is 97%). **a** Nitration of small and medium-sized building blocks. **b** Nitration of heteroaromatic compounds

Further screening on the nitration of benzene using **4a** revealed the beneficial effects of fluorinated alcoholic solvents on the reaction, and employing 1,1,1,3,3,3-hexafluoroisopropanol (HFIP) as a solvent at 55 °C afforded nitrobenzene in 99% in 3 h. Additionally, a careful screening of various Brønsted or Lewis acids (Supplementary Table 3) revealed that the reaction proceeded smoothly using 10 mol% of magnesium perchlorate [Mg(ClO_4_)_2_] in acetonitrile at 85 °C. With two methodologies in hand, we proceeded to explore the scope and functional group compatibility in the nitration of various small and medium-sized molecules (Fig. 2). HFIP proved to be suitably activating in almost all cases, and only for exceptionally challenging deactivated substrates was the [Mg(ClO_4_)_2_] protocol employed.

### Reaction scope

Monosubstituted arenes containing both electron-donating and electron-withdrawing groups (**5**–**20**) were successfully nitrated in excellent yields. Halides (**6**–**9**), alkyl (**10**–**11**), and alkoxy-groups (**12**–**13**) were well tolerated, with the expected *o-* and *p-*nitrated products obtained in yields above 90% in all cases (Fig. 2). Remarkably, the nitration of toluene resulted in a slight excess of the *para*-regioisomeric product, which is in stark contrast to the generally observed preference of *o*-nitrobenzene formation under mixed acid conditions or when using nitronium salts^24,25^. Likewise, the ratio of *p-* to *o-tert-*butyl-nitrobenzene is substantially higher than the expected ~ 4:1 ratio using mixed acid (**11**)^24^.

Nitration of cyclopropylbenzene (**14**) proceeded smoothly, with no ring opening observed. The method proved amenable to sulfides (**15**), whereby none of the oxidized side products generally observed when using mixed acid were formed, as well as tertiary amines (**16**). In the case of **15** the use of [Mg(ClO_4_)_2_] catalysis led to a noticeable increase in yield. Similarly [Mg(ClO_4_)_2_] was employed in the nitration of deactivated trifluoromethylbenzene (**17**) and benzonitrile (**18**), furnishing the anticipated *o-* and *m-*substituted products in good yields. The smooth nitration of benzonitrile under our conditions is noteworthy, as aryl nitriles are susceptible to hydrolysis under standard mixed acid conditions^26^. The nitration of methyl benzoate (**19**) and benzophenone (**20**) also proceeded smoothly without any hydrolysis of the ester or ketone moieties. Biphenyl underwent nitration selectively on one aromatic ring (**21**), while nitration of naphthalene (**22**) and anthracene (**23**) delivered only the expected isomers. The reaction proceeded well using various other di-and trisubstituted arenes containing both electron-donating and strongly withdrawing groups (**24**–**31**). Notably, the nitration proved to be compatible with aldehydes (**25**), carboxylic acids (**26**), phenols (**26**–**27**), and amides (**32–33)**, as well as sulfoxides (**34**) and boronate esters (**35**). To our surprise, nitration of methyl 4-*tert*-butyl benzoate resulted in introduction of the nitro group *ortho* to the ester substituent (**24**) as the major regioisomeric product. While the nitration of boronate esters remains poorly explored in the literature^27^, nitration of boronic acids using nitric acid results in formation of the *para*-regioisomeric product in 70% yield^28^. In contrast, we observed an almost 1:1 ratio of *para* to *ortho* products in the nitration of phenyl boronic acid pinacol ester **35**. This could be rationalized on the grounds of a stabilizing interaction between the reagent’s oxygen atom and boron’s empty p-orbital, directing nitration to the *ortho* position (vide infra, Fig. 4e).

Our protocol also proved applicable in the nitration of heteroarenes. While the nitration of 2,6-dichloro-and 2,6-dimethylpyridine typically involves mixed acid at temperatures above 100 °C^29,30^, we were pleased to observe the formation of **36** and **37** in acceptable yield using **4a** under our Lewis acid catalyzed conditions. This protocol was also suitable in the nitration of substituted pyrimidines (**38–39**), while substituted furan (**40**) and thiophenes (**41–42**) were nitrated in good yields in HFIP.

In all cases, we were unable to observe polynitration under established conditions. To further demonstrate the applicability and recyclability of **4a**, the nitration of benzene in HFIP was performed on a 7.5 g scale with no decrease in yield, and both saccharin and HFIP were recovered in 97% and 90%, respectively.

**Fig. 3:**
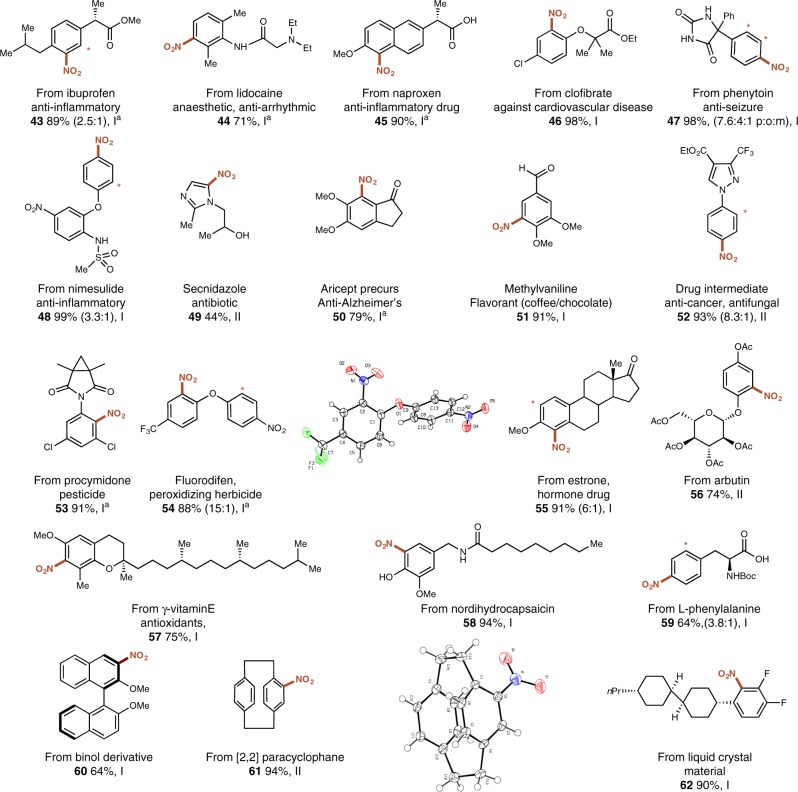
Direct electrophilic nitration of complex molecules and biorelevant compounds using **4a**. Standard procedures: **I** (Hetero)arene (0.5 mmol), **4a** (0.65 mmol), HFIP (0.5 M), 55 °C, 3 h ([a], 19 h); **II** (Hetero)arene (0.5 mmol), **4a** (0.65 mmol), [Mg(ClO_4_)_2_] (10 mol%), CH_3_CN (0.5 M), 85 °C, 5 h ([a], 19 h). Yields refer to the isolated product of a single regioisomer or a sum of separately isolated regioisomers. [*] The minor regioisomeric position is labeled

We subsequently investigated the application of our nitration protocol in late-stage functionalization. Nitroaromatics have in recent decades proven to be an important class of molecules for the preparation of biologically active substances, finding application as pesticides^31^, and as anticancer, antiparasitic and antitubercular agents, as well as antibiotics and tranquilizers^32^ and as such, we foresaw that our methodology could be of particular value in the context of medicinal and agrochemistry. To this end, a series of nitrated derivatives of known drugs, including ibuprofen (**43**), lidocaine (**44**), naproxen (**45**), clofibrate (**46**), phenytoin (**47**), and nimesulide (**48**) were synthesized in good to excellent yields, the latter furthermore demonstrating the protocol’s tolerance to sulfonamides (Fig. 3). In addition, our protocol enabled the direct preparation of antibiotic Secnidazol (**49**)^33^. A precursor to the Alzheimer’s drug Aricept (**50**), the commonly used fragrant and intermediate in the synthesis of various pharmaceuticals ventraldehyde (**51**), and drug intermediate **52** all smoothly underwent nitration. Furthermore, a nitrated derivative of pesticide procymidone (**53**) was prepared, and the herbicide fluorodifen (**54**)^34^ could be synthesized directly from the corresponding diphenyl ether in 88% yield and good regioselectivity. Our protocol was also successfully applied in the nitration of naturally occurring estrone (**55**), arbutin (**56**), vitamin E (**57**), nodihydrocapsaicin (**58**), and L-phenylalanine (**59**). To show the applicability of our protocol beyond pharmaceuticals and agrochemicals, nitrated derivatives of chiral building blocks binol (**60**) and [2,2] paracyclophane (**61**) were synthesized, thereby providing a convenient strategy for the development of chiral ligands for asymmetric catalysis. The reaction also proved suitable for the modification of organic materials, demonstrated through the nitration of a liquid crystalline material (**62**).

### Mechanistic considerations

**Fig. 4:**
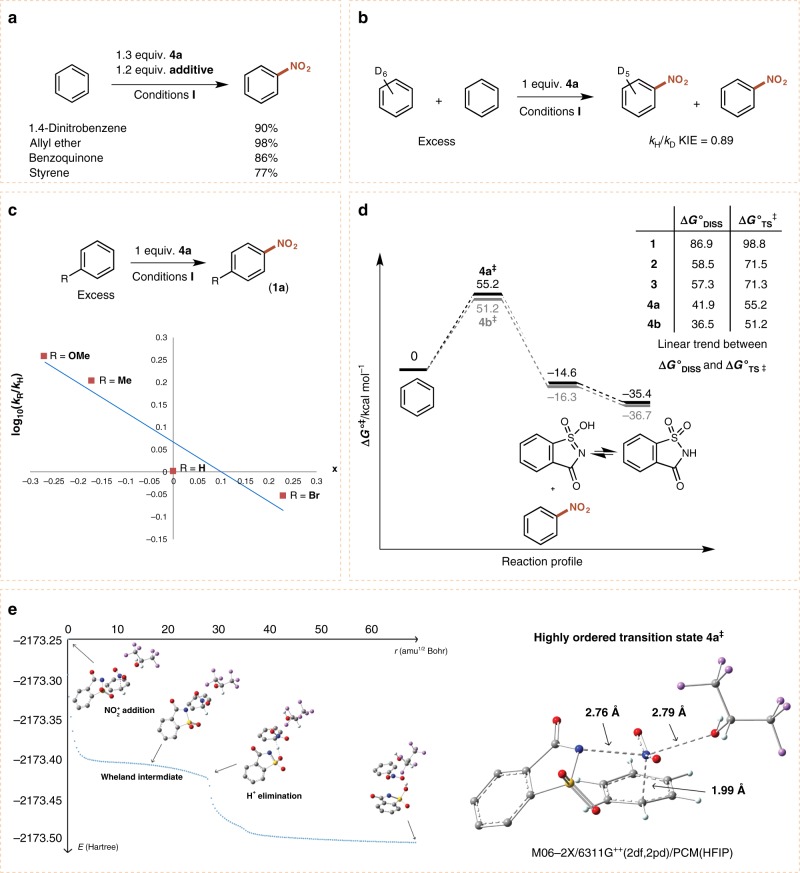
Mechanistic studies into the nitration of arenes using **4** and procedure **I**. **a** Radical trapping experiments. **b** Kinetic isotope effect by intermolecular competition experiment. **c** Hammett study. **d** Gibbs-free energy profile for nitration of benzene using **4a** and **4b**. **e** Transition state of nitronium addition to benzene and intrinsic reaction coordinate for nitration of benzene after rate-determining step

Despite arene nitration via electrophilic aromatic substitution being one of the oldest studied reactions, mechanistic aspects of the transformation are still being elucidated today^1,35^. In light of recent studies considering the effects of implicit solvent molecules on electrophilic nitration of arenes under acidic conditions^36,37^, we were motivated to gain mechanistic insight into the transfer of the NO_2_ group and the role of HFIP in facilitating this reaction. We commenced by performing the nitration of benzene in the presence of radical scavengers, and did not observe significant inhibition (Fig. 4a). An intermolecular competition experiment with a secondary isotope effect of 0.89 is consistent with the known fact that C–N bond formation is the rate-determining step in electrophilic aromatic substitution (Fig. 4b)^38^. Subsequently, a Hammett study revealed a small negative *ρ*-value of −0.66 (Fig. 4c). In the presence of reagent **4a**, the half-life (*t*_1/2_) of the first order reaction on the substrate was ~ 92 min (Supplementary Fig. 9), while using reagent **4b** the reaction rate drastically increased, with a *t*_1/2_ of ~ 8 min (Supplementary Fig. 10).

Owing to its unique properties, HFIP has in recent years proven to be a versatile solvent and additive with broad applications^39–41^. While HFIP may promote nitration due to its cation stabilization ability, we postulated that its strong hydrogen bonding capacity may also activate **4a**.

To further distinguish between a polar electrophilic process or radical mechanism, and to understand the role of HFIP, we turned to density functional theory (DFT) studies. Prior to nitronium transfer we did not observe formation of a π complex, but the NO_2_ group of **4a** was found to interact with HFIP via a hydrogen bond (Supplementary Fig. 15). The reaction then proceeds with a concerted and strongly asynchronous mechanism (Fig. 4e) whereby the C−N bond formation was found to be the rate-determining step (Fig. 4d), in agreement with the first-order reaction in benzene (Supplementary Fig. 9) and the observed KIE (Fig. 4b). In this transformation, the σ complex was not located as a discreet intermediate, although it is observed in the reaction profile and is rapidly followed by H^+^ elimination assisted by saccharin’s sulfoxide group (Fig. 4d, e)^36,42^.

The reagent’s activity as a controllable source of the nitronium ion is elucidated when considering the high level of organization in the transition state, where HFIP (O atom) interacts with the NO_2_ group (N atom, 2.79 Å), assisting the cleavage of the N−N bond and the concomitant addition of the nitronium species to the aromatic ring (Fig. 4e). It is this highly ordered transition state that enables delivery of the nitronium ion from **4a** in a controlled fashion.

Curious about the high *para*:*ortho* ratio in the nitration of *tert*-butyl-benzene, we calculated the selectivity according to the transition state distribution. A good match was found between the experimental and calculated ratios, and the high *para* selectivity can be explained by steric hindrance in the transition state, whereby **4a** shields one *ortho* position, and the HFIP molecule the other (Supplementary Fig. 22). This steric hindrance likewise also accounts for the introduction of the nitro group *ortho* to the ester substituent in **24**.

Finally, a trend was found between the activation Gibbs free energy (Δ*G*°^‡^) and the dissociation Gibbs free energy for the heterolytic cleavage of the N–N bond (Δ*G*°_DISS_) for the series of nitrating reagents **1–4**. The findings are in agreement with the drastically increased rate of reaction for **4b** compared to **4a** (Supplementary Figs. 9 and 10), and also allows prediction of the reactivity of **1–4** (Fig. 4d).

## Discussion

Using the bench-stable and recyclable electrophilic nitrating reagent **4a**, we have developed a general protocol for the direct synthesis of a broad range of nitroarenes and nitroheteroarenes demonstrating an exceptional functional group tolerance. Furthermore, our method was applicable to the late-stage C–H functionalization of a range of complex molecules. Mechanistic studies strongly support a classical electrophilic aromatic nitration, which was found to proceed through a unique and highly ordered transition state. As such, our reagent behaves as a controllable source of the nitronium ion, enabling such a mild and functional group tolerant reaction.

## Methods

### General procedure I for the nitration of (hetero)arenes

An oven-dried 25 ml micro-vial was charged on the benchtop with a magnetic pTFE-coated stirbar and **4a** (148 mg, 0.65 mmol, 1.3 equiv.). The vial was sealed and the atmosphere was cycled 3x with Ar/vac. (Hetero)arene substrate (0.5 mmol, 1.0 equiv.) in HFIP (1 ml) was added by plastic syringe and the reaction mixture was heated at 55 °C with vigorous stirring for 3 h. After cooling to room temperature, the solvent was removed under reduced pressure, and the product was purified by flash column chromatography (SiO_2_, ethyl acetate/*n*-hexange gradient).

### General procedure II for the nitration of (hetero)arenes

An oven-dried 25 ml micro-vial was charged on the benchtop with a magnetic pTFE-coated stirbar, **4a** (148 mg, 0.65 mmol, 1.3 equiv.) and [Mg(ClO_4_)_2_] (11.2 mg, 0.05 mmol, 10 mol%). The vial was sealed and the atmosphere was cycled 3x with Ar/vac. (Hetero)arene substrate (0.5 mmol, 1.0 equiv.) in MeCN (1 ml) was added with a plastic syringe, and the reaction mixture was heated at 85 °C with vigorous stirring for 5 h. After cooling to room temperature, the solvent was removed under reduced pressure, and the product was purified by flash column chromatography (SiO_2_, ethyl acetate/*n*-hexane gradient).

## Supplementary information

Supplementary Information

Peer Review File

Description of Additional Supplementary Data Files

Supplementary Data 1

Supplementary Data 2

Supplementary Data 3

Supplementary Data 4

## Data Availability

The authors declare that the data supporting the findings of this study, including synthetic procedures, NMR spectra, characterization for all new compounds and further details of computational studies, are available in the article and its Supplementary Information, or from the corresponding author upon reasonable request. The X-ray crystallographic coordinates for structures of **4a**, **4b**, **54**, and **61** have been deposited at the Cambridge Crystallographic Data Center (CCDC), under deposition numbers CCDC-1903469, CCDC-1903468, CCDC-1903470, and CCDC-1456828, respectively. These data can be obtained free of charge from The Cambridge Crystallographic Data Center via https://www.ccdc.cam.ac.uk/.
