# Supplementary material for: Facile access to nitroarenes and nitroheteroarenes using N-nitrosaccharin
Source: Nat Commun. 2019 Jul 30;10:3410. doi: 10.1038/s41467-019-11419-y (PMC6667458; doi:10.1038/s41467-019-11419-y)
Supplement: Supplementary file 1 — Supplementary Information [file 41467_2019_11419_MOESM1_ESM.pdf]

Facile Access to Nitroarenes and Nitroheteroarenes Using N-Nitrosaccharin

Calvo et al.

Supplementary Methods

Materials and methods: Starting materials (arene derivatives) are commercially available and were purchased from Sigma Aldrich or ABCR (Germany) unless otherwise noted. Compounds **1** (*19*, *43*-*45*), **2** (*20*) and **3** (*20*) have been prepared in accordance with previously reported procedures. All commercially available starting materials were analyzed by ^{1}H NMR spectroscopy prior to use. Anhydrous acetonitrile was stored over pre-conditioned 3 Å mol sieves for at least 2 days prior to use. HFIP was purchased from Fluorochem Ltd (1 Kg – 154.25 CHF) and used without purification. Analytical thin layer chromatography (TLC) was performed on Merck silica gel 60 F254 TLC glass plates and visualized with 254 nm light and potassium permanganate or ceric ammonium molybdate staining solutions followed by heating. Medium pressure liquid chromatography (MPLC) was performed on a CombiFlash Rf200 System from Teledyne ISCO with built-in UV-detector and fraction collector or manually using silica gel SilicaFlash P60, 40-63 µm. Teledyne ISCO RediSep Rf flash columns used have a 0.035–0.070 mm particle size and 230–400 mesh. Normal phase preparatory HPLC purification was conducted on a Teledyne Isco CombiFlash EZ Prep system using a Macherey-Nagel VP 250/21 Nucleosil 50-5 column.

NMR spectra were acquired on a Bruker AVIII HD 500 MHz, 400 MHz and 300 MHz spectrometers, operating at the denoted spectrometer frequency given in MHz for the specified nucleus. All experiments were acquired at 298.0 K with a calibrated Bruker Variable Temperature Controller unless otherwise noted. The chemical shifts are reported in parts per million (ppm) and coupling constants (*J*) are given in Hertz (Hz). ^{1}H NMR spectra are reported with the solvent resonance as the reference unless noted otherwise (CD_3CN at 1.94 ppm, $CDCl_3$ at 7.26 ppm, CD_3OD at 3.31 ppm, CD_2Cl_2 at 5.32 ppm, DMSO-d_6 at 2.50 ppm). Peaks are reported as s = singlet, d = doublet, t = triplet, q = quartet, m = multiplet or unresolved, br = broad signal, coupling constant(s) in Hz. ^{13}C NMR spectra were recorded with ^{1}H-decoupling and are reported with the solvent resonance as the reference unless noted otherwise (CD_3CN at 1.32 ppm, $CDCl_3$ at 77.16 ppm, CD_3OD at 49.00 ppm, CD_2Cl_2 at 54.00 ppm, DMSO-d_6 at 39.52 ppm). For ^{19}F NMR, an appropriately calibrated tertiary reference was employed, calibrated according to the literature method for improved reproducibility where 0.00 ppm represents the signal of the second isotopologue of neat $CFCl_3$.[i] IR spectra were recorded on a Thermo Fischer Scientific Nicolet 6700 FTIR equipped with a PIKE technologies GladiATRTM or on a Perkin-Elmer BX II using ATR FT-IR technology. The peaks are reported as absorption maxima (cm^{-1}). High-resolution mass spectrometric data were obtained at the mass spectrometry service operated by the Laboratory of Organic Chemistry at the ETHZ on VG-TRIBRID for electron impact ionization (EI), Varian IonSpec Spectrometer for electrospray ionization (ESI), or IonSpec Ultima Fourier Transform Mass Spectrometer for matrix-assisted laser desorption/ionization (MALDI) and are reported as (*m*/*z*). Melting points were determined on a Büchi Melting Point B-540 apparatus in open capillaries. UV-Vis spectroscopy was obtained with a Jasco V-630 Spectrophotometer. Photoluminescence spectroscopy was obtained with an Aminco Bowman Series 2 Luminescence Spectrometer with experiment and instrument setups described in the respective sections.

X-ray crystallography: Single crystals for X-ray diffraction were measured on XtaLAB Synergy, Dualflex, Pilatus 300K diffractometer with CuKα radiation (λ = 1.54184Å). All measurements were carried out at 100 K. The structures were solved using SHELXS, SHELXT5 or Superflip and refined by full-matrix least-squares analysis (SHELXL) using the program package OLEX2. Unless otherwise indicated below, all non-hydrogen atoms were refined anisotropically and hydrogen atoms were constrained to ideal geometries and refined with fixed isotropic displacement parameters (in terms of a riding model). The crystallographic data can be obtained free of charge from The Cambridge Crystallographic Data Center. Solid-state structures were visualized using the ORTEP3 program.

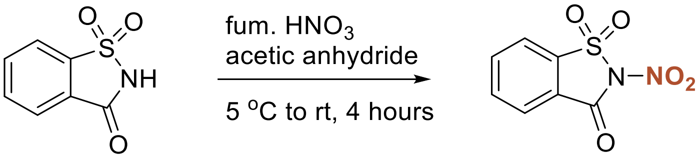

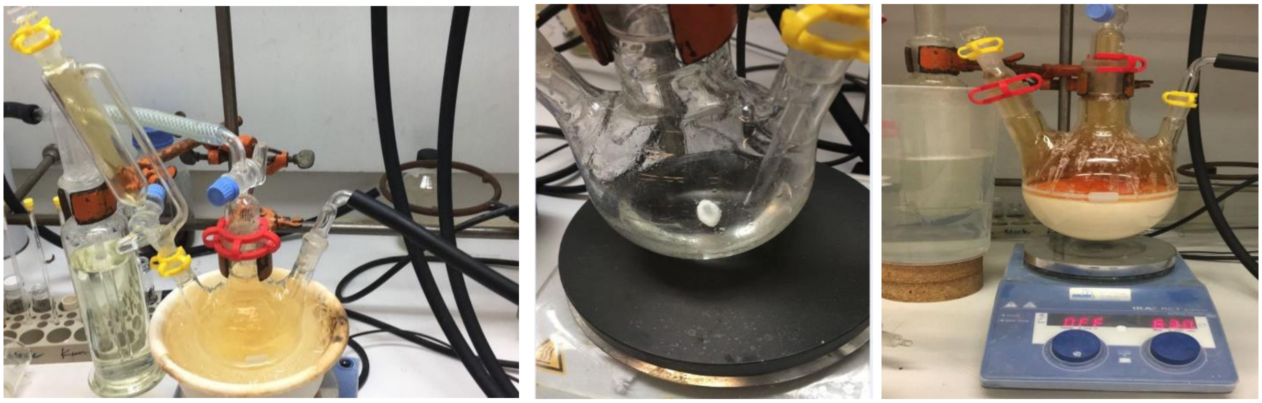

Supplementary Figure 1. Representative procedure for synthesis of reagent **4a**. Left: addition of nitric acid; middle: saccharin dissolved; right: reaction after 4 hours.

Representative procedure for synthesis of reagent 4a: In a 250 mL three necked round bottom flask equipped with a dropping funnel, air outlet and stirring bar was placed N-saccharin (10.0 g, 54.64 mmol) in acetic anhydride (25.7 mL, 0.27 mol). The solution was cooled to 0-5 °C with an ice-bath and fuming concentrated nitric acid (25.1 mL, 0.61 mol) was added dropwise to the solution during 30 minutes, while dry air was bubbled through the solution rapidly in order to remove excess nitrogen oxides. N-Saccharin completely dissolved once all nitric acid was added. The cooling bath was removed and the reaction mixture was stirred at room temperature during at least 4 hours with continuous bubbling of air through the liquid. The precipitate which had formed during the reaction was collected on a sintered glass filter and dried under high vacuum until dryness (11.8 g, 95% yield). The mother liquor was quenched with a cold solution of 1N NaOH. The material can be recrystallized from hot chloroform or acetonitrile and is a white crystalline compound.

The sensitivity of reagent **4a** was tested by hammer blow and with the drop-weight impact machine. The hammer test is an initial indication of the sensitivity of the molecule to an outside impact stimulus. Reagent **4a** in amount of 1 gr were placed on a clean steel surface of a witness plate and hit with a hammer (250 gram). No fume, heavy smoke, sparks, explosion, or heat were recorded, suggesting that this molecule is shock insensitive. For the fall-hammer test MP-3 Falling Hammer equipment was used. Sample of **4a** (200 mg) was placed on a clean steel surface and a 1 kg hammer was raised to a predetermined height (0.5 m and 0.8 m) by a manual crank. The hammer was dropped from various height onto the striker. No effects have been recorded.

^{1}H NMR (300 MHz, CD_3CN): δ = 8.05 (dt, *J* = 7.4, 1.5 Hz, 1H), 8.14 (dt, *J* = 6.1, 1.4 Hz, 1H), 8.16-8.23 (m, 2H); **^{13}C NMR** (75 MHz): δ = 121.7, 123.1, 126.5, 134.4, 135.9, 137.6, 151.7; **IR** (ATR, neat): 3097, 1781, 1717, 1601, 1463, 1292, 1176, 1068, 1007, 891, 758, 662, 582, 500; **HRMS** (EI) *m/z* calc'd for $C_7H_4N_2O_5S$: [M^+] 227.9836, found 227.9842; **Anal. calcd.** for $C_7H_4N_2O_5S$: C 36.85, H 1.77, N 12.28 found: C 36.88, H 1.87, N 12.41.

Although we encountered no incidents while synthesizing this molecule or products reported herein, safety precautions must be taken such as wearing safety glasses, protected shield, full body protective clothing, etc.

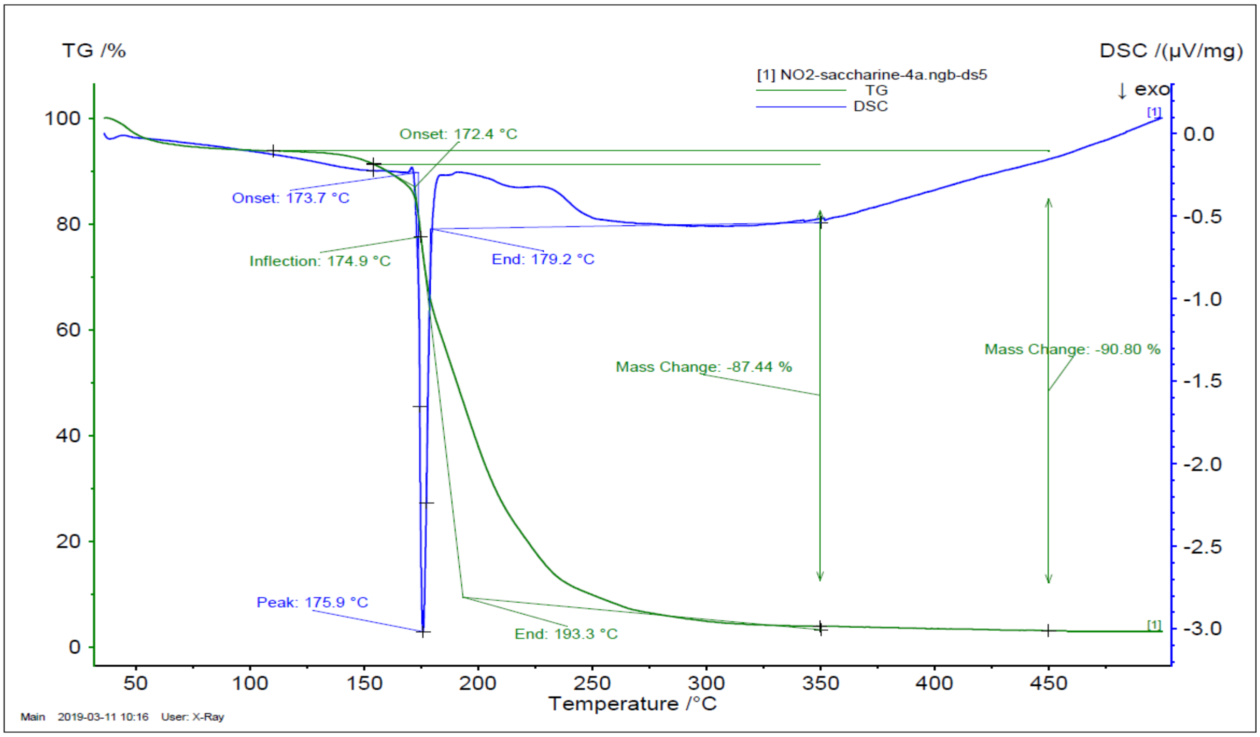

Supplementary Figure 2. TGA-DSC profile of nitrating reagent **4a**.

TGA-DSC profile of nitrating reagent 4a: Thermogravimetric and differential scanning calorimetry (TGA-DSC) measurments were performed in order to determine the melting point and decomposition temperature of **4a**. Reagent **4a** shows an exothermic decomposition at 173 °C, accompained with a mass loss of 87% .

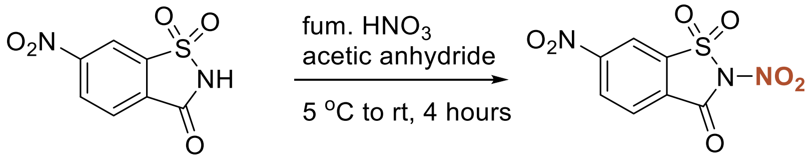

Supplementary Figure 3. Representative procedure for synthesis of reagent **4b**.

Representative procedure for synthesis of reagent 4b: In a 250 mL three necked round bottom flask equipped with a dropping funnel, air outlet and stirring bar was placed 6-nitrosaccharin (10.0 g, 36.63 mmol) in acetic anhydride (28.2 mL, 0.30 mol). The solution was cooled 5-10 °C with an ice-bath and concentrated fuming nitric acid (28.2 mL, 0.67 mol) was added dropwise to the solution during 30 minutes, while dry air was bubbled through the solution rapidly in order to remove excess nitrogen oxides. 6-Nitrosaccharin was

completely dissolved once all nitric acid was added. The reaction mixture was stirred at 5-10 °C during 4 hours with constant bubbling of dry air through the liquid. The reaction mixture was placed in the freezer for 10 hours to complete precipitation of the product. The precipitate was collected on a sintered glass filter, washed with cold chloroform and dried under high vacuum until dryness (9.6 g, 96% yield). The mother liquor was quenched with a cold solution of 1N NaOH. The product is a light-yellow (off-white) crystalline compound.

^{1}H NMR (500 MHz, CD_3CN): δ = 9.07 (d, *J* = 2.1 Hz, 1H), 8.76 (dd, *J* = 8.5, 2.0 Hz, 1H), 8.43 (d, *J* = 8.4 Hz, 1H); **^{13}C NMR** (125 MHz, CD_3CN): δ = 118.3, 127.8, 128.5, 130.6, 135.4, 150.3, 152.9; **IR** (ATR, neat): 3073, 1732, 1601, 1529, 1424, 1347, 1180, 1064, 1024, 786, 737, 649, 490; **Anal. calcd.** for $C_7H_3N_3O_7S$ C 30.78, H 1.11, N 15.38 found: C 30.81, H 1.19, N 15.50.

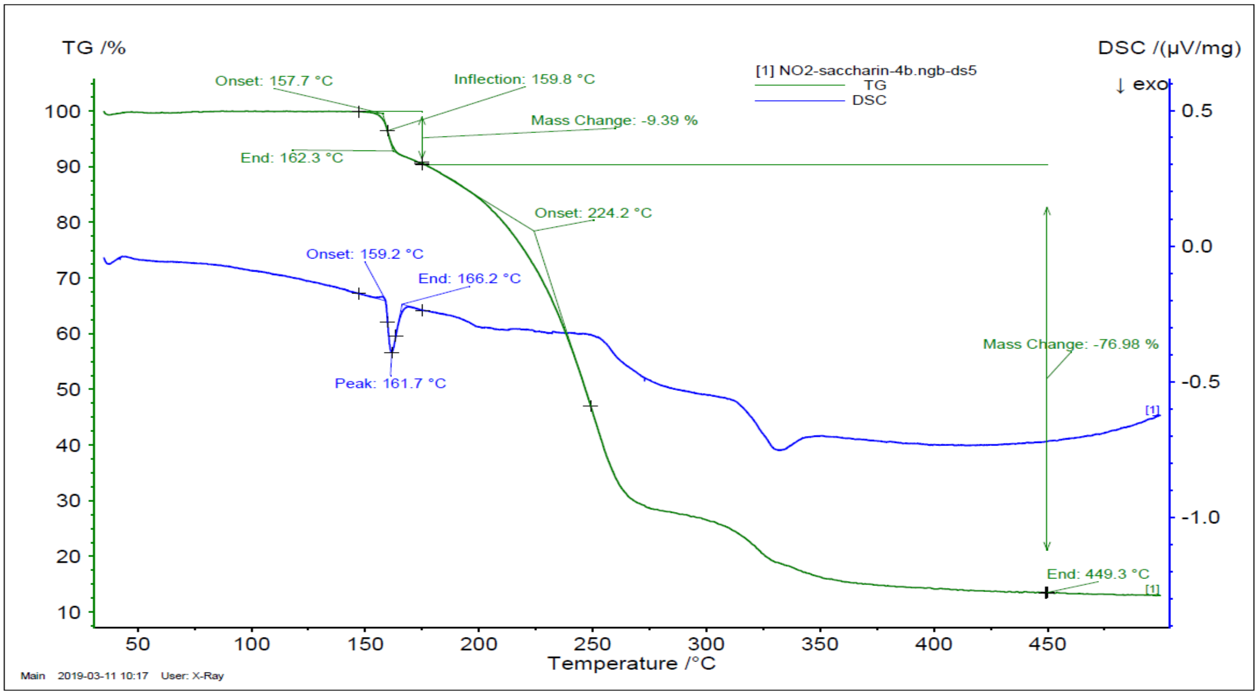

Supplementary Figure 4. TGA-DSC profile of nitrating reagent **4b**.

TGA-DSC profile of nitrating reagent 4b:TGA-DSC measurments were performed on reagent **4b**, which was found to be stable unil 159 °C, whereby an exothermic event accompanied by a 9.4% mass loss was observed. This was followed by gradual decomposition between 166 °C - 450 °C.

Supplementary Table 1. Stability of *N*-nitrosaccharins **4** in different solvents.

Entry	Solvent	Decomposition after 10 min[a]	Decomposition after 24 h[a]
1	Benzene-d_6	no	no
2	Toluene-d_8	no	no
3	Dichloromethane-d_2	no	no
4	Chloroform-d_1	no	no
5	DCE-d_4	no	no
6	THF-d_8	no	no
7	Acetone-d_6	no	no
8	Acetonitrile-d_3	no	no
9	HFIP-d_2	no	no
10	NMP-d_6	no	no
11	Cyclohexane-d_{12}	no	no
12	Methanol-d_4	partial	complete
13	DMF-d_7	partial	complete
14	DMSO-d_6	complete	complete
15	Water-d_2	[b]	complete

[a] Determined by ^{1}H NMR. [b] Not determined due to poor solubility.

Stability of *N*-nitrosaccharins 4 in different solvents: Reagents **4a** and **4b** were found to be stable in a variety of apolar and polar aprotic solvents including benzene, toluene, dichloromethane, chloroform, dichloroethane, tetrahydrofuran, acetone, acetonitrile, hexafluoroisopropanol (HFIP), *N*-methyl-2-pyrrolidinone (NMO) and cyclohexane, showing no signs of decomposition (Supplementary Table **1**, entries 1-11). The reagents are not compatible with the use of highly polar solvents such as MeOH, DMF and DMSO and water, with hydrolysis and other decomposition products detected after 10 minutes or 24 hours (entries 12-15).

Supplementary Table 2. Optimization of the reaction conditions I.

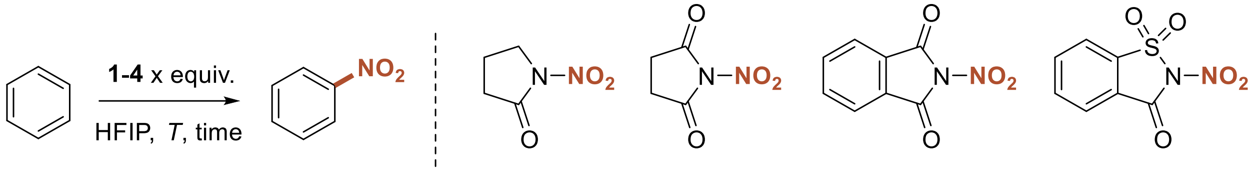

Entry	**[NO₂]** (x equiv.)	**C** [M]	**Temp** (°C)	**Time**	**Yield** (%)[a]
1	1 (1.7)	0.14	85	40 min	0
2	2 (1.7)	0.14	85	40 min	0
3	3 (1.7)	0.14	85	40 min	4
4	4a (1.7)	0.14	85	40 min	99
2	4a (1.7)	0.14	60	1 h	99
3	4a (1.7)	0.14	55	3 h	99
4	4a (1.7)	0.14	50	5 h	95
5	4a (1.7)	0.14	40	8 h	90
6	4a (1.5)	0.14	55	3	99
7	4a (1.3)	0.14	55	3	99
8	4a (1.2)	0.14	55	3	97
9	4a (1.1)	0.14	55	3	91
10	4a (1.3)	0.28	55	3	99
11	4a (1.3)	0.56	55	3	99 [b]
12	4a (1.3)	0.84	55	3	95

[a] *Reaction conditions*: benzene (1.0 equiv), reagent (x equiv), solvent, Ar atmosphere, temperature. Yields were determined by GC-MS using decane as internal standard. [b] Isolated yield.

Optimization of the reaction conditions I: An oven-dried, 25 mL micro-vial was charged on the benchtop with a magnetic pTFE-coated stirbar and nitrating reagent **1-4** (x equiv.). The vial was sealed and the atmosphere was cycled three times with Ar/vac. Benzene (44.69 µL, 0.5 mmol, 1.0 equiv.) in HFIP (1 mL) was added with a plastic syringe and the reaction mixture was heated at 40-85 °C with vigorous stirring for 1-8 hours. An internal standard of *n*-decane (97 µL, 0.5 mmol, 1.0 equiv.) was added with a microsyringe. An aliquot was analyzed by GC-MS to obtain the calibrated yield of nitrobenzene.

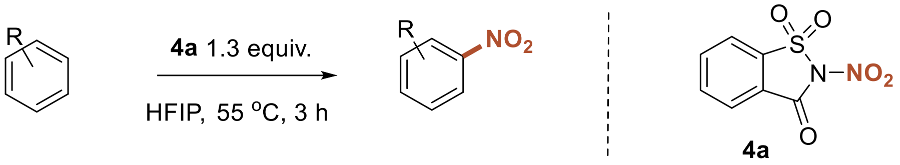

Supplementary Figure 5. General procedure **I** for the nitration of (hetero)arenes.

General procedure I for the nitration of (hetero)arenes: An oven-dried, 25 mL micro-vial was charged on the benchtop with a magnetic pTFE-coated stirbar and **4a** (148 mg, 0.65 mmol, 1.3 equiv.). The vial was sealed and the atmosphere was cycled 3x with Ar/vac. Hetero(arene) substrate (0.5 mmol, 1.0 equiv.) and HFIP (1 mL) were added and the reaction mixture was heated at 55 °C with vigorous stirring for 3 hours. After cooling to room temperature, the solvent was removed under reduced pressure, and the product was purified by flash column chromatography (SiO_2, ethyl acetate/n-hexane gradient).

Supplementary Table 3. Optimization of the reaction conditions I.

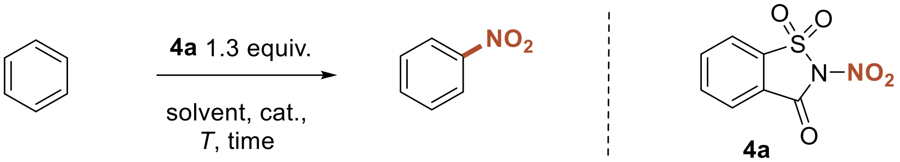

**Entry**	**Solvent**[c]	**Catalyst** (10 mol%)	**Temp** (°C)	**Time** (h)	**Yield** (%)[a]
1	CH_3CN	-	85	19 h	75
2	CH_3NO_2	-	85	19 h	95
3	CF_3CH_2OH	-	85	19 h	99
4	$CF_3CF_2CH_2OH$	-	85	19 h	99
5	$(CF_3)_2CHOH$	-	85	19 h	99
6	CH_3CN+air	-	85	19 h	70
7	CH_3CN+CH_3NO_2	-	85	19 h	99
8	DMF	-	85	19 h	5
9	DCE	-	85	19 h	15
10	MeOH/iPrOH	-	85	19 h	-
11	CH_3CN	TFA	85	19 h	85
12	CH_3CN	Acetic acid	85	19 h	79
13	CH_3CN	$Fe(OTf)_2$	85	19 h	72
14	CH_3CN	$Fe(OTf)_3$	85	19 h	28
15	CH_3CN	$FeBr_3$	85	19 h	80
16	CH_3CN	$FeBr_2$	85	19 h	47
17	CH_3CN	$FeCl_2$	85	19 h	41
18	CH_3CN	$VO(acac)_2$	85	19 h	25
19	CH_3CN	$NiBr_2$	85	19 h	43

20	CH_3CN	$Cu(OTf)_2$	85	19 h	86
21	CH_3CN	$MgCl_2$	85	19 h	59
22	CH_3CN	$Mg(NO_3)_2\ 6H_2O$	85	19 h	61
23	CH_3CN	$Mg(ClO_4)_2$	85	19 h	>99
24	CH_3CN	$Mg(ClO_4)_2$	85	5 h	>99[b]
25	CH_3CN	$Mg(ClO_4)_2$	85	3 h	90
26	CH_3CN	$MgNTf_2$	85	19 h	97
27	CH_3CN	$MgNTf_2$	85	5 h	97
28	CH_3CN	$ZnNTf_2$	85	19 h	>99
29	CH_3CN	$ZnNTf_2$	85	5 h	90
30	CH_3CN	$ZnCl_2$	85	19 h	21
31	CH_3CN	$AlCl_3$	85	19 h	9

[a] *Reaction conditions*: **benzene** (1.0 equiv), **4a** (1.3 equiv), 10 mol% catalyst, solvent, C[M] 0.54, Ar atmosphere, temperature. Yields were determined by GC-MS using decane as internal standard. [b] Isolated yield. [c] Other solvents such as DMF, 1,4 –dioxane, NMP, DMA, DMSO were found to be inefficient and led to the formation of trace amounts of nitrobenzene.

Optimization of the reaction conditions I: An oven-dried, 25 mL micro-vial was charged on the benchtop with a magnetic pTFE-coated stirbar, nitrating reagent **4a** (148 mg, 0.65 mmol, 1.3 equiv.) and catalyst (0.05 mmol, 10 mol%). The vial was sealed and the atmosphere was cycled 3x with Ar/vac. Benzene (44.69 μL, 0.5 mmol, 1.0 equiv.) and solvent (1 mL) were added with a plastic syringe, and the reaction mixture was heated at 85 °C with vigorous stirring for 3-19 hours. An internal standard of *n*-decane (97 μL, 0.5 mmol, 1.0 equiv.) was added with a microsyringe. An aliquot was analyzed by GC-MS to obtain the calibrated yield of nitrobenzene.

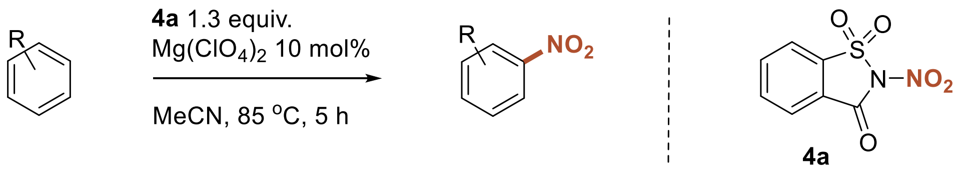

Supplementary Figure 6. General procedure **II** for the nitration of (hetero)arenes.

General procedure II for the nitration of (hetero)arenes: An oven-dried, 25 mL micro-vial was charged on the benchtop with a magnetic pTFE-coated stirbar, nitrating reagent **4a** (148 mg, 0.65 mmol, 1.3 equiv.) and $Mg(ClO_4)_2$ (11.2 mg, 0.05 mmol, 10 mol%). The vial was sealed and the atmosphere was cycled 3x with Ar/vac. Hetero(arene) substrate (0.5 mmol, 1.0 equiv.) in MeCN (1 mL) was added with a plastic syringe, and the reaction mixture was heated at 85 °C with vigorous stirring for 5 hours. After cooling to room temperature, the solvent was removed under reduced pressure, and the product was purified by flash column chromatography (SiO_2, ethyl acetate/n-hexane gradient). **Note**: Li, Mg, Zn and Ni perchlorates are not dangerous chemicals if not employed under extreme acidic conditions and not exposed to high temperatures (>300−500 °C) (*40-41*).

Nitrobenzene (**5**) [CAS: 98-95-3]

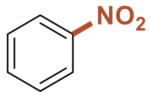

Yield 99%, 61 mg (using the general procedure I); yield 99% (using the general procedure II); yellow oil; **^{1}H NMR** (300 MHz, $CDCl_3$) δ 8.16 (d, *J* = 7.6 Hz, 2H), 7.68 (t, *J* = 7.6 Hz, 1H), 7.52 (m, 2H); **^{13}C NMR** (101 MHz, $CDCl_3$) δ 147.9, 134.5, 129.1, 123.2.

1-Fluoro-4-nitrobenzene (**6A**) [CAS: 350-46-9] and 1-fluoro-2-nitrobenzene (**6B**) [CAS: 1493-27-2]

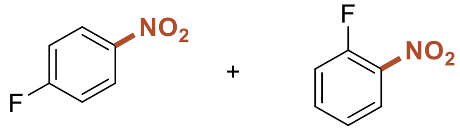

(4 : 1)

Yield of **6A** 76.8%, 54 mg (using the general procedure I); light yellow solid; **mp** 109-112 °C; **^{1}H NMR** (300 MHz, $CDCl_3$) δ 8.55 – 8.08 (m, 2H), 7.64 – 6.86 (m, 2H); **^{13}C NMR** (75 MHz, $CDCl_3$) δ 166.3 (d, *J* = 257.9 Hz), 144.4, 126.3 (d, *J* = 10.0 Hz), 116.4 (d, *J* = 23.7 Hz); **^{19}F NMR** (282 MHz, $CDCl_3$) δ -102.00.

Yield of **6B** 19.2%, 13.5 mg (using the general procedure I); yellow oil; **^{1}H NMR** (300 MHz, $CDCl_3$) δ 8.23 – 8.05 (m, 1H), 7.76 – 7.60 (m, 1H), 7.54 – 7.25 (m, 2H); **^{13}C NMR** (75 MHz, $CDCl_3$) δ 155.6 (d, *J* = 264.8 Hz), 137.5, 135.6 (d, *J* = 8.6 Hz), 126.2 (d, *J* = 2.8 Hz), 124.6 (d, *J* = 4.4 Hz), 118.5 (d, *J* = 20.6 Hz); **^{19}F NMR** (282 MHz, $CDCl_3$) δ -117.65.

1-Chloro-4-nitrobenzene (**7A**) [CAS: 100-00-5] and 1-chloro-2-nitrobenzene (**7B**) [CAS: 88-73-3]

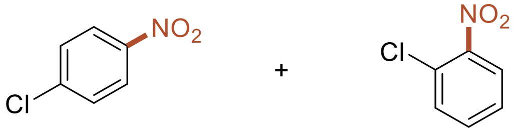

(1.5 : 1)

Yield of **7A** 57.6%, 45.3 mg (using the general procedure I); yellow solid; **mp** 82-84 °C; **^{1}H NMR** (300 MHz, $CDCl_3$) δ 8.19 (d, *J* = 9.0 Hz, 2H), 7.52 (d, *J* = 9.0 Hz, 2H); **^{13}C NMR** (75 MHz, $CDCl_3$) δ 146.6, 141.4, 129.6, 124.9.

Yield of **7B** 38.4%, 30.2 mg (using the general procedure I); yellow solid; **mp** 32-33 °C; **^{1}H NMR** (300 MHz, $CDCl_3$) δ 7.87 (dd, *J* = 8.0, 1.6 Hz, 1H), 7.63 – 7.47 (m, 2H), 7.42 (ddd, *J* = 8.7, 6.9, 2.0 Hz, 1H); **^{13}C NMR** (75 MHz, $CDCl_3$) δ 148.3, 133.2, 131.9, 127.6, 127.1, 125.6.

1-Iodo-4-nitrobenzene (**8A**) [639-98-6] and 1-iodo-2-nitrobenzene (**8B**) [CAS: 609-73-4]

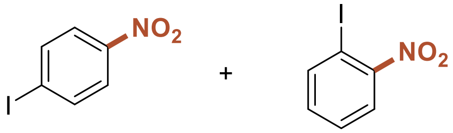

(2.1 : 1)

Yield of **8A** 63%, 78.4 mg (using the general procedure I); yellow solid; **mp** 53-55°C; **^{1}H NMR** (300 MHz, $CDCl_3$) δ 7.93 (d, *J* = 9.0 Hz, 2H), 7.92 (d, *J* = 9.0 Hz, 2H); **^{13}C NMR** (75 MHz, $CDCl_3$) δ 147.8, 138.7, 124.9, 102.7.

Yield of **8B** 30%, 37.3 mg (using the general procedure I); yellow oil; **^{1}H NMR** (300 MHz, $CDCl_3$) δ 7.98 (dd, *J* = 7.9, 1.3 Hz, 1H), 7.79 (dd, *J* = 8.1, 1.5 Hz, 1H), 7.42 (td, *J* = 7.8, 1.3 Hz, 1H), 7.33 – 7.11 (m, 1H); **^{13}C NMR** (75 MHz, $CDCl_3$) δ 153.1, 141.9, 133.4, 129.1, 125.5, 86.2.

1-Bromo-4-nitrobenzene (**9A**) [CAS: 586-78-7] and 1-bromo-2-nitrobenzene (**9B**) [CAS: 577-19-5]

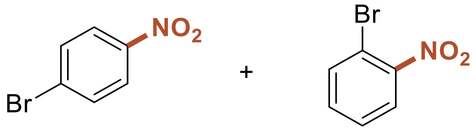

(1.5 : 1)

Yield of **9A**, 55.2%, 55.7 mg (using the general procedure I); yellow solid; **mp** 123-125 °C; **^{1}H NMR** (300 MHz, $CDCl_3$) δ 8.10 (d, *J* = 9.0 Hz, 2H), 7.69 (d, *J* = 9.0 Hz, 2H); **^{13}C NMR** (75 MHz, $CDCl_3$) δ 147.1, 132.7, 130.0, 125.0.

Yield of **9B** 36.8%, 37 mg (using the general procedure I); yellow solid; **mp** 42-44 °C; **^{1}H NMR** (300 MHz, $CDCl_3$) δ 7.84 (dd, *J* = 7.5, 2.3 Hz, 1H), 7.75 (dt, *J* = 8.3, 1.9 Hz, 1H), 7.56 – 7.37 (m, 2H); **^{13}C NMR** (75 MHz, $CDCl_3$) δ 149.9, 135.1, 133.2, 128.3, 125.6, 114.5.

1-Methyl-4-nitrobenzene (**10A**) [CAS: 99-99-0] and 1-methyl-2-nitrobenzene (**10B**) [CAS: 88-72-2]

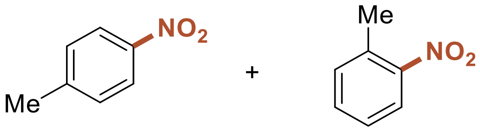

(1.1 : 1)

Yield of **10A** 49.7%, 34 mg (using the general procedure I); light yellow solid; **mp** 51-53 °C; **^{1}H NMR** (300 MHz, $CDCl_3$) δ 8.09 (d, *J* = 8.6 Hz, 2H), 7.31 (d, *J* = 7.4 Hz, 2H), 2.45 (s, 3H); **^{13}C NMR** (75 MHz, $CDCl_3$) δ 146.2, 145.99, 129.8, 123.5, 21.6.

Yield of **10B** 45.3%, 31 mg (using the general procedure I); yellow oil; **^{1}H NMR** (300 MHz, $CDCl_3$) δ 7.95 (d, *J* = 8.2 Hz, 1H), 7.49 (t, *J* = 7.4 Hz, 1H), 7.40 – 7.27 (m, 2H), 2.59 (s, 3H); **^{13}C NMR** (75 MHz, $CDCl_3$) δ 149.3, 133.5, 133.0, 132.8, 126.9, 124.6, 20.4.

1-(Tert-butyl)-4-nitrobenzene (**11A**) [CAS: 3282-56-2] and 1-(tert-butyl)-2-nitrobenzene (**11B**) [CAS: 1886-57-3]

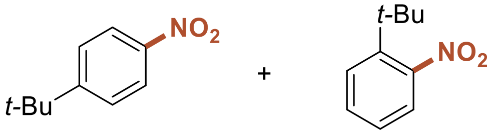

(9 : 1)

Yield of **11A** 84.6%, 75.8 mg (using the general procedure I); light yellow solid; **mp** 99-102 °C; **^{1}H NMR** (300 MHz, $CDCl_3$) δ 8.50 – 7.99 (m, 2H), 7.86 – 7.34 (m, 2H), 1.36 (s, 9H); **^{13}C NMR** (75 MHz, $CDCl_3$) δ 158.88, 145.96, 126.3, 123.4, 35.4, 31.1.

Yield of **11B** 9.4%, 8.4 mg (using the general procedure I); yellow oil; **^{1}H NMR** (300 MHz, $CDCl_3$) δ 7.58 – 7.44 (m, 1H), 7.36 (ddd, *J* = 8.2, 6.3, 2.4 Hz, 1H), 7.30 – 7.12 (m, 2H), 1.34 (s, 9H); **^{13}C NMR** (75 MHz, $CDCl_3$) δ 151.3, 141.3, 130.8, 128.6, 126.9, 123.9, 35.7, 30.7.

1-Methoxy-4-nitrobenzene (**12A**) [CAS: 100-17-4] and 1-methoxy-2-nitrobenzene (**12B**) [CAS: 91-23-6]

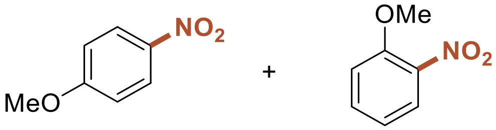

(1.85 : 1)

Yield of **12A** 61%, 46.7 mg (using the general procedure I); light yellow solid; **mp** 52-54 °C; **^{1}H NMR** (300 MHz, $CDCl_3$) δ 8.51 – 8.08 (m, 2H), 7.21 – 6.80 (m, 2H), 3.98 (s, 3H); **^{13}C NMR** (75 MHz, $CDCl_3$) δ 164.6, 141.6, 125.9, 114.1, 56.0.

Yield of **12B** 33%, 25 mg (using the general procedure I); yellow oil; **^{1}H NMR** (300 MHz, $CDCl_3$) δ 7.83 (dd, *J* = 8.1, 1.7 Hz, 1H), 7.54 (ddd, *J* = 8.4, 7.4, 1.7 Hz, 1H), 7.20 – 6.92 (m, 2H), 3.95 (s, 3H); **^{13}C NMR** (75 MHz, $CDCl_3$) δ 153.0, 134.2, 125.7, 120.3, 113.6, 56.5.

1-Nitro-4-(trifluoromethoxy)benzene (**13A**) [CAS: 713-65-5] and 1-nitro-2-(trifluoromethoxy)benzene (**13B**) [CAS: 1644-88-8]

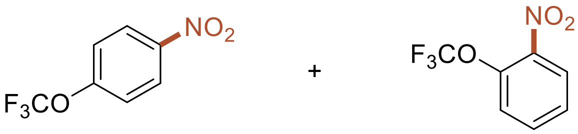

(4.8 : 1)

Yield of **13A** 71.2%, 74 mg (using the general procedure I, 19 hours); yellow oil; yield 79.7%, 82.5 mg (using the general procedure II, 19 hours); **^{1}H NMR** (300 MHz, $CDCl_3$) δ 8.59 – 8.02 (m, 2H), 7.64 – 7.27 (m, 2H); **^{13}C NMR** (75 MHz, $CDCl_3$) δ 153.7 (q, *J* = 1.6 Hz), 146.0, 125.8, 121.0, 120.2 (q, *J* = 258.7 Hz); **^{19}F NMR** (282 MHz, $CDCl_3$) δ -57.81.

Yield of **13B** 14.8%, 15 mg (using the general procedure I, 19 hours); yellow oil; yield 17.3%, 18 mg (using the general procedure II, 19 hours); **^{1}H NMR** (300 MHz, $CDCl_3$) δ 7.99 (dd, *J* = 8.4, 1.7 Hz, 1H), 7.67 (td, *J* = 7.8, 1.7 Hz, 1H), 7.56 – 7.39 (m, 2H); **^{13}C NMR** (75 MHz, $CDCl_3$) δ 142.8, 141.3 (q, *J* = 2.0 Hz), 134.2, 127.6, 125.9, 123.3, 120.2 (q, *J* = 260.6 Hz); **^{19}F NMR** (282 MHz, $CDCl_3$) δ -57.58.

1-Cyclopropyl-4-nitrobenzene (**14A**) [CAS: 6921-44-4] and 1-Cyclopropyl-2-nitrobenzene (**14B**) [CAS: 10292-65-6]

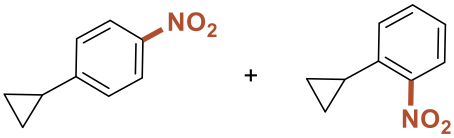

(2.1 : 1)

Yield of **14A**, 65.7%, 54 mg (using the general procedure I); light yellow oil; **^{1}H NMR** (300 MHz, $CDCl_3$) δ 8.42 – 7.91 (m, 2H), 7.26 – 6.96 (m, 2H), 2.24 – 1.74 (m, 1H), 1.27 – 1.03 (m, 2H), 0.94 – 0.69 (m, 2H); **^{13}C NMR** (75 MHz, $CDCl_3$) δ 152.6, 145.9, 125.9, 123.7, 15.9, 11.0.

Yield of **14B** 31.3%, 25.5 mg (using the general procedure I); white solid, **mp** 33-34 °C; **^{1}H NMR** (300 MHz, $CDCl_3$) δ 7.87 (d, *J* = 8.1 Hz, 1H), 7.54 (t, *J* = 7.6 Hz, 1H), 7.43 – 7.29 (m, 1H), 7.23 (d, *J* = 7.8 Hz, 1H), 2.46 (ddd, *J* = 13.9, 8.5, 5.4 Hz, 1H), 1.23 – 1.01 (m, 2H), 0.83 – 0.70 (m, 2H); **^{13}C NMR** (75 MHz, $CDCl_3$) δ 151.2, 138.1, 132.6, 127.9, 126.4, 124.1, 12.5, 8.1.

(4-Nitrophenyl)(trifluoromethyl)sulfane (**15A**) [CAS: 403-66-7] and (2-nitrophenyl)(trifluoromethyl)-sulfane (**15B**) [CAS: 1644-87-7]

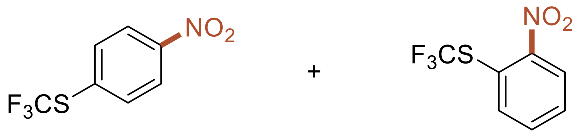

(1.2 : 1)

Yield of **15A** 45%, 50 mg (using the general procedure I, 19 hours); yield 52.9%, 59 mg (using the general procedure II); yellow oil; **^{1}H NMR** (400 MHz, $CDCl_3$) δ 8.40 – 8.12 (m, 2H), 7.83 (d, *J* = 8.7 Hz, 2H); **^{13}C NMR** (101 MHz, $CDCl_3$) δ 149.2, 136.1, 132.6 (q, *J* = 2.0 Hz), 128.9 (q, *J* = 308.8 Hz), 124.3; **^{19}F NMR** (376 MHz, $CDCl_3$) δ -41.34.

Yield of **15B** 41%, 45.6 mg (using the general procedure I, 19 hours); yield 44.1%, 49.2 mg (using the general procedure II); yellow oil; **^{1}H NMR** (400 MHz, $CDCl_3$) δ 8.12 (dd, *J* = 8.2, 1.4 Hz, 1H), 7.84 (d, *J* = 8.0 Hz, 1H), 7.68 (td, *J* = 7.8, 1.4 Hz, 1H), 7.55 (td, *J* = 7.9, 1.2 Hz, 1H); **^{13}C NMR** (101 MHz, $CDCl_3$) δ 149.3, 133.7, 132.72 – 131.91 (m), 129.4, 188.7 (q, *J* = 311.0 Hz), 125.7, 124.37 – 124.06 (m); **^{19}F NMR** (376 MHz, $CDCl_3$) δ -41.23.

N,N-Dimethyl-4-nitroaniline (**16A**) [CAS: 100-23-2] and N,N-dimethyl-2-nitroaniline (**16B**) [CAS: 610-17-3]

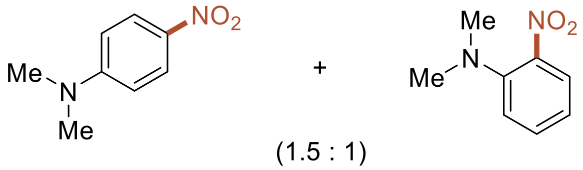

(1.5 : 1)

Yield of **16A** 54.6%, 45.3 mg (using the general procedure I); yellow solid; **mp** 162-164 °C; **^{1}H NMR** (300 MHz, $CDCl_3$) δ 8.10 (d, *J* = 9.4 Hz, 2H), 6.59 (d, *J* = 9.4 Hz, 2H), 3.10 (s, 6H); **^{13}C NMR** (75 MHz, $CDCl_3$) δ 154.3, 136.9, 126.1, 110.3, 40.3.

Yield of **16B** 36.4%, 30.2 mg (using the general procedure I); yellow oil; **^{1}H NMR** (300 MHz, $CDCl_3$) δ 8.70 (d, *J* = 2.7 Hz, 1H), 8.21 (dd, *J* = 9.5, 2.7 Hz, 1H), 7.01 (d, *J* = 9.4 Hz, 1H), 3.06 (s, 6H); **^{13}C NMR** (75 MHz, $CDCl_3$) δ 149.2, 136.6, 135.8, 127.8, 124.2, 116.6, 42.4.

1-Nitro-3-(trifluoromethyl)benzene (**17A**) [CAS: 98-46-4] and 1-nitro-2-(trifluoromethyl)benzene (**17B**) [CAS: 384-22-5]

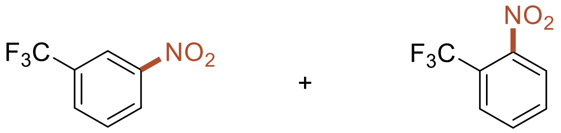

(4.9 : 1)

Yield of **17A** 59.8%, 57 mg (using the general procedure I, 19 hours); yellow oil; yield 72%, 69 mg (using the general procedure II); **^{1}H NMR** (300 MHz, $CDCl_3$) δ 8.50 (d, *J* = 1.9 Hz, 1H), 8.44 (dd, *J* = 8.3, 2.2 Hz, 1H), 7.98 (d, *J* = 7.8 Hz, 1H), 7.74 (t, *J* = 8.0 Hz, 1H); **^{13}C NMR** (75 MHz, $CDCl_3$) δ 148.3, 132.4 (q, *J* = 34.1 Hz), 131.2 (q, *J* = 3.5 Hz), 130.4, 126.7 (d, *J* = 0.75 Hz), 122.7 (q, *J* = 270.7 Hz), 120.9 (q, *J* = 3.9 Hz); **^{19}F NMR** (282 MHz, $CDCl_3$) δ -62.95.

Yield of **17B** 12.2%, 12 mg (using the general procedure I, 19 hours); yellow oil; yield 15%, 14 mg (using the general procedure II); **^{1}H NMR** (300 MHz, $CDCl_3$) δ 7.95 – 7.78 (m, 2H), 7.80 – 7.67 (m, 2H); **^{13}C NMR** (75 MHz, $CDCl_3$) δ 148.3, 133.1, 132.6, 127.9 (q, *J* = 5.2 Hz), 125.0, 123.8 (q, *J* = 33.7 Hz), 122.1 (q, *J* = 271.5 Hz); **^{19}F NMR** (282 MHz, $CDCl_3$) δ -60.01.

3-Nitrobenzonitrile (**18**) [CAS: 619-24-9]

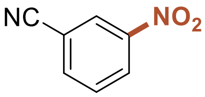

Yield 81%, 60 mg (using the general procedure I, 19 hours); yield 92%, 68 mg (using the general procedure II); yellow solid; **mp** 115-117 °C; **^{1}H NMR** (300 MHz, $CDCl_3$) δ 8.53 (t, *J* = 1.9 Hz, 1H), 8.48 (ddd, *J* = 8.3, 2.4, 1.2 Hz, 1H), 8.00 (dt, *J* = 7.7, 1.4 Hz, 1H), 7.74 (t, *J* = 8.0 Hz, 1H); **^{13}C NMR** (75 MHz, $CDCl_3$) δ 148.3, 137.6, 130.7, 127.6, 127.3, 116.6, 114.2.

Methyl 3-nitrobenzoate (**19A**) [CAS: 618-95-1] and methyl 2-nitrobenzoate (**19B**) [CAS: 606-27-9]

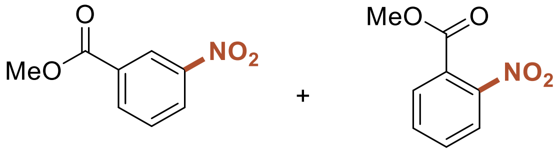

(4 : 1)

Yield of **19A** 69.6%, 63 mg (using the general procedure II); yellow crystalline compound; **mp** 77-79 °C; **^{1}H NMR** (300 MHz, $CDCl_3$) δ 8.86 (t, *J* = 2.0 Hz, 1H), 8.39 (ddt, *J* = 14.1, 7.8, 1.3 Hz, 2H), 7.65 (t, *J* = 8.0 Hz, 1H), 3.99 (s, 3H); **^{13}C NMR** (75 MHz, $CDCl_3$) δ 164.9, 148.3, 135.3, 131.3, 129.7, 127.4, 124.6, 52.8.

Yield of **19B** 17.4%, 15.7 mg (using the general procedure II); yellow oil; **^{1}H NMR** (300 MHz, $CDCl_3$) δ 7.91 (dd, *J* = 7.4, 1.9 Hz, 1H), 7.83 – 7.72 (m, 1H), 7.65 (pd, *J* = 7.4, 1.7 Hz, 2H), 3.92 (s, 3H); **^{13}C NMR** (75 MHz, $CDCl_3$) δ 165.9, 148.3, 132.9, 131.8, 129.9, 127.6, 123.9, 53.3.

4-Nitrobenzophenone (**20A**) [CAS: 1144-74-7], 3-nitrobenzophenone (**20B**) [CAS: 2243-80-3] and 2-Nitrobenzophenone (**20C**) [CAS: 2243-79-0]

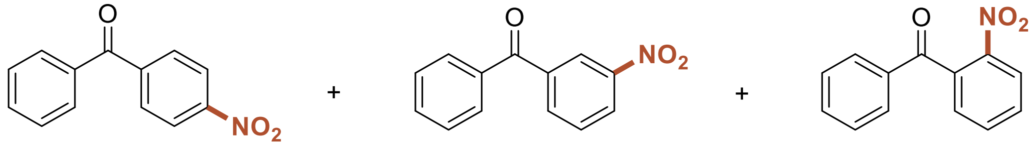

(0.6 : 1 : 0.8)

Yield of **20A** 24.5%, 28 mg (using the general procedure I, 19 hours); light yellow solid; **mp** 136-139 °C; **^{1}H NMR** (400 MHz, $CDCl_3$) δ 8.35 (d, *J* = 8.6 Hz, 2H), 7.94 (d, *J* = 8.6 Hz, 2H), 7.80 (d, *J* = 8.3 Hz, 2H), 7.66 (t, *J* = 7.4 Hz, 1H), 7.53 (t, *J* = 7.8 Hz, 2H); **^{13}C NMR** (101 MHz, $CDCl_3$) δ 194.8, 149.8, 142.9, 136.3, 133.5, 130.7, 130.1, 128.7, 123.5.

Yield of **20B** 40.8%, 46.3 mg (using the general procedure I, 19 hours); light yellow solid; **mp** 94-96 °C; **^{1}H NMR** (400 MHz, $CDCl_3$) δ 8.62 (t, *J* = 1.8 Hz, 1H), 8.45 (ddd, *J* = 8.2, 2.2, 1.0 Hz, 1H), 8.14 (dt, *J* = 7.6, 1.2 Hz, 1H), 7.87 – 7.76 (m, 2H), 7.71 (t, *J* = 7.9 Hz, 1H), 7.66 (t, *J* = 7.4 Hz, 1H), 7.53 (t, *J* = 7.7 Hz, 2H), **^{13}C NMR** (101 MHz, $CDCl_3$) δ 194.2, 148.1, 139.1, 136.3, 135.4, 133.4, 130.0, 129.6, 128.7, 126.7, 124.7.

Yield of **20C** 32.7%, 37 mg (using the general procedure I, 19 hours); light yellow solid; **mp** 104-106 °C; **^{1}H NMR** (400 MHz, $CDCl_3$) δ 8.24 (d, *J* = 8.2 Hz, 1H), 7.76 (dd, *J* = 11.4, 8.0 Hz, 3H), 7.68 (t, *J* = 7.8 Hz, 1H), 7.59 (t, *J* = 7.4 Hz, 1H), 7.54 – 7.41 (m, 3H); **^{13}C NMR** (101 MHz, $CDCl_3$) δ 193.4, 146.7, 136.2, 135.9, 134.2, 133.8, 130.5, 129.2, 128.9, 128.8, 124.5.

4-Nitro-1,1'-biphenyl (**21A**) [CAS: 92-93-3] and 2-nitro-1,1'-biphenyl (**21B**) [CAS: 86-00-0]

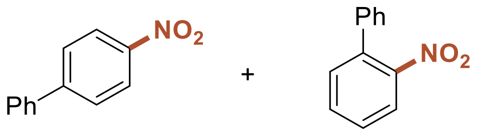

(1 : 1.5)

Yield of **21A** 39.6%, 39.5 mg (using the general procedure I); yellow solid; **mp** 113-114 °C; **^{1}H NMR** (300 MHz, $CDCl_3$) δ 8.30 (d, *J* = 8.8 Hz, 2H), 7.74 (d, *J* = 8.8 Hz, 2H), 7.69 – 7.58 (m, 2H), 7.58 – 7.39 (m, 3H); **^{13}C NMR** (75 MHz, $CDCl_3$) δ 147.7, 147.2, 138.8, 129.2, 128.9, 127.8, 127.4, 124.2.

Yield of **21B** 59.4%, 59 mg (using the general procedure I); yellow solid; **mp** 36-38 °C; **^{1}H NMR** (300 MHz, $CDCl_3$) δ 7.86 (d, *J* = 8.0 Hz, 1H), 7.62 (td, *J* = 7.5, 1.1 Hz, 1H), 7.55 – 7.38 (m, 5H), 7.38 – 7.29 (m, 2H); **^{13}C NMR** (75 MHz, $CDCl_3$) δ 149.4, 137.4, 136.4, 132.3, 132.0, 128.7, 128.3, 128.2, 127.9, 124.1.

1-Nitronaphthalene (**22**) [CAS: 86-57-7]

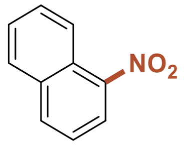

Yield 94%, 81 mg (using the general procedure I); light yellow solid; **mp** 56-59 °C; **^{1}H NMR** (300 MHz, $CDCl_3$) δ 8.57 (d, *J* = 8.7 Hz, 1H), 8.24 (d, *J* = 7.6 Hz, 1H), 8.12 (d, *J* = 8.2 Hz, 1H), 7.96 (d, *J* = 8.2 Hz,

1H), 7.73 (ddd, J = 8.6, 6.9, 1.5 Hz, 1H), 7.63 (t, J = 7.5 Hz, 1H), 7.55 (t, J = 7.9 Hz, 1H); ^{13}**C NMR** (75 MHz, $CDCl_3$) δ 134.7, 134.4, 129.5, 128.6, 127.4, 125.2, 124.2, 124.0, 123.2.

9-Nitroanthracene (**23**) [CAS: 602-60-8]

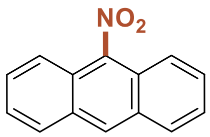

Yield 92%, 103 mg (using the general procedure I); yellow solid; **mp** 145-147 °C; ^{1}**H NMR** (300 MHz, $CDCl_3$) δ 8.55 (s, 1H), 8.02 (d, J = 8.4 Hz, 2H), 7.93 (dd, J = 8.8, 1.1 Hz, 2H), 7.62 (ddd, J = 8.7, 6.7, 1.3 Hz, 2H), 7.52 (ddd, J = 8.0, 6.7, 1.1 Hz, 2H); ^{13}**C NMR** (75 MHz, $CDCl_3$) δ 144.30, 130.8, 130.4, 128.9, 128.4, 126.2, 122.7, 121.4.

Methyl 4-(tert-butyl)-3-nitrobenzoate (**24A**) [CAS: 91641-96-2] and methyl 4-(tert-butyl)-2-nitrobenzoate (**24B**) [CAS: 20587-31-9]

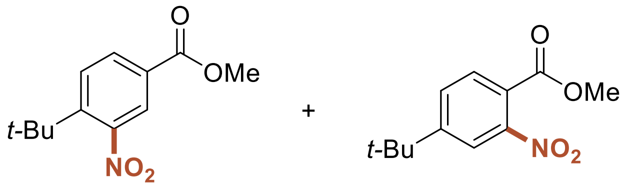

(1 : 2.5)

Yield of **24A** 62.1%, 74 mg (using the general procedure I, 19 hours); yellow oil; yield 67.2%, 79.8 mg (using the general procedure II); ^{1}**H NMR** (400 MHz, $CDCl_3$) δ 8.07 (dd, J = 8.4, 1.9 Hz, 1H), 7.97 (d, J = 1.9 Hz, 1H), 7.64 (d, J = 8.4 Hz, 1H), 3.93 (s, 3H), 1.42 (s, 9H); ^{13}**C NMR** (101 MHz, $CDCl_3$) δ 164.9, 151.1, 146.2, 131.4, 129.1, 129.0, 125.1, 52.6, 36.1, 30.5.

Yield of **24B** 24.9%, 29.5 mg (using the general procedure I, 19 hours); light yellow oil; yield 26.8%, 32 mg (using the general procedure II); ^{1}**H NMR** (400 MHz, $CDCl_3$) δ 7.86 (d, J = 1.7 Hz, 1H), 7.73 – 7.61 (m, 2H), 3.90 (s, 3H), 1.36 (s, 9H); ^{13}**C NMR** (101 MHz, $CDCl_3$) δ 165.8, 156.5, 148.6, 129.8, 129.7, 124.3, 120.9, 53.1, 35.4, 30.9.

2-Fluoro-5-nitrobenzaldehyde (**25**) [CAS: 27996-87-8]

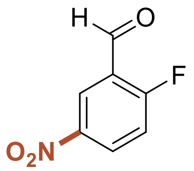

Yield 75%, 63.4 mg (using the general procedure I, 19 hours); yield 84%, 71 mg (using the general procedure II); pale-orange solid; **mp** 58-60°C; ^{1}**H NMR** (300 MHz, $CDCl_3$) δ 10.37 (s, 1H), 8.74 (dd, J = 5.9, 2.9 Hz, 1H), 8.49 (ddd, J = 9.0, 4.4, 2.9 Hz, 1H), 7.40 (t, J = 9.0 Hz, 1H); ^{13}**C NMR** (75 MHz, $CDCl_3$) δ 184.8 (d, J = 5.9 Hz), 167.3 (d, J = 268.4 Hz), 144.9, 131.0 (d, J = 11.0 Hz), 124.9 (d, J = 4.1 Hz), 124.6 (d, J = 10.5 Hz), 118.3 (d, J = 23.2 Hz); ^{19}**F NMR** (282 MHz, $CDCl_3$) δ -111.19 (dt, J = 9.9, 5.2 Hz); IR (ATR, neat): 3076, 2899, 1693, 1619, 1523, 1470, 1346, 1226, 1070, 934, 744, 547.

2,6-Dihydroxy-3-nitrobenzoic acid (**26**)

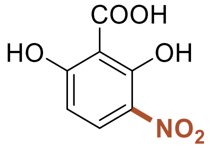

Yield 88%, 88 mg (using the general procedure I); yellow solid; **mp** 215.5-217.0 °C; **^{1}H NMR** (400 MHz, C_3D_6O) δ 14.95 (s, 1H), 8.00 (d, *J* = 9.4 Hz, 1H), 6.24 (d, *J* = 9.4 Hz, 1H), 3.45 (bs, 2H); **^{13}C NMR** (101 MHz, C_3D_6O) δ 176.8, 169.7, 162.7, 131.1, 129.4, 106.6, 104.5; **IR** (ATR, neat): 3436, 1718, 1595, 1450, 1249, 1145, 922, 824, 755, 590; **HRMS** (ESI+) calcd (m/z) for $C_7H_4NO_6$: [M-H] 198.0044; found 198.0049.

4-Chloro-3-nitrophenol (**27**) [CAS: 610-78-6]

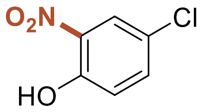

Yield 93%, 81 mg (using the general procedure I); yellow solid; **mp** 126 °C; **^{1}H NMR** (300 MHz, $CDCl_3$) δ 10.47 (s, 1H), 8.11 (d, *J* = 2.6 Hz, 1H), 7.54 (dd, *J* = 9.0, 2.6 Hz, 1H), 7.14 (d, *J* = 9.0 Hz, 1H); **^{13}C NMR** (75 MHz, $CDCl_3$) δ 153.7, 137.6, 125.3, 124.4, 121.5.

4-Chloro-2-nitro-1-(trifluoromethoxy)benzene (**28A**) [CAS: 448-38-4] and 1-chloro-2-nitro-4-(trifluoromethoxy)benzene (**28B**) [CAS: 588-09-0]

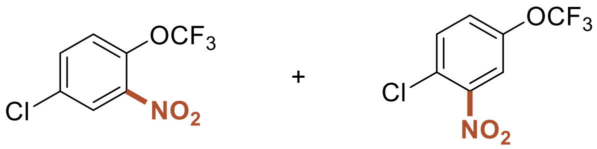

(1 : 0.6)

Yield of **28A** 57.5%, 69 mg (using the general procedure I, 19 hours); colorless oil; yield 60.6%, 73 mg (using the general procedure II); **^{1}H NMR** (500 MHz, $CDCl_3$) δ 7.99 (d, *J* = 2.5 Hz, 1H), 7.64 (dd, *J* = 8.9, 2.6 Hz, 1H), 7.42 (dq, *J* = 8.8, 1.4 Hz, 1H); **^{13}C NMR** (126 MHz, $CDCl_3$) δ 142.9, 139.8, 134.2, 133.4, 126.1, 124.5, 120.1 (q, *J* = 261.4 Hz); **^{19}F NMR** (471 MHz, $CDCl_3$) δ -57.75.

Yield of **28B** 34.5%, 41.6 mg (using the general procedure I, 19 hours); colorless oil; yield 36.4%, 44 mg (using the general procedure II); **^{1}H NMR** (500 MHz, $CDCl_3$) δ 7.78 (d, *J* = 2.8 Hz, 1H), 7.62 (d, *J* = 8.9 Hz, 1H), 7.41 (ddd, *J* = 8.9, 2.9, 1.0 Hz, 1H); **^{13}C NMR** (126 MHz, $CDCl_3$) δ 148.1, 147.5 (q, *J* = 2.3 Hz), 133.2, 125.6, 125.5, 120.1 (q, *J* = 260.3 Hz), 118.50; **^{19}F NMR** (471 MHz, $CDCl_3$) δ -58.30.

1,3-Dichloro-2,4-dinitrobenzene (**29**) [CAS: 10199-85-6]

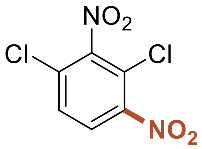

Yield 37%, 44 mg (using the general procedure I, 19 hours); yield 79%, 94 mg (using the general procedure II); yellow solid; **mp** 70-72 °C; **^{1}H NMR** (400 MHz, $CDCl_3$) δ 8.03 (dd, *J* = 8.9, 1.0 Hz, 1H), 7.65 (dd, *J* = 8.9, 1.0 Hz, 1H); **^{13}C NMR** (101 MHz, $CDCl_3$) δ 149.5, 146.7, 130.9, 129.5, 126.7, 121.5.

2-Bromo-1,3,5-trimethyl-4-nitrobenzene (**30**) [CAS: 90561-85-6]

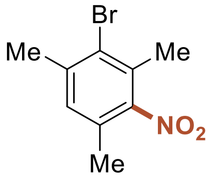

Yield 96%, 117.2 mg (using the general procedure I); yellow solid; **mp** 58-62 °C; **^{1}H NMR** (300 MHz, $CDCl_3$) δ 7.10 (s, 1H), 2.49 (s, 3H), 2.43 (s, 3H), 2.29 (s, 3H); **^{13}C NMR** (75 MHz, $CDCl_3$) δ 150.7, 140.6, 130.3, 129.6, 127.7, 125.5, 24.0, 18.9, 17.0.

1,3,5-Trimethoxy-2-nitrobenzene (**31**) [CAS: 14227-18-0]

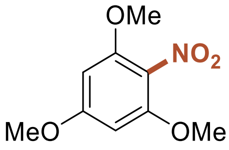

Yield 93%, 99 mg (using the general procedure I); yellow crystalline compound; **mp** 151-153 °C; **^{1}H NMR** (300 MHz, $CDCl_3$) δ 6.11 (s, 2H), 3.85 (s, 6H), 3.83 (s, 3H); **^{13}C NMR** (75 MHz, $CDCl_3$) δ 162.2, 153.4, 126.6, 90.8, 56.4, 55.7.

N-(*o*-Nitrophenyl)succinimide (**32A**) [CAS: 18377-52-1] and N-(p-Nitrophenyl)succinimide (**32B**) [CAS: 35488-92-7]

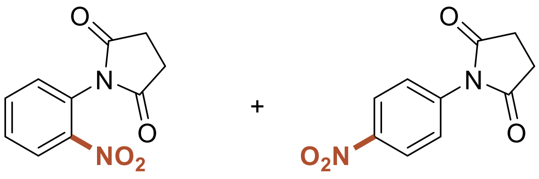

(1.1 : 1)

Yield of **32A** 51.3%, 56.4 mg (using the general procedure I, 19 hours); yield 51%, 56 mg (using the general procedure II); yellow solid; **mp** 157-158 °C; **^{1}H NMR** (300 MHz, $CDCl_3$) δ 8.18 (d, *J* = 8.2 Hz, 1H), 7.76 (t, *J* = 7.7 Hz, 1H), 7.61 (t, *J* = 7.9 Hz, 1H), 7.39 (d, *J* = 7.9 Hz, 1H), 2.98 (d, *J* = 15 Hz, 2H), 2.92 (d, *J* = 15 Hz, 2H); **^{13}C NMR** (75 MHz, $CDCl_3$) δ 175.4, 145.3, 134.4, 130.6, 130.2, 126.2, 126.0, 28.9.

Yield of **32B** 42.7%, 47 mg (using the general procedure I, 19 hours); yield 46%, 50.6 mg (using the general procedure II); yellow solid; **mp** 208-209 °C; **^{1}H NMR** (300 MHz, $CDCl_3$) δ 8.41 (d, *J* = 8.9 Hz, 2H), 7.67 (d, *J* = 8.9 Hz, 2H), 3.03 (s, 4H); **^{13}C NMR** (75 MHz, $CDCl_3$) δ 175.3, 147.2, 137.5, 127.0, 124.5, 28.6.

2-Methyl-5-nitroisoindoline-1,3-dione (**33**)

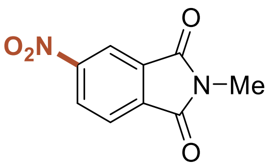

Yield 86%, 87 mg (using the general procedure II); yellow solid; **mp** 179-180 °C; ^{1}**H NMR** (300 MHz, CD_3CN) δ 8.58 (dd, *J* = 8.1, 1.9 Hz, 1H), 8.53 (d, *J* = 2.0 Hz, 1H), 8.03 (d, *J* = 8.1 Hz, 1H), 3.15 (s, 3H); ^{13}**C NMR** (75 MHz, CD_3CN) δ 166.2, 165.9, 151.5, 136.6, 133.4, 129.0, 123.8, 117.6, 23.4.

1-Chloro-4-((4-chlorophenyl)sulfonyl)-2-nitrobenzene (**34**) [CAS: 41890-38-4]

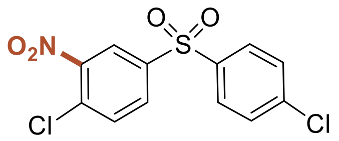

Yield 72%, 119.5 mg (using the general procedure II); white solid; **mp** 59-61 °C; ^{1}**H NMR** (400 MHz, C_2D_6SO) δ 8.68 (d, *J* = 2.2 Hz, 1H), 8.26 (dd, *J* = 8.5, 2.3 Hz, 1H), 8.10 – 8.00 (m, 3H), 7.78 – 7.71 (m, 2H); ^{13}**C NMR** (101 MHz, C_2D_6SO) δ 148.4, 141.0, 140.2, 138.8, 134.1, 132.6, 131.4, 130.6, 130.4, 125.4.

Pinacol 3-nitrobenzeneboronate (**35A**) [CAS: 68716-48-3] and pinacol 2-nitrobenzeneboronate (**35B**) [CAS: 190788-59-1]

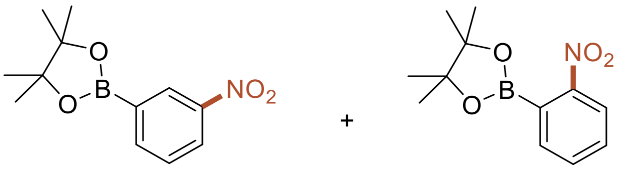

(1 : 0.9)

Yield of **35A** 42.6%, 53 mg (using the general procedure I); yellow solid; **mp** 73-74 °C; ^{1}**H NMR** (300 MHz, $CDCl_3$) δ 8.73 – 8.55 (m, 1H), 8.33 – 8.24 (m, 1H), 8.09 (d, *J* = 7.3 Hz, 1H), 7.53 (t, *J* = 7.8 Hz, 1H), 1.36 (s, 12H); ^{13}**C NMR** (75 MHz, $CDCl_3$) δ 147.9, 140.7, 129.4, 128.8, 125.8, 84.6, 24.9.

Yield of **35B** 38.4%, 47.6 mg (using the general procedure I); orange oil; ^{1}**H NMR** (300 MHz, $CDCl_3$) δ 8.14 (d, *J* = 8.1 Hz, 1H), 7.71 – 7.60 (m, 1H), 7.60 – 7.46 (m, 2H), 1.42 (s, 12H); ^{13}**C NMR** (75 MHz, $CDCl_3$) δ 151.0, 133.7, 132.9, 130.1, 123.0, 84.6, 24.8.

2,6-Dichloro-3-nitropyridine (**36**) [CAS: 16013-85-7]

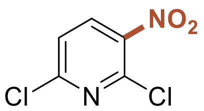

Yield 65%, 64.5 mg (using the general procedure II); yellow solid; **mp** 53-55 °C; ^{1}**H NMR** (300 MHz, CD_3CN) δ 8.36 (d, *J* = 8.4 Hz, 1H), 7.63 (d, *J* = 8.5 Hz, 1H); ^{13}**C NMR** (75 MHz, CD_3CN) δ 152.4, 143.5, 142.1, 137.3, 124.3, 116.9.

2,6-Dimethyl-3-nitropyridine (**37**) [CAS: 15513-52-7]

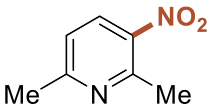

Yield 67%, 51 mg (using the general procedure II); yellow solid; **mp** 36-38 °C; ^{1}**H NMR** (300 MHz, $CDCl_3$) δ 8.15 (d, *J* = 8.3 Hz, 1H), 7.14 (d, *J* = 8.3 Hz, 1H), 2.80 (s, 3H), 2.59 (s, 3H); ^{13}**C NMR** (75 MHz, $CDCl_3$) δ 163.0, 153.3, 143.7, 132.9, 121.4, 24.7, 24.1.

2,4-Dichloro-5-nitropyrimidine (**38**) [CAS: 49845-33-2]

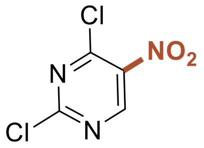

Yield 59%, 57 mg (using the general procedure II); yellow solid; **mp** 30-32 °C; **^{1}H NMR** (400 MHz, CD_3CN) δ 9.25 (s, 1H); **^{13}C NMR** (101 MHz, CD_3CN) δ 161.7, 157.5, 155.2, 142.2.

2,4-Dichloro-6-methyl-5-nitropyrimidine (**39**) [CAS: 13162-26-0]

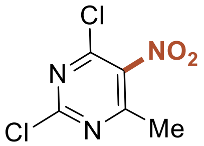

Yield 56%, 58 mg (using the general procedure II); yellow solid; **mp** 50-52 °C; **^{1}H NMR** (300 MHz, CD_3CN) δ 2.59 (d, J = 1.0 Hz, 3H), **^{13}C NMR** (75 MHz, CD_3CN) δ 164.7, 158.5, 152.7, 143.2.

1-(5-Nitrofuran-2-yl)ethan-1-one (**40**) [CAS: 5275-69-4]

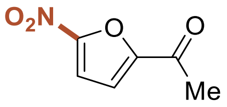

Yield 77%, 59.7 mg (using the general procedure I); yellow solid; **mp** 78-79 °C; **^{1}H NMR** (300 MHz, $CDCl_3$) δ 7.30 (d, J = 3.8 Hz, 1H), 7.20 (d, J = 3.8 Hz, 1H), 2.54 (s, 3H); **^{13}C NMR** (75 MHz, $CDCl_3$) δ 186.8, 151.9, 151.5, 116.7, 111.9, 26.3.

2,3-Bimethyl-5-nitrothiophene (**41**) [CAS: 87650-06-4]

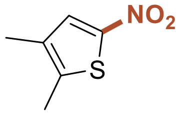

Yield 82%, 65 mg (using the general procedure I), yellow solid; **mp** 89-90 °C; **^{1}H NMR** (400 MHz, $CDCl_3$) δ 7.64 (s, 1H), 2.40 (s, 3H), 2.16 (s, 3H); **^{13}C NMR** (101 MHz, $CDCl_3$) δ 147.1, 143.2, 134.1, 131.2, 14.0, 13.7.

Cyclopropyl(5-nitrothiophen-2-yl)methanone (**42**) [CAS: 1330049-33-6]

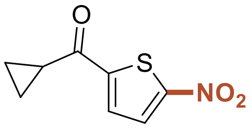

Yield 84%, 83 mg (using the general procedure I, 19 hours); white solid; **mp** 104-105 °C; **^{1}H NMR** (300 MHz, $CDCl_3$) δ 8.52 (d, J = 1.4 Hz, 1H), 8.29 (d, J = 1.5 Hz, 1H), 2.55 (tt, J = 7.7, 4.5 Hz, 1H), 1.35 – 1.24 (m, 2H), 1.21 – 1.08 (m, 2H); **^{13}C NMR** (75 MHz, $CDCl_3$) δ 192.2, 145.4, 132.9, 125.1, 17.9, 12.4.

(S)-(+)-Ibuprofen methyl ester-NO_2 (**43**)

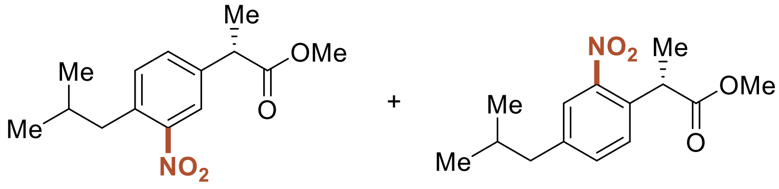

43A (2.5 : 1) **43B**

Yield of two isomers (**43A+43B**) 89%, 118 mg (using the general procedure I, 19 hours); yellow oil; **^{1}H NMR of mixture** (400 MHz, $CDCl_3$) δ 7.80 (d, *J* = 1.9 Hz, 2.5H), 7.70 (s, 1H), 7.44 (dd, *J* = 8.0, 2.0 Hz, 2.5H), 7.36 (d, *J* = 1.1 Hz, 2H), 7.25 (d, *J* = 7.9 Hz, 2.5H), 4.28 (q, *J* = 7.2 Hz, 1H), 3.77 (q, *J* = 7.2 Hz, 2.5H), 3.68 (s, 7.5H), 3.66 (s, 3H), 2.75 (d, *J* = 7.1 Hz, 5H), 2.53 (d, *J* = 7.2 Hz, 2H), 1.89 (dt, *J* = 13.5, 6.8 Hz, 3.5H), 1.58 (d, *J* = 7.2 Hz, 3H), 1.53 (d, *J* = 7.3 Hz, 7.5H), 0.91 (dd, *J* = 6.6, 4.7 Hz, 21H); **^{13}C NMR** (101 MHz, $CDCl_3$) δ 174.1, 173.9, 149.9, 148.9, 142.4, 139.8, 135.3, 134.1, 133.1, 132.5, 131.6, 129.5, 125.2, 123.8, 52.4, 52.34, 44.7, 44.6, 41.5, 41.0, 30.1, 29.6, 22.6, 22.4, 18.5, 18.1; **IR** (ATR, neat): 2956, 1735, 1527, 1347, 1192, 1166, 1066, 854, 818, 678; **HRMS** (ESI+) calcd (m/z) for $C_{14}H_{19}NNaO_4$: [M-Na$^+$] 288.1200; found 288.1206.

Lidocaine-NO_2 (**44**) [CAS: 39942-49-9]

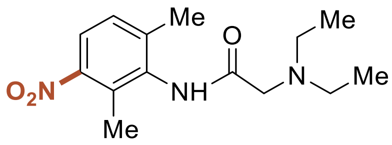

Yield 71%, 99 mg (using the general procedure I, 19 hours); yellow oil; **^{1}H NMR** (400 MHz, $CDCl_3$) δ 9.08 (s, 1H), 7.73 (d, *J* = 8.4 Hz, 1H), 7.20 (d, *J* = 8.4 Hz, 1H), 3.24 (s, 2H), 2.71 (q, *J* = 7.1 Hz, 4H), 2.38 (s, 3H), 2.29 (s, 3H), 1.14 (t, *J* = 7.1 Hz, 6H); **^{13}C NMR** (101 MHz, $CDCl_3$) δ 170.5, 148.9, 141.2, 135.9, 130.7, 128.1, 123.0, 57.4, 48.9, 19.2, 14.8, 12.6; **IR** (ATR, neat): 3260, 2969, 1673, 1518, 1485, 1343, 1290, 1203, 1088, 824, 747, 503; **HRMS** (ESI+) calcd (m/z) for $C_{14}H_{22}N_3O_3$: [M$^+$] 280.1652; found 280.1656.

(+)-(S)-Naproxen-NO_2 (**45**) [CAS: 847265-06-9]

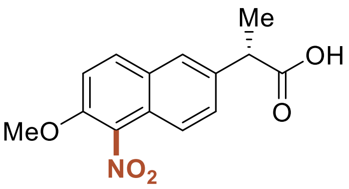

Yield 90%, 124 mg (using the general procedure I); yellow solid; **mp** 132-134 °C **^{1}H NMR** (400 MHz, C_2D_6SO) δ 12.42 (s, 1H), 8.22 (d, *J* = 9.2 Hz, 1H), 7.95 (d, *J* = 1.7 Hz, 1H), 7.69 (d, *J* = 9.2 Hz, 1H), 7.64 (dd, *J* = 8.8, 1.8 Hz, 1H), 7.56 (d, *J* = 8.8 Hz, 1H), 4.04 (s, 3H), 3.88 (q, *J* = 7.1 Hz, 1H), 1.47 (d, *J* = 7.1 Hz, 3H); **^{13}C NMR** (101 MHz, C_2D_6SO) δ 175.5, 148.6, 138.6, 135.3, 132.9, 130.1, 128.1, 126.8, 124.0, 120.2, 114.8, 57.7, 44.9, 18.6; **IR** (ATR, neat): 2945, 1722, 1608, 1518, 1359, 1281, 1214, 1163, 1076, 903, 818, 641; **HRMS** (ESI+) calcd (m/z) for $C_{14}H_{14}NO_5$: [M$^+$] 276.0868; found 276.0866.

Clofibrate-NO_2 (**46**)

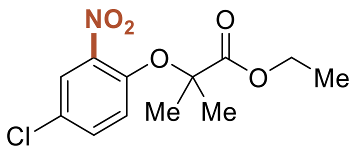

Yield 98%, 141 mg (using the general procedure I); light yellow oil; **^{1}H NMR** (300 MHz, $CDCl_3$) δ 7.74 (d, J = 2.5 Hz, 1H), 7.39 (dd, J = 9.0, 2.5 Hz, 1H), 6.95 (d, J = 9.0 Hz, 1H), 4.24 (q, J = 7.1 Hz, 2H), 1.64 (s, 6H), 1.26 (t, J = 7.1 Hz, 3H); **^{13}C NMR** (75 MHz, $CDCl_3$) δ 173.2, 147.6, 143.3, 132.8, 127.2, 125.2, 121.4, 82.1, 61.9, 25.1, 14.1; **IR** (ATR, neat): 2988, 1735, 1604, 1531, 1478, 1384, 1354, 1281, 1176, 1100, 1019, 882, 843, 655; **HRMS** (ESI+) calcd (m/z) for $C_{12}H_{14}NO_5ClNa$: [M+Na] 310.0449; found 310.0453.

Phenytoin-NO_2 (**47**)

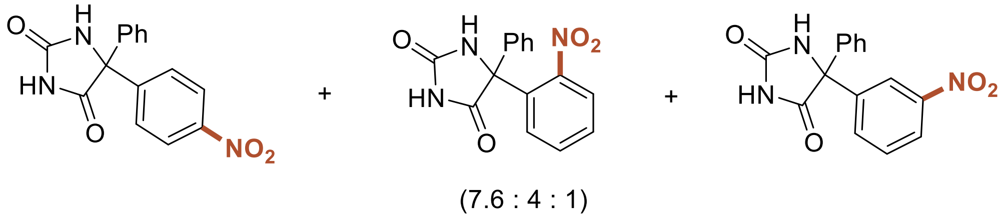

Total yield of isomers 98%, 145.5 mg (using the general procedure I); white solid, **mp** 235-240 °C; **^{1}H NMR of 47A** (500 MHz, C_2D_6SO) δ 11.3 (s, 1H), 9.51 (s, 1H), 8.29 (d, J= 10.0 Hz, 2H), 7.67 (d, J= 10.0 Hz, 2H); 7.43 – 7.34 (m, 5H); **^{1}H NMR of 47B** (500 MHz, C_2D_6SO) δ 11.3 (s, 1H), 9.5 (s, 1H), 8.30 −8.24 (m, 2H), 7.88 (d, J = 10.0 Hz, 1H), 7.74 (t, 10.0 Hz, 1H), 7.43 – 7.34 (m, 5H), **^{1}H NMR of 47C** (500 MHz, C_2D_6SO) δ 11.1 (s, 1H), 9.3 (s, 1H), 8.11 (d, J = 5.0 Hz, 1H), 7.97 (t, J = 5.0 Hz, 1H), 7.98 – 7.91 (m, J = 5.0 Hz, 3H), 7.43 – 7.34 (m, 5H). **^{13}C NMR of mixture** (125 MHz, C_2D_6SO) δ 175.3, 174.57, 174.41, 156.44, 156.27, 148.2, 147.6, 147.0, 142.0, 140.4, 135.55, 134.89, 133.8, 130.8, 129.3, 129.2, 128.95, 128.93, 129.91, 128.52, 128.47, 127.0, 126.9, 125.1, 123.2, 123.7, 121.5, 121.4, 70.6, 70.5, 70.1; **IR** (ATR, neat): 3048, 1771, 1714, 1519, 1347, 1225, 1095, 852, 691; **HRMS** (ESI+) calcd (m/z) for $C_{15}H_{11}N_3O_4Na$: [M+Na$^+$] 320.0642; found 320.0642.

Nimesulide-NO_2 (**48**)

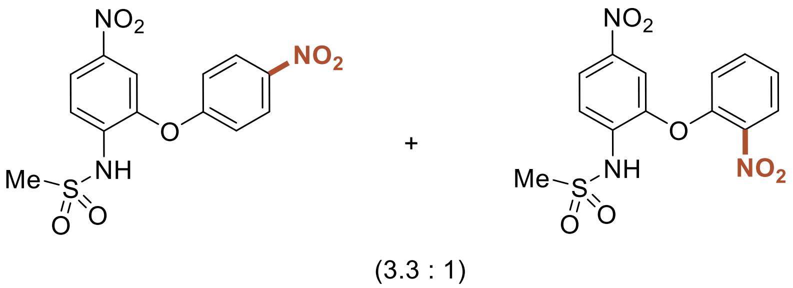

Yield of **48A** 76.1%, 134 mg (using the general procedure I); yellow solid, **mp** 177.2-178.0 °C; **^{1}H NMR** (400 MHz, $CDCl_3$) δ 10.36 (s, 1H), 8.54 – 8.24 (m, 2H), 8.18 (dd, J = 9.1, 2.6 Hz, 1H), 7.96 (d, J = 2.6 Hz, 1H), 7.82 (d, J = 9.1 Hz, 1H), 7.26 (d, J = 9.2 Hz, 2H), 3.18 (s, 3H); **^{13}C NMR** (101 MHz, $CDCl_3$) δ 166.67, 149.31, 148.33, 148.26, 142.28, 131.20, 126.79, 123.33, 121.74, 46.12; **IR** (ATR, neat): 3264, 1717, 1586, 1509, 1336, 1226, 1160, 965, 897, 744, 514; **HRMS** (ESI+) calcd (m/z) for $C_{13}H_{11}N_3NaO_7S$: [M+Na] 376.0212; found 376.0214.

Yield of **48B** 23%, 40.5 mg (using the general procedure I); yellow-red solid, **mp** 173.1-173.6 °C; **^{1}H NMR** (300 MHz, $CDCl_3$) δ 8.16 (dd, *J* = 8.2, 1.6 Hz, 1H), 8.06 (dd, *J* = 9.0, 2.4 Hz, 1H), 7.85 (d, *J* = 9.0 Hz, 1H), 7.77 (td, *J* = 8.2, 1.6 Hz, 1H), 7.60 (s, 1H), 7.57 (d, *J* = 2.4 Hz, 1H), 7.56 – 7.46 (m, 1H), 7.33 (dd, *J* = 8.2, 1.1 Hz, 1H), 3.19 (s, 3H); **^{13}C NMR** (75 MHz, $CDCl_3$) δ 147.1, 145.5, 143.6, 141.3, 135.7, 134.3, 127.1, 126.9, 123.4, 120.5, 117.9, 111.1, 40.5; **IR** (ATR, neat): 3263, 1599, 1517, 1336, 1267, 1186, 1160, 952, 738, 516; **HRMS** (ESI+) calcd (m/z) for $C_{13}H_{11}N_3NaO_7S$: [M+Na] 376.0212; found 376.0212.

Secinidazole (**49**) [CAS: 3366-95-8]

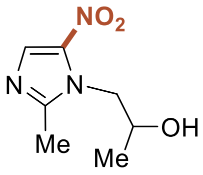

Yield 44%, 41 mg (using the general procedure II, 19 hours); white solid, **mp** 80.5-81.5 °C; **^{1}H NMR** (300 MHz, CD_3CN) δ 7.91 (s, 1H), 4.60 – 4.21 (m, 1H), 4.21 – 3.90 (m, 2H), 3.26 (s, 1H), 2.46 (s, 3H), 1.23 (d, *J* = 5.9 Hz, 3H); **^{13}C NMR** (101 MHz, CD_3CN) δ 151.6, 138.6, 132.3, 66.0, 52.4, 19.7, 13.7; **IR** (ATR, neat): 3503, 3136, 1526, 1447, 1388, 1352, 1181, 1083, 932, 839, 741, 490; **HRMS** (ESI+) calcd (m/z) for $C_7H_{12}N_3O_3$: [M+H] 186.0873; found 186.0877.

5,6-Dimethoxy-7-nitro-2,3-dihydro-1H-inden-1-one (**50**) [CAS: 66773-29-3]

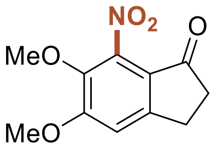

Yield 79%, 94 mg (using the general procedure I, 19 hours); yellow solid; **mp** 146-148 °C; **^{1}H NMR** (400 MHz, $CDCl_3$) δ 7.03 (s, 1H), 4.00 (s, 3H), 3.90 (s, 3H), 3.29 – 2.96 (m, 2H), 2.90 – 2.56 (m, 2H); **^{13}C NMR** (101 MHz, $CDCl_3$) δ 200.2, 159.3, 152.9, 140.5, 139.9, 120.5, 110.2, 62.5, 56.9, 36.8, 25.9.

5-Nitroveratraldehyde (**51**) [CAS: 22027-96-9]

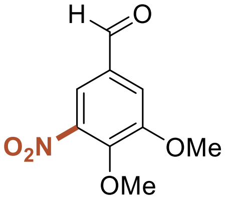

Yield 91%, 96 mg (using the general procedure I); light yellow solid; **mp** 61-62 °C; **^{1}H NMR** (300 MHz, Chloroform-*d*) δ 10.46 (s, 1H), 7.62 (s, 1H), 7.43 (s, 1H), 4.04 (s, 3H), 4.03 (s, 3H); **^{13}C NMR** (75 MHz, Chloroform-*d*) δ 187.8, 153.4, 152.6, 144.0, 125.7, 109.9, 107.3, 56.9, 56.9; **HRMS** (EI) calcd (m/z) for $C_9H_9NO_5$: [M^+] 211.04752; found 211.04744.

Ethyl 1-(4-nitrophenyl)-3-(trifluoromethyl)-1H-pyrazole-4-carboxylate (**52A**) and ethyl 1-(2-nitrophenyl)-3-(trifluoromethyl)-1H-pyrazole-4-carboxylate (**52B**)

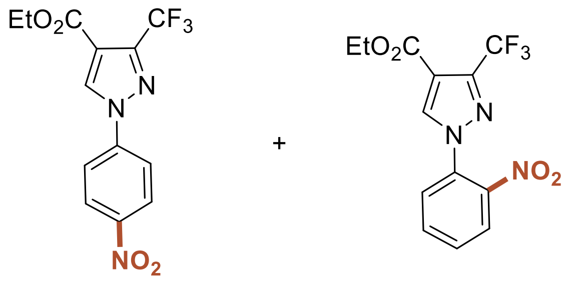

(8.5 : 1)

Yield of **52A** 83%, 136.5 mg (using the general procedure II); yellow solid; **mp** 115.3-116.5 °C; **^{1}H NMR** (400 MHz, $CDCl_3$) δ 8.41 (d, *J* = 9.0 Hz, 2H), 7.75 – 7.59 (m, 2H), 4.41 (q, *J* = 7.1 Hz, 2H), 1.41 (t, *J* = 7.1 Hz, 3H); **^{13}C NMR** (101 MHz, $CDCl_3$) δ 160.5, 148.2, 143.9, 143.3, 132.74(q, *J* = 40.5 Hz), 126.8, 124.6, 118.95 (q, *J* = 271.7 Hz), 117.9 (q, *J* = 1.1 Hz), 61.6, 14.1; **^{19}F NMR** (376 MHz, $CDCl_3$) δ -54.94; **IR** (ATR, neat): 1735, 1523, 1345, 1224, 1151, 1018, 970, 855, 756, 704; **HRMS** (ESI+) calcd (m/z) for $C_{13}H_{11}F_3N_3O_4$: [M+H] 330.0696; found 330.0697.

Yield of **52B** 10%, 16.5 mg (using the general procedure II); yellow solid; **mp** 119-120 °C; **^{1}H NMR** (400 MHz, $CDCl_3$) δ 8.23 (dd, *J* = 8.0, 1.7 Hz, 1H), 8.20 – 8.15 (m, 1H), 7.88 – 7.71 (m, 2H), 7.56 (dd, *J* = 7.7, 1.6 Hz, 1H), 4.41 (q, *J* = 7.1 Hz, 2H), 1.41 (t, *J* = 7.1 Hz, 3H); **^{13}C NMR** (101 MHz, $CDCl_3$) δ 160.5 (q, *J* = 1.0 Hz), 144.9, 143.5, 134.3 (q, *J* = 40.3 Hz), 133.9, 132.9, 131.4, 129.7 (q, *J* = 1.0 Hz), 125.7, 118.9 (q, *J* = 271.6 Hz), 116.9 (q, *J* = 1.5 Hz), 61.4, 14.1; **^{19}F NMR** (376 MHz, $CDCl_3$) δ -56.66; **IR** (ATR, neat): 2914, 1724, 1565, 1385, 1249, 1145, 1067, 972, 752; **HRMS** (ESI+) calcd (m/z) for $C_{13}H_{10}F_3N_3O_4Na$: [M+Na] 352.0516; found 352.0515.

Procymidone–NO_2 (**53**)

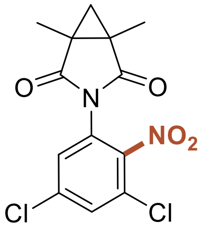

Yield 91%, 150 mg (using the general procedure I, 19 hours); yellow solid; **mp** 199.3-200.0 °C; **^{1}H NMR** (400 MHz, $CDCl_3$) δ 7.58 (d, *J* = 2.1 Hz, 1H), 7.33 (s, 1H), 1.91 (d, *J* = 4.8 Hz, 1H), 1.48 (s, 6H), 1.22 (d, *J* = 4.8 Hz, 1H); **^{13}C NMR** (101 MHz, $CDCl_3$) δ 174.7, 144.5, 137.5, 131.1, 129.0, 128.4, 127.1, 32.6, 30.7, 9.9; **IR** (ATR, neat): 3074, 1780, 1720, 1571, 1442, 1360, 1143, 1143, 1110, 806, 731, 522; **HRMS** (ESI+) calcd (m/z) for $C_{13}H_{10}F_3N_3O_4Na$: [M+H] 329.009; found 329.0089.

Fluorodifen (**54A**) [CAS: 15457-05-3] and 2,4-dinitrophenyl-4-trifluoromethylphenyl ether (**54B**) [CAS: 31716-59-3]

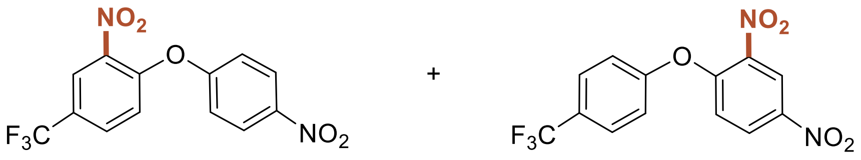

(15 : 1)

Yield of **54A** 82.5%, 135 mg (using the general procedure I, 19 hours); yellow solid; **mp** 94-96 °C; **^{1}H NMR** (300 MHz, $CDCl_3$) δ 8.39 – 8.23 (m, 3H), 7.89 (dd, *J* = 8.8, 2.2 Hz, 1H), 7.30 (d, *J* = 8.7 Hz, 1H), 7.20 – 7.08 (m, 2H); **^{13}C NMR** (126 MHz, $CDCl_3$) δ 160.6, 151.1, 144.5, 141.8, 133.97 – 130.25 (m), 128.1 (q, *J* = 34.8 Hz), 126.4, 124.1 (q, *J* = 3.7 Hz), 123.0, 122.5 (q, *J* = 273.3 Hz), 118.5; **^{19}F NMR** (282 MHz, $CDCl_3$) δ -62.47; **IR** (ATR, neat): 2916, 1600, 1586, 1533, 1347, 1322, 1230, 902, 804, 747, 633; **HRMS** (EI) calcd (m/z) for $C_{13}H_7N_2F_3O_5$: [M^+] 328.03016; found 328.03004.

Yield of **54B** 5.5%, 9.1 mg (using the general procedure I, 19 hours); white solid; **mp** 114-116 °C; **^{1}H NMR** (300 MHz, $CDCl_3$) δ 8.91 (d, *J* = 2.7 Hz, 1H), 8.42 (dd, *J* = 9.2, 2.7 Hz, 1H), 7.78 (d, *J* = 8.5 Hz, 2H), 7.31 – 7.24 (m, 2H), 7.16 (d, *J* = 9.2 Hz, 1H); **^{13}C NMR** (75 MHz, $CDCl_3$) δ 156.8, 154.8, 142.6, 140.5, 129.1, 128.7 (d, *J* = 33.2 Hz), 128.2 (q, *J* = 3.6 Hz), 123.8 (q, *J* = 270.7 Hz), 122.3, 120.3, 119.9; **^{19}F NMR** (282 MHz, $CDCl_3$) δ -62.23; **IR** (ATR, neat): 3095, 1601, 1532, 1508, 1349, 1317, 1270, 1123, 1063, 833, 676, 638; **HRMS** (EI) calcd (m/z) for $C_{13}H_7N_2F_3O_5$: [M^+] 328.03016; found 328.03004.

4-Nitroestrone 3 methyl ether (**55A**) [CAS: 14846-62-9] and 2-nitroestrone 3 methyl ether (**55B**) [CAS:16223-65-7]

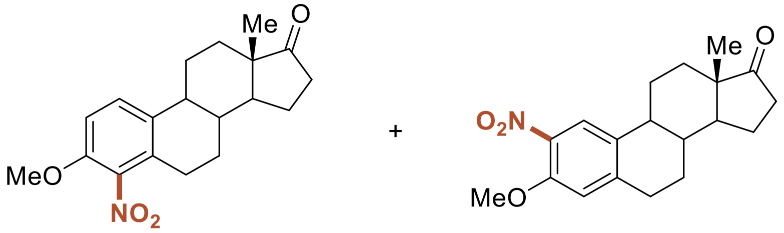

(6 : 1)

Yield of **55A** 78%, 128.5 mg (using the general procedure I); yellow solid; **mp** 150-152 °C; **^{1}H NMR** (500 MHz, $CDCl_3$) δ 7.34 (d, *J* = 8.8 Hz, 1H), 6.85 (d, *J* = 8.8 Hz, 1H), 3.86 (s, 3H), 2.86 – 2.69 (m, 2H), 2.51 (dd, *J* = 19.1, 8.8 Hz, 1H), 2.42 – 2.35 (m, 1H), 2.30 – 2.21 (m, 1H), 2.15 (dt, *J* = 18.6, 8.9 Hz, 1H), 2.09 – 2.00 (m, 2H), 1.97 (dd, *J* = 9.1, 2.6 Hz, 1H), 1.68 – 1.36 (m, 6H), 0.91 (s, 3H); **^{13}C NMR** (126 MHz, $CDCl_3$) δ 148.5, 141.7, 133.2, 128.9, 127.6, 109.9, 56.3, 50.2, 47.8, 43.8, 37.5, 35.8, 31.4, 25.9, 25.4, 23.9, 21.5, 13.8; **IR** (ATR, neat): 2929, 1729, 1619, 1514, 1450, 1267, 1014, 755; **HRMS** (ESI+) calcd (m/z) for $C_{19}H_{23}NO_4$: [M-Na^+] 352.1515; found 352.1519.

Yield of **55B** 13%, 21 mg (using the general procedure I); yellow solid; **mp** 244-248 °C; **^{1}H NMR** (500 MHz, $CDCl_3$) δ 7.84 (s, 1H), 6.80 (s, 1H), 3.94 (s, 3H), 2.97 (p, *J* = 11.4 Hz, 2H), 2.54 (dd, *J* = 19.1, 8.7 Hz, 1H), 2.45 – 2.38 (m, 1H), 2.32 – 2.22 (m, 1H), 2.17 (dt, *J* = 18.7, 8.9 Hz, 1H), 2.12 – 2.05 (m, 2H), 2.01 (d, *J* = 12.3 Hz, 1H), 1.71 – 1.47 (m, 6H), 0.94 (s, 3H); **^{13}C NMR** (126 MHz, $CDCl_3$) δ 151.2, 144.4, 137.4, 132.4, 123.1, 113.6, 56.5, 50.3, 47.9, 43.5, 37.9, 35.8, 31.3, 29.1, 26.1, 25.7, 21.5, 13.8; **IR** (ATR, neat):

2917, 1728, 1526, 1491, 1404, 1377, 1282, 1074, 856, 816, 657; **HRMS** (ESI+) calcd (m/z) for $C_{19}H_{23}NO_4$: [M-Na$^+$] 352.1515; found 352.1519.

Arbutin peracetate-NO_2 (**56**)

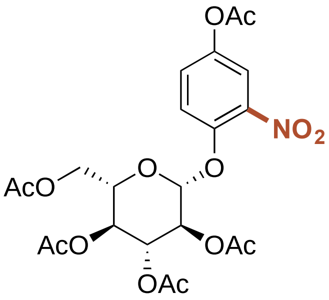

Yield 74%, 195 mg (using the general procedure II); yellow solid, **mp** 149.6-150.0 °C; **^{1}H NMR** (300 MHz, CD_3CN) δ 7.63 (d, *J* = 2.7 Hz, 1H), 7.52 – 7.34 (m, 2H), 5.42 – 5.31 (m, 2H), 5.30 – 5.11 (m, 2H), 4.24 (qd, *J* = 12.4, 3.9 Hz, 2H), 4.15 – 4.02 (m, 1H), 2.29 (d, *J* = 3.3 Hz, 3H), 2.09 – 1.93 (m, 15H); **^{13}C NMR** (75 MHz, CD_3CN) δ 169.9, 169.5, 169.2, 168.9, 168.9, 146.4, 145.2, 140.4, 127.3, 119.2, 118.2, 99.2, 71.8, 71.6, 69.9, 67.6, 61.2, 19.8, 19.6, 19.6, 19.5, 19.5; **IR** (ATR, neat): 1751, 1533, 1366, 1227, 1185, 1038, 926, 597; **HRMS** (ESI+) calcd (m/z) for $C_{22}H_{25}KNO_{14}$: [M+K] 566.0907; found 566.0898.

(R)-6-Methoxy-2,8-dimethyl-7-nitro-2-((4R,8R)-4,8,12-trimethyltridecyl)chromane (**57**)

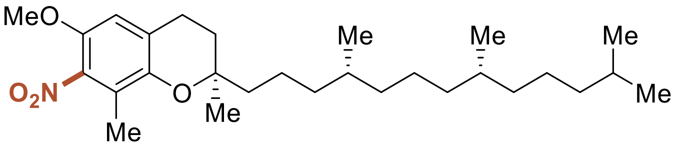

Yield 75%, 173 mg (using the general procedure I); yellow oil; **^{1}H NMR** (300 MHz, $CDCl_3$) δ 6.71 (s, 1H), 3.82 (s, 3H), 2.68 (t, *J* = 6.9 Hz, 2H), 2.19 (s, 3H), 1.77 (h, *J* = 7.0 Hz, 2H), 1.56 – 1.11 (m, 24H), 0.88 – 0.85 (m, 12H); **^{13}C NMR** (75 MHz, $CDCl_3$) δ 145.4, 143.3, 138.9, 129.7, 113.6, 113.2, 75.9, 56.7, 39.5, 39.1, 37.2, 37.1, 37.1, 37.0, 32.5, 32.4, 29.9, 27.7, 24.5, 24.2, 23.6, 22.4, 22.3, 20.6, 19.5, 19.3, 18.1, 16.3; **IR** (ATR, neat): 2924, 1529, 1476, 1375, 1239, 1102, 1016, 911, 809; **HRMS** (ESI+) calcd (m/z) for $C_{28}H_{47}NO_4$: [M$^+$] 461.3500; found 461.3500.

Nordihydrocapsaicin-NO_2 (**58**)

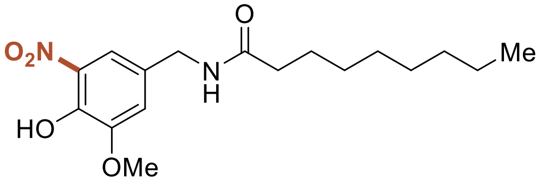

Yield 94%, 159 mg (using the general procedure I); yellow solid; **mp** 121.5-122.5 °C; **^{1}H NMR** (400 MHz, $CDCl_3$) δ 10.72 (s, 1H), 7.57 (d, *J* = 1.9 Hz, 1H), 7.12 (d, *J* = 1.9 Hz, 1H), 5.99 (s, 1H), 4.41 (d, *J* = 6.0 Hz, 2H), 3.94 (s, 3H), 2.26 (t, *J* = 7.6 Hz, 2H), 1.67 (p, *J* = 7.4 Hz, 2H), 1.39 – 1.21 (m, 11H), 0.88 (t, *J* = 6.8 Hz, 3H); **^{13}C NMR** (101 MHz, $CDCl_3$) δ 173.3, 150.2, 145.7, 133.4, 130.2, 117.5, 114.2, 56.7, 42.7, 36.7, 31.8, 29.3, 29.1, 25.7, 22.6, 14.1; **IR** (ATR, neat): 3296, 2920, 2847, 1642, 1532, 1327, 1268, 1220, 1130, 1060, 857, 689; **HRMS** (ESI+) calcd (m/z) for $C_{17}H_{30}N_3O_5$: [M+NH$_4$] 356.2180; found 356.2186.

N-Boc-*p*-nitro-L-phenylalanine (**59A**) [CAS: 33305-77-0] and *N*-Boc-*o*-nitro-L-phenylalanine (**59B**) [CAS: 185146-84-3]

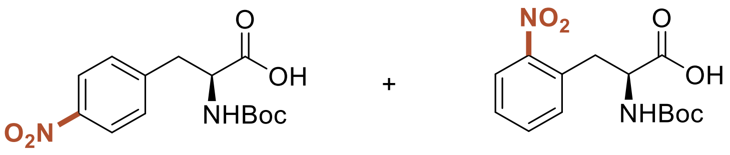

(3.8 : 1)

Yield 51%, 125 mg (using the general procedure I), yellow solid; **mp** 111.9-112.6 °C; **^{1}H NMR** (300 MHz, C_2D_6SO) δ 12.71 (s, 1H), 8.15 (d, *J* = 8.6 Hz, 2H), 7.53 (d, *J* = 8.4 Hz, 2H), 7.20 (d, *J* = 8.6 Hz, 1H), 4.19 (ddd, *J* = 10.5, 8.5, 4.5 Hz, 1H), 3.18 (dd, *J* = 13.7, 4.6 Hz, 1H), 2.96 (dd, *J* = 13.7, 10.5 Hz, 1H), 1.30 (s, 9H); **^{13}C NMR** (75 MHz, C_2D_6SO) δ 173.0, 155.3, 146.4, 146.2, 130.4, 123.1, 78.1, 54.5, 36.2, 28.0.

Yield 13%, 20 mg (using the general procedure I), yellow solid, **mp** 129-131 °C; **^{1}H NMR** (300 MHz, C_2D_6SO) δ 12.36 (s, 1H), 7.91 (d, *J* = 8.1 Hz, 1H), 7.72 (dt, *J* = 13.8, 6.9 Hz, 3H), 7.50 (t, *J* = 7.5 Hz, 1H), 5.33 (q, *J* = 8.3 Hz, 1H), 2.91 – 2.54 (m, 2H), 1.31 (s, 9H); ^{13}C NMR (75 MHz, C_2D_6SO) δ 171.2, 154.7, 147.9, 138.3, 128.2, 128.2, 123.9, 78.2, 46.9, 28.1.

(R)-2,2'-dimethoxy-3-nitro-1,1'-binaphthalene (**60**)

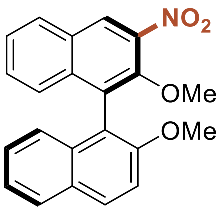

Yield 64%, 115 mg (using the general procedure I); yellow solid; **mp** 189-191 °C; **^{1}H NMR** (300 MHz, $CDCl_3$) δ 8.77 (d, *J* = 2.3 Hz, 1H), 8.10 (d, *J* = 9.1 Hz, 1H), 7.94 (d, *J* = 9.1 Hz, 1H), 7.87 (dd, *J* = 9.4, 2.3 Hz, 1H), 7.82 (d, *J* = 8.2 Hz, 1H), 7.52 (d, *J* = 9.0 Hz, 1H), 7.39 (d, *J* = 9.0 Hz, 1H), 7.27 (ddd, *J* = 8.1, 6.8, 1.3 Hz, 1H), 7.19 – 7.09 (m, 2H), 6.95 (d, *J* = 8.5 Hz, 1H), 3.75 (s, 3H), 3.70 (s, 3H); **^{13}C NMR** (75 MHz, $CDCl_3$) δ 158.2, 154.9, 143.8, 136.9, 133.7, 131.9, 130.12, 129.2, 128.2, 127.2, 126.8, 126.7, 125.2, 124.6, 123.8, 120.1, 119.5, 117.9, 115.5, 113.9, 56.7, 56.6; **IR** (ATR, neat): 3062, 2934, 1616, 1509, 1462, 1334, 1265, 1149, 1060, 828, 743, 595; **HRMS** (ESI+) calcd (m/z) for $C_{22}H_{18}NO_4$: [M^+] 360.1225; found 360.1230.

[2,2]Paracyclophane-NO_2 (**61**) [CAS: 10122-96-0]

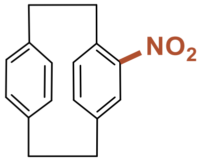

Yield 94%, 119 mg (using the general procedure II); yellow solid; **mp** 155-157 °C; **^{1}H NMR** (500 MHz, $CDCl_3$) δ 7.22 (d, *J* = 1.9 Hz, 1H), 6.79 (dd, *J* = 7.8, 1.9 Hz, 1H), 6.66 – 6.60 (m, 2H), 6.57 (qd, *J* = 7.9, 1.9 Hz, 2H), 6.51 – 6.45 (m, 1H), 4.03 (ddd, *J* = 13.3, 9.5, 2.0 Hz, 1H), 3.19 (tdt, *J* = 12.8, 7.2, 3.1 Hz, 4H), 3.12 – 3.02 (m, 2H), 2.90 (ddd, *J* = 13.3, 10.0, 7.1 Hz, 1H); **^{13}C NMR** (126 MHz, $CDCl_3$) δ 149.3, 142.1, 139.8, 139.3, 137.8, 137.3, 136.5, 133.2, 133.1, 132.4, 129.9, 129.6, 36.0, 35.0, 34.8, 34.5; **IR** (ATR, neat): 2924, 1602, 1516, 1482, 1330, 1180, 1094, 903, 804, 634, 507.

3-HHB(F)-F-NO_2 (**62**)

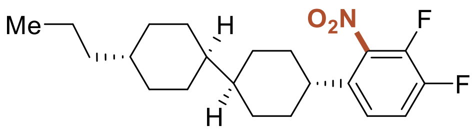

Yield 90%, 164 mg (using the general procedure I); yellow solid; **mp** 67-69 °C; **^{1}H NMR** (300 MHz, $CDCl_3$) δ 7.66 (dd, *J* = 9.6, 7.3 Hz, 1H), 7.24 (dd, *J* = 11.3, 7.6 Hz, 1H), 3.03 (t, *J* = 11.8 Hz, 1H), 1.97 – 1.68 (m, 9H), 1.47 – 0.78 (m, 20H); **^{13}C NMR** (75 MHz, $CDCl_3$) δ 152.8 (dd, *J* = 256.7, 12.4 Hz), 147.5 (dd, *J* = 252.0, 14.1 Hz), 145.42 – 144.20 (m), 140.1 (dd, *J* = 6.2, 4.1 Hz), 116.8 (d, *J* = 19.1 Hz), 114.3 (dd, *J* = 21.3, 1.9 Hz), 43.3, 42.9, 39.8, 38.9, 37.6, 34.1, 33.6, 30.1, 29.99, 20.1, 14.4; **^{19}F NMR** (282 MHz, $CDCl_3$) δ -128.75 – -128.94 (m), -137.26 (ddd, *J* = 21.7, 9.7, 7.7 Hz); **IR** (ATR, neat): 2916, 2847, 1600, 1529, 1508, 1445, 1356, 1298, 1186, 882, 804, 633; **HRMS** (EI) calcd (m/z) for $C_{21}H_{28}NOF_2$: [M^+] 348.21335; found 348.21286.

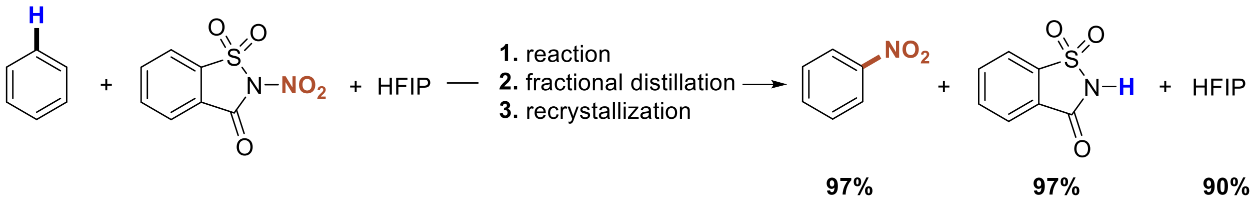

Supplementary Figure 7. Scale up synthesis of nitrobenzene.

An oven-dried, 50 mL two-neck round-bottom flask was charged on the benchtop with a magnetic pTFE-coated stirbar and reagent **4a** (25 g, 109.2 mmol, 1.3 equiv.). The flask was closed with a septum and the atmosphere was cycled tree times with Ar/vac. Benzene (7.5 ml, 83.92 mmol, 1.0 equiv.) in HFIP (20 mL) was added with a plastic syringe and the reaction mixture was heated at 55 °C with vigorous stirring for 3 hours. HFIP (18 mL) and nitrobenzene **5** (10.0 g, 97%) were separated by fractional distillation of the crude reaction mixture. The remaining solid residue was recrystallized from acetonitrile to obtain analytically pure saccharin (19 g, 97%).

a)

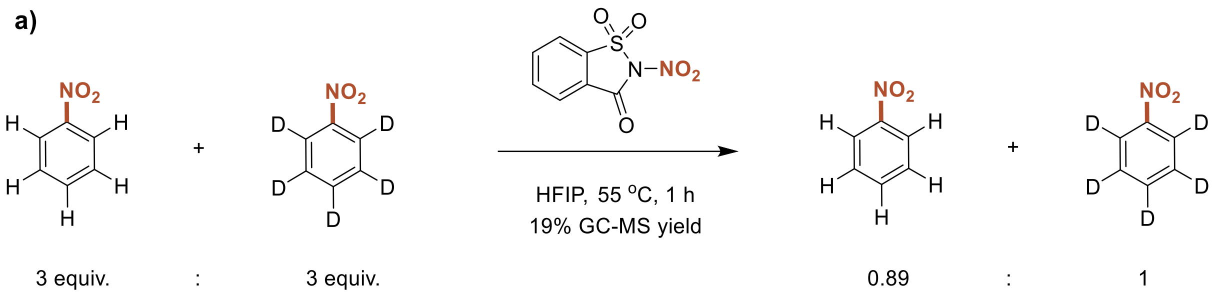

b)

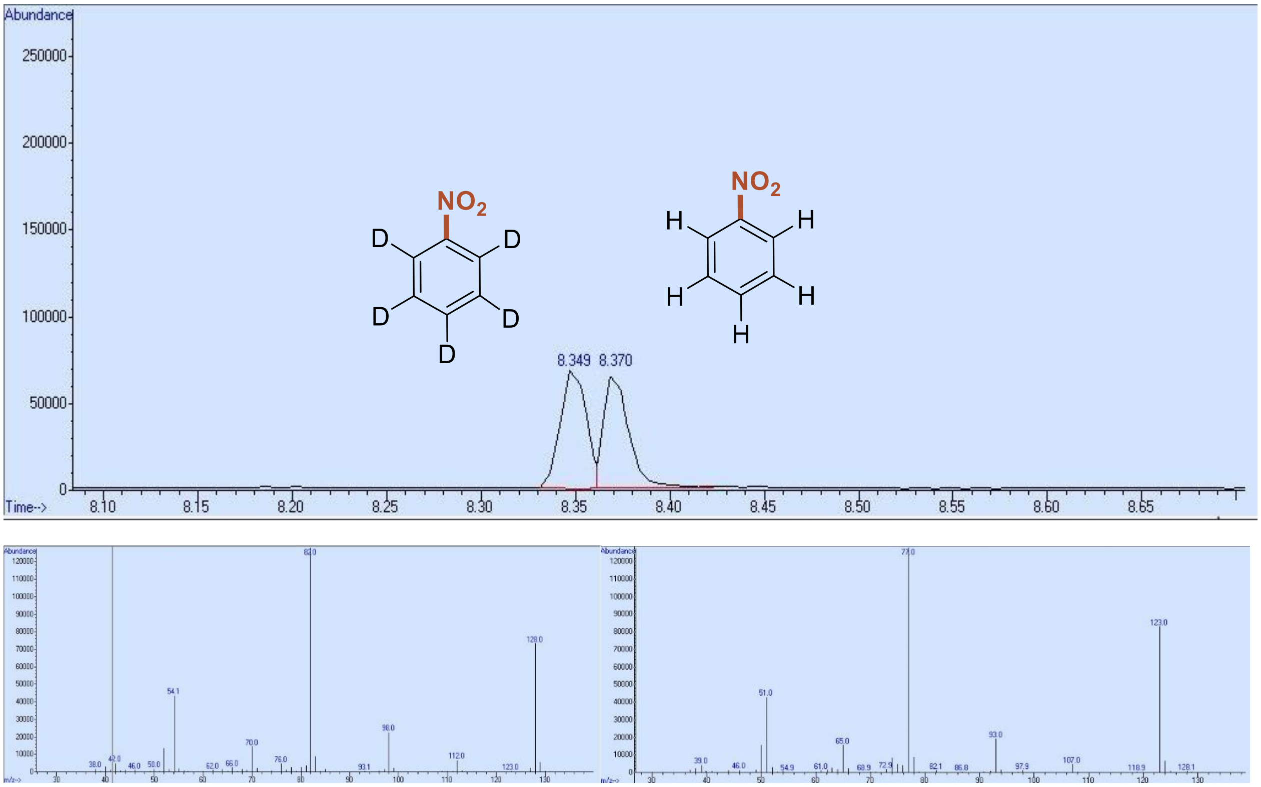

peak #	R.T. min	first scan	max scan	last scan	PK TY	peak height	corr. area	corr. % max.	% of total
1	8.349	1503	1505	1515	M	79570	804250	100.00%	52.767%
2	8.370	1498	1501	1503	M	69570	719901	89.51%	47.233%

Supplementary Figure 8. a) Competitive kinetic isotope effect experiment. b) GC-MS Chromatogram of a kinetic isotope effect of C_6H_6 vs C_6D_6.

An oven-dried 10 mL microvial was charged on the bench with a magnetic stir bar and **4a** (114 mg, 0.5 mmol, 1 equiv.). The vial was sealed and. HFIP (1 mL) was added with a syringe followed by benzene (134 µL, 1.5 mmol, 3 equiv.) and deuterated benzene (133 µL, 1.5 mmol, 3 equiv.) with a microsyringe. The reaction was placed in a heating block at 55 °C and stirred vigorously for 1 h. An internal standard of decane (97 µL, 0.5 mmol, 1.0 equiv.) was added and the reaction mixture was analyzed with GC-MS. As can be seen in the following figure (Supplementary Fig. 8), the observed ratio between $C_6H_5NO_2$ (8.349 min) and $C_6D_5NO_2$ (8.370 min) indicated a 0.89:1 KIE. The KIE measurement was performed in duplicate with independently conducted experiments.

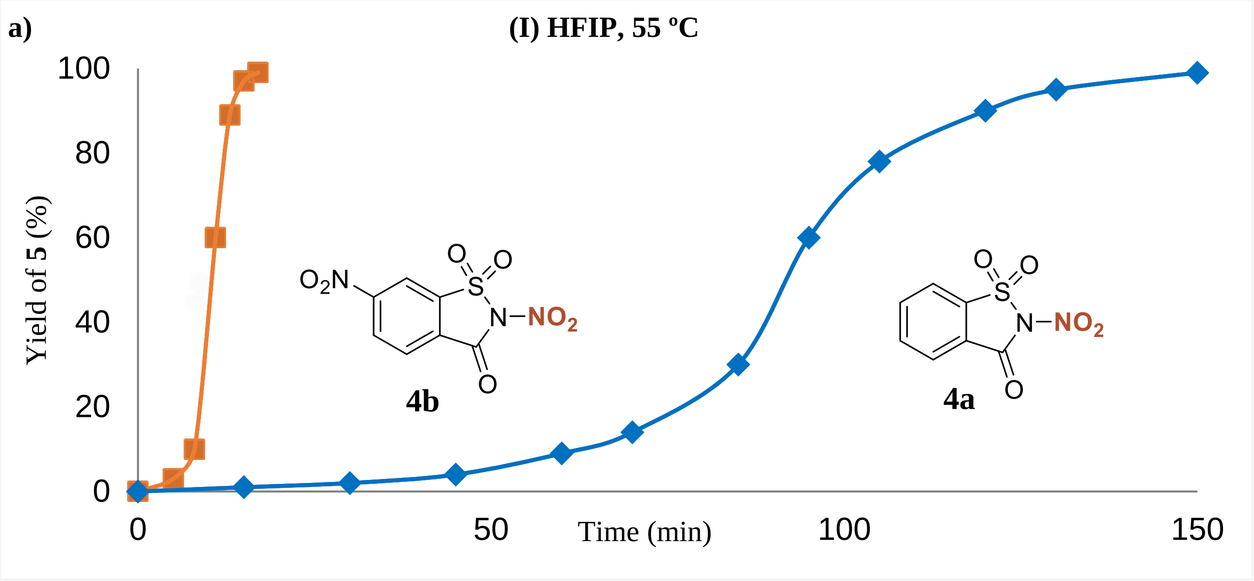

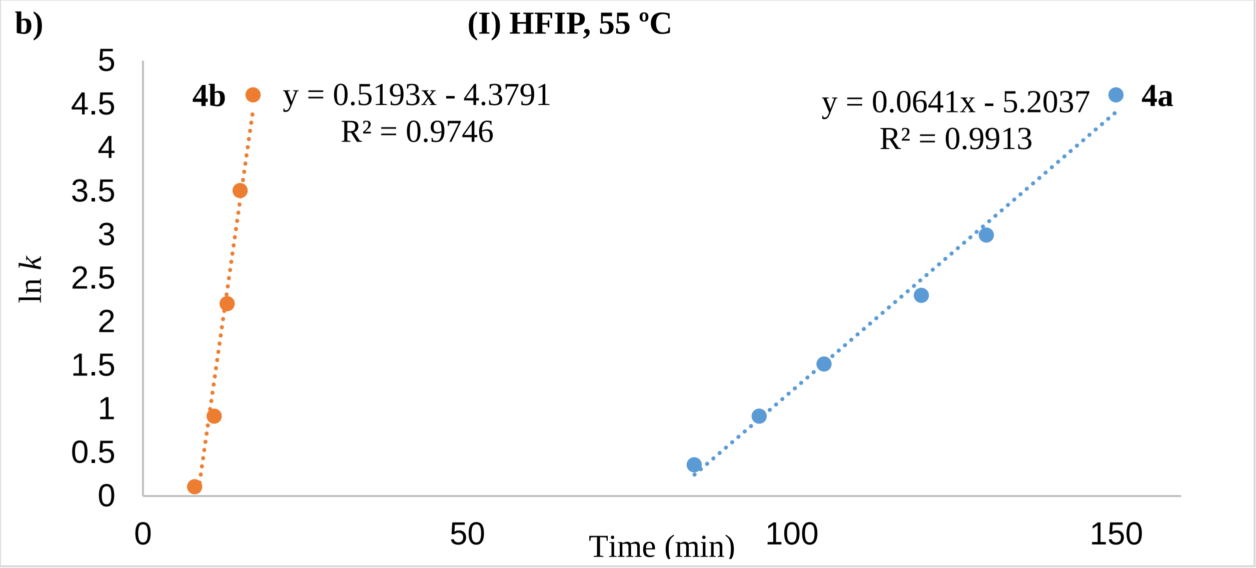

Supplementary Figure 9. a) Reaction profile for the nitration of benzene under reaction conditions I. b) ln *k* vs time.

A 25 mL round bottom flask was charged with reagent **4a** or **4b** (0.65 mmol, 1.3 equiv.) and sealed under nitrogen atmosphere. Benzene (44.69 µL, 0.5 mmol, 1.0 equiv.), *n*-decane (97 µL, 0.5 mmol, 1.0 equiv.) and HFIP (1 mL) were added and the reaction mixture was vigorously stirred at 55 °C. Samples of the reaction mixture (50 µL) were periodically taken and the yield of the nitrobenzene was determined by GC-MS. The experiment was repeated twice, and the average values for the formation of nitrobenzene (%) were plotted vs the reaction time (min). The induction period could be due to the formation of a π complex, however we did not observe such a species by DFT. Under these conditions the NO_2 group of **4a** was found to interact with HFIP *via* a hydrogen bond prior to nitronium transfer. We hypothesize that the induction period is due to the breaking of this weak adduct. Nevertheless, autocatalysis cannot be excluded.

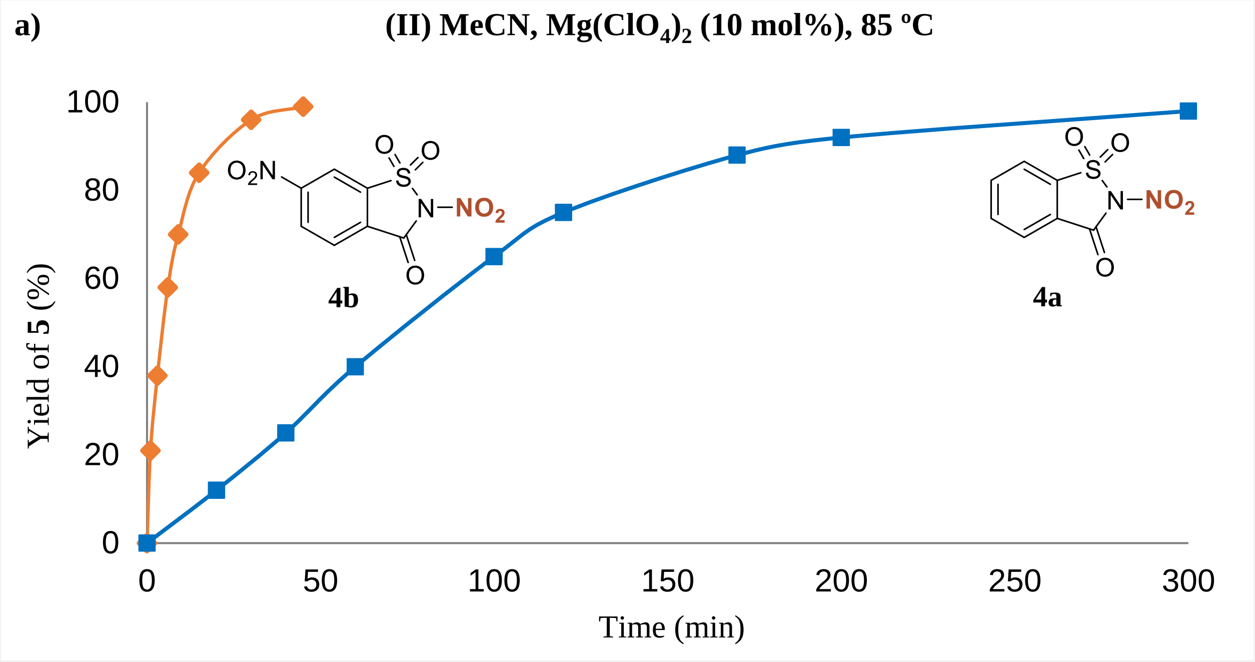

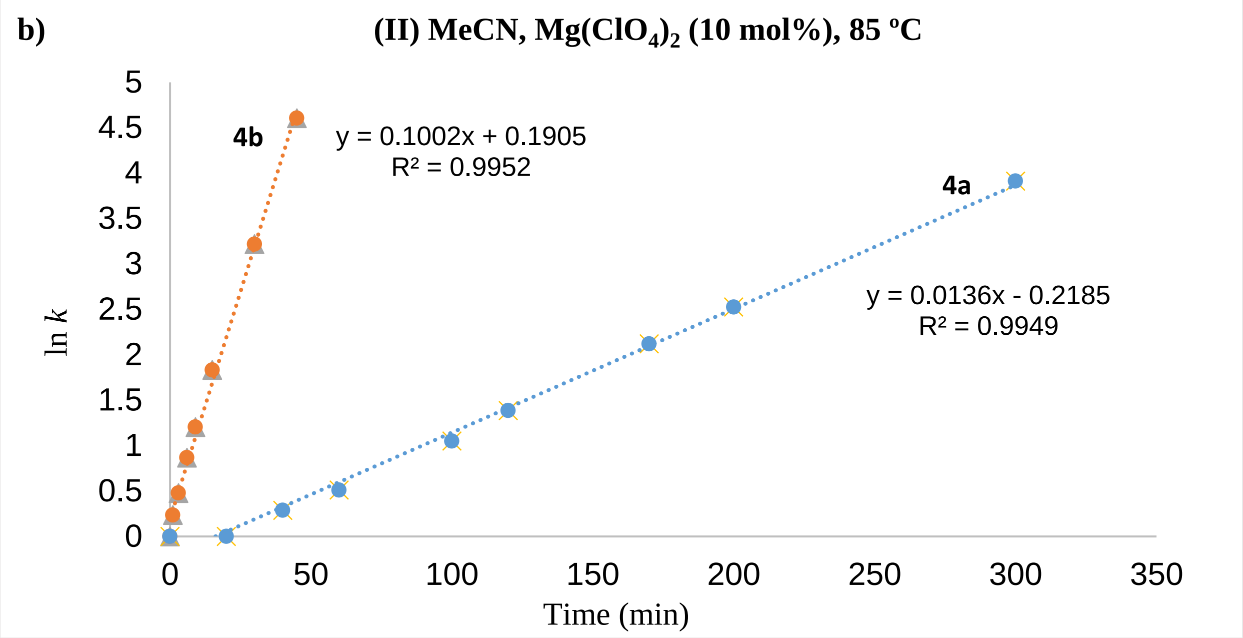

Supplementary Figure 10. a) Reaction profile for the nitration of benzene under reaction conditions II. b) ln *k* vs time.

A 25 mL round bottom flask was charged with reagent **4a** or **4b** (0.65 mmol, 1.3 equiv.), $Mg(ClO_4)_2$ (11.2 mg, 0.05 mmol, 10 mol%) and sealed under nitrogen atmosphere. Benzene (44.69 μL, 0.5 mmol, 1.0 equiv.), *n*-decane (97 μL, 0.5 mmol, 1.0 equiv.) and MeCN (1 mL) were added and the reaction mixture was vigorously stirred at 55 °C. Samples of the reaction mixture (50 μL) were periodically taken and the yield of the nitrobenzene was determined by GC-MS. The experiment was repeated twice, and the average values for the formation of nitrobenzene (%) were plotted vs the reaction time (min).

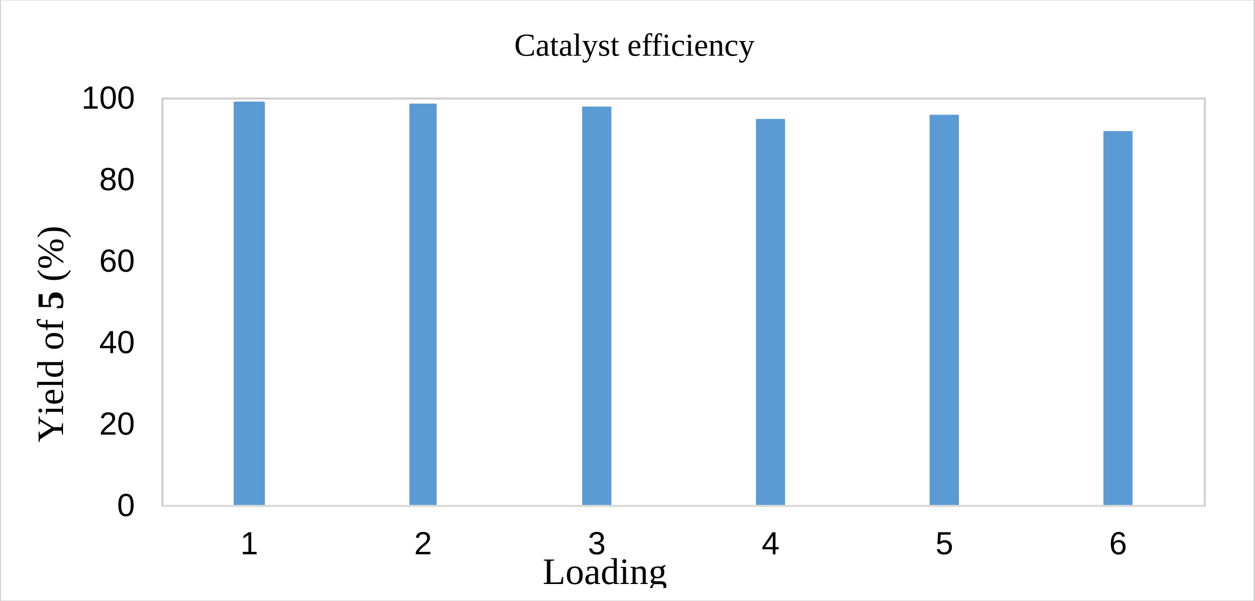

Supplementary Figure 11. Catalyst ($Mg(ClO_4)_2$) efficiency for the nitration of benzene under reaction conditions II. (*Run 1 (99%), Run 2 (99%), Run 3 (98%), Run 4 (95%), Run 5 (96%) and Run 6 (92%)*).

Catalyst ($Mg(ClO_4)_2$) Efficiency for the Nitration of Benzene under Reaction Conditions II: A 100 mL Schlenk flask was charged with reagent **4a** (148 mg, 0.65 mmol, 1.3 equiv.), $Mg(ClO_4)_2$ (11.2 mg, 0.05 mmol, 10 mol%) and sealed under nitrogen atmosphere. Benzene (44.69 µL, 0.5 mmol, 1.0 equiv.), *n*-decane (97 µL, 0.5 mmol, 1.0 equiv.) and MeCN (1 mL) were added and the reaction mixture was vigorously stirred at 55 °C for 5 hours. Samples of the reaction mixture (50 µL) were taken and the yield of nitrobenzene determined by GC-MS. When almost all strating material had been consumed, the same Schlenk flask was subsequently charged with reagent **4a** (148 mg, 0.65 mmol, 1.3 equiv.), benzene (44.69 µL, 0.5 mmol, 1.0 equiv.), *n*-decane (97 µL, 0.5 mmol, 1.0 equiv.) and MeCN (0.5 mL), and the procedure was repeated 6 times. Supplementary Fig. **11** shows the yield for the formation of nitrobenzene (%). These results show that the magnesium catalyst can be reused without loss of its efficiency. The slight loss of yield after 6 runs may be the result of a high concentration of saccharin as well as nitrobenzene in the reaction mixture accumulated after 6 consecutive loads.

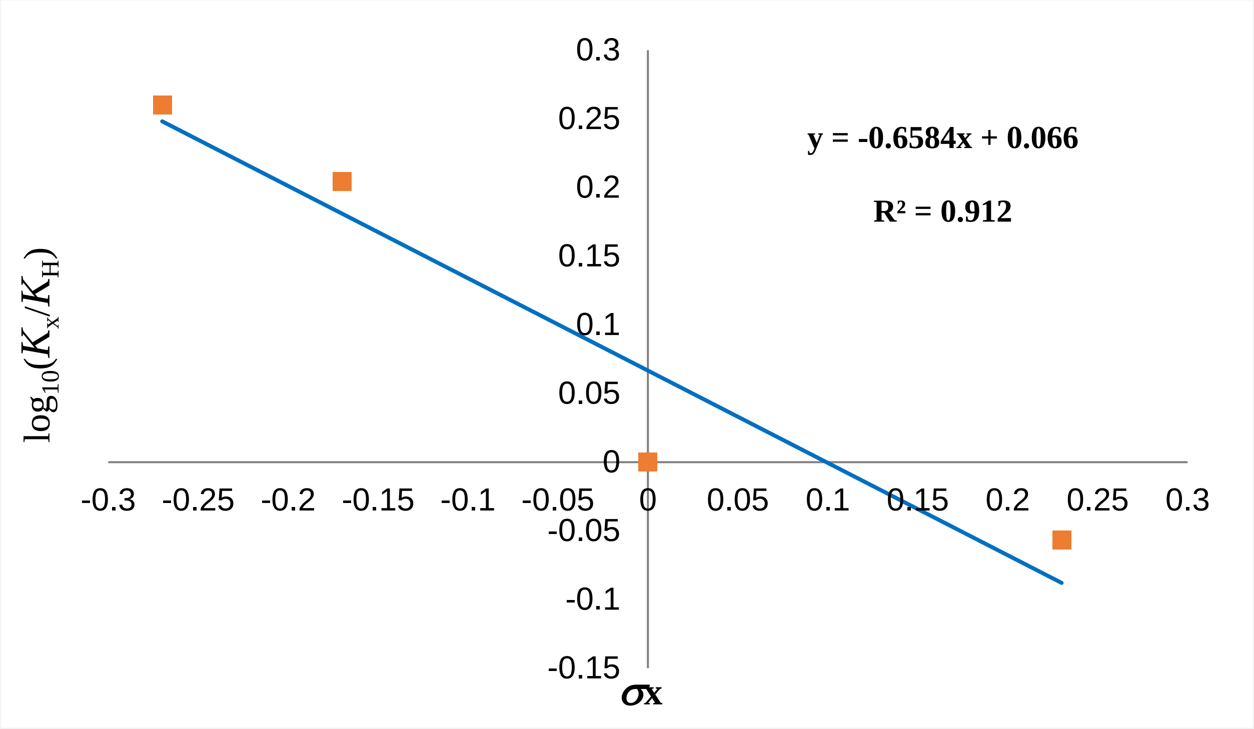

Data analysis for substituted arenes-1

Substituent	4-Br	H	4-CH_3	4-OCH_3
σ	0.23	0	-0.17	-0.27
**Integrals**				
Initial	2.5119	1.2685	2.3212	1.809
Final	2.069	1.114	1.622	1.204
S/S_0	0.8236793	0.8782026	0.6987765	0.6655611
$\ln(S/S_0)$	-0.193974	-0.129878	-0.3584243	-0.4071249
$\ln(S/S_0)/\ln(S_H/S_{H0})$	0.8692792	1	1.60625	1.8244975
$\log_{10}(k_{rel})$	-0.0608407	0	0.2058132	0.2611433

Data analysis for substituted arenes-2

Substituent	4-Br	H	4-CH_3	4-OCH_3
σ	0.23	0	-0.17	-0.27
**Integrals**				
Initial	2.5119	1.2685	2.3212	1.809
Final	2.051	1.1	1.619	1.195
S/S_0	0.8165134	0.8671659	0.6974841	0.660586
$\ln(S/S_0)$	-0.202712	-0.1425249	-0.3602756	-0.414628
$\ln(S/S_0)/\ln(S_H/S_{H0})$	0.9084375	1	1.6145464	1.8581224
$\log_{10}(k_{rel})$	-0.041705	0	0.2080505	0.2690743

Data analysis for substituted arenes-3

Substituent	4-Br	H	4-CH_3	4-OCH_3
σ	0.23	0	-0.17	-0.27
**Integrals**				
Initial	2.5119	1.2685	2.3212	1.809
Final	2.075	1.109	1.629	1.217
S/S_0	0.8260679	0.8742609	0.7017922	0.6727474
$\ln(S/S_0)$	-0.1910783	-0.1343764	-0.354118	-0.3963854
$\ln(S/S_0)/\ln(S_H/S_{H0})$	0.8563021	1	1.5869514	1.7763695
$\log_{10}(k_{rel})$	-0.067373	0	0.2005636	0.2495333

Data analysis for substituted arenes/average

Substituent	4-Br	H	4-CH_3	4-OCH_3
σ	0.23	0	-0.17	-0.27
**Integrals**				
Initial	2.5119	1.2685	2.3212	1.809
Final	2.065	1.107	1.624	1.205
S/S_0	0.8220869	0.8726843	0.6996381	0.6661139
$\ln(S/S_0)$	-0.1959092	-0.1361814	-0.3571921	-0.4062946
$\ln(S/S_0)/\ln(S_H/S_{H0})$	0.8779515	1	1.6007276	1.820777
$\log_{10}(k_{rel})$	-0.0565295	0	0.2043174	0.2602568

Supplementary Figure 12. **Competition experiment**: Relative rate constants determined from the relative integrations of GC-MS diagrams for the competitive nitration reactions between various substituted arenes and reagent **4a** under general reaction conditions I. Values of log10(K_X/K_H) vs σx for the nitration reactions.

Competition experiment: The effect of different substituents on the stabilization of the putative electrophilic σ-complex was evaluated through a series of competition experiments with substituted arenes

(see Supplementary Fig. 12). The experiments were performed following the method described by Harper and coworkers (*42*-*43*). A 25 mL round bottom flask was charged with reagent **4a** (23 mg, 0.1 mmol) and sealed under nitrogen atmosphere. Subsequently a solution consisting of benzene (47 μL, 0.525 mmol,), bromo-benzene (53 μL, 0.503 mmol), toluene (53 μL, 0.500 mmol) and anisole (54 μL, 0.502 mmol) in HFIP (4 mL) was introduced via syringe. The final reaction mixture was vigorously stirred at 55 °C for 3 hours. After completion of the nitration reaction, decane (49 μL, 0.25 mmol) was added to the vessel through a micro-syringe and the final solution was stirred for an additional 5 minutes. The amounts of the corresponding arenes were determined by GC-MS analysis with respect to decane. The experiment was repeated three times with the use of the same amounts of arenes.

Supplementary Table 4. Use of radical inhibitors under reaction conditions I.

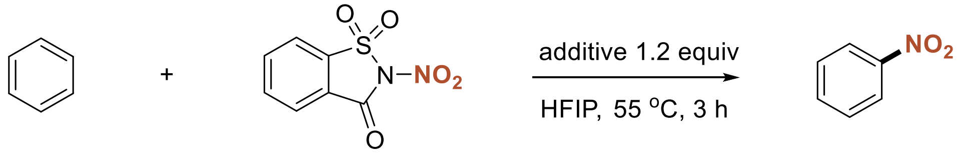

1 equiv. 1.3 equiv.

Radical inhibitors (1. 2 equiv.)	Yield of nitrobenzene
1,4-Dinitribenzene (DNB)	90%
Allyl ether (DAE)	98%
Benzoquinone (BQ)	86%
Styrene	77%

Radical trapping mechanistic experiment: A 25 mL round bottom flask was charged with reagent **4a** (148 mg, 0.65 mmol, 1.3 equiv.) and sealed under nitrogen atmosphere. Benzene (44.69 μL, 0.5 mmol, 1.0 equiv.), additive (0.6 mmol, 1.2 equiv.,) and HFIP (1 mL) were added via syringe and the reaction mixture was vigorously stirred at 55 °C. After 3 h the crude reaction mixture was directly analyzed with GC-MS using *n*-decane (97 μL, 0.5 mmol, 1.0 equiv.) as analytical standard. Results are summarized in Supplementary Table 4**.** It has been experimentally demonstrated that TEMPO and BHT are not suitable radical inhibitors under these reaction conditions, since they participate in a background reaction with nitrating reagent **4a** in HFIP.

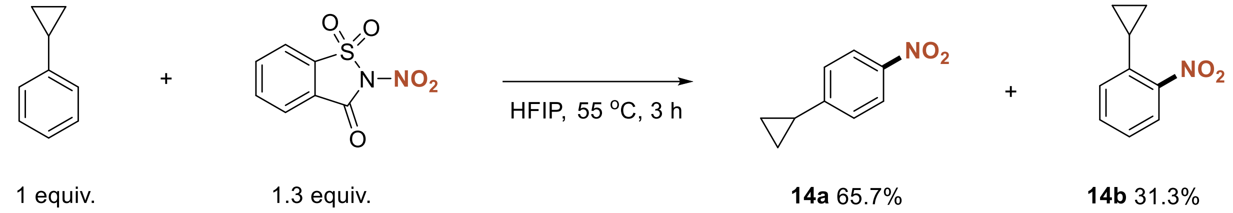

Supplementary Figure 13. Radical clock experiment.

A 25 mL round bottom flask was charged with reagent **4a** (148 mg, 0.65 mmol, 1.3 equiv.) and sealed under nitrogen atmosphere. Cyclopropylbenzene (59 mg, 0.5 mmol, 1.0 equiv.) in HFIP (1 mL) was added via plastic syringe, and the reaction mixture was vigorously stirred at 55 °C for 3 h. The solvent was evaporated and the crude product was purified by flash column chromatography on silica gel (hexane/ethyl acetate 30:1) to afford **14a** and **14b** in 65.7% and 31.3% yield, respectively.

Supplementary Table 5. Crystal data and structure refinement details for compound **4a**.

Empirical formula	$C_7H_3N_3O_7S$
Formula weight	273.18
Temperature/K	100.0
Crystal system	orthorhombic
Space group IT number	61
Space group name	P b c a
a/Å	9.4437(3)
b/Å	13.7774(5)
c/Å	14.6729(6)
α/°	90.0
β/°	90.0
γ/°	90.0
Volume/Å^3	1909.09(12)
Z	8
ρ_{calc}g/cm^3	1.901
μ/mm^{-1}	0.377
F(000)	1104.0
Crystal size/mm^3	0.16 × 0.3 × 0.41
Radiation	MoKα (λ = 0.71073)
2Θ range for data collection/°	2.174 to 29.565
Index ranges	-9 ≤ h ≤ 9, -11 ≤ k ≤ 11, -26 ≤ l ≤ 26
Reflections collected	22172
Independent reflections	2778
Data/restraints/parameters	2778/0/163
Goodness-of-fit on F^2	1.174
Final R indexes [I>=2σ (I)]	R_1 = 0.0586, wR_2 = 0.1093
Final R indexes [all data]	R_1 = 0.0436, wR_2 = 0.1320
Largest diff. peak/hole / e Å^{-3}	0.597/-0.576

Colorless crystals of **4a** were obtained by slow evaporation from a saturated solution of chloroform / acetonitrile 1 : 1.

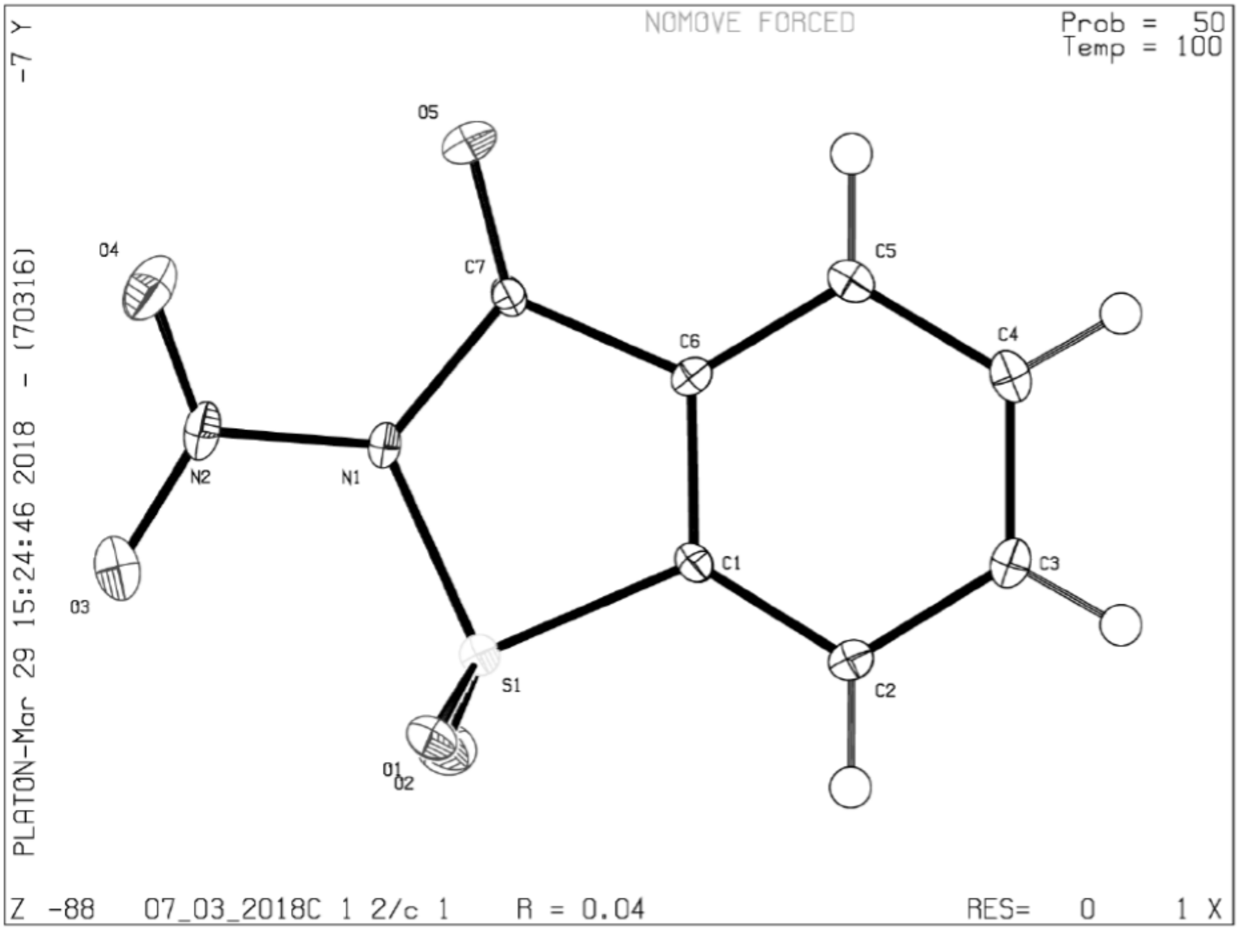

B Level alerts: Because of crystal packing effects intermolecular distances between O2 and O4 (N2) are relatively short 2.83 and 2.84 Å, respectively. However no residual electron density is detected between these two pairs of atoms.

Supplementary Table 6. Crystal data and structure refinement details for compound **4b.**

Empirical formula	$C_7H_3N_3O_7S$
Formula weight	273.18
Temperature/K	100.0
Crystal system	orthorhombic
Space group IT number	61
Space group name	P b c a
a/Å	9.4437(3)
b/Å	13.7774(5)
c/Å	14.6729(6)
α/°	90.0
β/°	90.0
γ/°	90.0
Volume/Å^3	1909.09(12)
Z	8
ρ_{calc}g/cm^3	1.901
μ/mm^{-1}	0.377
F(000)	1104.0
Crystal size/mm^3	0.16 × 0.3 × 0.41
Radiation	MoKα (λ = 0.71073)
2Θ range for data collection/°	2.174 to 29.565
Index ranges	-9 ≤ h ≤ 9, -11 ≤ k ≤ 11, -26 ≤ l ≤ 26
Reflections collected	22172
Independent reflections	2778
Data/restraints/parameters	2778/0/163
Goodness-of-fit on F^2	1.174
Final R indexes [I>=2σ (I)]	R_1 = 0.0586, wR_2 = 0.1093
Final R indexes [all data]	R_1 = 0.0436, wR_2 = 0.1320
Largest diff. peak/hole / e Å^{-3}	0.597/-0.576

Colorless crystals of **4b** were obtained by slow evaporation from a saturated solution of chloroform /acetonitrile 1 : 1.

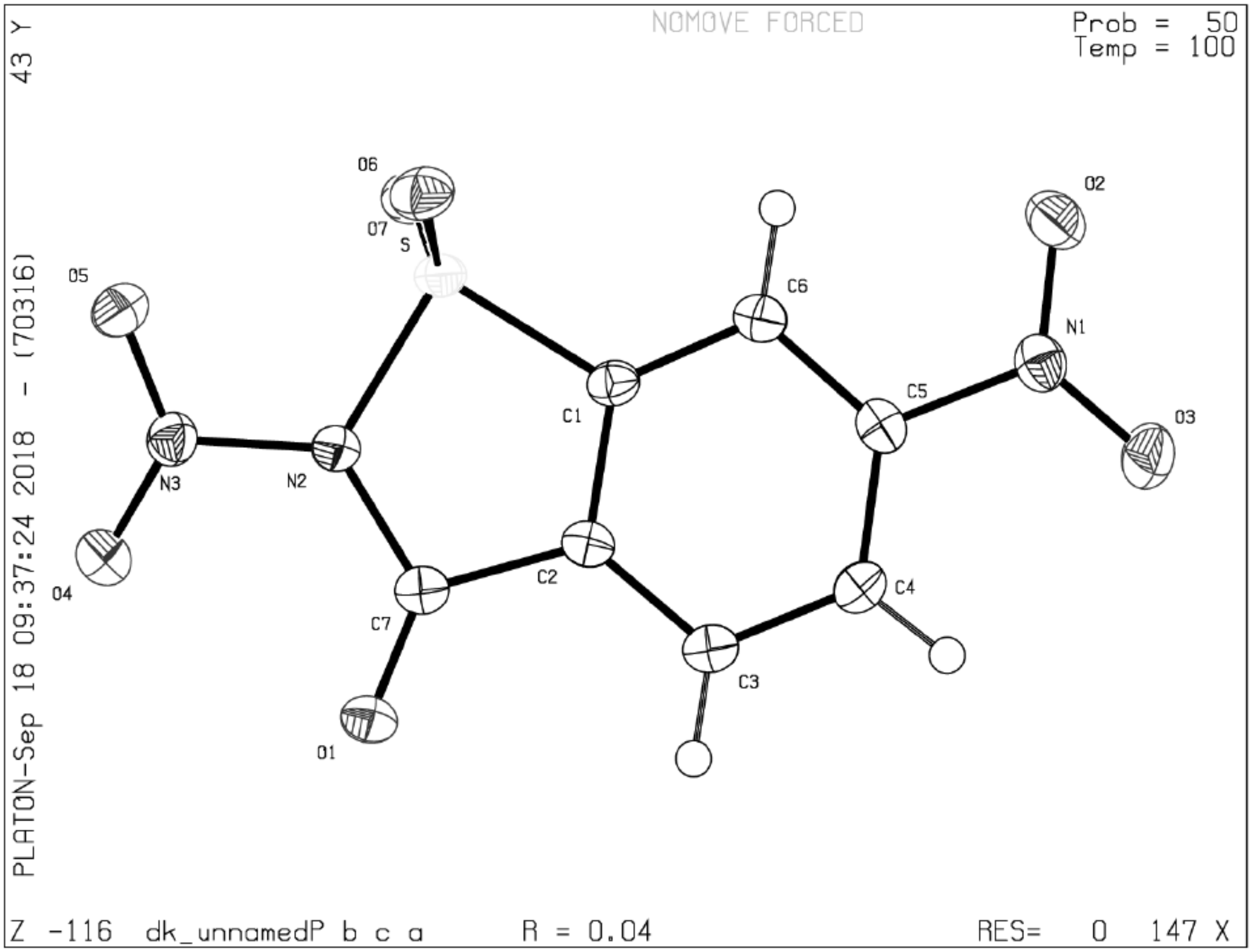

B Level alerts: Because of crystal packing effects an intermolecular distance between O7 and N3 is relatively short 2.79 Å. However, no residual electron density is detected between this pair of atoms.

Supplementary Table 7. Crystal data and structure refinement details for compound **61.**

Empirical formula	$C_{16}H_{15}NO_2$
Formula weight	253.29
Temperature/K	100.0
Crystal system	monoclinic
Space group IT number	9
Space group name	C 1 c 1
a/Å	14.308(3)
b/Å	7.3956(16)
c/Å	11.575(2)
α/°	90.0
β/°	91.230(3)
γ/°	90.0
Volume/Å^3	1224.6(4)
Z	4
ρ_{calc}g/cm^3	1.374
μ/mm^{-1}	0.091
F(000)	536.0
Crystal size/mm^3	0.18 × 0.28 × 0.42
Radiation	MoKα (λ = 0.71073)
2Θ range for data collection/°	2.848 to 26.989
Index ranges	-9 ≤ h ≤ 9, -14 ≤ k ≤ 14, -17 ≤ l ≤ 18
Reflections collected	6274
Independent reflections	2612
Data/restraints/parameters	173/0/2612
Goodness-of-fit on F^2	1.056
Final R indexes [I>=2σ (I)]	R_1 = 0.0659, wR_2 = 0.1308
Final R indexes [all data]	R_1 = 0.0504, wR_2 = 0.1450
Largest diff. peak/hole / e Å^{-3}	0.418/-0.231

Colorless crystals of [2,2]paracyclophane-NO_2 (**61**) were obtained by slow evaporation from a saturated solution of ethyl acetate / hexane 1 : 1.

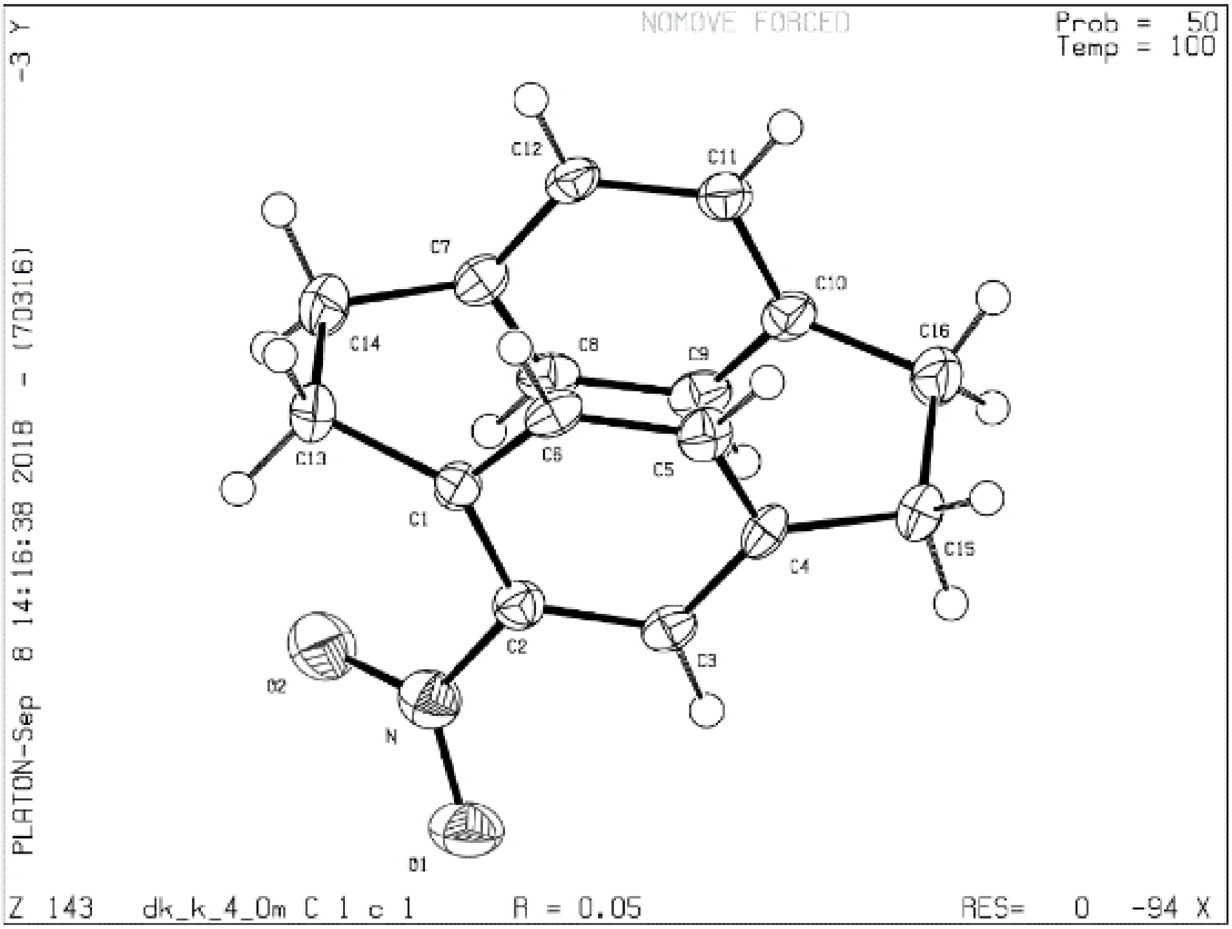

Supplementary Table 8. Crystal data and structure refinement details for compound **54**.

Empirical formula	$C_{13}H_7N_2O_5F_3$
Formula weight	328.21
Temperature/K	100.0
Crystal system	monoclinic
Space group IT number	15
Space group name	P 1 21/c 1
a/Å	21.4056(18)
b/Å	12.0113(10)
c/Å	10.2695(9)
α/°	90.0
β/°	91.143(2)
γ/°	90.0
Volume/Å^3	2639.9(4)
Z	8
ρ_{calc}g/cm^3	1.652
μ/mm^{-1}	0.155
F(000)	1328.0
Crystal size/mm^3	0.1 × 0.28 × 0.49
Radiation	MoKα (λ = 0.71073)
2Θ range for data collection/°	0.951 to 27.995
Index ranges	$-13 \leq h \leq 13$, $-15 \leq k \leq 15$, $-28 \leq l \leq 28$
Reflections collected	38576
Independent reflections	6295
Data/restraints/parameters	415/0/6295
Goodness-of-fit on F^2	1.101
Final R indexes [I>=2σ (I)]	R_1 = 0.0962, wR_2 = 0.1182
Final R indexes [all data]	R_1 = 0.0491, wR_2 = 0.1630
Largest diff. peak/hole / e Å^{-3}	0.258/-0.354

Colorless crystals of Fluorodifen (**54**) were obtained by slow evaporation from a saturated solution in ethyl acetate / hexane 1 : 1.

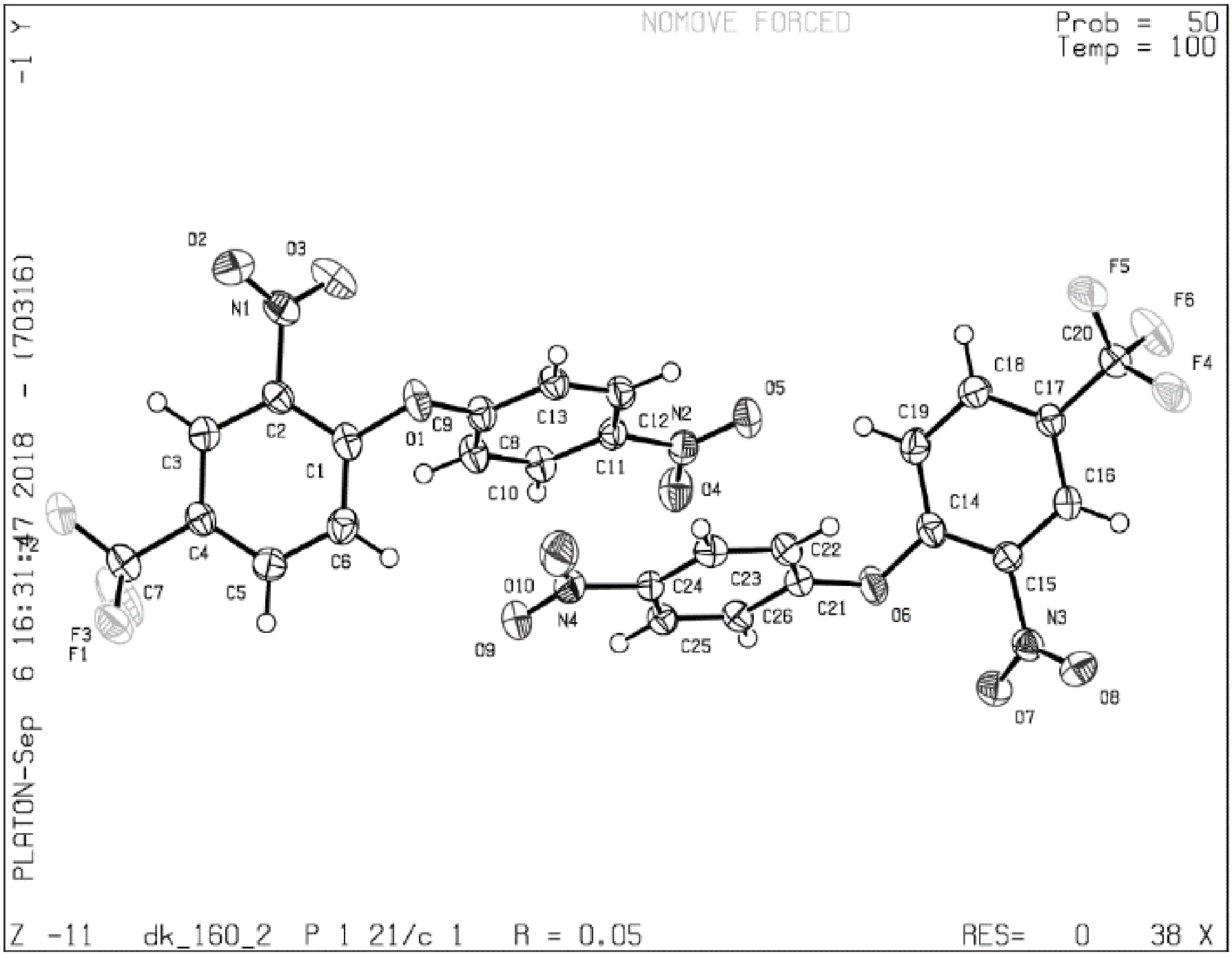

Computational Details: All DFT calculations were performed with the Gaussian09 program package (Revision D.01) (*50*) The structures of all minima and transition states were fully optimized using the hybrid density functional M06–2X (*51*) with the ultrafine pruned (99,590) grid. The 6-311G^{++}(2df,2pd) Pople basis set (*52-53*) was used for all the atoms. The implicit solvation model IEF-PCM was used as implemented in Gaussian (*54-55*), applying all the parameters of 2-propanol, apart from the dielectric constant (ε), which was modified to 16.70 (HFIP) (*56*) according to the literature for the description of HFIP as solvent (*57-58*). Stationary points were characterized by vibrational analysis (only real frequencies for minima, one imaginary frequency for transition states (TSs)), and intrinsic reaction coordinate (IRC) calculations were carried out on the TSs in order to confirm their correct identification. Computed harmonic frequencies were used to calculate the thermal contribution to Gibbs free energy at 298 K and 1 atm. The thermochemistry analysis was performed as implemented in Gaussian09 software. TSs were modelled according to the literature by substitution of H_2SO_4 (*36*) with the nitrating agent (**1-4**) and by introduction of an explicit HFIP molecule (*37*).

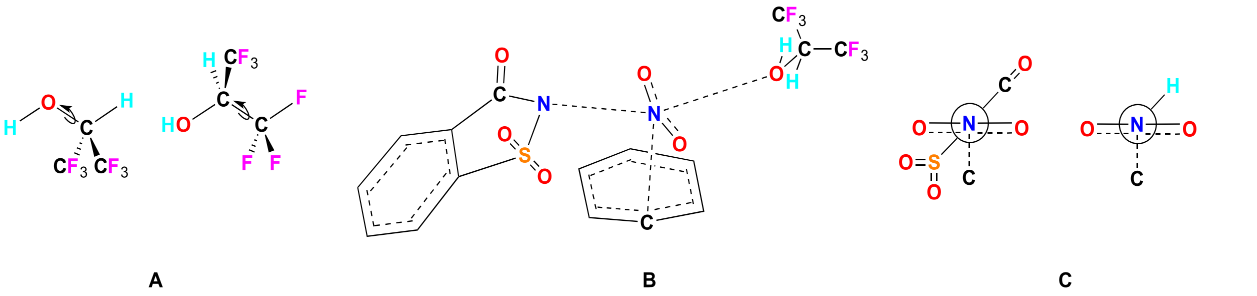

Supplementary Figure 14. **A**) Rotation around torsion angles H–O–C–H and O–C–C–F. **B**) Schematic representation of the TS for **4a**. **C**) Newmann projections along the N···N bond (torsion angle O–N···N–C(=O)) and along the N···O bond (torsion angle O–N···O–H).

The nitrating agents (**1-4**) are characterized by a rigid skeleton without conformational degrees of freedom. On the other side, HFIP is conformationally flexible and several conformers are possible, according to the rotation around the torsion angles H–O–C–H and O–C–C–F (Supplementary Fig. 14A). However, preliminary calculations showed that the two hydrogen atoms prefer the *anti*-conformation (H–O–C–H ≈ 180°), whereas the CF_3 groups assume a staggered conformation (O–C–C–F ≈ 60, 180, 300°). For these reasons, only the relative position of the **1-4** and HFIP with respect to NO_2 were considered (Supplementary Fig. 14B). The relative position is described by the torsion angle O–N···N–C(=O) (Supplementary Fig. 14C) for **1-4** and by the torsion angle O–N···O–H (Supplementary Fig. 14C). According to the two torsion angles (Supplementary Fig. 14C), TSs were calculated for **1-4** (see Supplementary Tables 9-13).

Supplementary Table 9. Gibbs free energy ($\Delta G^{\circ\ddagger}$, in kcal mol^{-1}) for transition states (TS) with **1** as the nitrating agent.

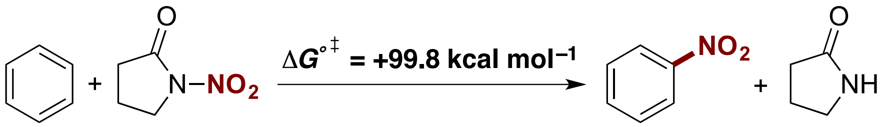

**TS_1**	$\Delta G^{\circ\ddagger}$
TS(1)_1	98.8
TS(2)_1	99.9
TS(3)_1	100.0

Supplementary Table 10. Gibbs free energy ($\Delta G^{\circ\ddagger}$, in kcal mol^{-1}) for transition states (TS) with **2** as the nitrating agent.

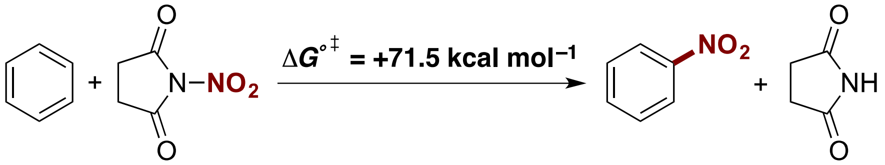

**TS_2**	$\Delta G^{\circ\ddagger}$
TS(1)_2	71.5
TS(2)_2	71.6
TS(3)_2	72.4

Supplementary Table 11. Gibbs free energy ($\Delta G^{\circ\ddagger}$, in kcal mol^{-1}) for transition states (TS) with **3** as the nitrating agent.

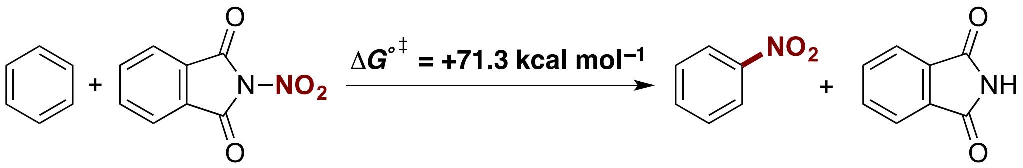

**TS_3**	$\Delta G^{\circ\ddagger}$
TS(1)_3	71.3
TS(2)_3	71.7
TS(3)_3	72.4

Supplementary Table 12. Gibbs free energy ($\Delta G^{\circ\ddagger}$, in kcal mol^{-1}) for transition states (TS) with **4a** as the nitrating agent.

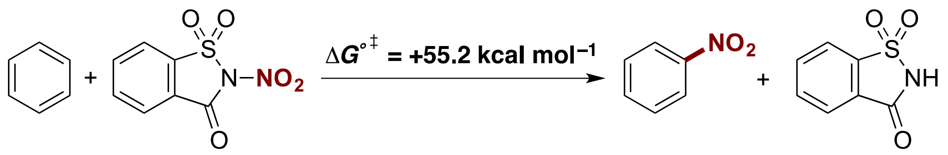

TS_4a	$\Delta G^{\circ\ddagger}$
TS(1)_4a	55.2
TS(2)_4a	55.6
TS(3)_4a	55.9
TS(4)_4a	55.9
TS(5)_4a	56.0
TS(6)_4a	56.2
TS(7)_4a	56.2
TS(8)_4a	56.2
TS(9)_4a	56.7
TS(10)_4a	56.8
TS(11)_4a	56.8

Supplementary Table 13. Gibbs free energy ($\Delta G^{\circ\ddagger}$, in kcal mol^{-1}) for transition states (TS) with **4b** as the nitrating agent.

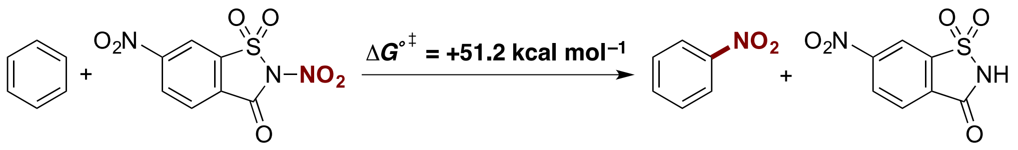

TS_4b	$\Delta G^{\circ\ddagger}$
TS(1)_4b	51.2
TS(2)_4b	51.3
TS(3)_4b	51.5
TS(4)_4b	51.5
TS(5)_4b	51.7
TS(6)_4b	51.8
TS(7)_4b	51.8
TS(8)_4b	51.8
TS(9)_4b	52.4
TS(10)_4b	52.4

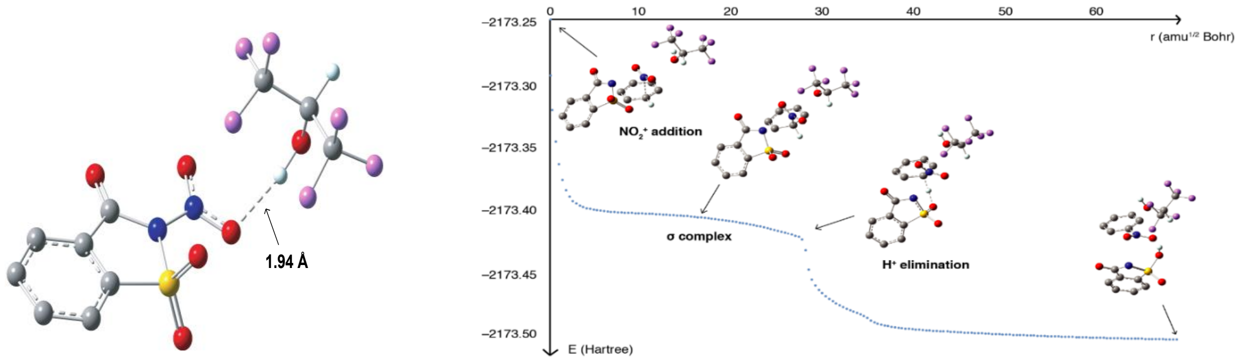

Supplementary Figure 15. Intrinsic reaction coordinate (IRC) of NO_2^+ addition and H^+ elimination by **4a**.

Intrinsic reaction coordinate (IRC): Using density functional theory (DFT) calculations, we did not observe formation of a π complex, but the NO_2 group of **4a** was found to interact with HFIP *via* a hydrogen bond prior to nitronium transfer with a distance of 1.94 Å (Saccharin−NOO····H−OCH(CF_3)$_2$) (Supplementary Figure 15, left structure). The reaction proceeds with a concerted and strongly asynchronous mechanism whereby the NO_2^+ addition is the rate determining step, in agreeance with the first order reaction in benzene and the observed KIE. In this case, the σ complex was not located as a discreet intermediate, although it is observed in the reaction profile and is rapidly followed by H^+ elimination (Fig. 4E). The existence of the σ complex as an intermediate is strongly dependent on the applied hybrid functional, as already observed by Schaefer III (*35*-*36*, *42*,*59*-*60*). Whereas such electrophilic addition mechanisms are generally described for polar solvents which facilitate H^+ elimination, this role is undertaken by saccharin in our transformation. The deprotonation is assisted by its sulfoxide group, generating the sulfenic acid derivative, followed by prototropic equilibrium to generate saccharin (Supplementary Figure 15).

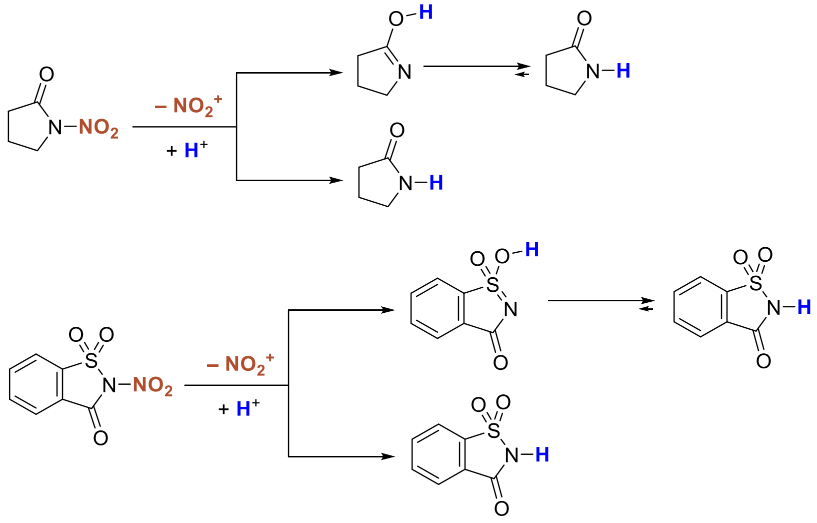

Supplementary Figure 16. H^+ Transfer from benzene to **1** and **4a** at O or N atom.

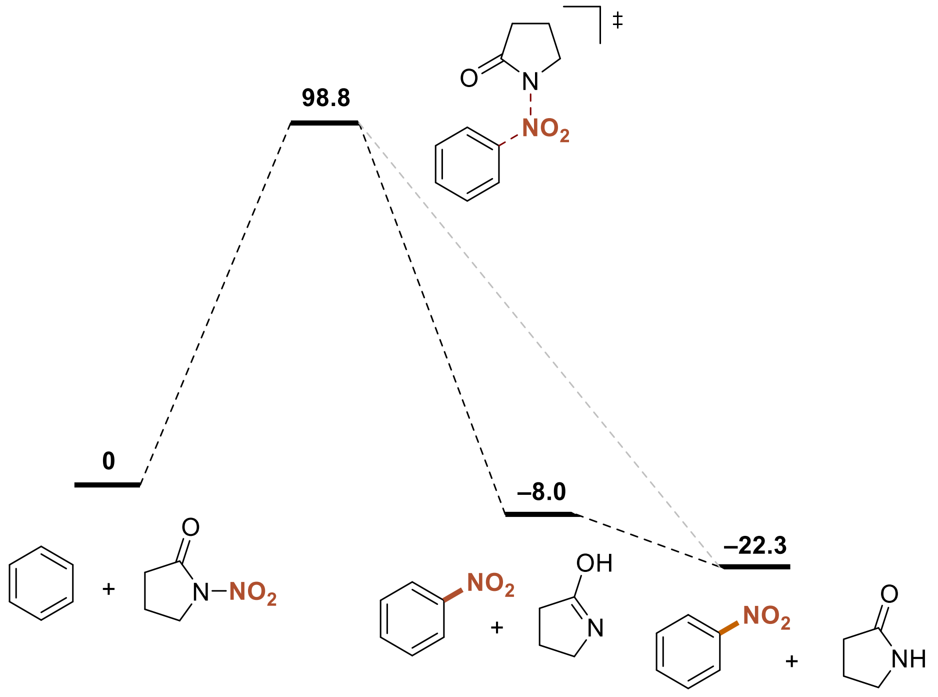

Supplementary Figure 17. Reaction profile for the nitration of benzene by reagent **1**.

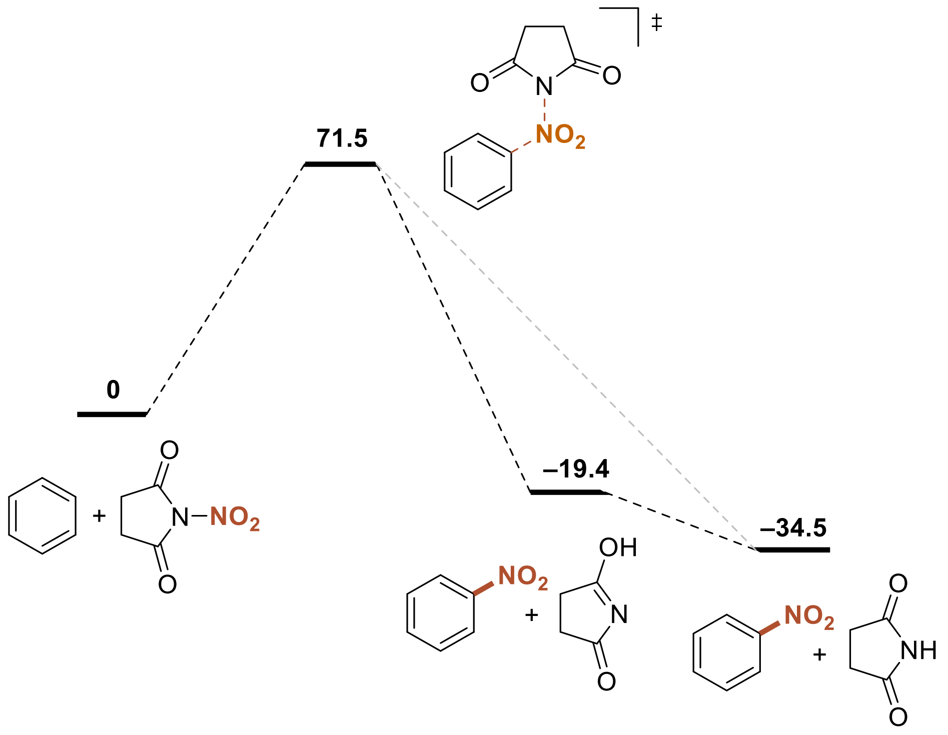

Supplementary Figure 18. Reaction profile for the nitration of benzene by reagent **2**.

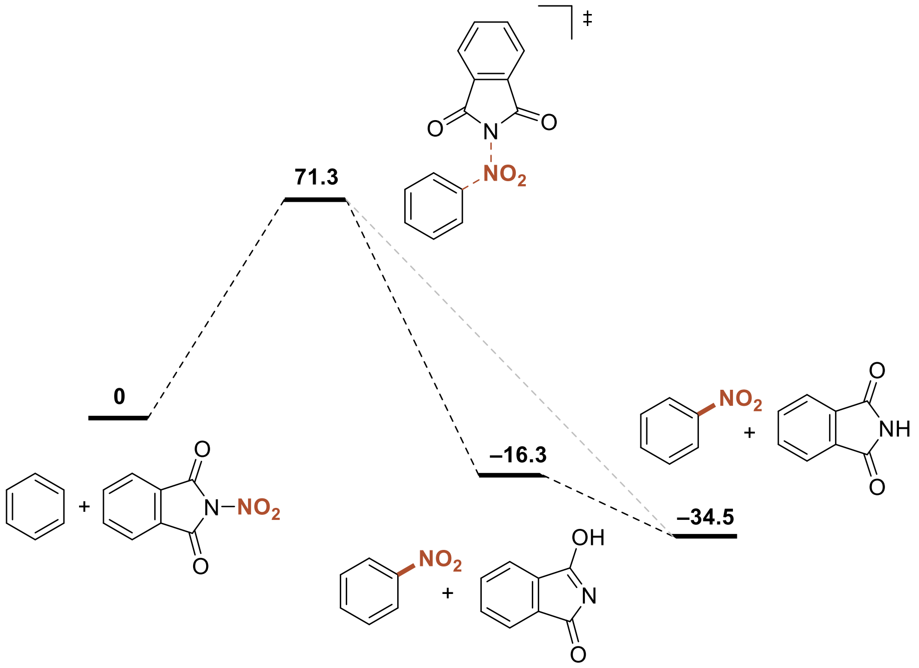

Supplementary Figure 19. Reaction profile for the nitration of benzene by reagent **3**.

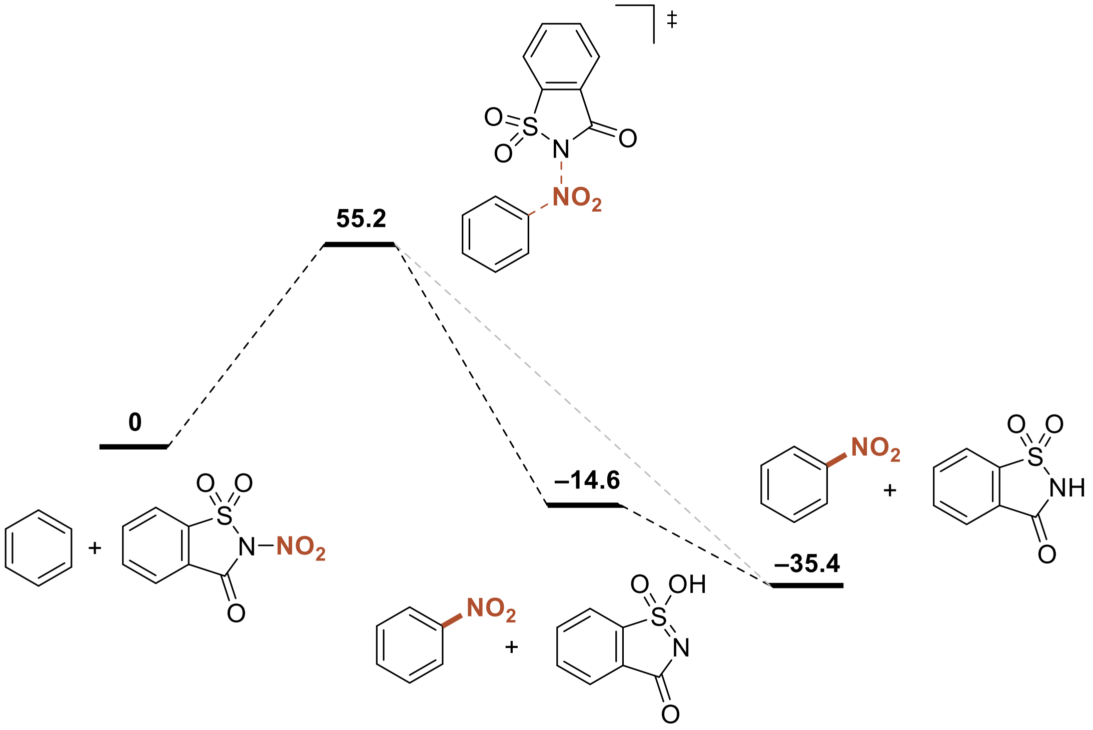

Supplementary Figure 20. Reaction profile for the nitration of benzene by reagent **4a**.

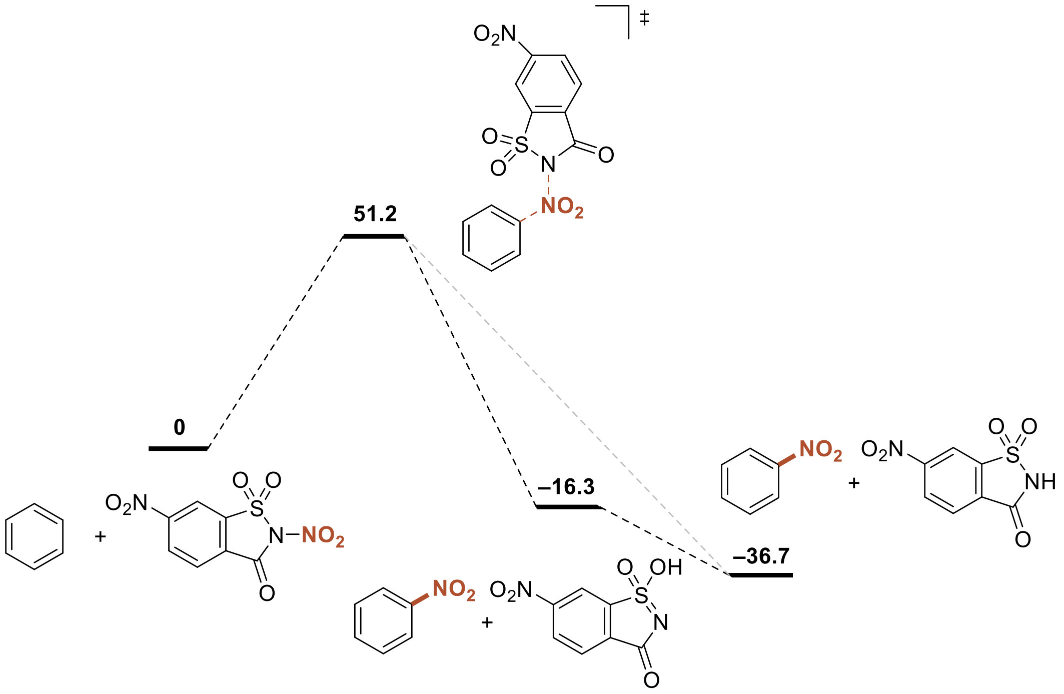

Supplementary Figure 21. Reaction profile for the nitration of benzene by **4b**.

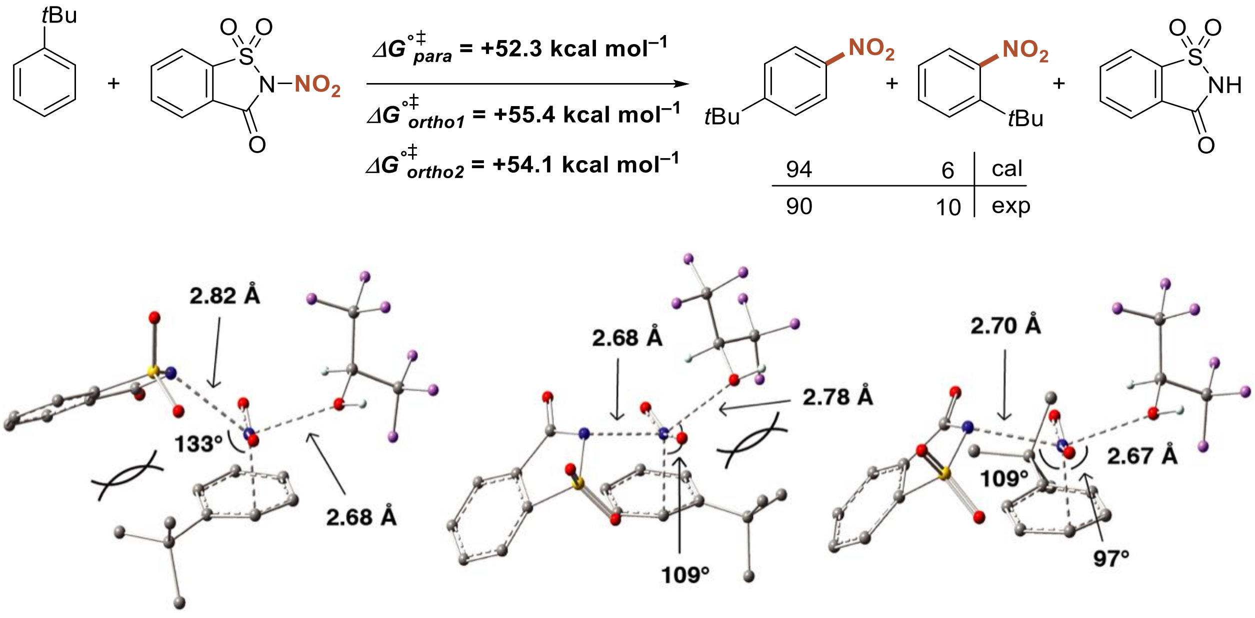

Supplementary Figure 22. Calculated *ortho* : *para* selectivity according to TS's distribution.

Calculated *ortho:para* selectivity in the nitration of tert-butylbenzene: Curious about the high *para*:*ortho* ratio in the nitration of *tert*-butyl-benzene, we calculated the selectivity according to the

transition state distribution. A good match was found between the experimental and calculated ratios, and the high *para* selectivity can be explained by steric hindrance in the transition state, whereby **4a** shields one *ortho* position, and the HFIP molecule the other. The *ortho*:*para* selectivity is explained by the steric hindrance of the nitrating agent, which shields the ortho1 position, and HFIP, which shields the ortho2 position in the TS.

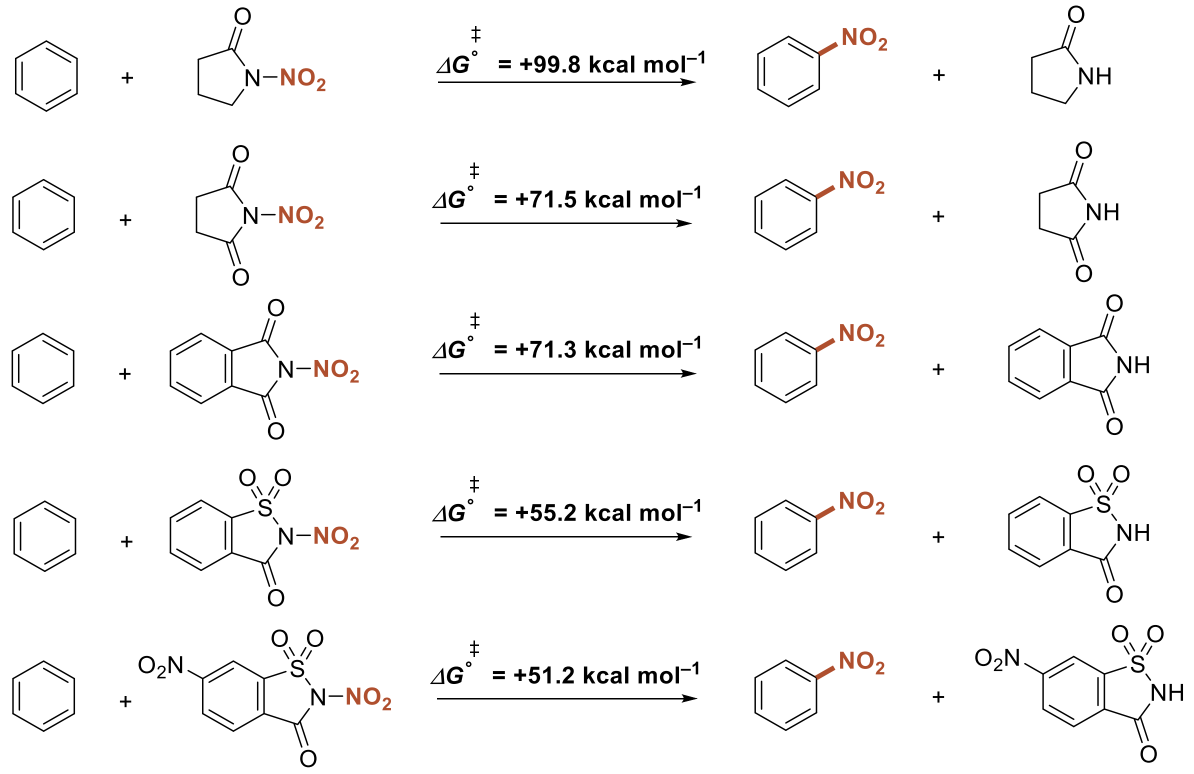

Supplementary Figure 23. Dissociation Gibbs free energies ($\Delta G°$, in kcal mol^{-1}) for **1-4**.

Supplementary Table 14. Intrinsic reaction coordinate for TS(1)_**4a**

Reaction Coordinate (amu$^{1/2}$ Bohr)	Energy (Hartree)
0	–2173.295266
0.2710	–2173.323995
0.6514	–2173.349398
1.0317	–2173.367698
1.4115	–2173.380356
1.7907	–2173.388732
2.1691	–2173.394066
2.5464	–2173.397385
2.9228	–2173.399506
3.3005	–2173.401032
3.6799	–2173.402257
4.0590	–2173.403218
4.4361	–2173.403913
4.8144	–2173.404454
5.1950	–2173.404919
5.5757	–2173.405318
5.9565	–2173.405662
6.3372	–2173.405954
6.7177	–2173.406201
7.0979	–2173.406409
7.4779	–2173.406585
7.8579	–2173.406740
8.2382	–2173.406880
8.6187	–2173.407011
8.9993	–2173.407137
9.3791	–2173.407260
9.7608	–2173.407382
10.1416	–2173.407505
10.5223	–2173.407632
10.9032	–2173.407763
11.2840	–2173.407900
11.6649	–2173.408044
12.0458	–2173.408195
12.4266	–2173.408355
12.8075	–2173.408523
13.1884	–2173.408701
13.5693	–2173.408888
13.9502	–2173.409086
14.3310	–2173.409296
14.7119	–2173.409518
15.0928	–2173.409752
15.4737	–2173.409998
15.8546	–2173.410257
16.2355	–2173.410531
16.6164	–2173.410821
16.9973	–2173.411126

17.3782	–2173.411447
17.7590	–2173.411783
18.1399	–2173.412131
18.5208	–2173.412488
18.9017	–2173.412853
19.2826	–2173.413229
19.6635	–2173.413616
20.0444	–2173.414019
20.4252	–2173.414442
20.8061	–2173.414887
21.1870	–2173.415358
21.5678	–2173.415857
21.9487	–2173.416385
22.3294	–2173.416939
22.7105	–2173.417516
23.0914	–2173.418113
23.4722	–2173.418733
23.8531	–2173.419379
24.2340	–2173.420053
24.6149	–2173.420753
24.9957	–2173.421474
25.3765	–2173.422215
25.7574	–2173.422979
26.1382	–2173.423770
26.5189	–2173.424595
26.8995	–2173.425478
27.2782	–2173.426514
27.6378	–2173.428733
28.0092	–2173.437199
28.3883	–2173.453135
28.7284	–2173.461133
29.1059	–2173.465812
29.4840	–2173.469246
29.8637	–2173.472058
30.2440	–2173.474499
30.6245	–2173.476682
31.0052	–2173.478637
31.3859	–2173.480401
31.7666	–2173.481997
32.1474	–2173.483446
32.5281	–2173.484765
32.9089	–2173.485965
33.2894	–2173.487069
33.6690	–2173.488129
34.0481	–2173.489218
34.4273	–2173.490528
34.8062	–2173.492169
35.1829	–2173.494056
35.5551	–2173.495636

35.9237	–2173.496706
36.3011	–2173.497484
36.6808	–2173.498121
37.0612	–2173.498678
37.4417	–2173.499171
37.8221	–2173.499604
38.2025	–2173.499986
38.5829	–2173.500325
38.9635	–2173.500634
39.3442	–2173.500919
39.7250	–2173.501183
40.1058	–2173.501430
40.4866	–2173.501661
40.8674	–2173.501879
41.2482	–2173.502085
41.6290	–2173.502282
42.0099	–2173.502471
42.3907	–2173.502653
42.7716	–2173.502829
43.1525	–2173.502999
43.5534	–2173.503165
43.9142	–2173.503326
44.2951	–2173.503483
44.6760	–2173.503637
45.0569	–2173.503788
45.4378	–2173.503936
45.8186	–2173.504082
46.1996	–2173.504225
46.5804	–2173.504365
46.9614	–2173.504501
47.3423	–2173.504635
47.7232	–2173.504766
48.1041	–2173.504894
48.4850	–2173.505020
48.8659	–2173.505143
49.2468	–2173.505265
49.6277	–2173.505386
50.0086	–2173.505505
50.3895	–2173.505623
50.7704	–2173.505740
51.1513	–2173.505856
51.5322	–2173.505971
51.9131	–2173.506084
52.2940	–2173.506195
52.6750	–2173.506305
53.0559	–2173.506413
53.4368	–2173.506519
53.8177	–2173.506623
54.1986	–2173.506726

54.5795	–2173.506827
54.9604	–2173.506927
55.3413	–2173.507025
55.7222	–2173.507123
56.1031	–2173.507220
56.4840	–2173.507317
56.8649	–2173.507412
57.2458	–2173.507506
57.7222	–2173.507598
58.0076	–2173.507688
58.3885	–2173.507776
58.7694	–2173.507861
59.1503	–2173.507943
59.5312	–2173.508024
59.9121	–2173.508102
60.2930	–2173.508180
60.6739	–2173.508256
61.0548	–2173.508331
61.4357	–2173.508404
61.8166	–2173.508475
62.1975	–2173.508544
62.5784	–2173.508612
62.9593	–2173.508678
63.3402	–2173.508743
63.7211	–2173.508807
64.1020	–2173.508871
64.4829	–2173.508934
64.8638	–2173.508998
65.2447	–2173.509061
65.6256	–2173.509124
66.0065	–2173.509187
66.3874	–2173.509250
66.7683	–2173.509313
67.1492	–2173.509374
67.5301	–2173.509435
67.9110	–2173.509496
68.2919	–2173.509555
68.6728	–2173.509613

Supplementary Table 15. Gibbs free energies and imaginary frequencies of TS

Compound		Gibbs Free Energy (Hartree)	Imaginary Frequency (cm^{-1})
**TS(1)_1**		–1512.823578	266.49
**TS(2)_1**		–1512.821915	268.54
**TS(3)_1**		–1512.821702	334.37
**TS(1)_2**		–1586.915456	182.20
**TS(2)_2**		–1586.915349	214.88
**TS(3)_2**		–1586.915151	194.68
**TS(1)_3**		–1739.305953	178.55
**TS(2)_3**		–1739.305376	201.75
**TS(3)_3**		–1739.304258	208.00
**TS(1)_4a**		–2174.590332	151.69
**TS(2)_4a**		–2174.589646	182.51
**TS(3)_4a**		–2174.589153	150.77
**TS(4)_4a**		–2174.589147	136.11
**TS(5)_4a**		–2174.589025	133.33
**TS(6)_4a**		–2174.588733	168.21
**TS(7)_4a**		–2174.588654	149.29
**TS(8)_4a**		–2174.588625	163.99
**TS(9)_4a**		–2174.587889	167.61
**TS(10)_4a**		–2174.587724	165.48
**TS(11)_4a**		–2174.587645	190.53
**TS(1)_4b**		–2379.094915	170.73
**TS(2)_4b**		–2379.094751	171.84
**TS(3)_4b**		–2379.094511	162.18
**TS(4)_4b**		–2379.094483	162.72
**TS(5)_4b**		–2379.094215	144.81
**TS(6)_4b**		–2379.094033	143.72
**TS(7)_4b**		–2379.094021	159.70
**TS(8)_4b**		–2379.094004	161.37
**TS(9)_4b**		–2379.093079	181.51
**TS(10)_4b**		–2379.093070	153.11
**TS(o-*t*Bu1)_4a**		–2331.713185	183.50
**TS(o-*t*Bu2)_4a**		–2331.715297	239.13
**TS(p-*t*Bu)_4a**		–2331.718123	276.56

Supplementary Table 16. Gibbs free energies of intermediates.

Compound	Gibbs Free Energy (Hartree)
**1**	–491.009802
**2**	–565.058118
**3**	–717.448344
**4a**	–1152.706933
**4b**	–1357.205225
**Benzene**	–232.144466
**HFIP**	–789.826835
**1(1)+HFIP**	–1280.828949
**1(2)+HFIP**	–1280.828666
**1(3)+HFIP**	–1280.827859
**1(4)+HFIP**	–1280.827766
**2(1)+HFIP**	–1354.873518
**2(2)+HFIP**	–1354.873316
**3(1)+HFIP**	–1507.264823
**3(2)+HFIP**	–1507.264724
**4a(1)+HFIP**	–1942.523866
**4a(2)+HFIP**	–1942.523512
**4a(3)+HFIP**	–1942.522832
**4a(4)+HFIP**	–1942.522781
**4b(1)+HFIP**	–2147.021660
**4b(2)+HFIP**	–2147.021595
**4b(3)+HFIP**	–2147.020759
**4b(4)+HFIP**	–2147.020178
**1–H**	–286.534833
**2–H**	–360.602657
**3–H**	–512.992849
**4a–H**	–948.252796
**4b–H**	–1152.753289
**Benzene–NO_2**	–436.654962
**1–**	–286.044515
**2–**	–360.138054
**3–**	–512.530228
**4a–**	–947.813404
**4b–**	–1152.320276
**NO_2+**	–204.826764
**1–H(isomer)**	–286.511989
**2–H(isomer)**	–360.578503
**3–H(isomer)**	–512.963753
**4a–H(isomer)**	–948.219687
**4b–H(isomer)**	–1152.720777
***t*Bu–Benzene**	–389.267737
***t*Bu-Benzene–NO_2(*ortho*)**	–593.765755
***t*Bu-Benzene–NO_2(*para*)**	–593.779753

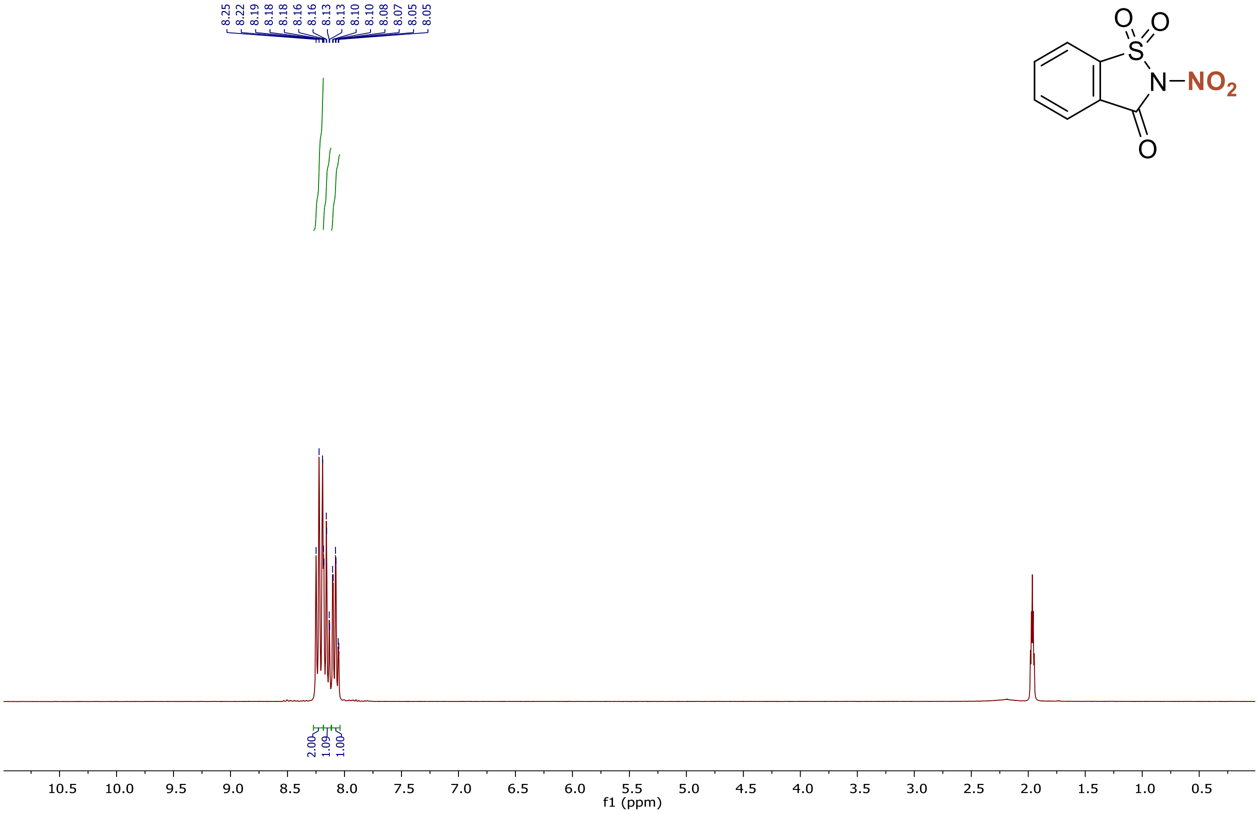

Supplementary Figure 24. ^{1}H NMR spectra for **4a**.

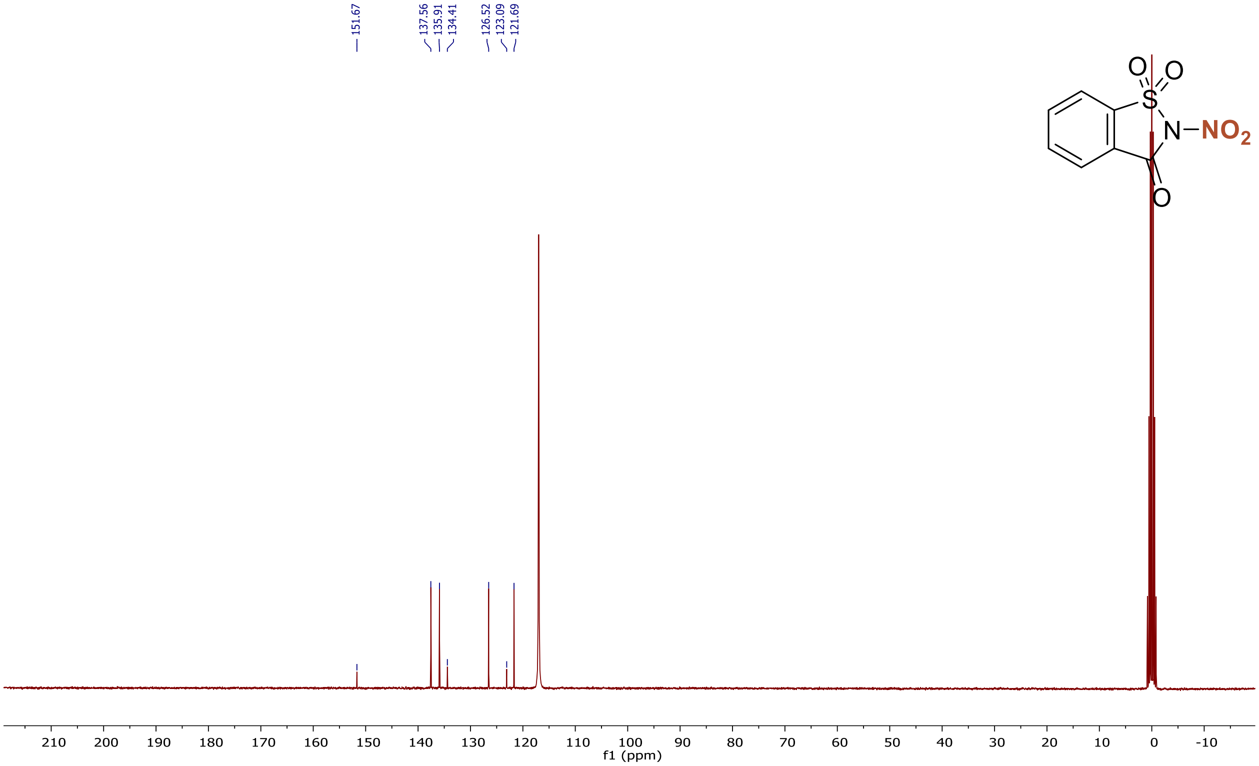

Supplementary Figure 25. ^{13}C NMR spectra for **4a**.

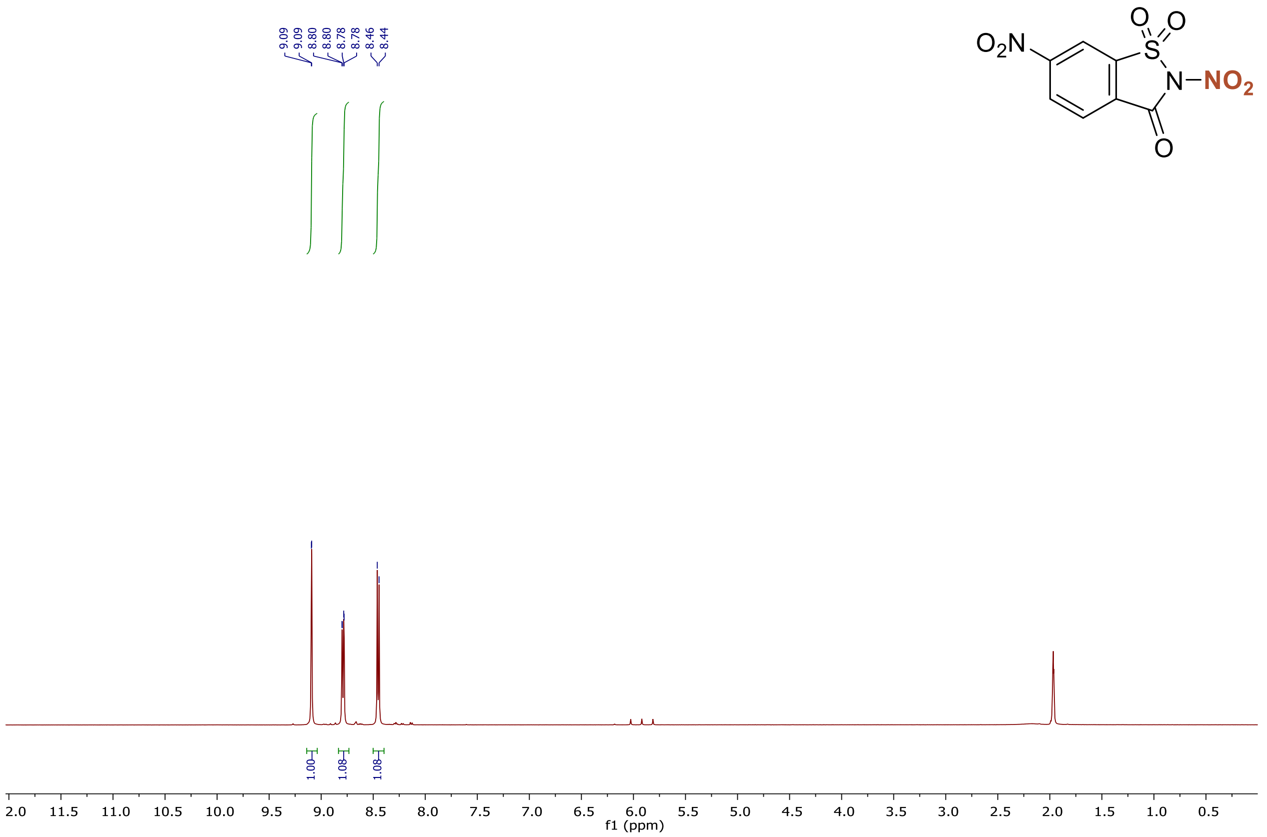

Supplementary Figure 26. ^{1}H NMR spectra for **4b**.

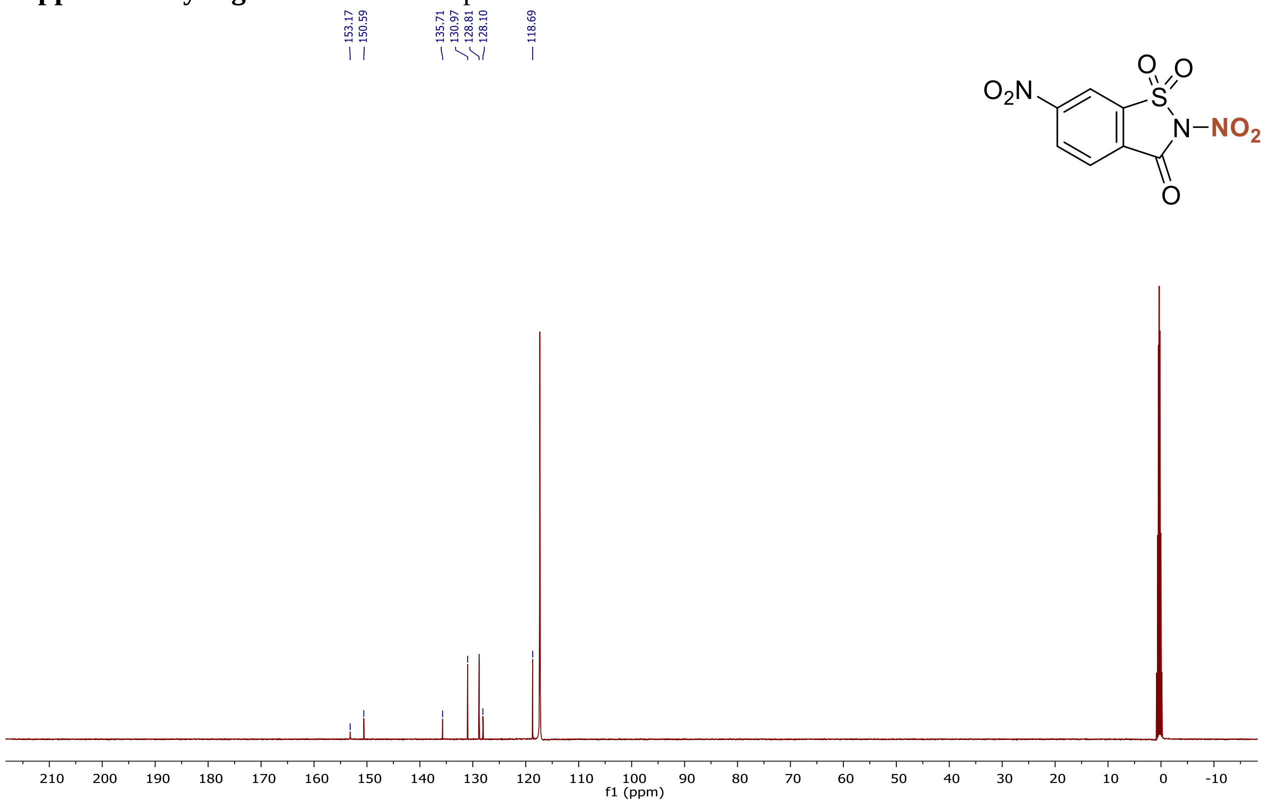

Supplementary Figure 27. ^{13}C NMR spectra for **4b**.

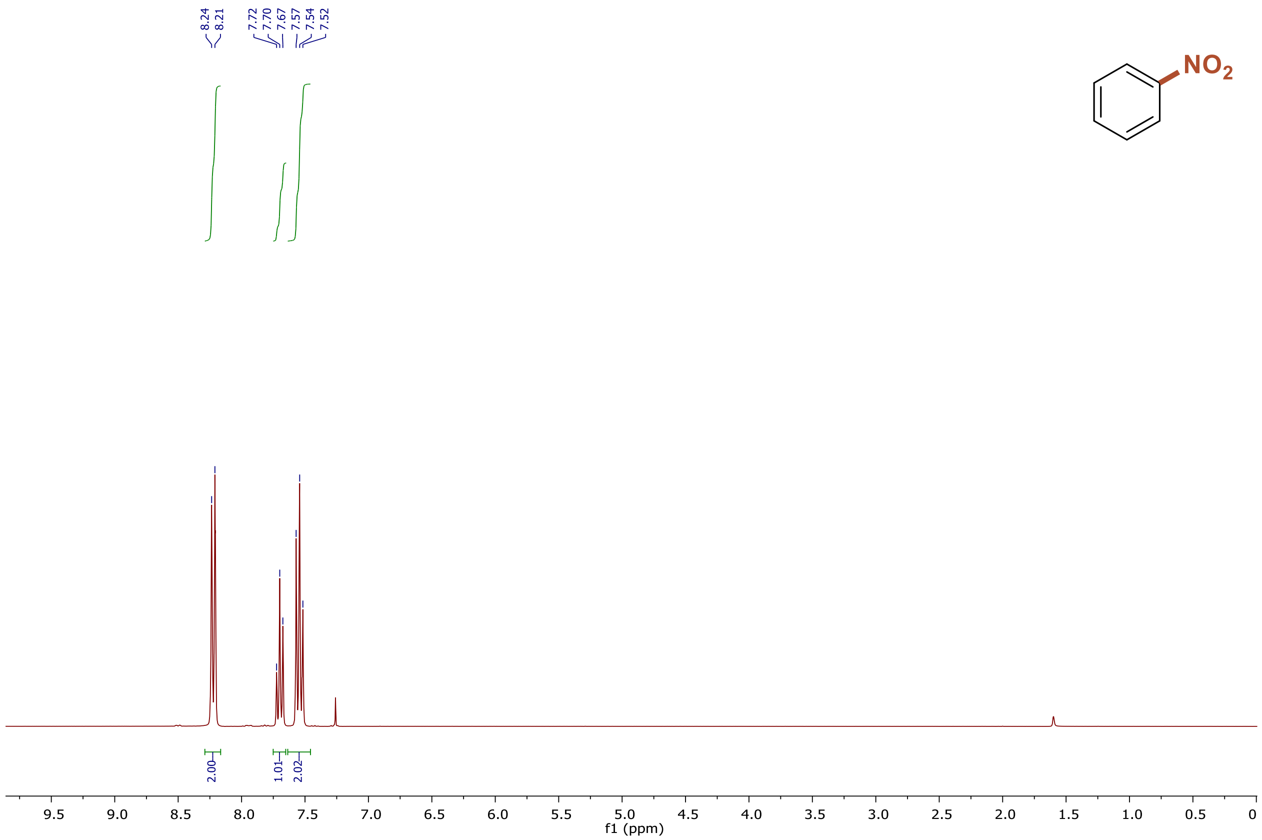

Supplementary Figure 28. ^{1}H NMR spectra for **5**.

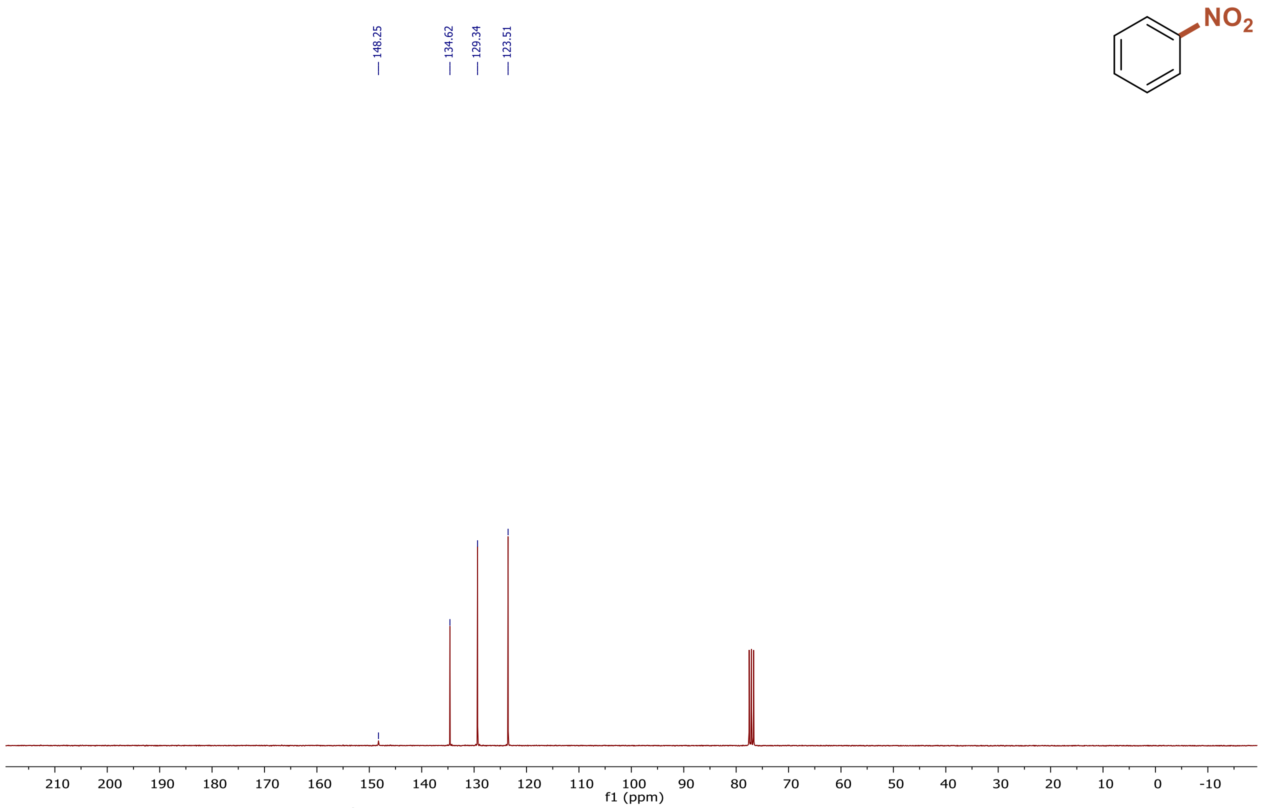

Supplementary Figure 29. ^{13}C NMR spectra for **5**.

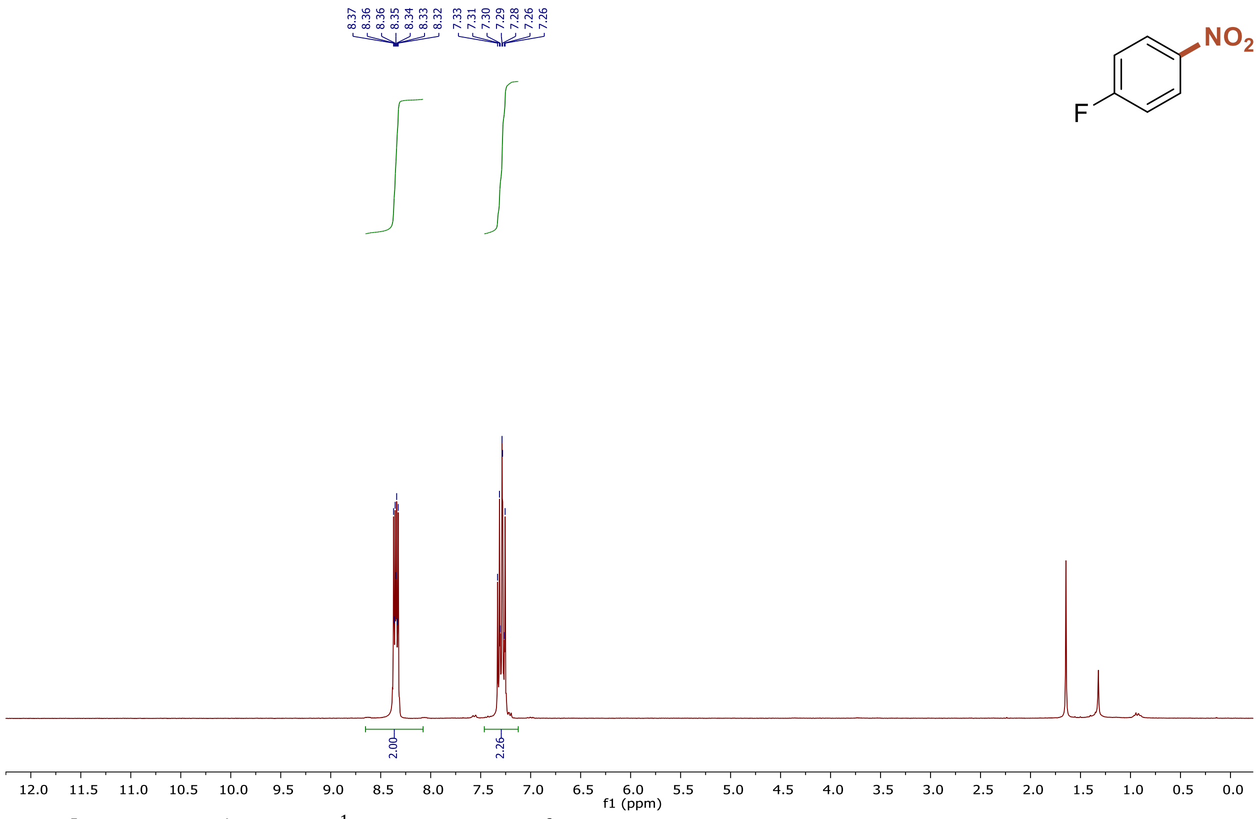

Supplementary Figure 30. ^{1}H NMR spectra for **6A**.

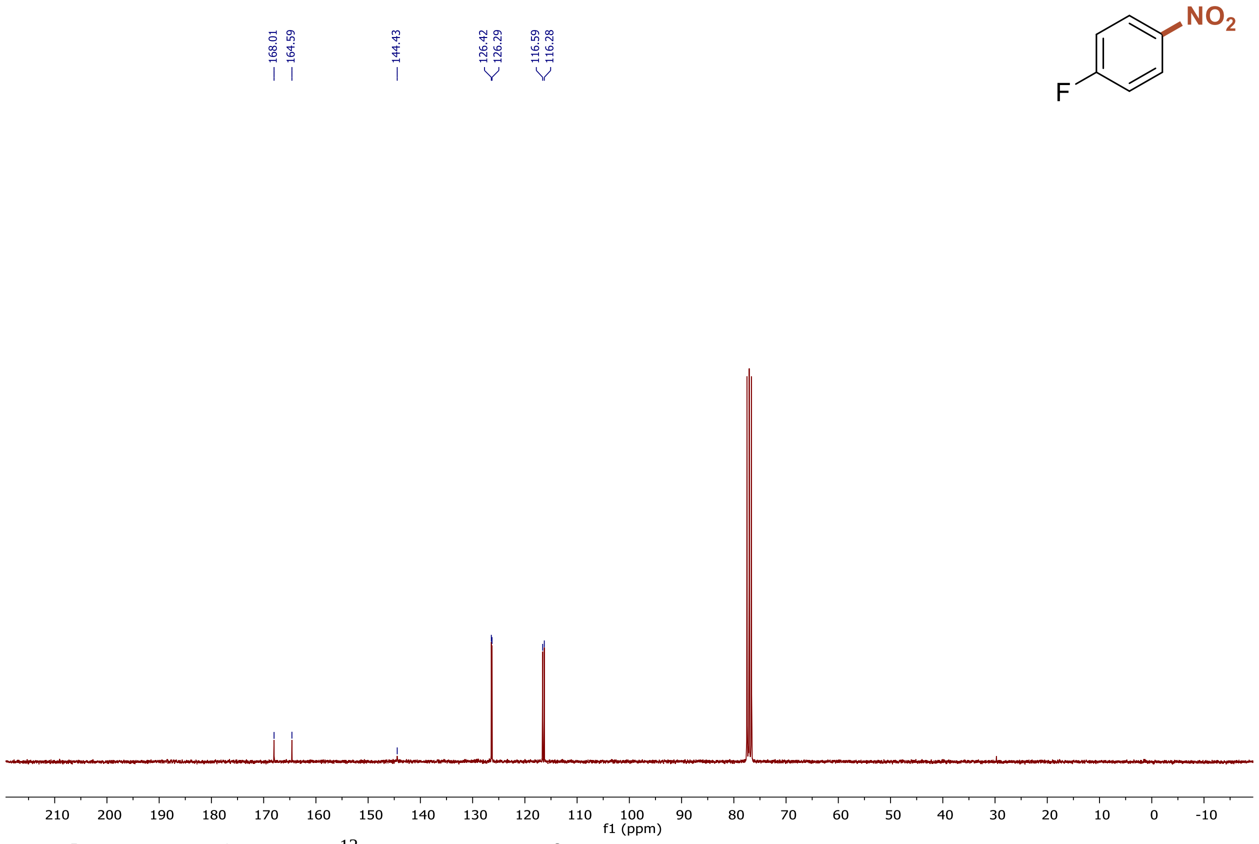

Supplementary Figure 31. ^{13}C NMR spectra for **6A**.

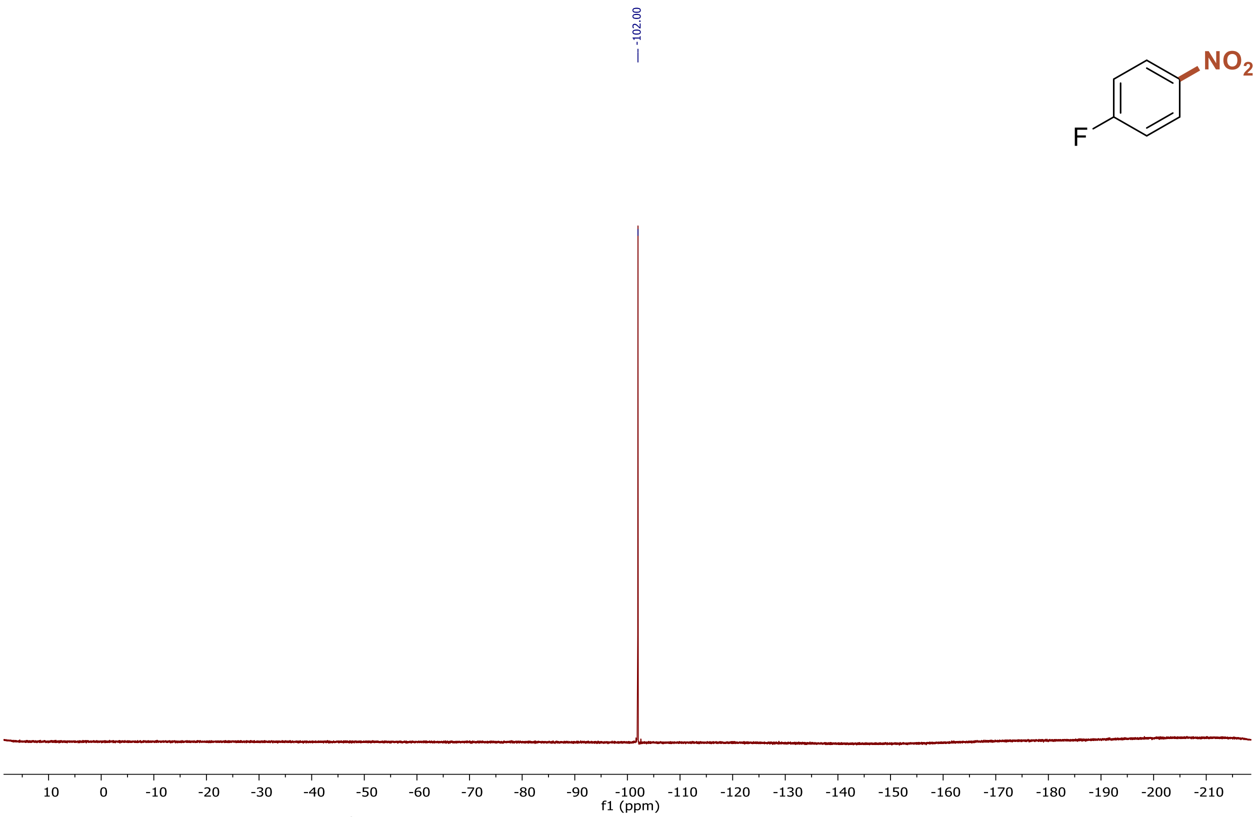

Supplementary Figure 32. ^{19}F-NMR spectra for **6A.**

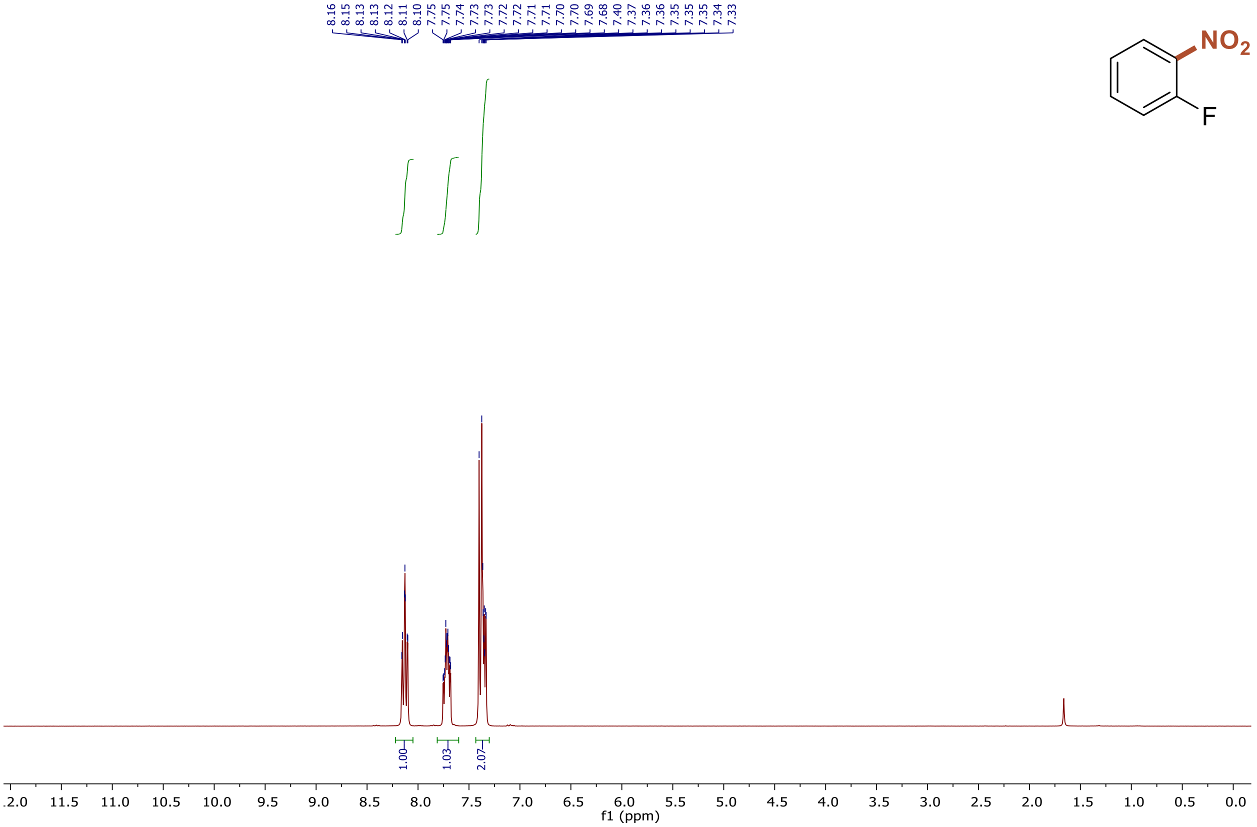

Supplementary Figure 33. ^{1}H NMR spectra for **6B**.

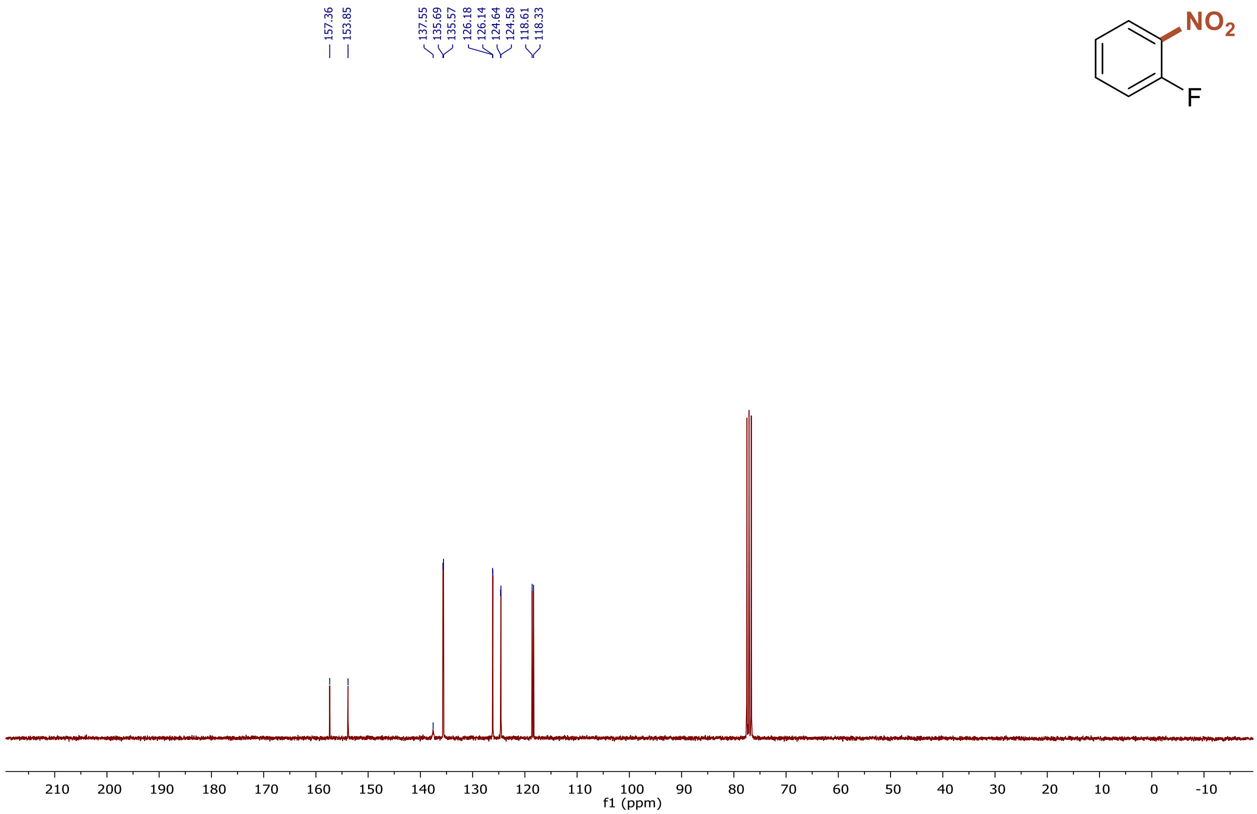

Supplementary Figure 34. ^{1}H NMR spectra for **6B.**

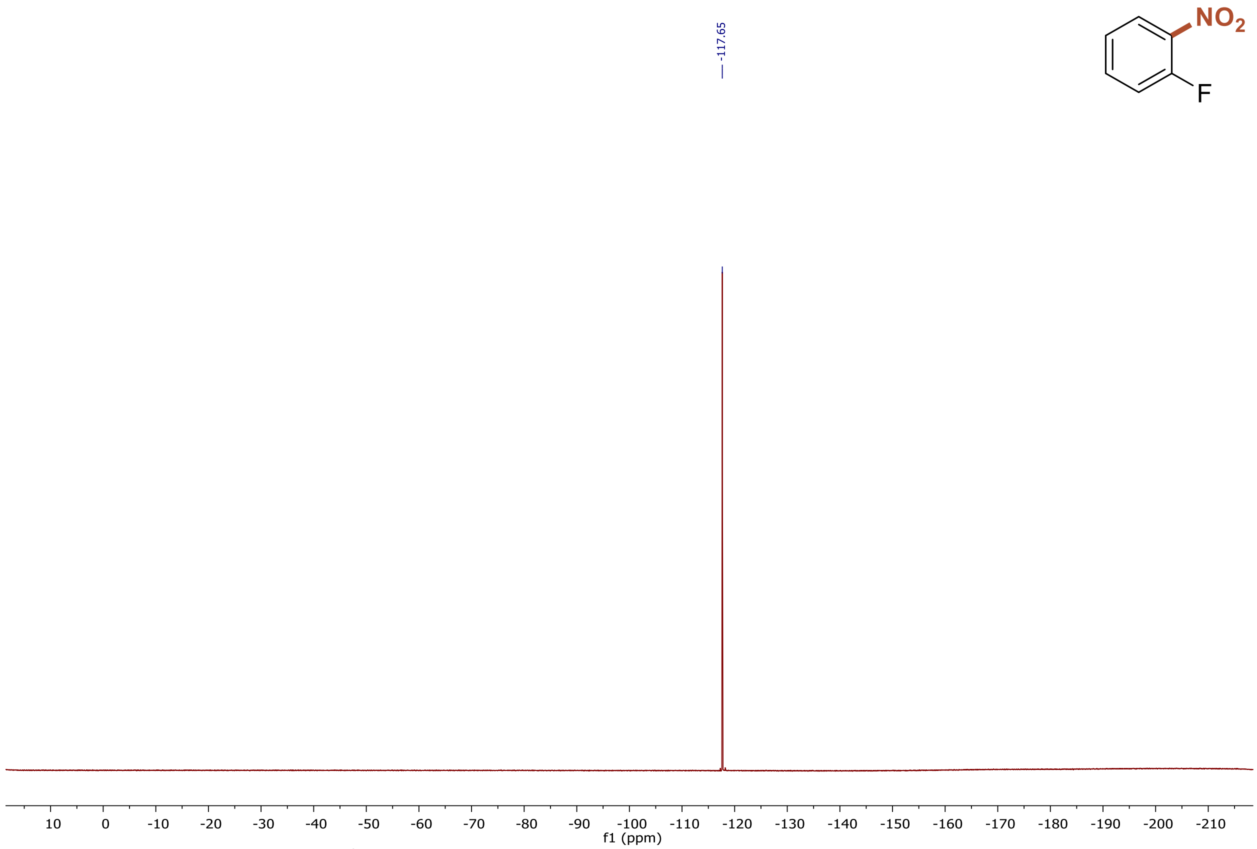

Supplementary Figure 35. ^{19}F NMR spectra for **6B.**

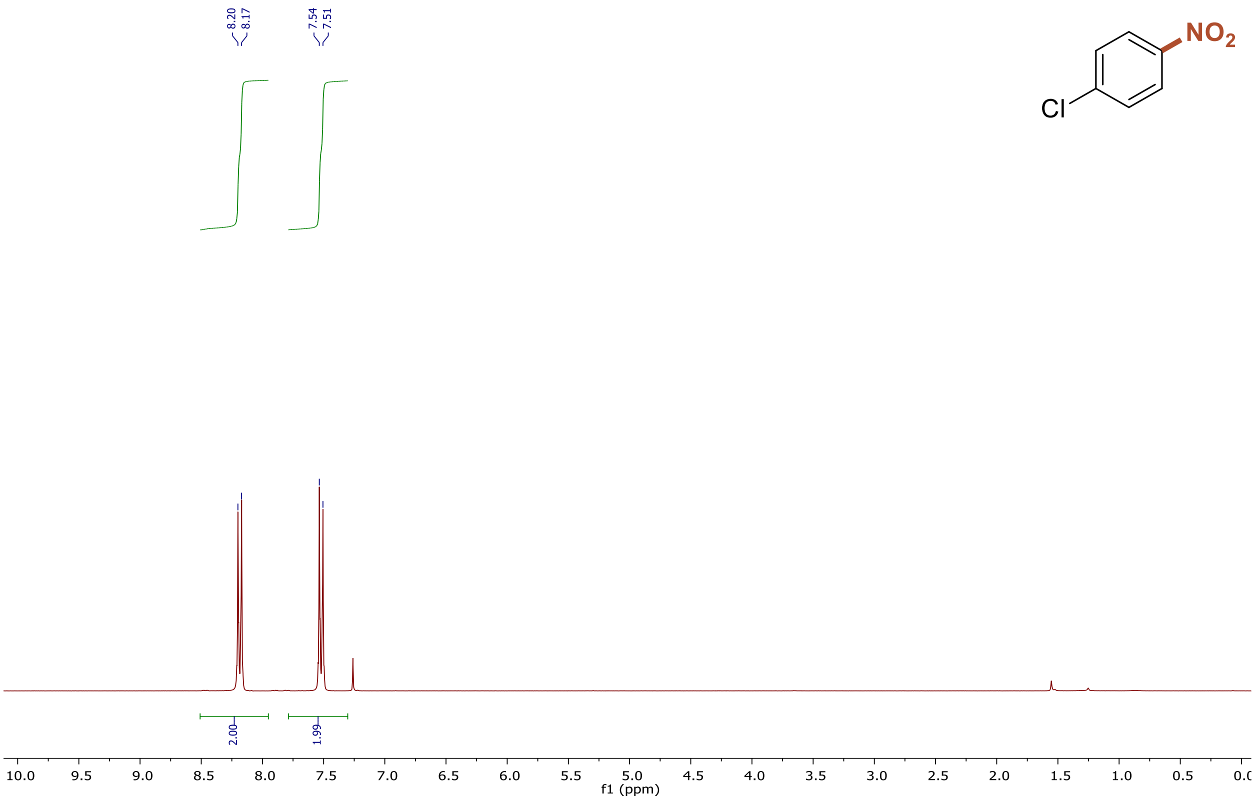

Supplementary Figure 36. ^{1}H NMR spectra for **7A**.

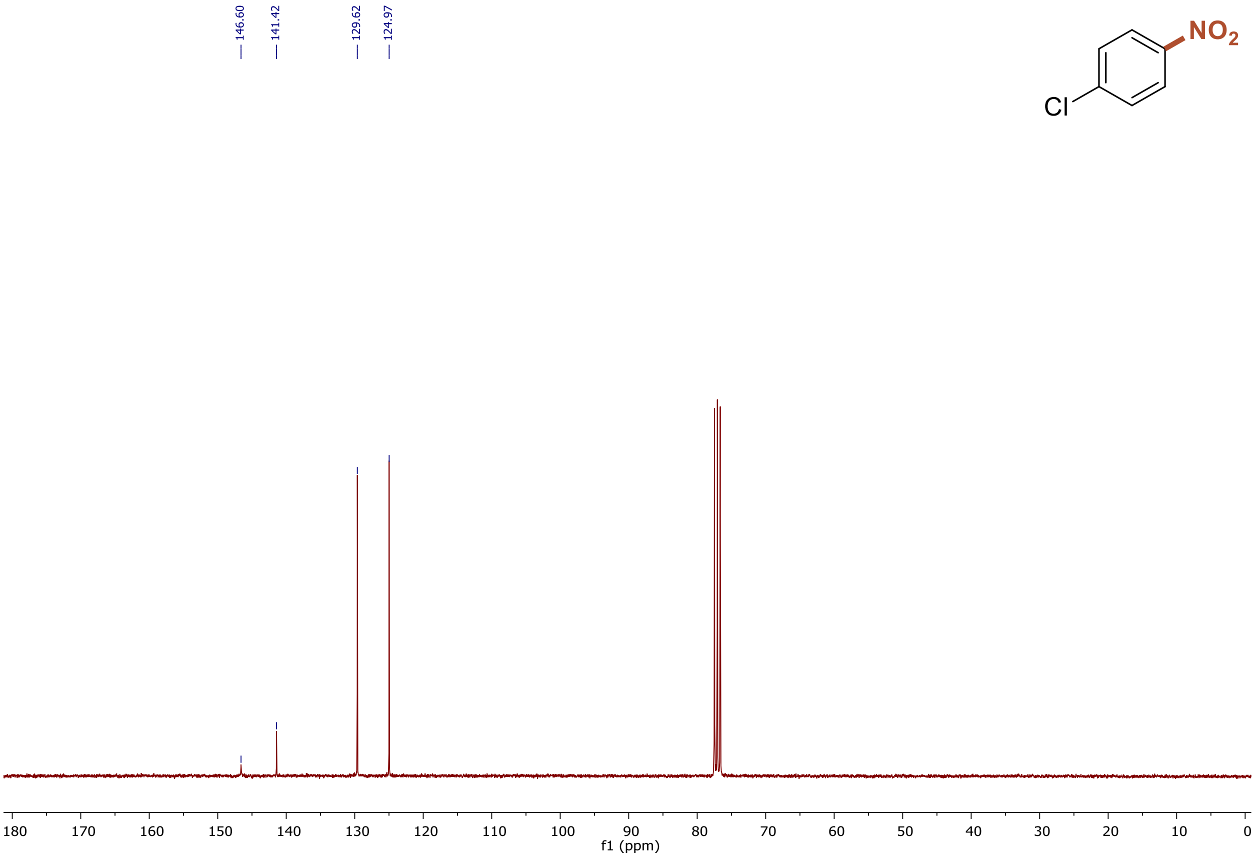

Supplementary Figure 37. ^{13}C NMR spectra for **7A.**

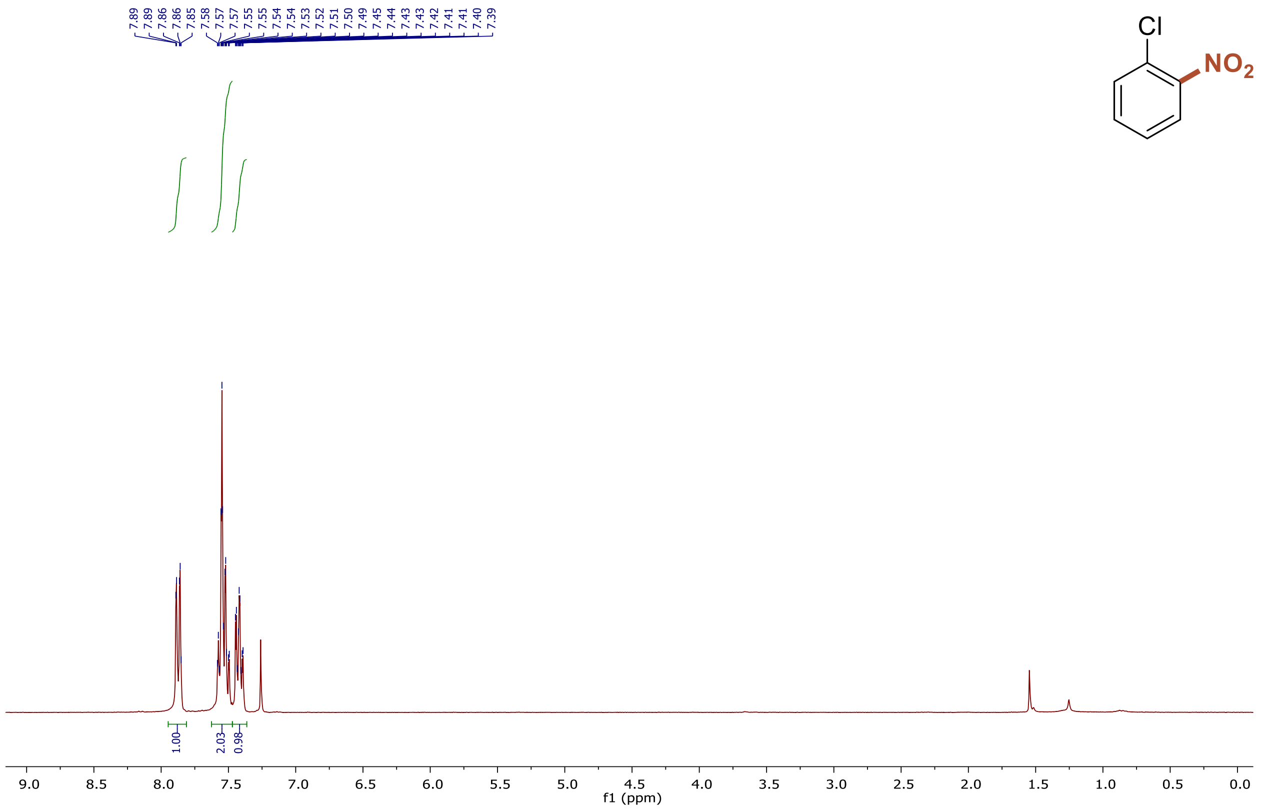

Supplementary Figure 38. ^{1}H NMR spectra for **7A**

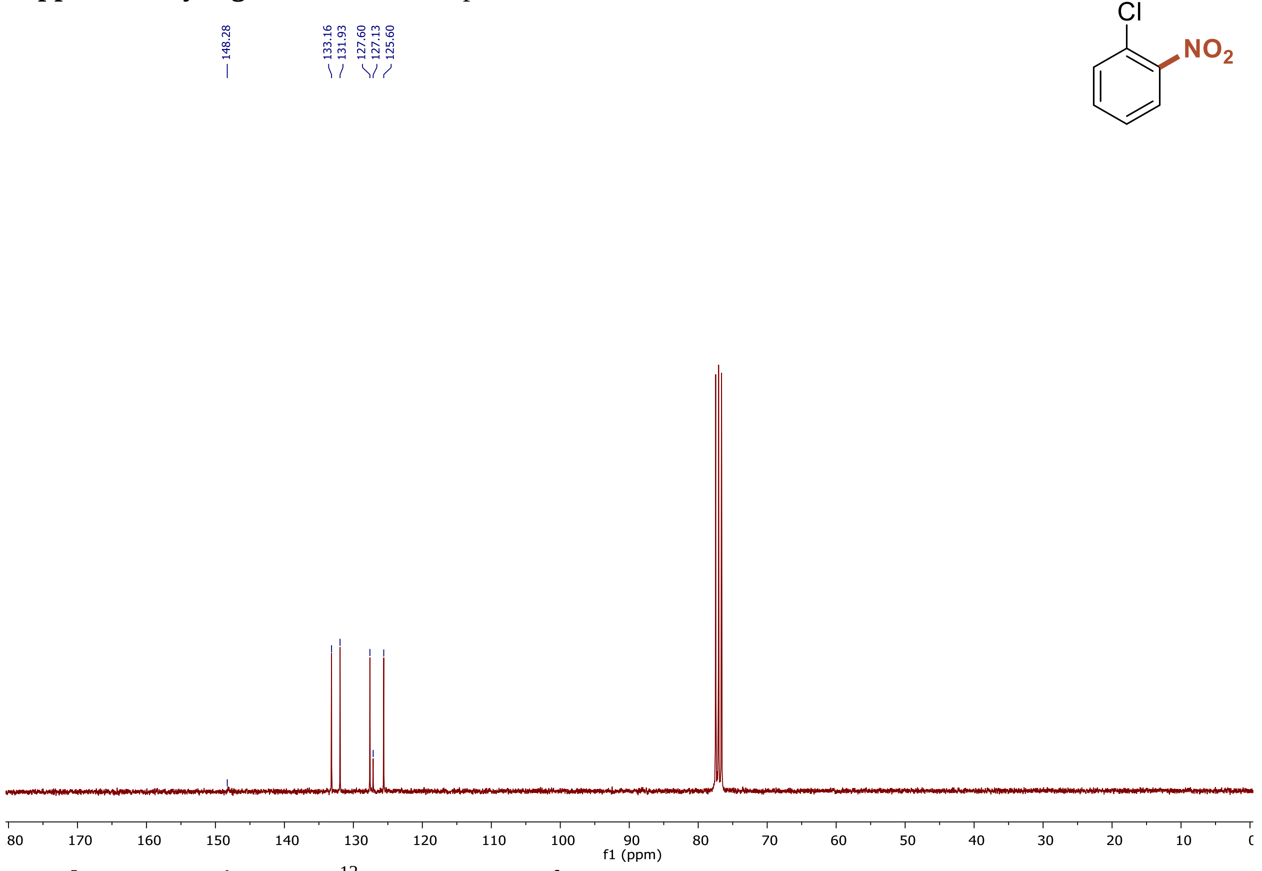

Supplementary Figure 39. ^{13}C NMR spectra for **7A**.

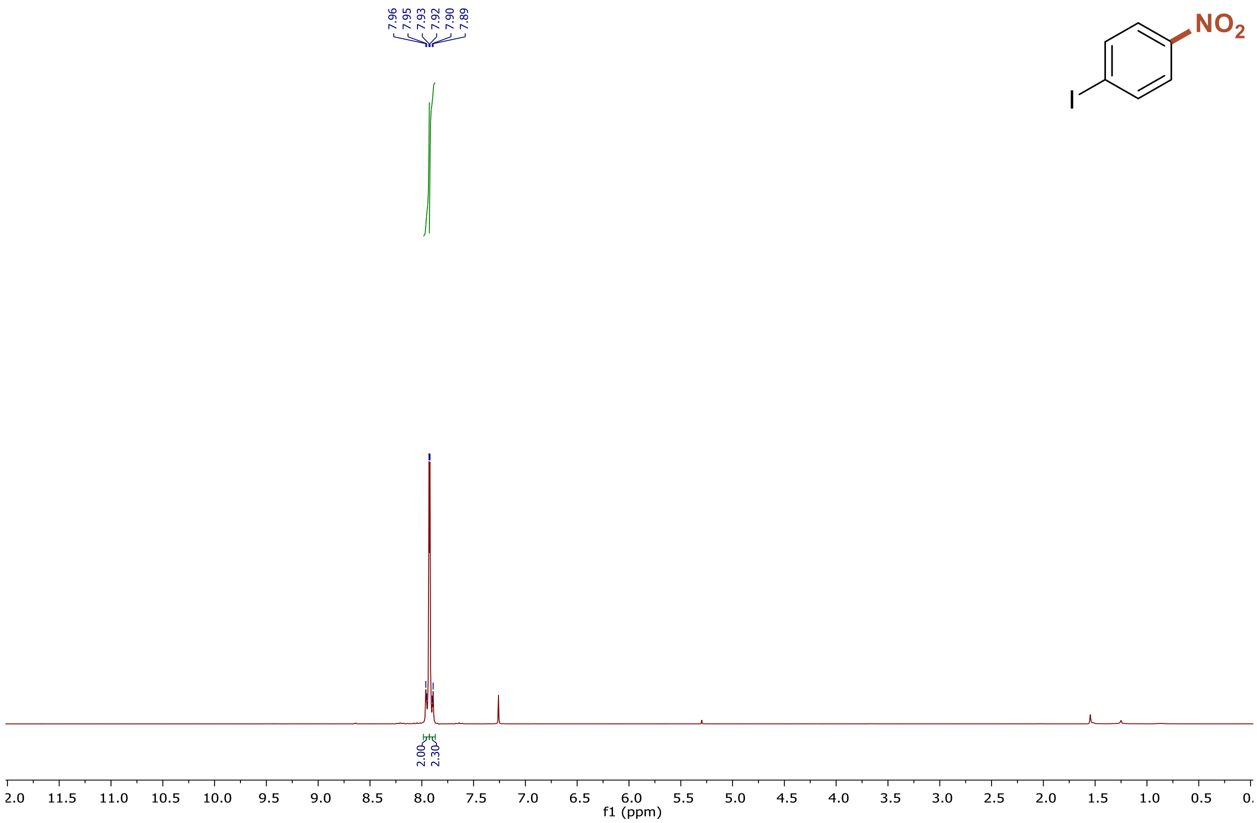

Supplementary Figure 40. ^{1}H NMR spectra for **8A**.

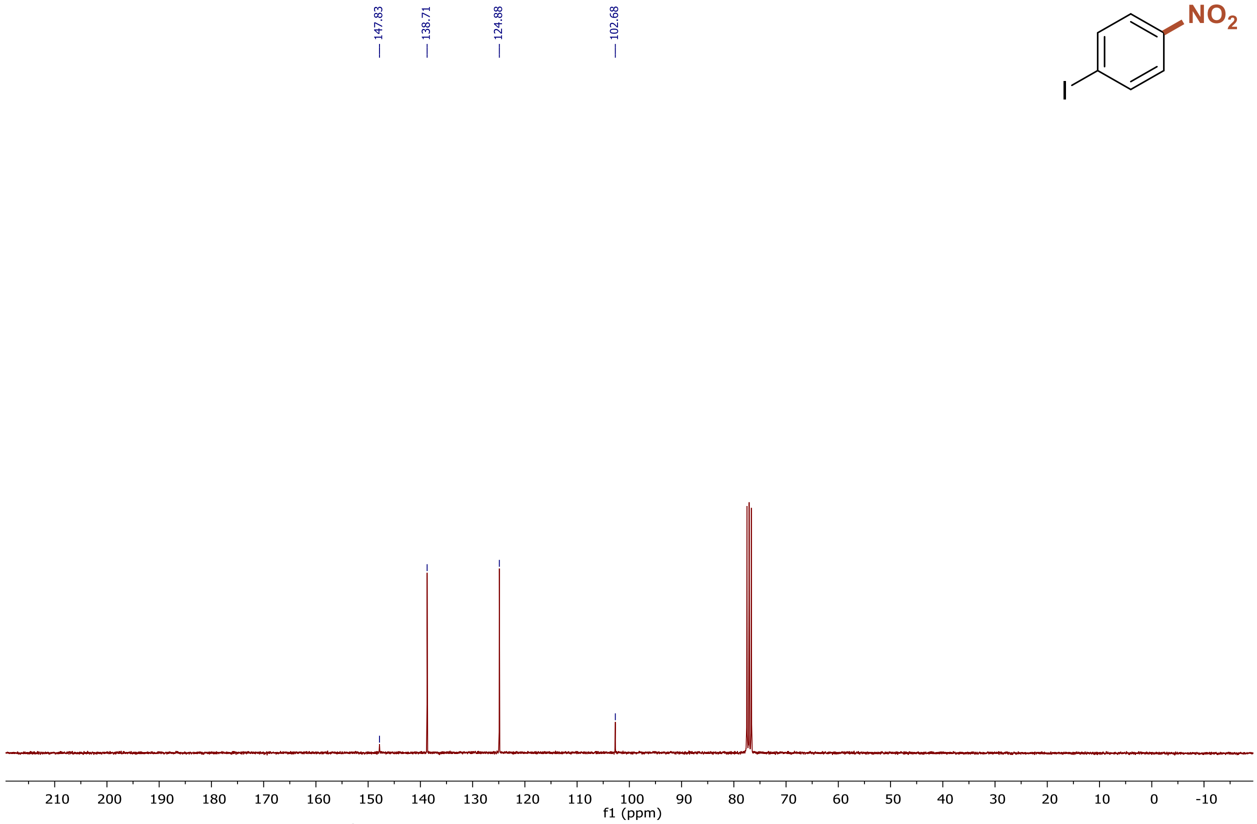

Supplementary Figure 41. ^{13}C NMR spectra for **8A**.

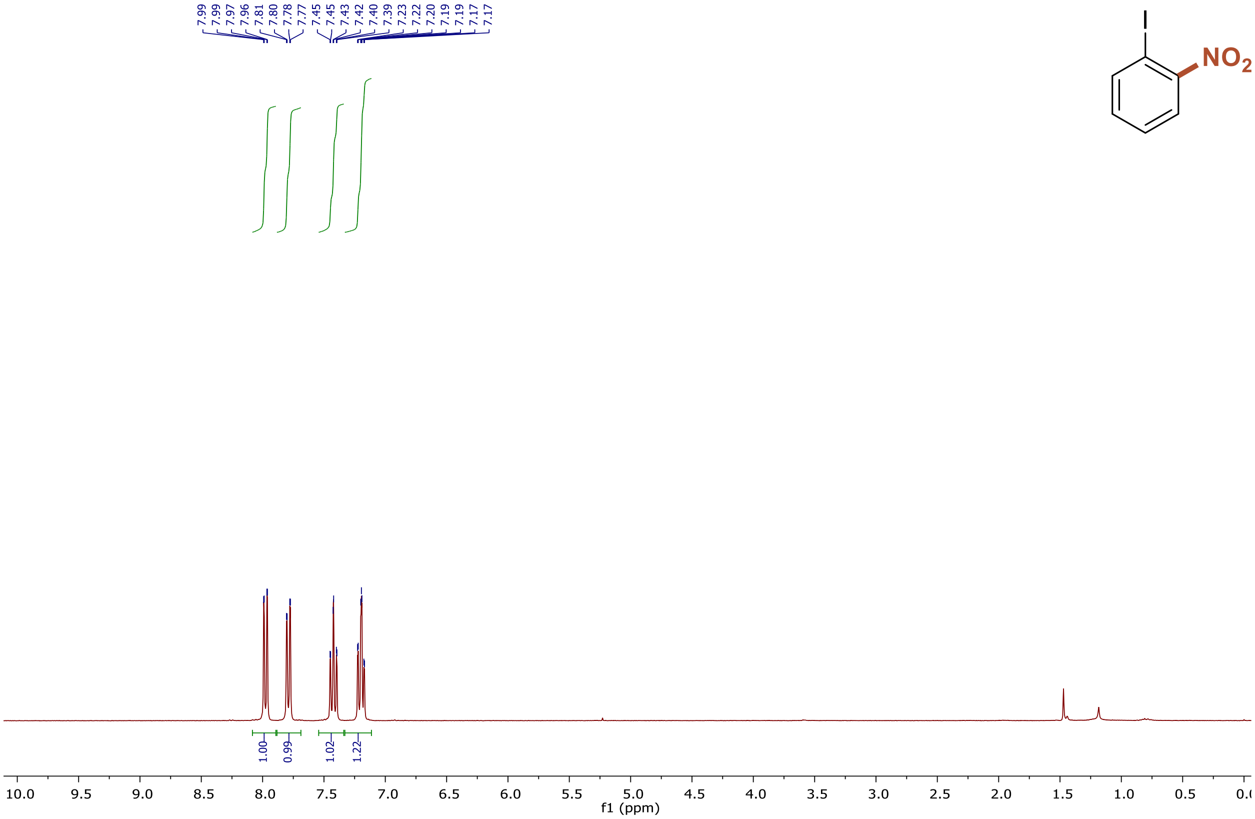

Supplementary Figure 42. ^{1}H NMR spectra for **8B**.

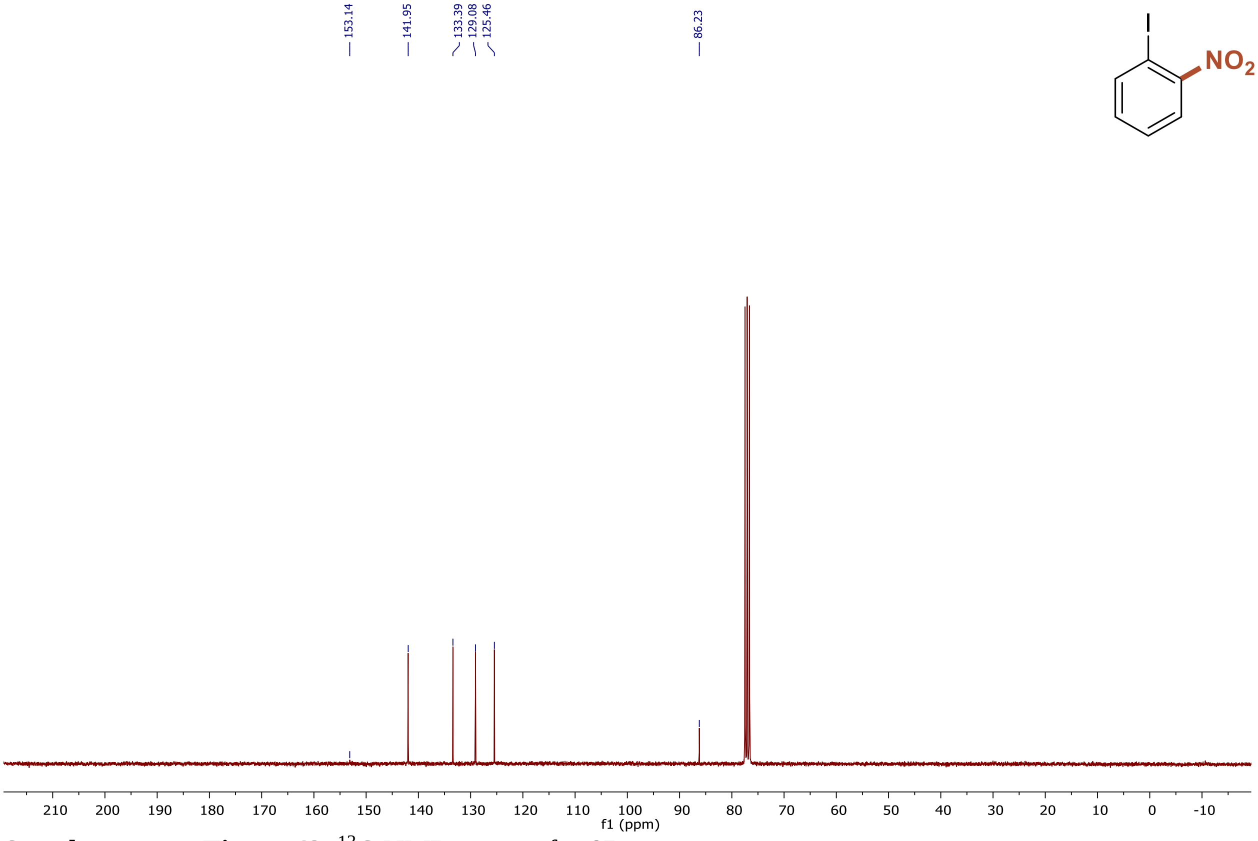

Supplementary Figure 43. ^{13}C NMR spectra for **8B**.

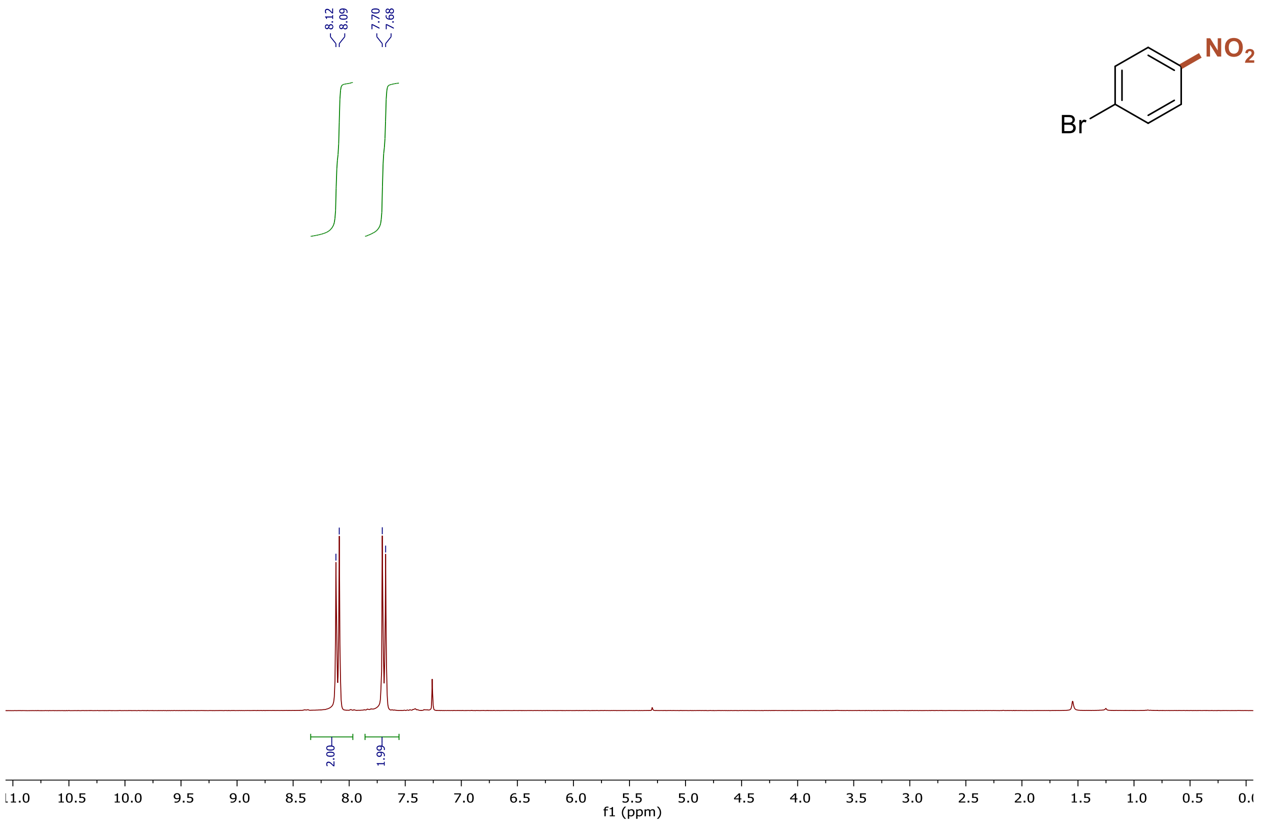

Supplementary Figure 44. ^{1}H NMR spectra for **9A**.

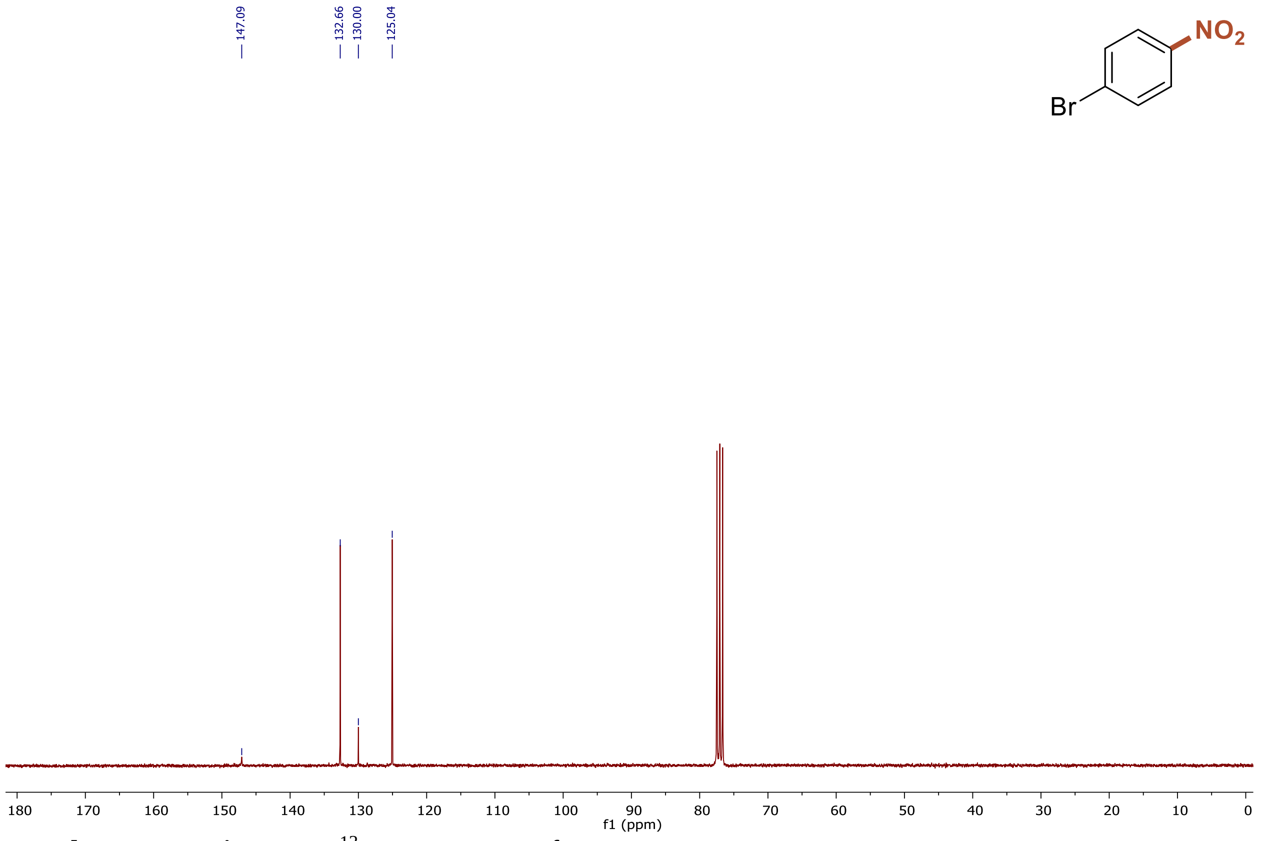

Supplementary Figure 45. ^{13}C NMR spectra for **9A**.

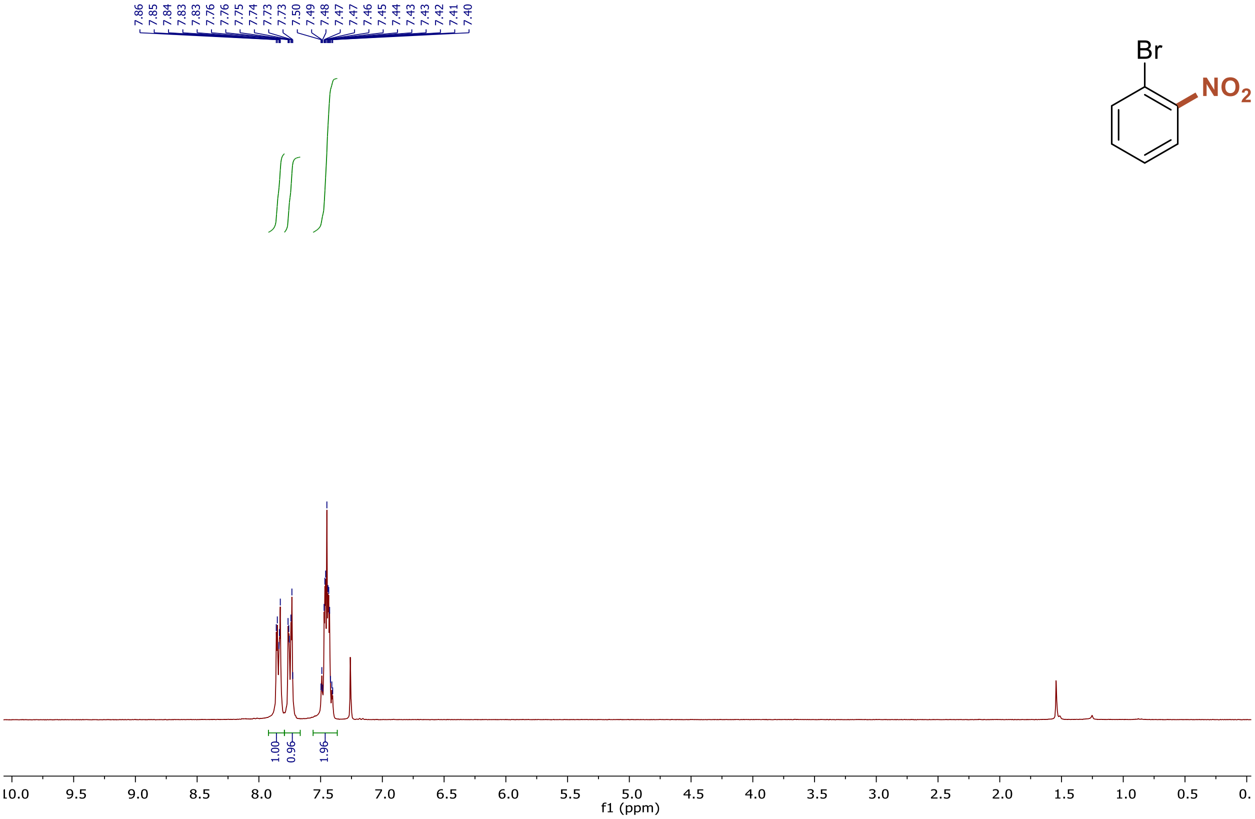

Supplementary Figure 46. ^{1}H NMR spectra for **9B**.

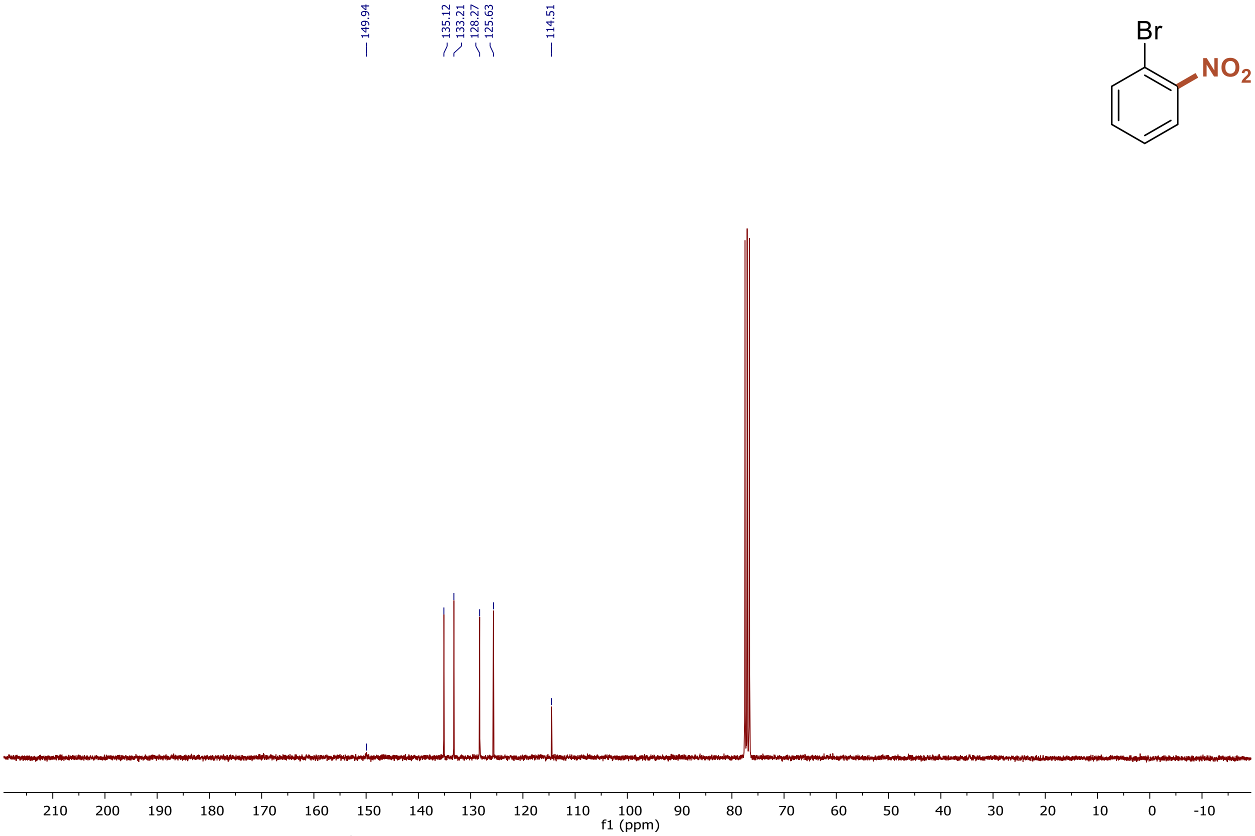

Supplementary Figure 47. ^{13}C NMR spectra for **9B**.

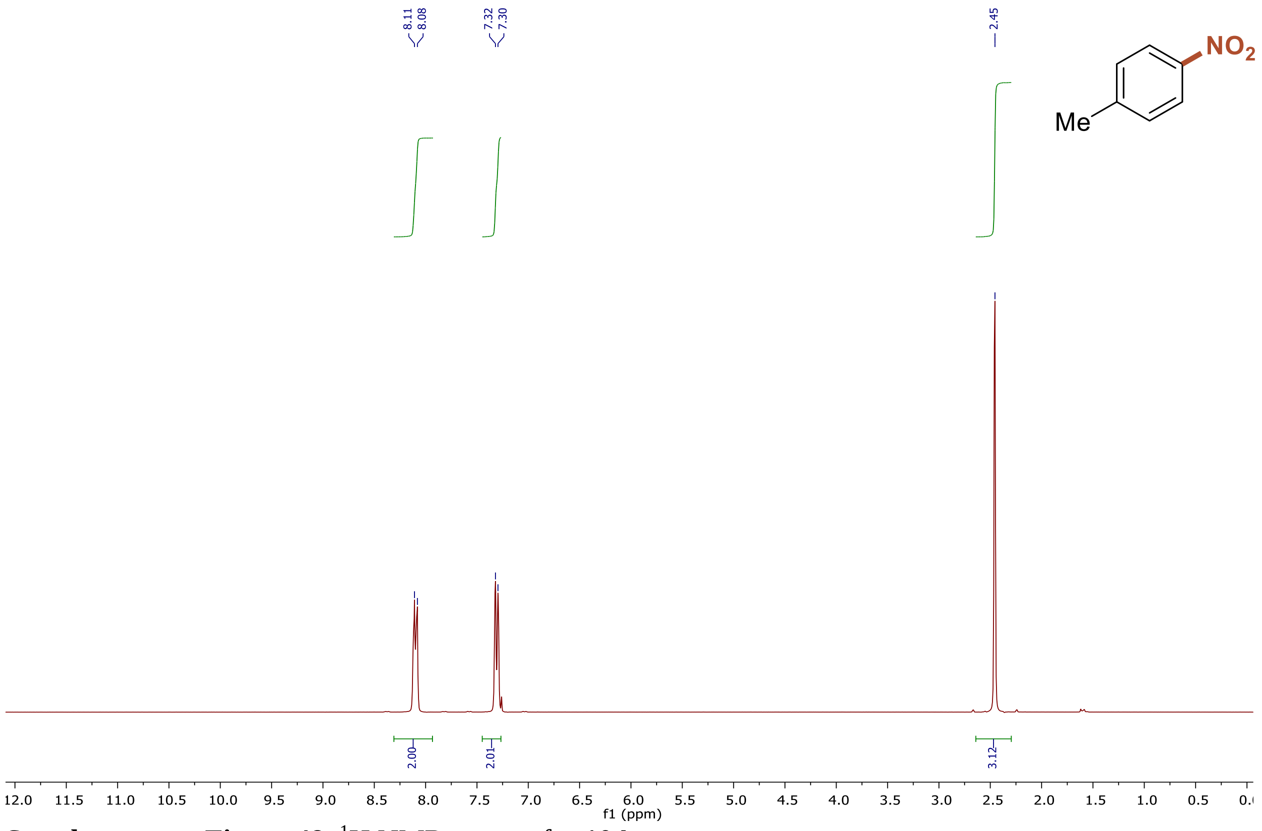

Supplementary Figure 48. ^{1}H NMR spectra for **10A**.

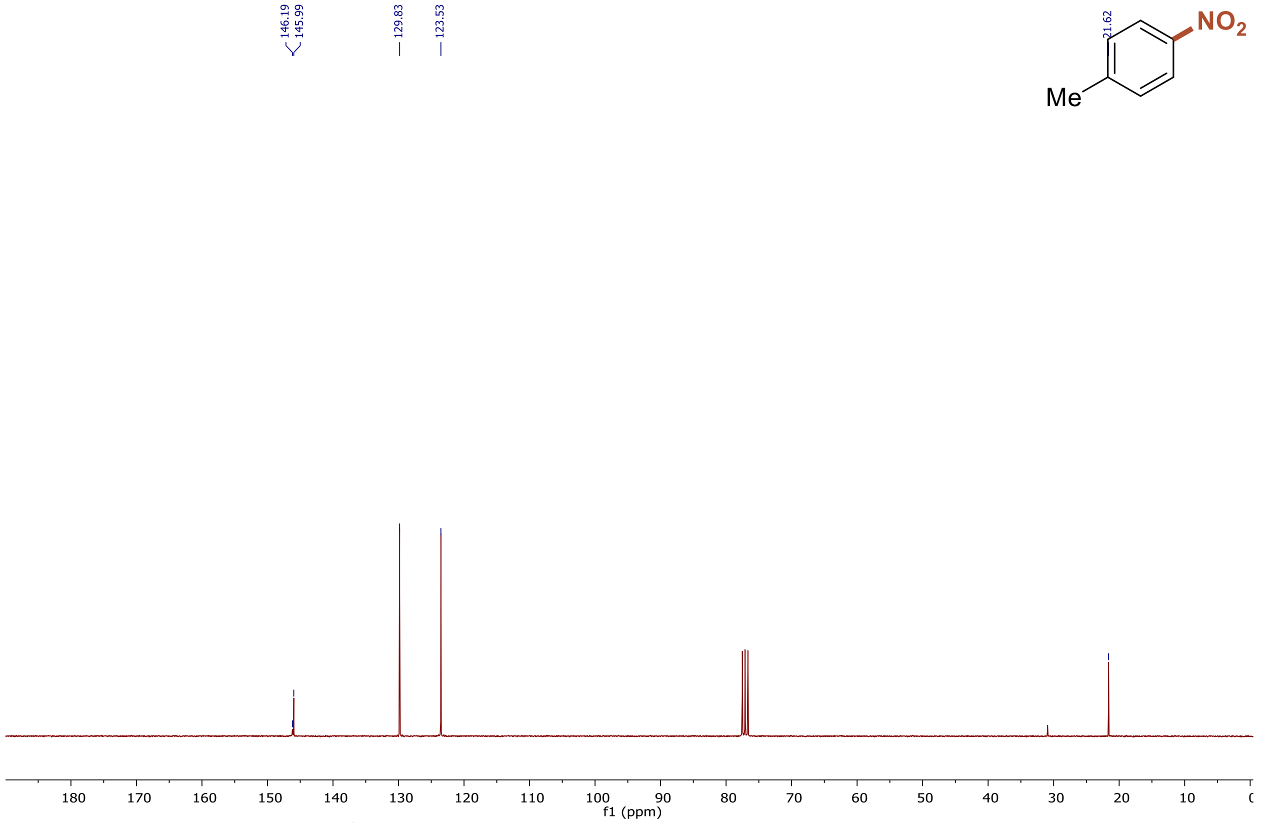

Supplementary Figure 49. ^{13}C NMR spectra for **10A**.

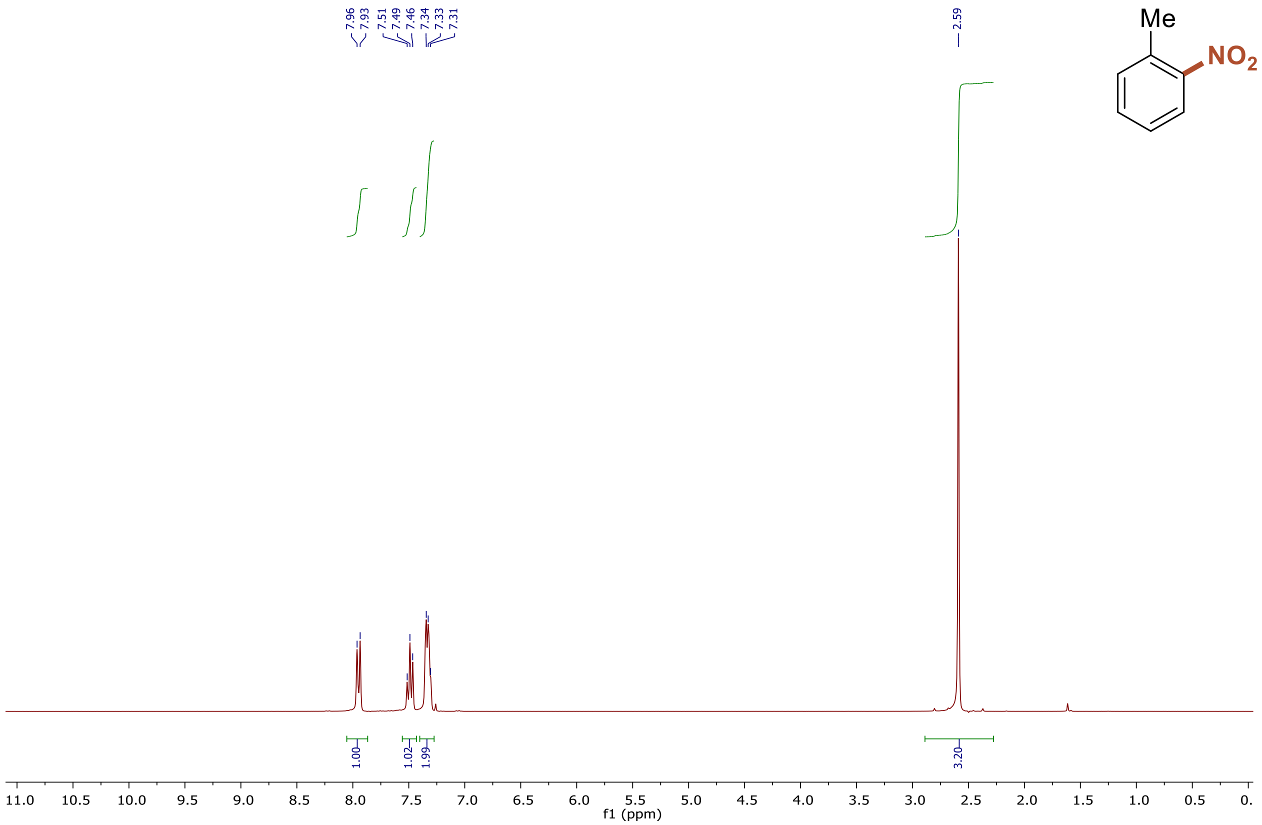

Supplementary Figure 50. ^{1}H NMR spectra for **10B**.

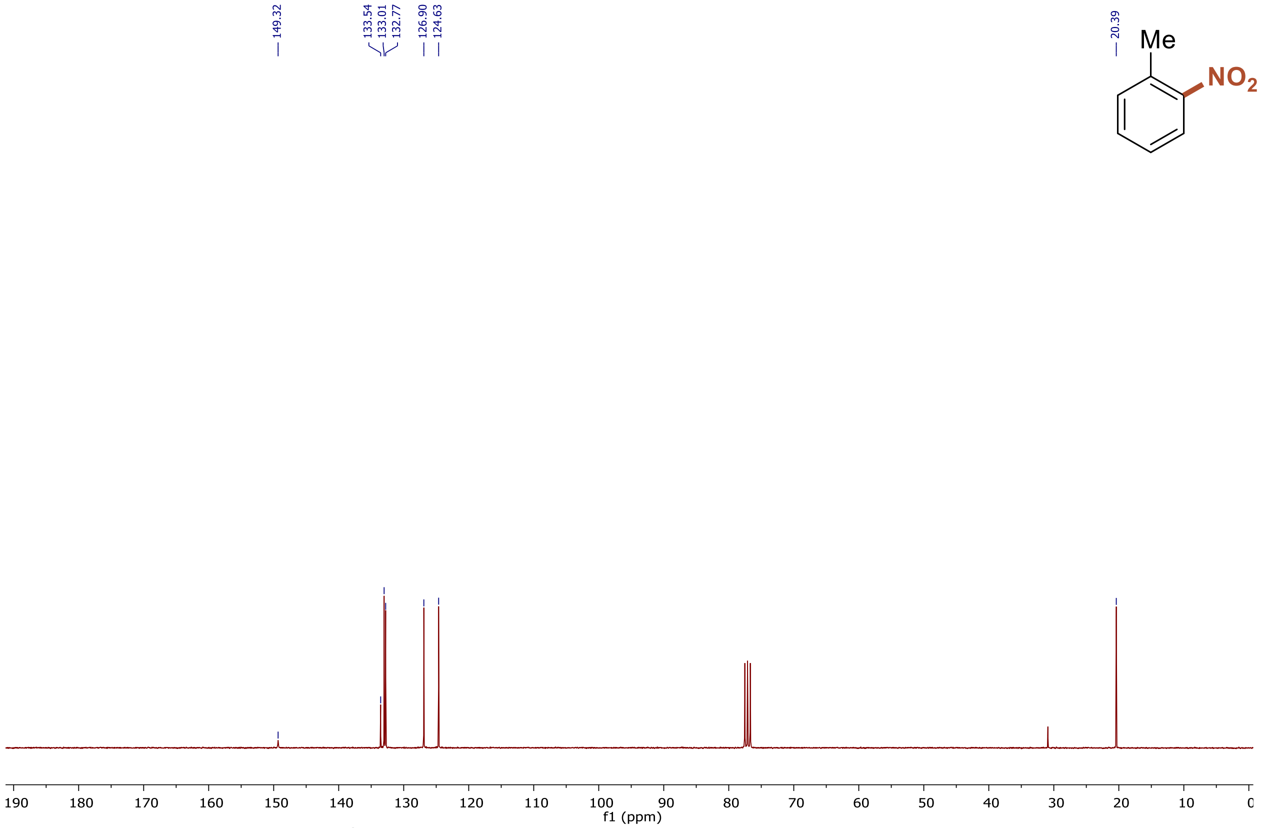

Supplementary Figure 51. ^{13}C NMR spectra for **10B**.

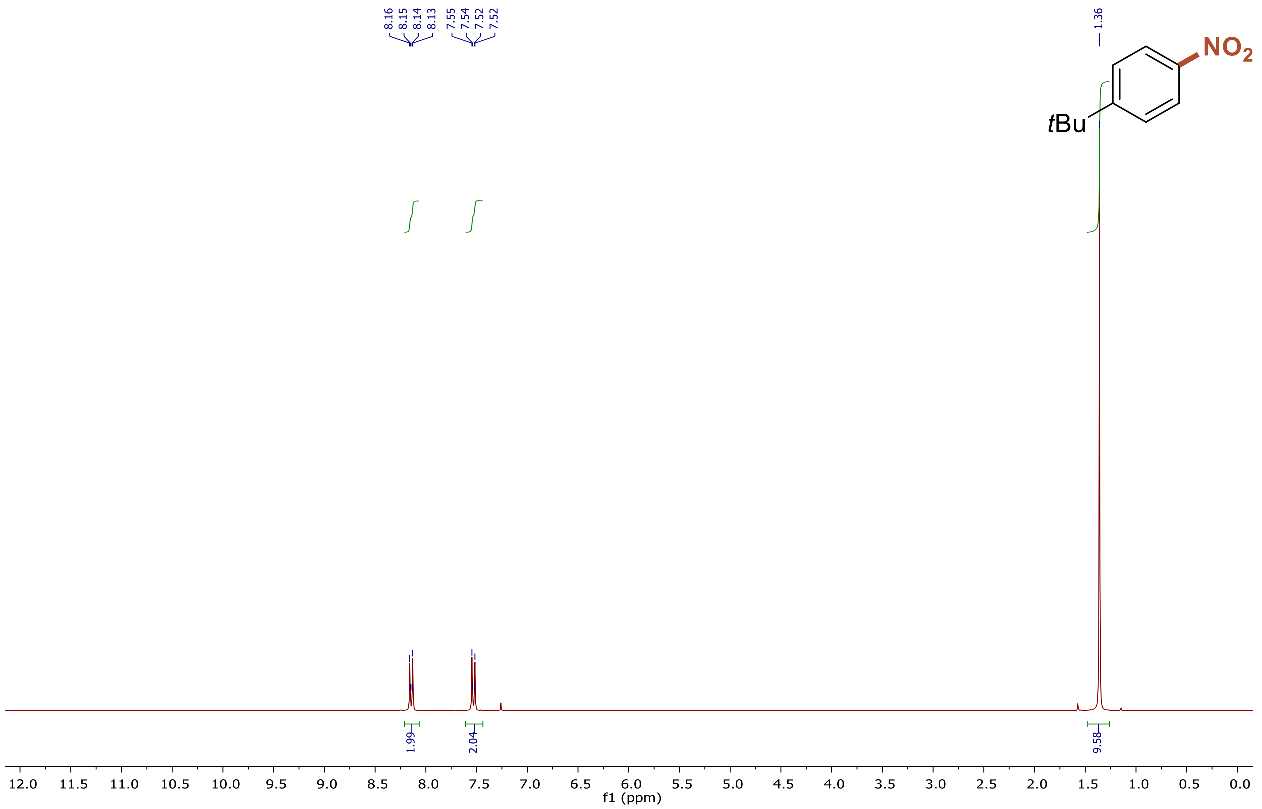

Supplementary Figure 52. ^{1}H NMR spectra for **11A**.

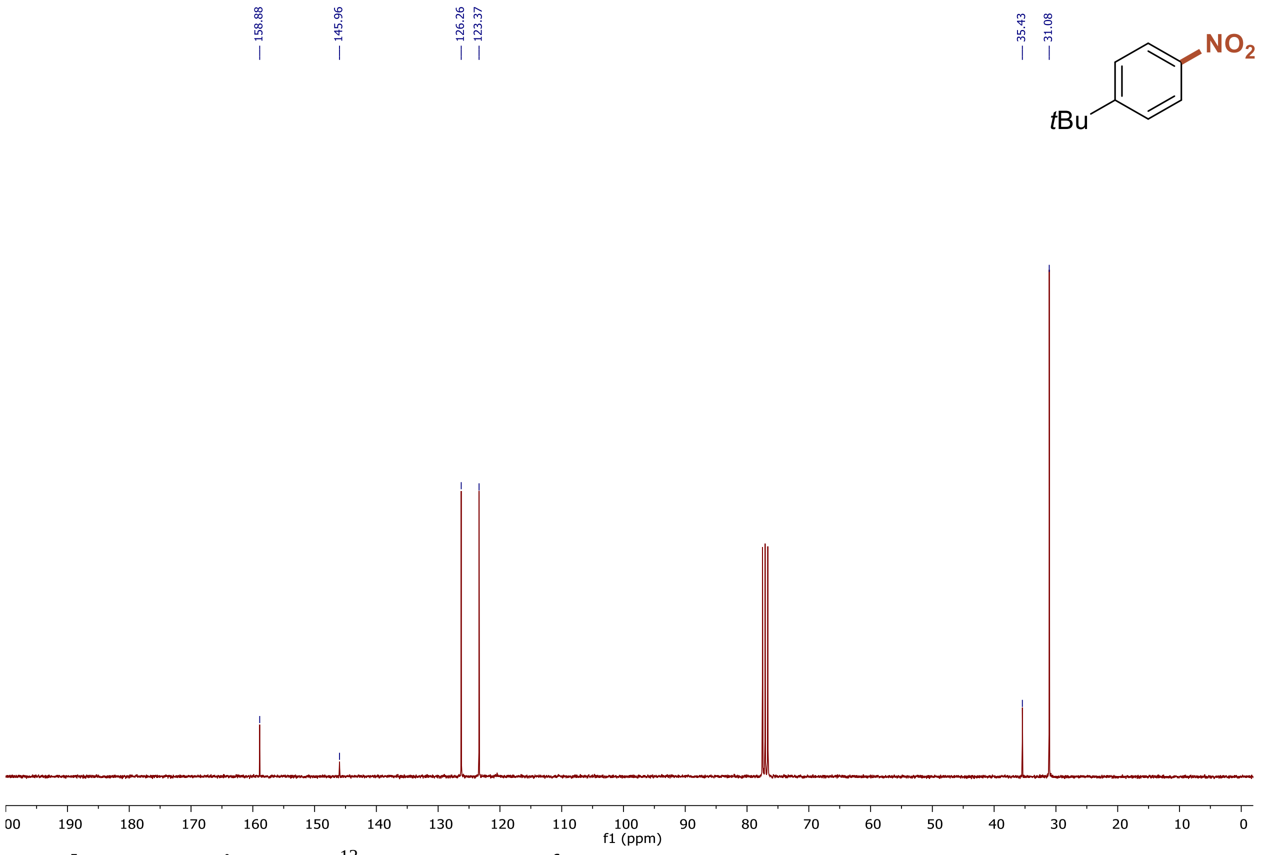

Supplementary Figure 53. ^{13}C NMR spectra for **11A**.

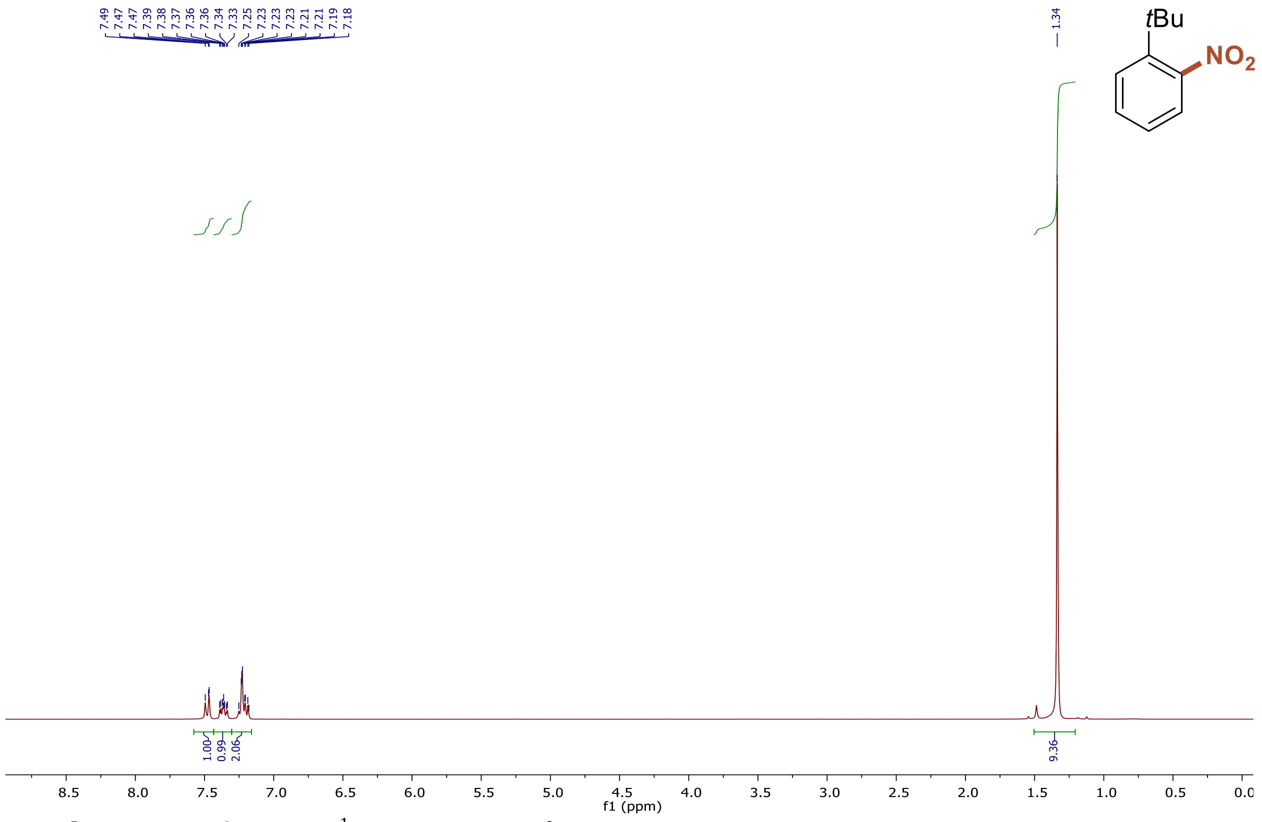

Supplementary Figure 54. ^{1}H NMR spectra for **11B**.

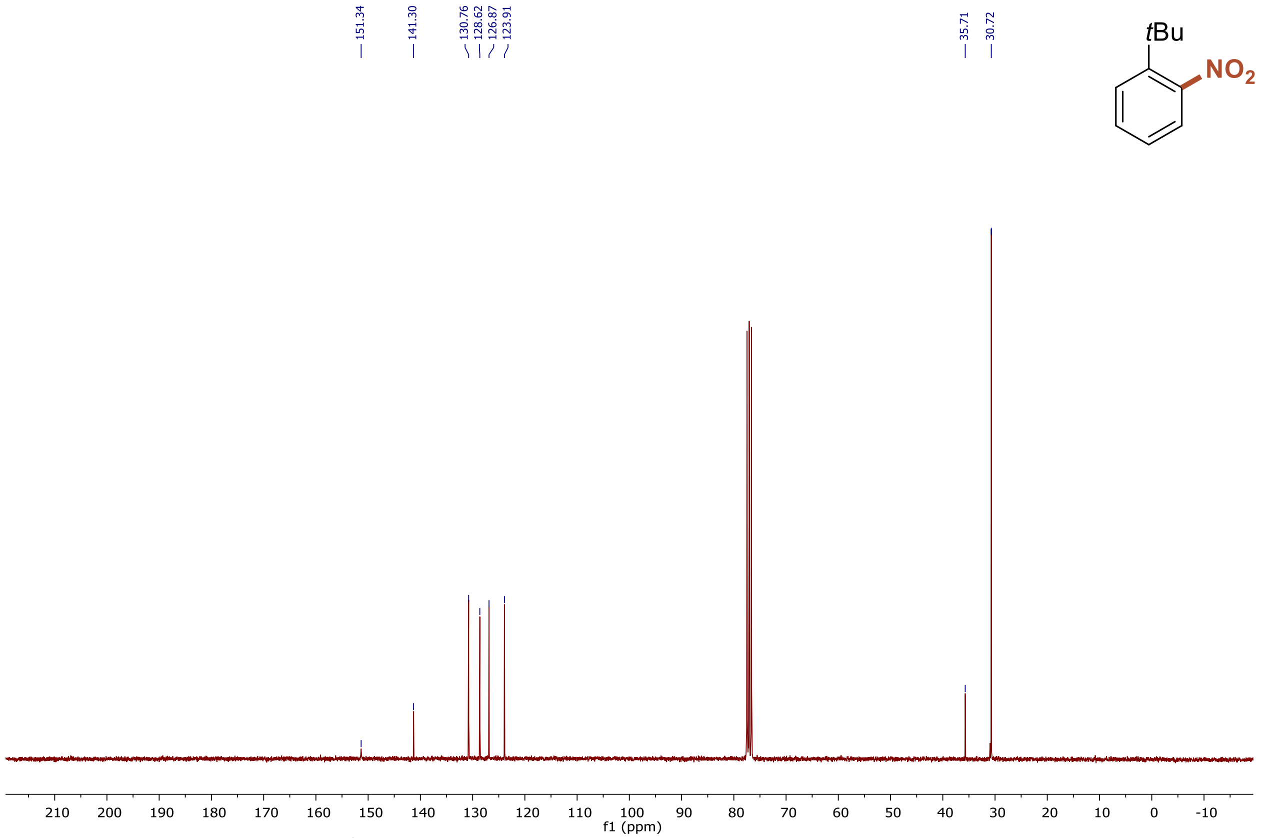

Supplementary Figure 55. ^{13}C NMR spectra for **11B**.

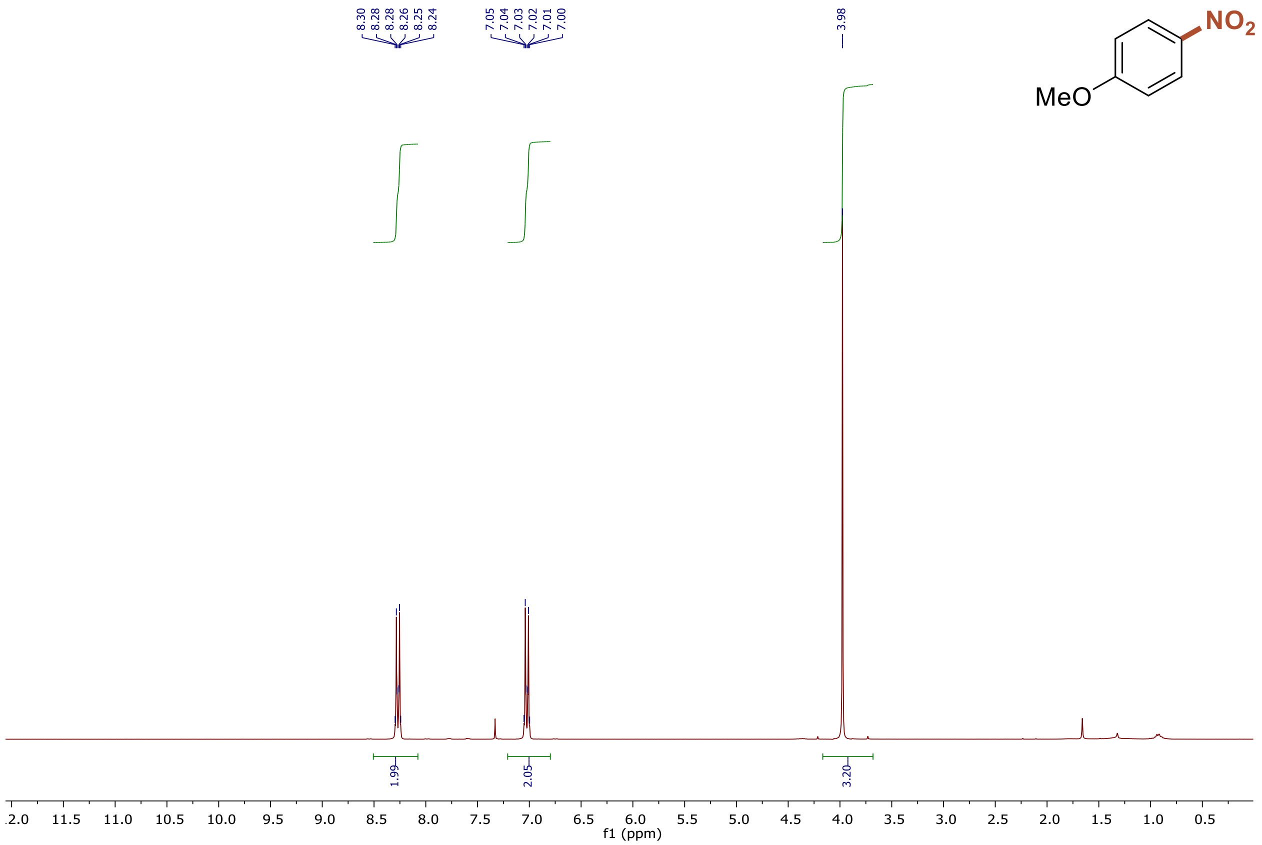

Supplementary Figure 56. ^{1}H NMR spectra for **12A**.

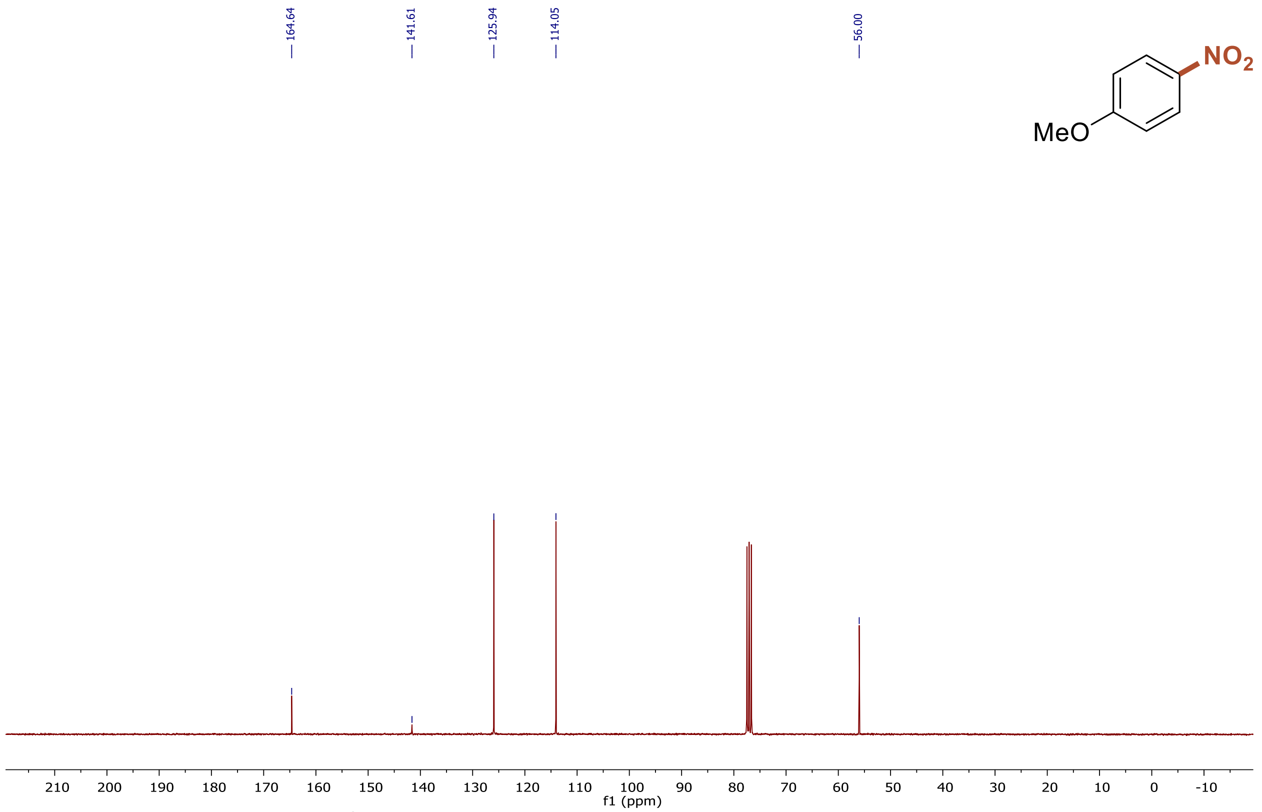

Supplementary Figure 57. ^{13}C NMR spectra for **12A**.

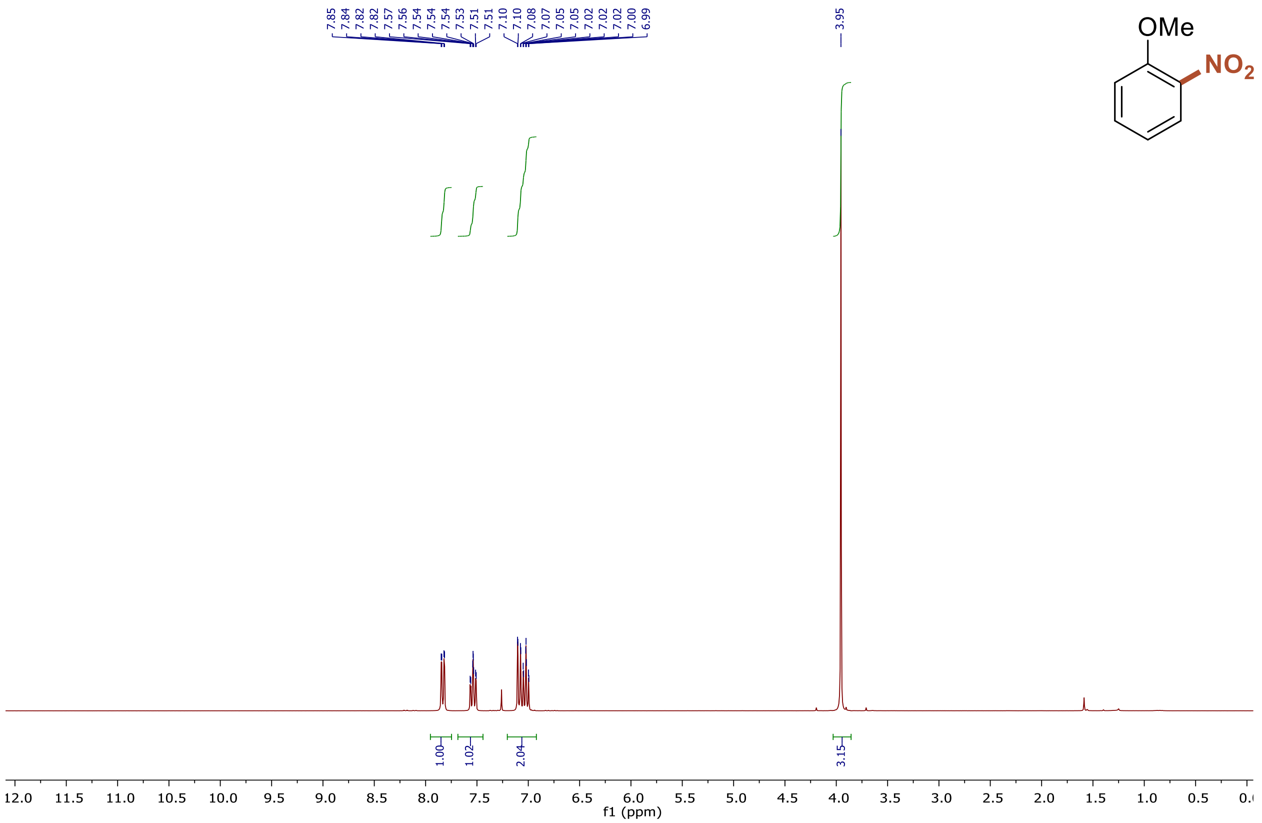

Supplementary Figure 58. ^{1}H NMR spectra for **12B**.

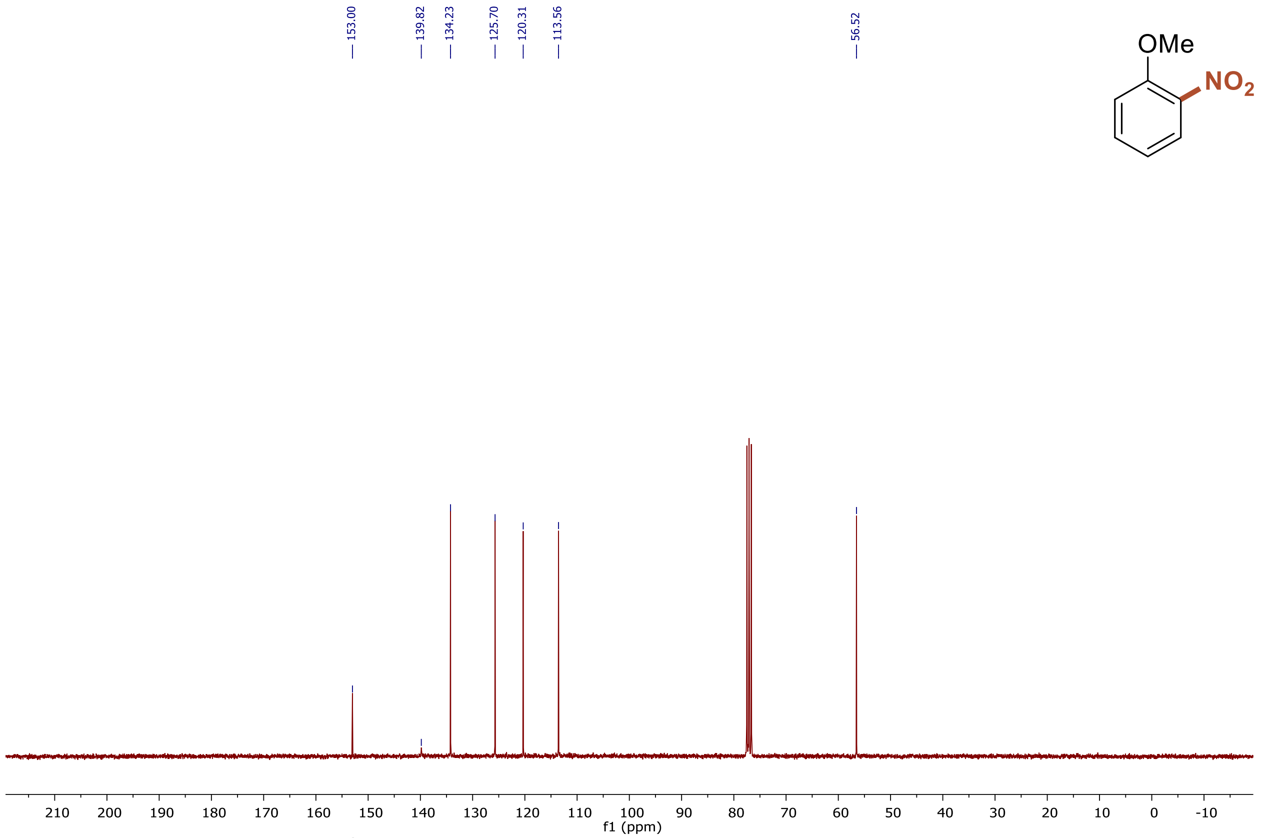

Supplementary Figure 59. ^{13}C NMR spectra for **12B**.

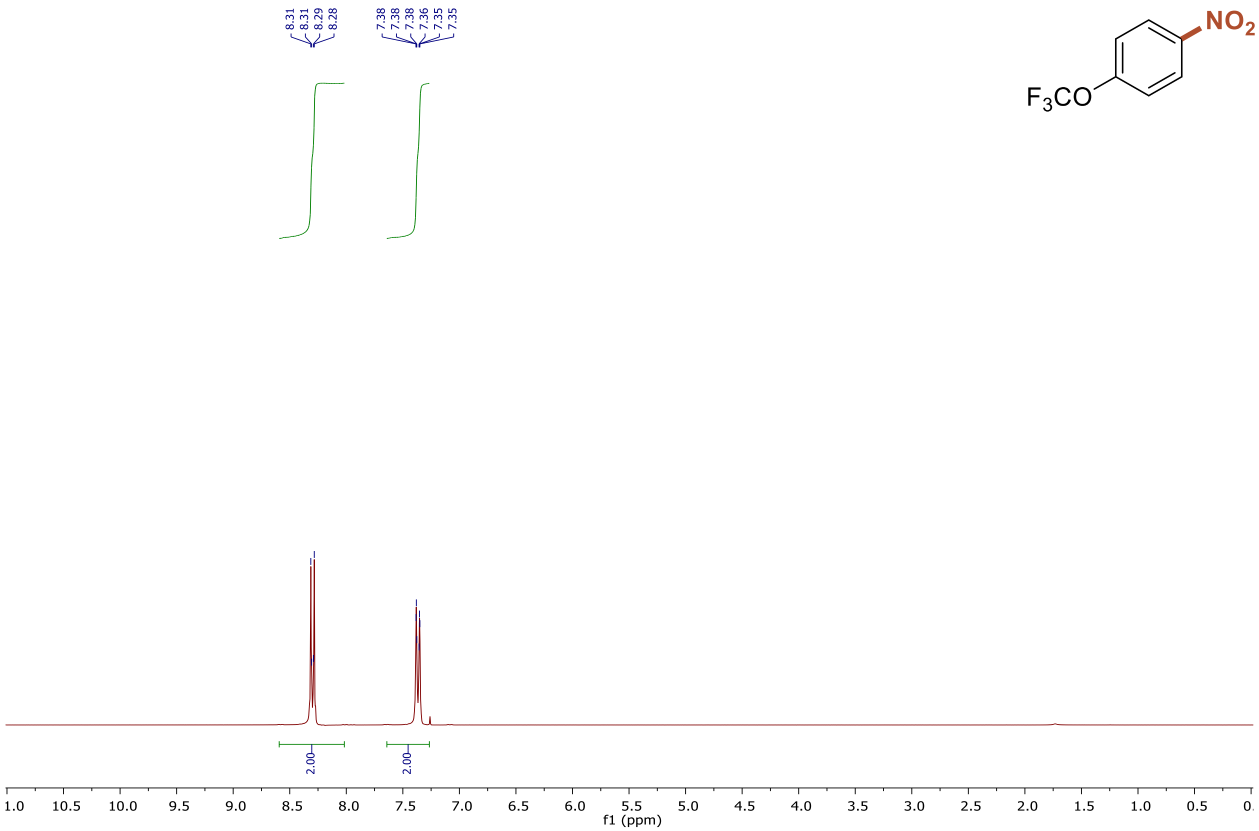

Supplementary Figure 60. ^{1}H NMR spectra for **13A**.

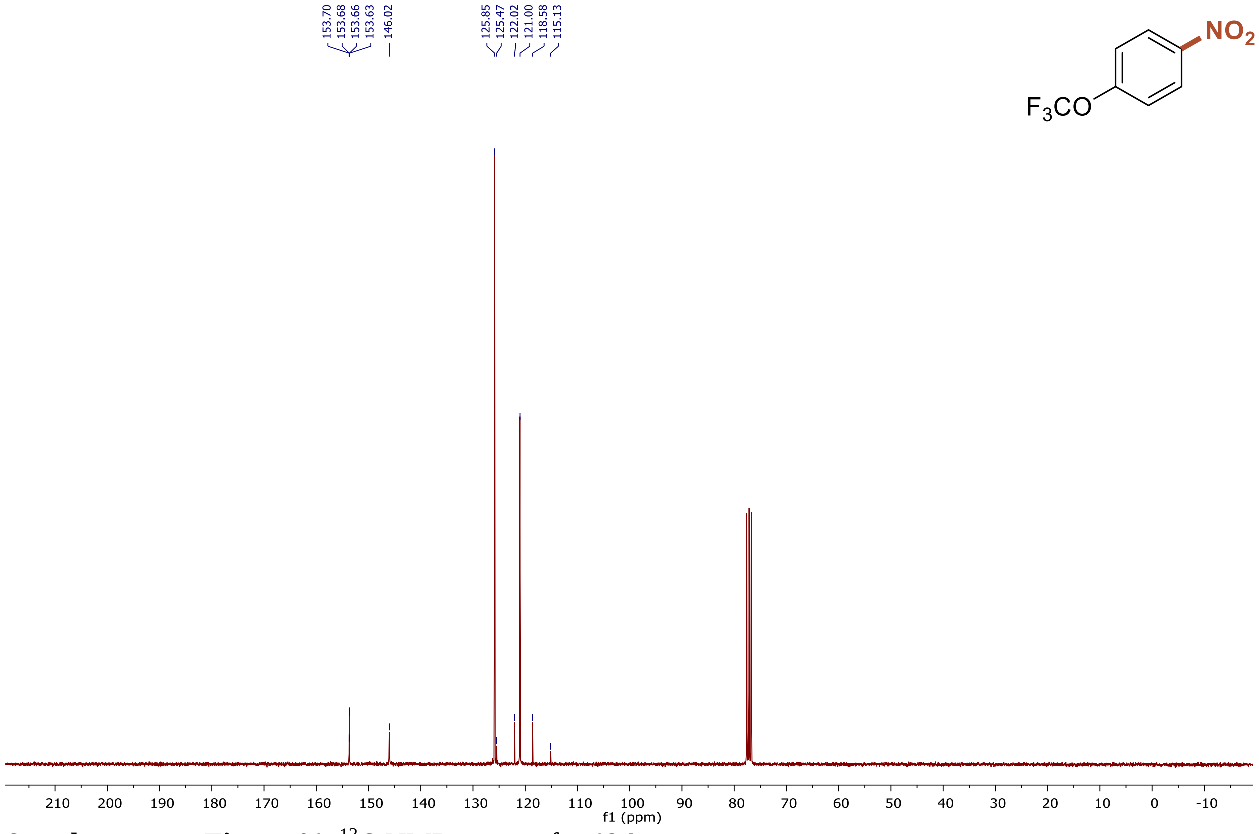

Supplementary Figure 61. ^{13}C NMR spectra for **13A**.

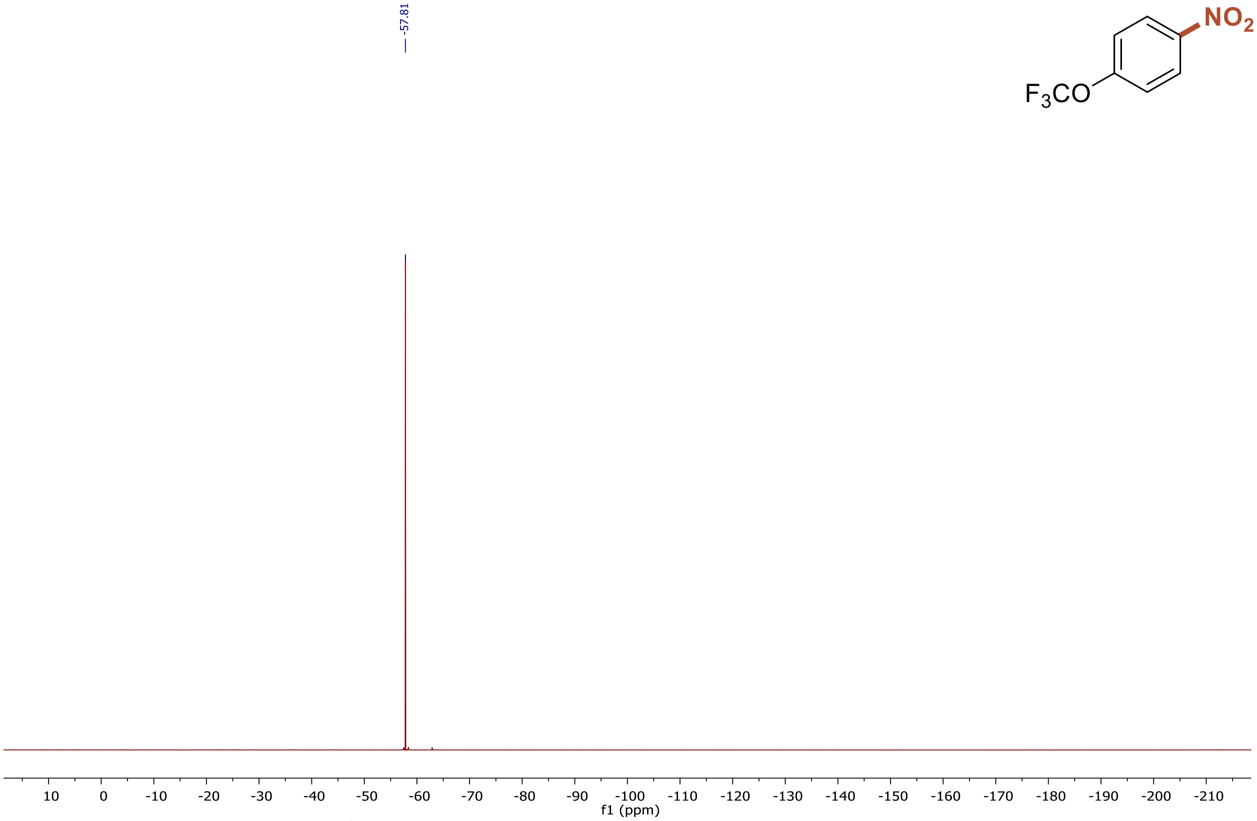

Supplementary Figure 62. ^{19}F NMR spectra for **13A**.

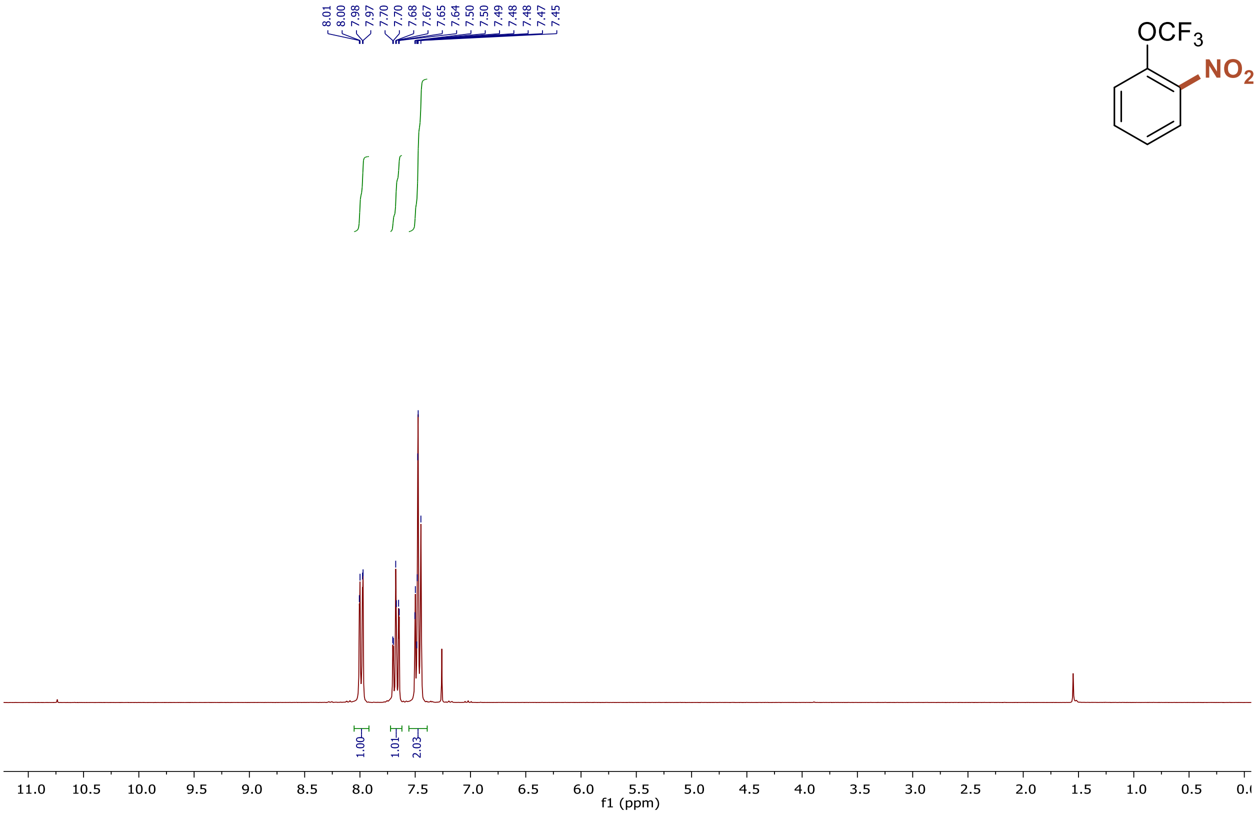

Supplementary Figure 63. ^{1}H NMR spectra for **13B**.

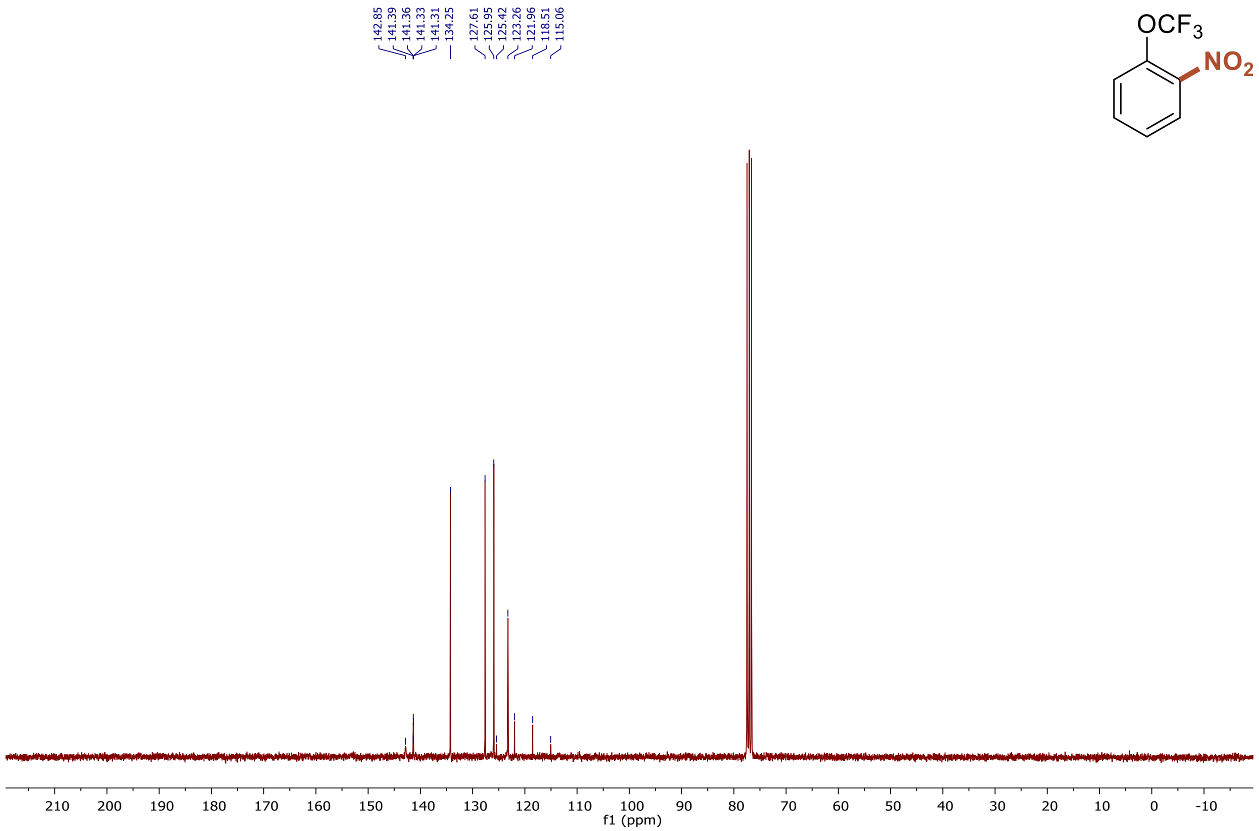

Supplementary Figure 64. ^{13}C NMR spectra for **13B**.

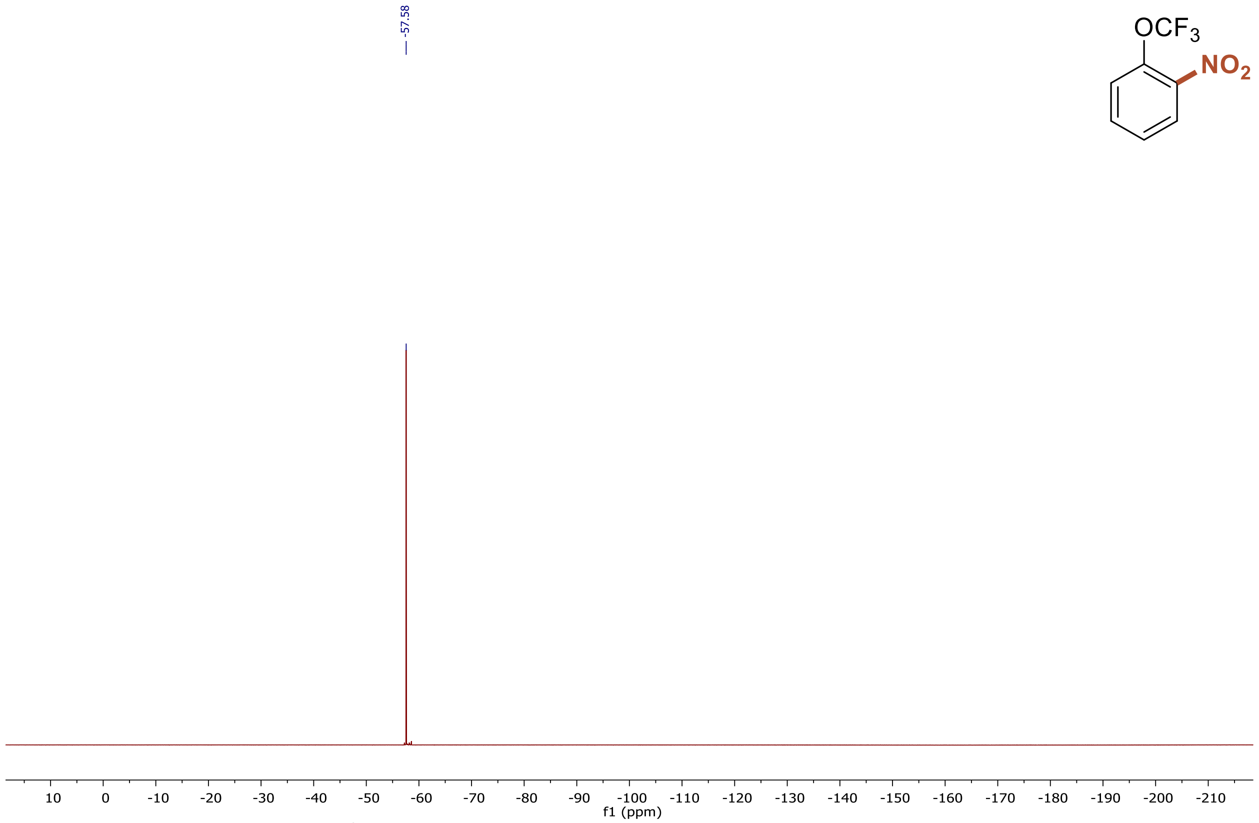

Supplementary Figure 65. ^{19}F NMR spectra for **13B**.

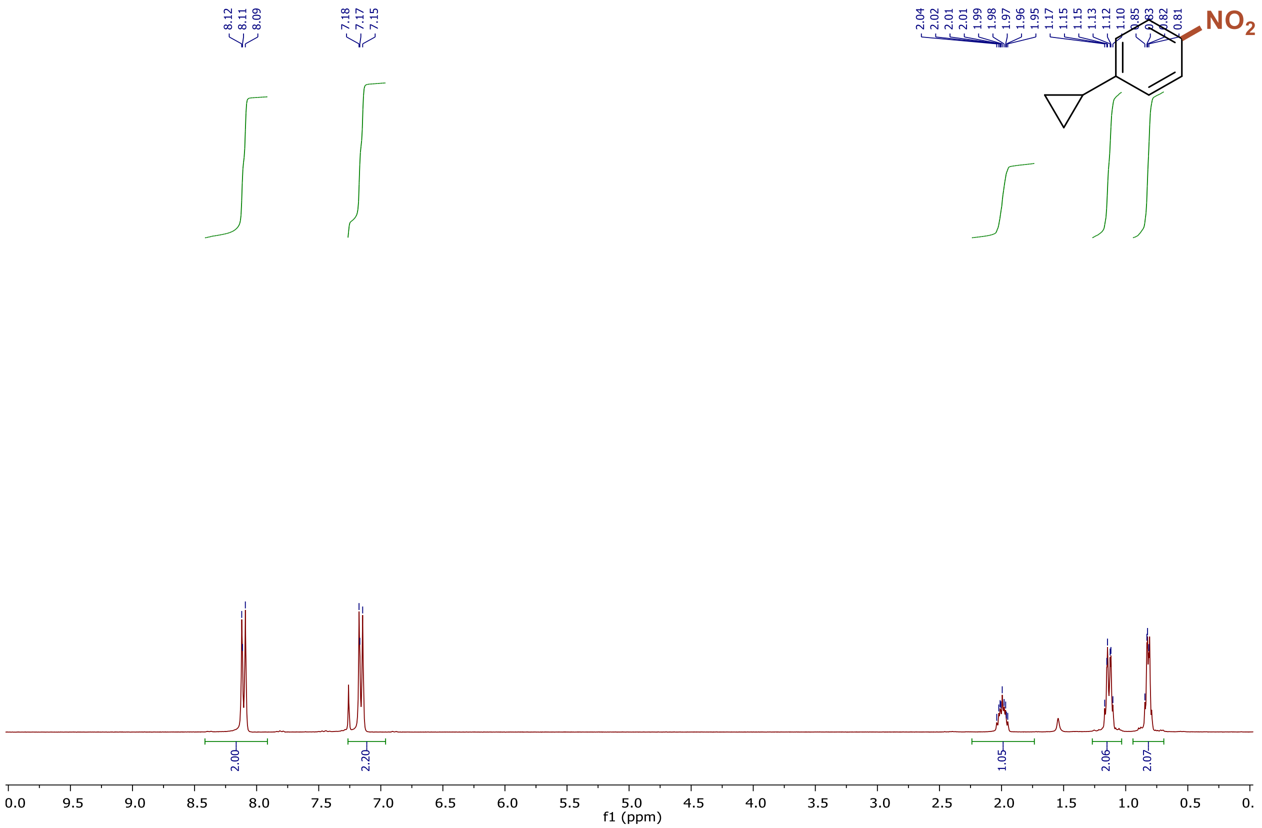

Supplementary Figure 66. ^{1}H NMR spectra for **14A**.

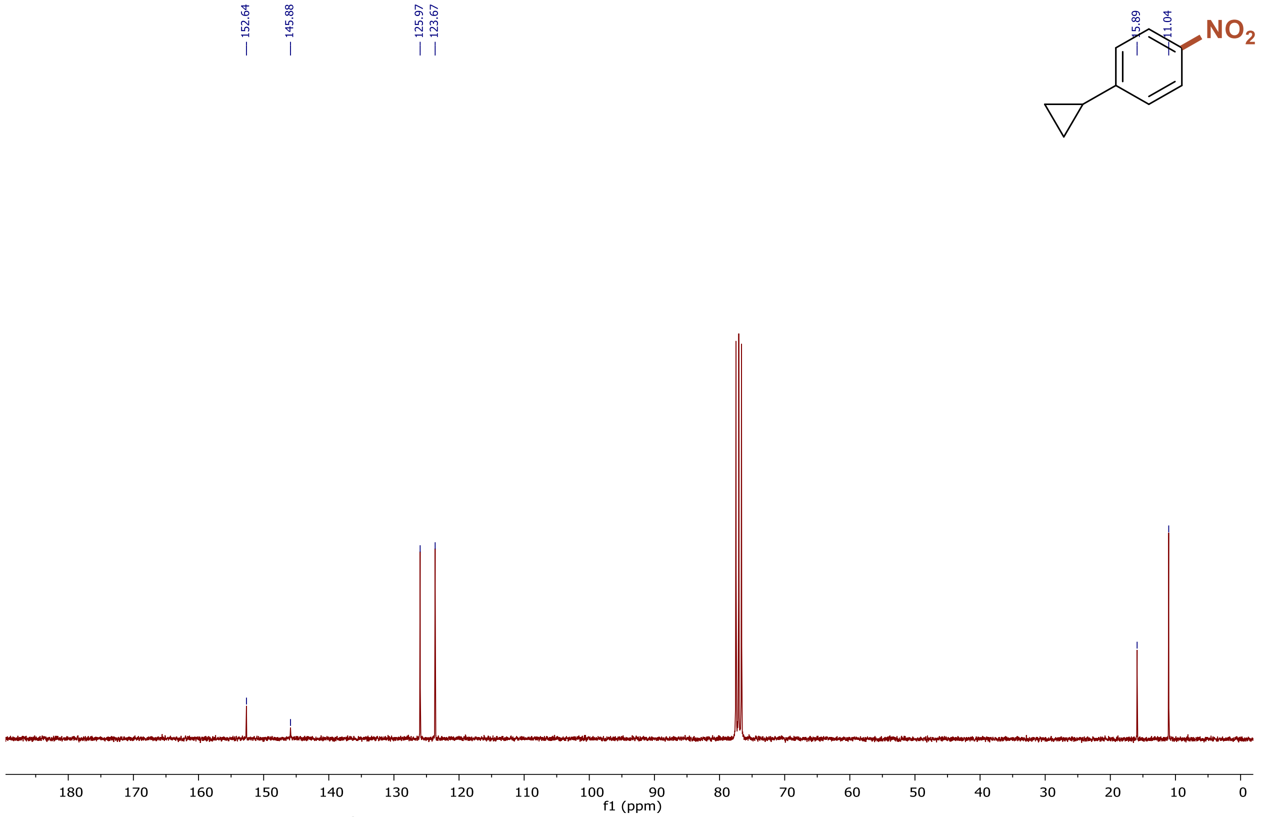

Supplementary Figure 67. ^{13}C NMR spectra for **14A**.

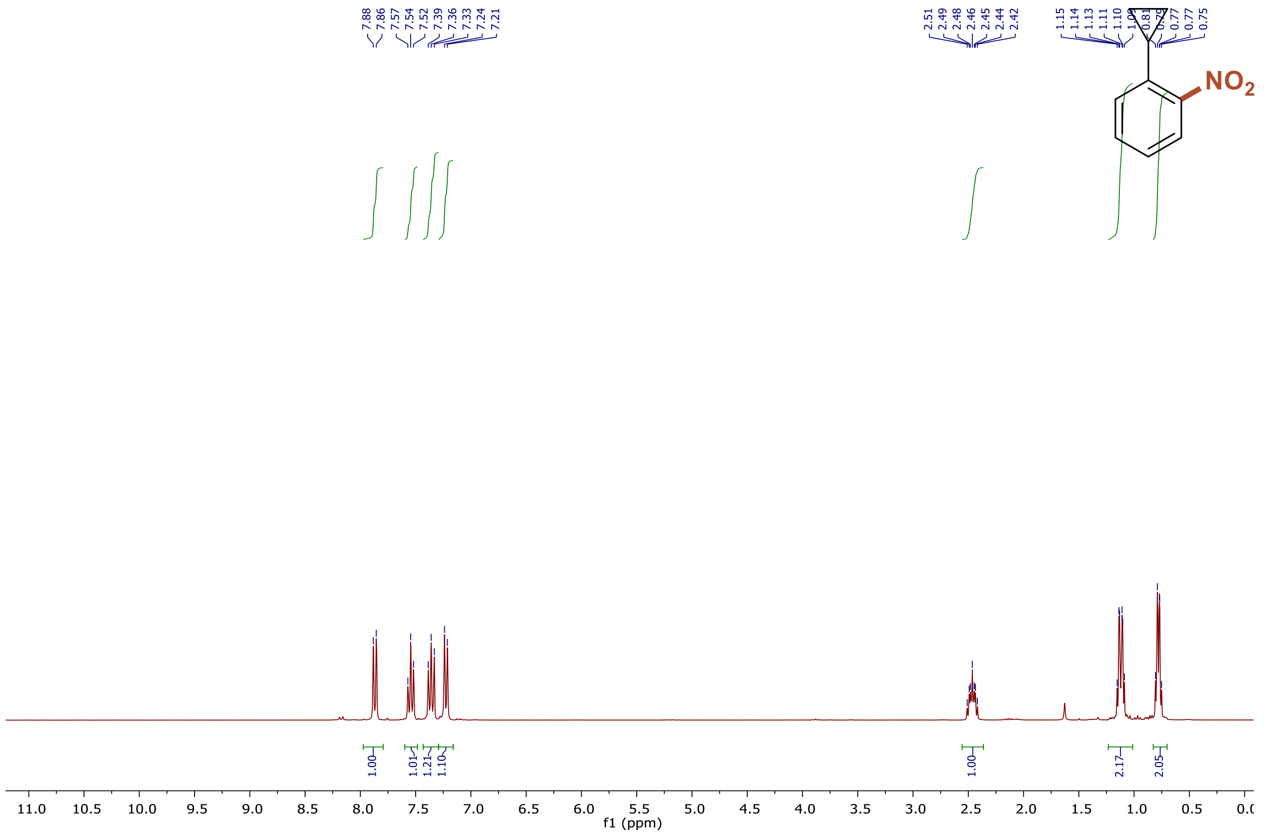

Supplementary Figure 68. ^{1}H NMR spectra for **14B**.

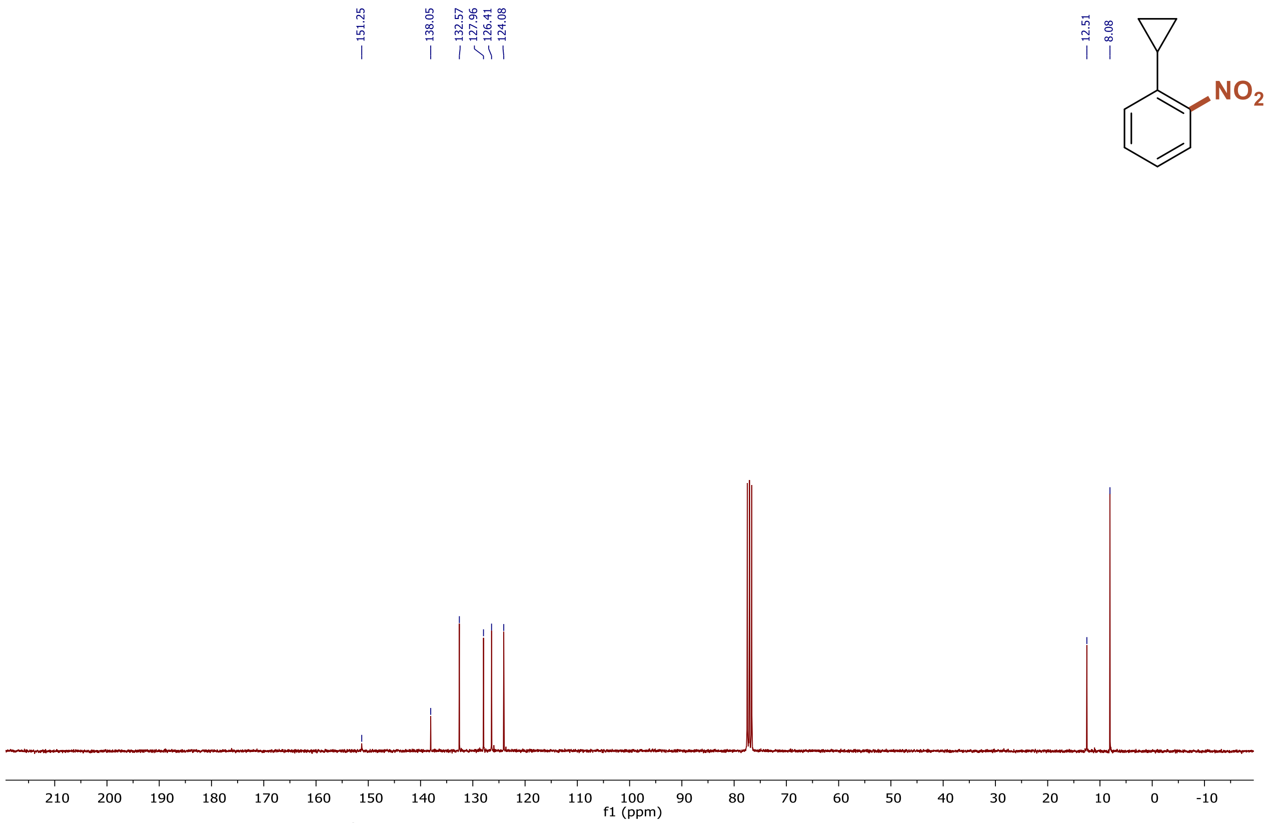

Supplementary Figure 69. ^{13}C NMR spectra for **14B**.

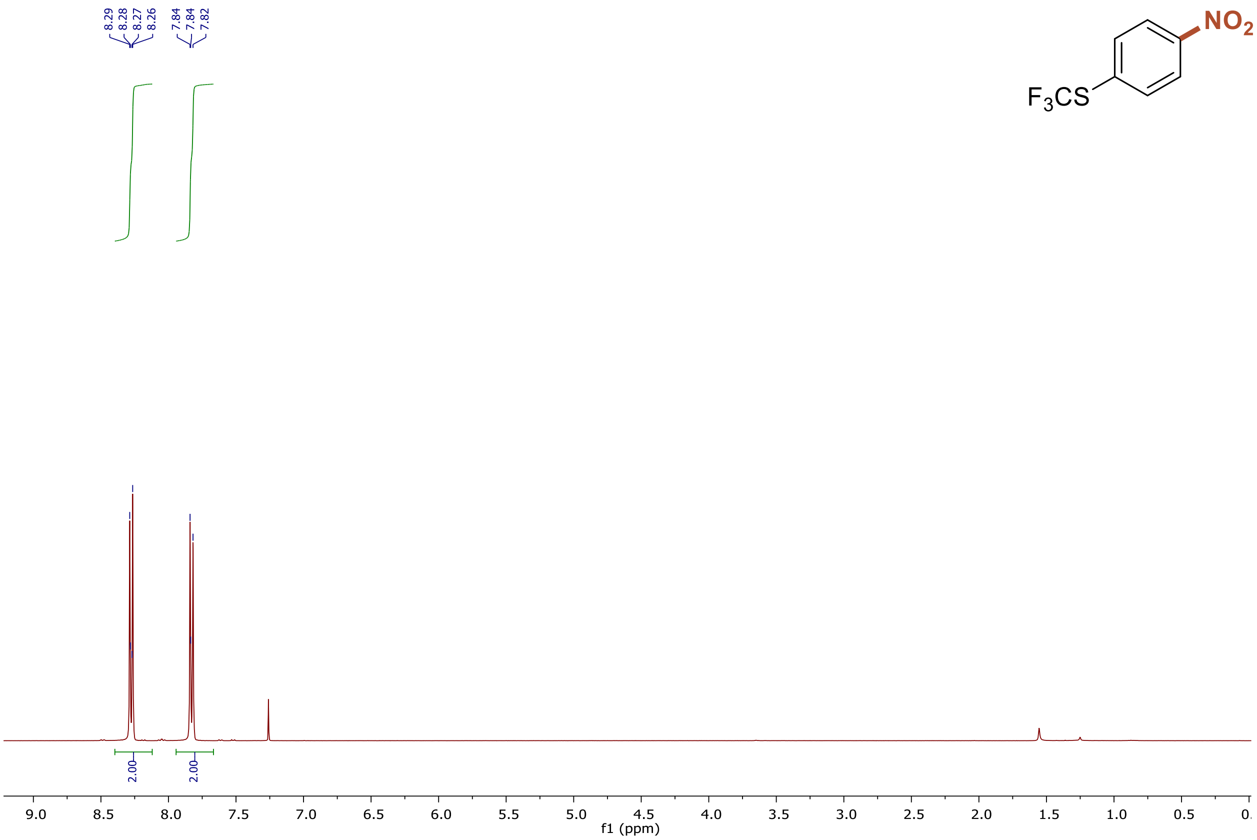

Supplementary Figure 70. ^{1}H NMR spectra for **15A**.

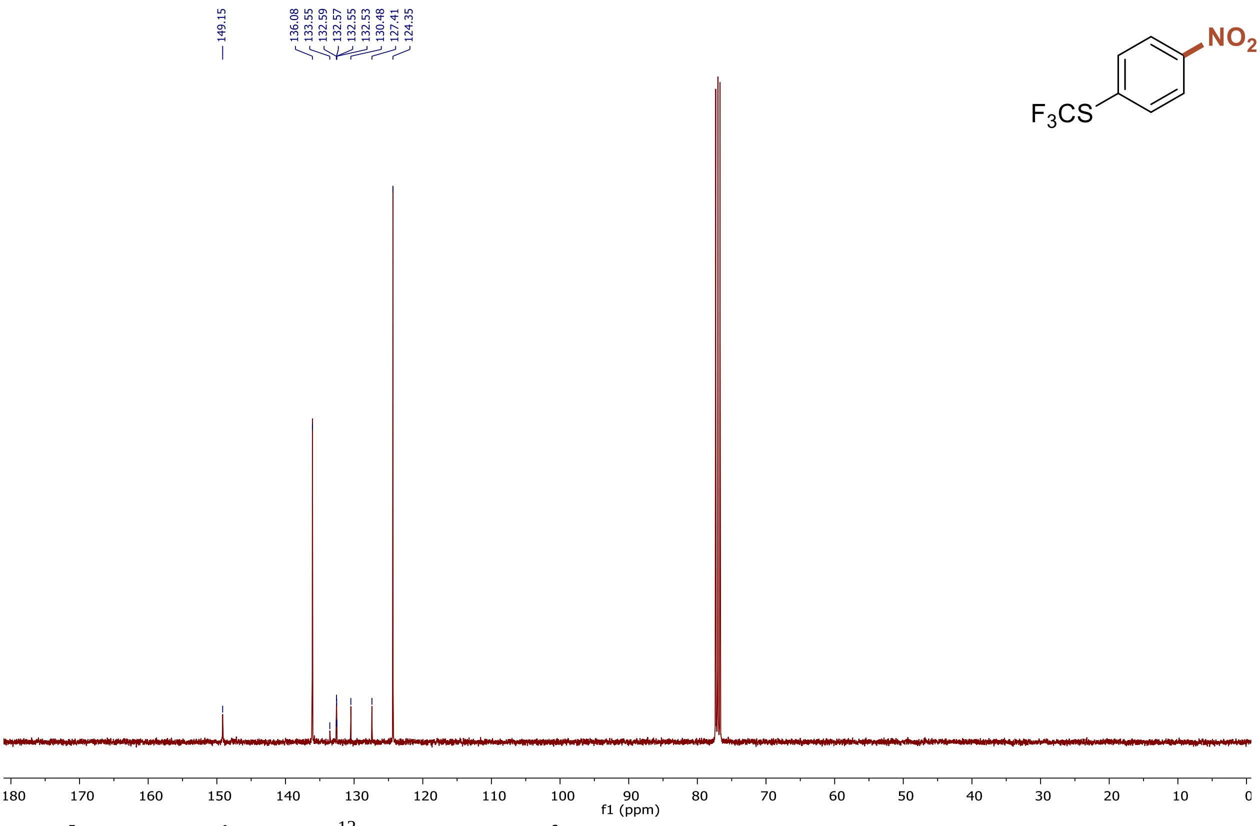

Supplementary Figure 71. ^{13}C NMR spectra for**15A**.

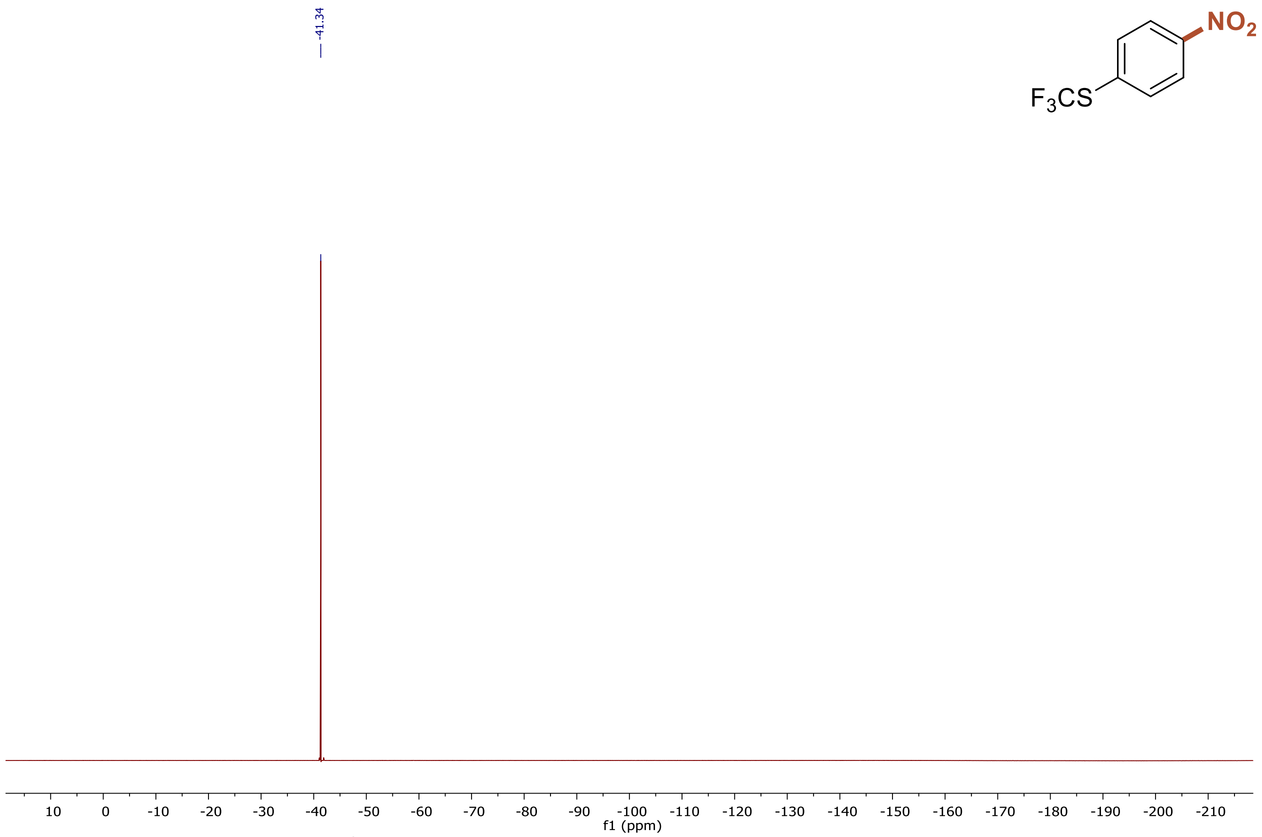

Supplementary Figure 72. ^{19}F NMR spectra for **15A**.

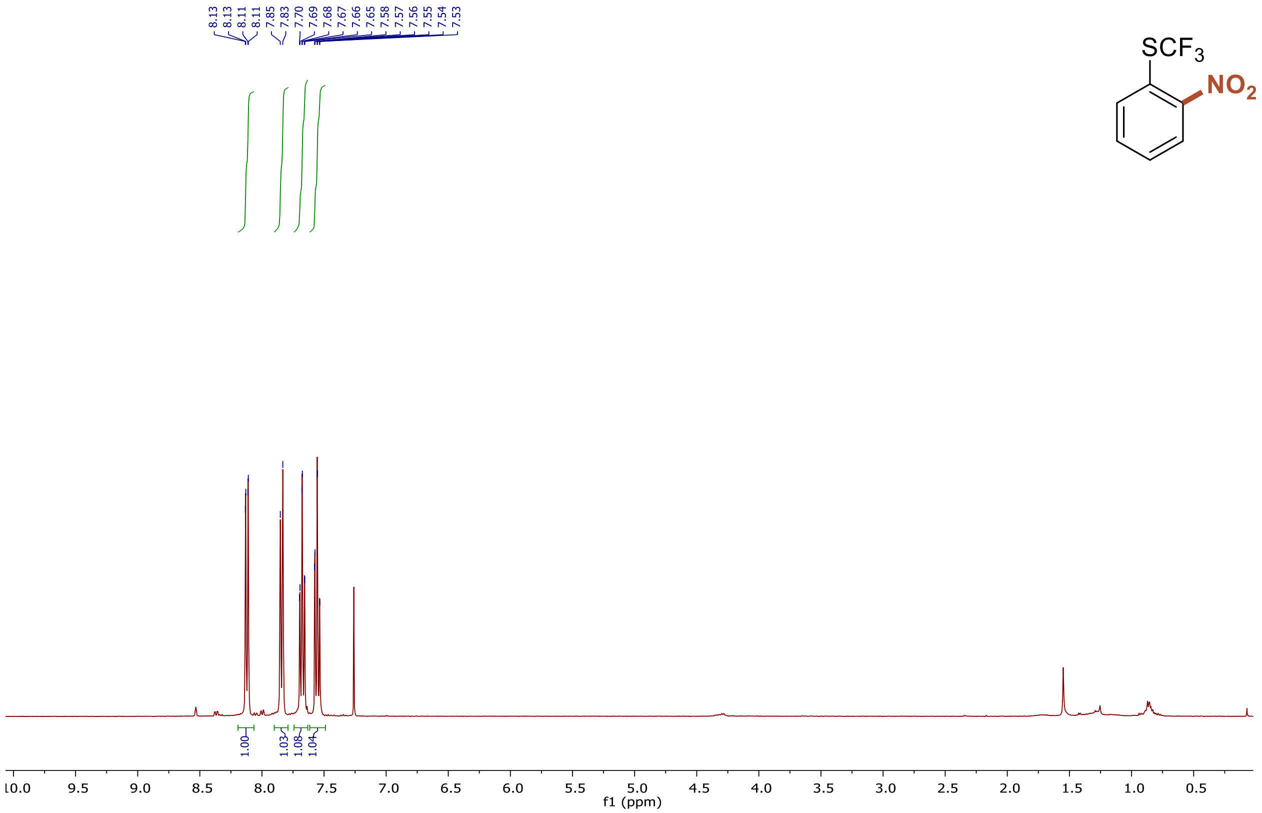

Supplementary Figure 73. ^{1}H NMR spectra for **15B**.

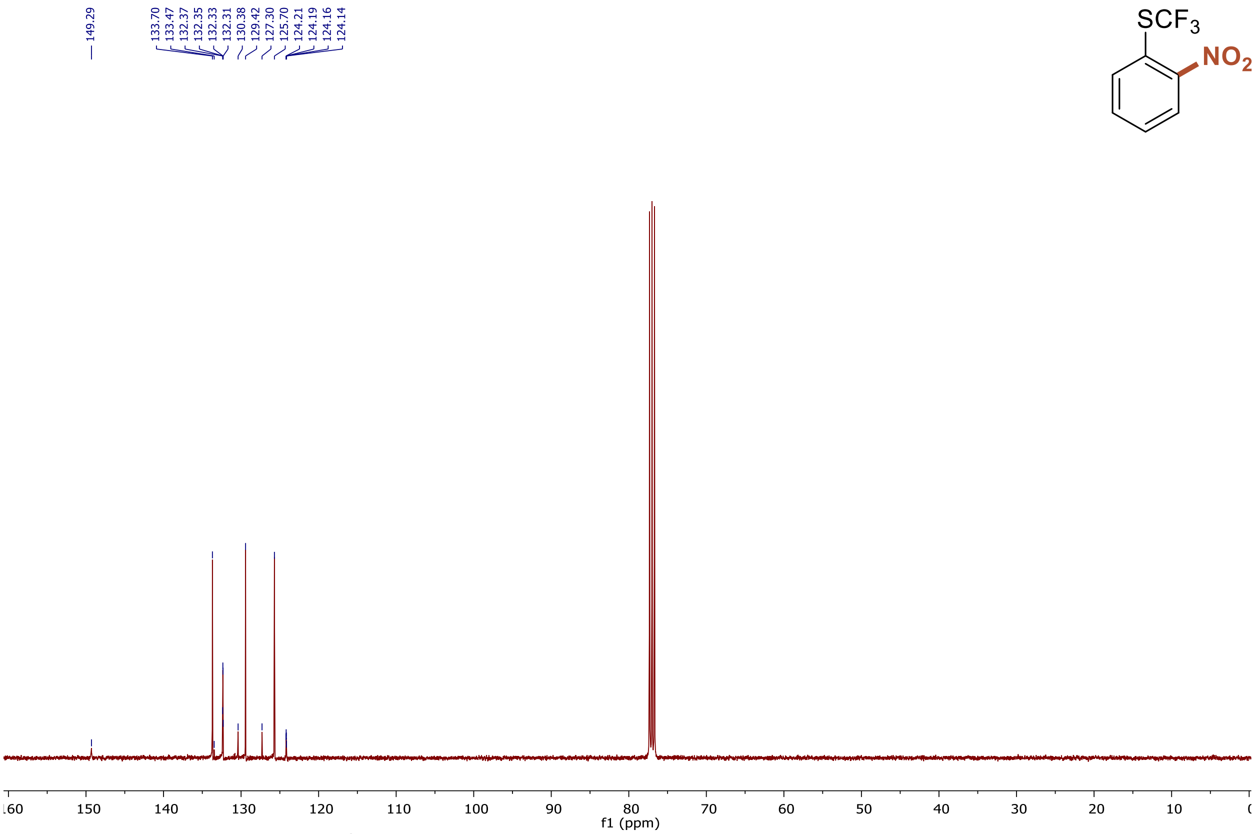

Supplementary Figure 74. ^{13}C NMR spectra for **15B**.

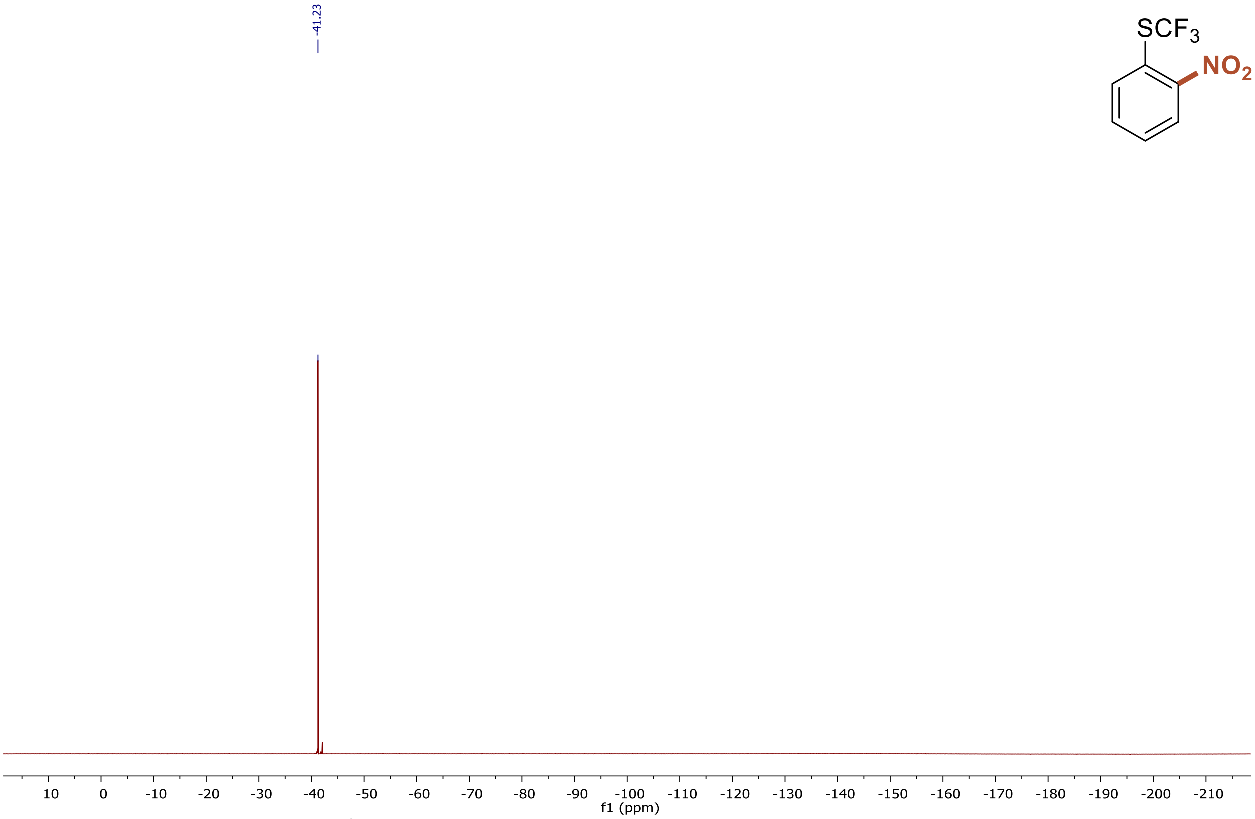

Supplementary Figure 75. ^{19}F NMR spectra for **15B**.

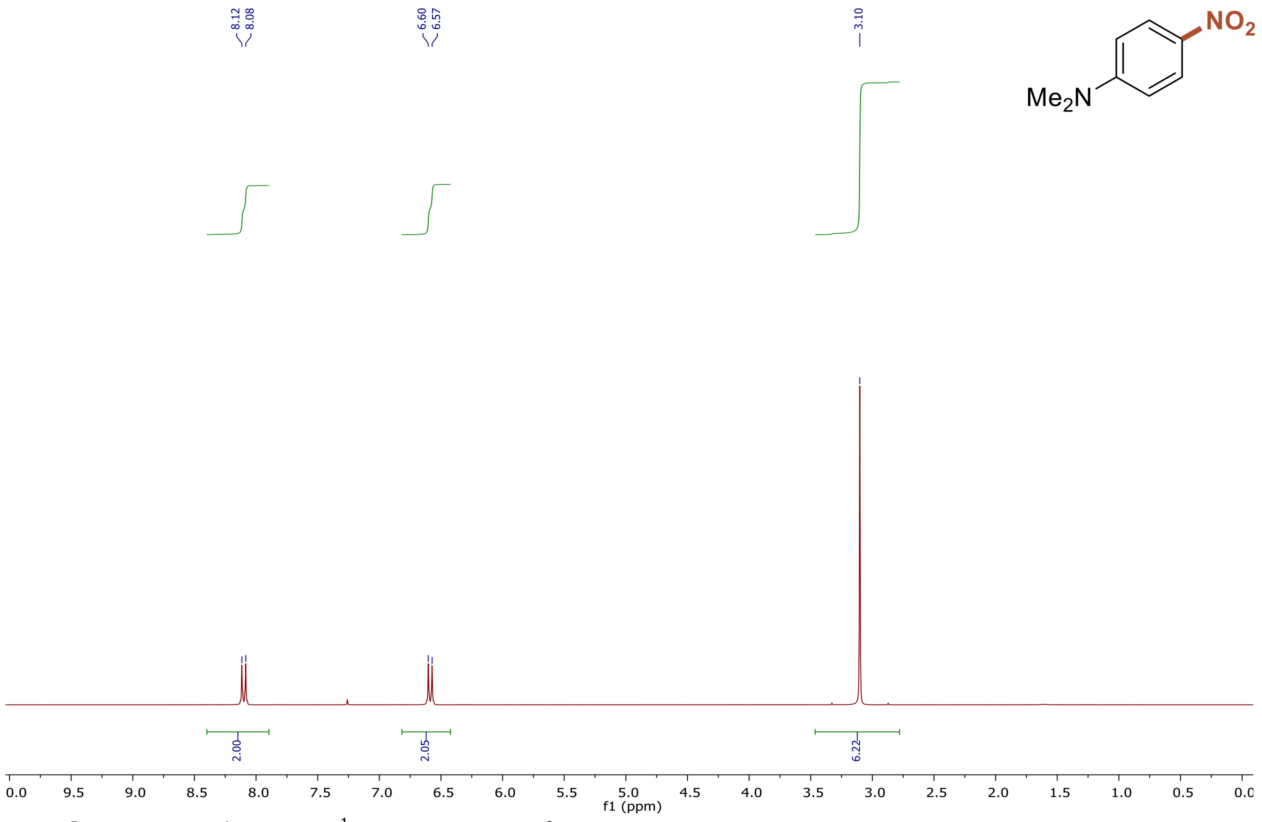

Supplementary Figure 76. ^{1}H NMR spectra for **16A**.

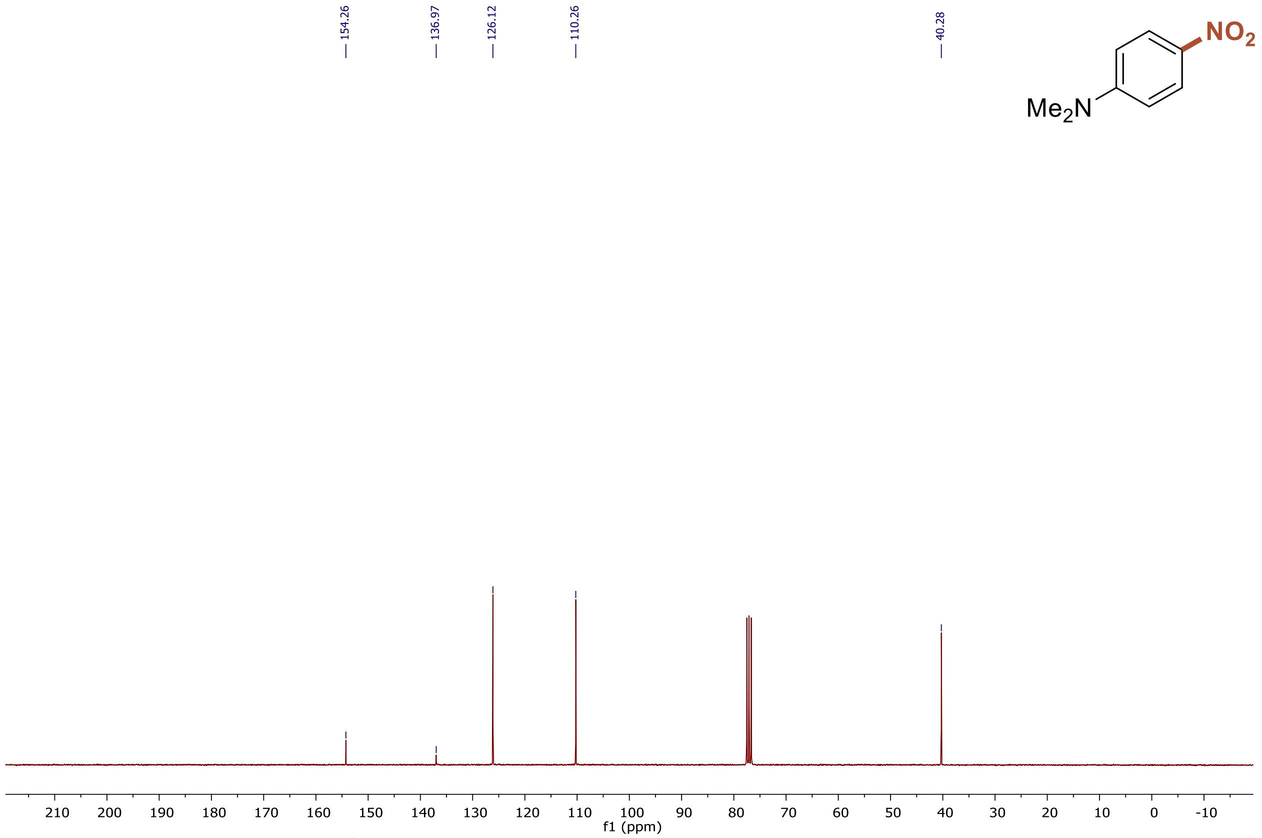

Supplementary Figure 77. ^{13}C NMR spectra for **16A**.

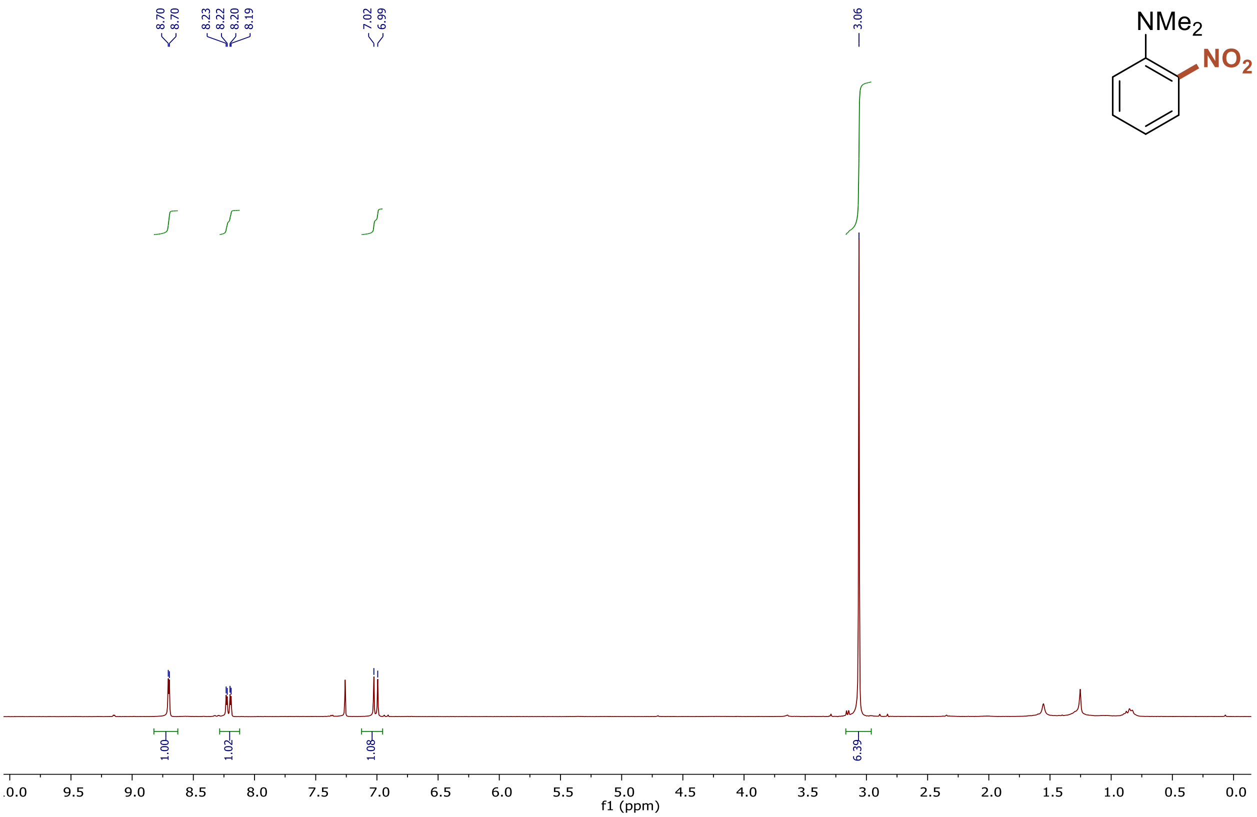

Supplementary Figure 78. ^{1}H NMR spectra for **16B**.

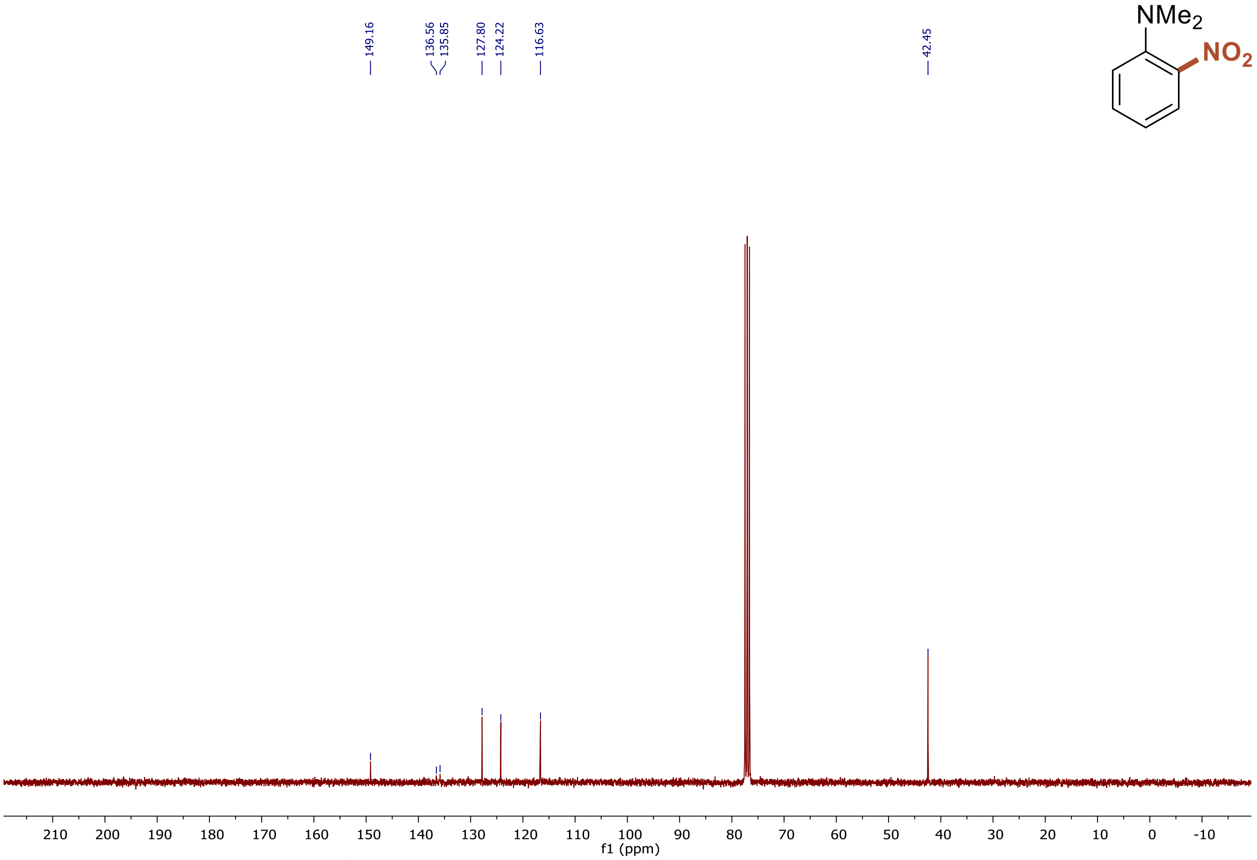

Supplementary Figure 79. ^{13}C NMR spectra for **16B**.

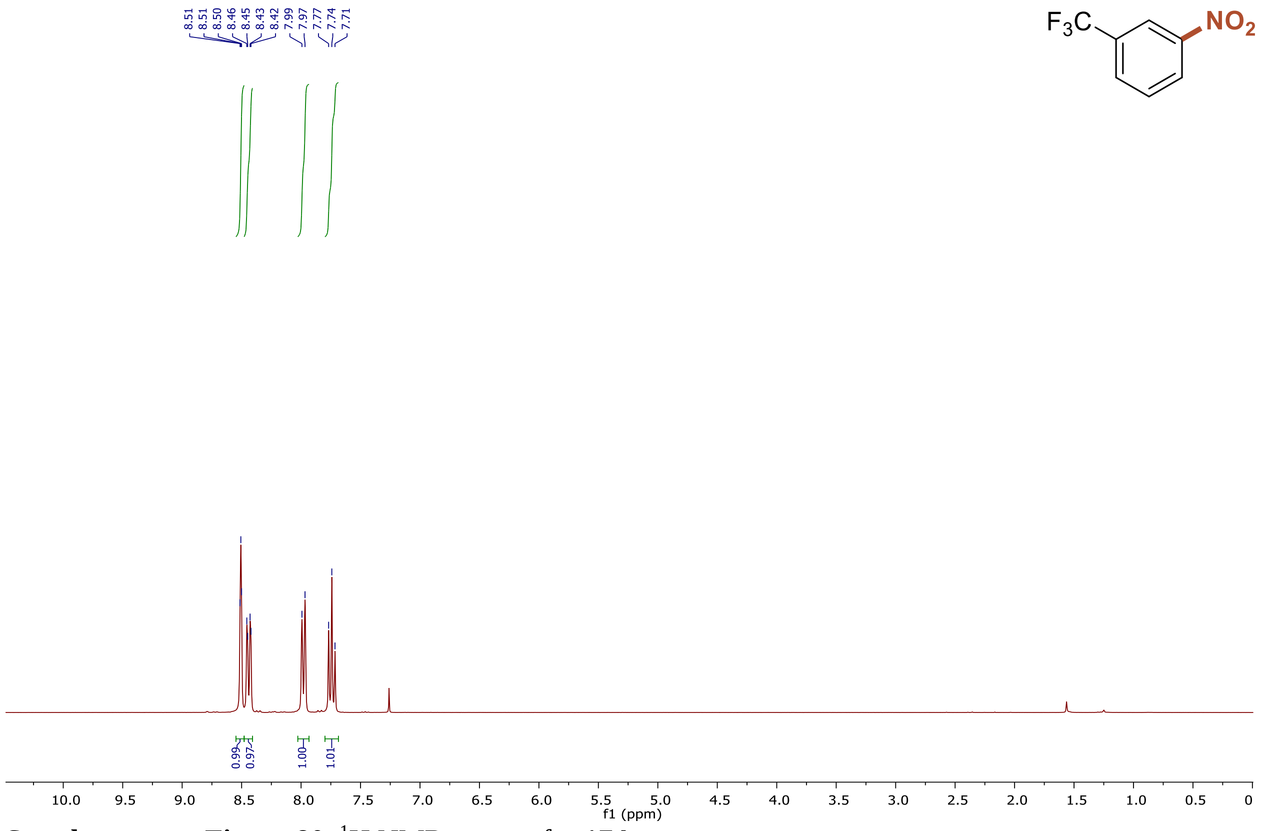

Supplementary Figure 80. ^{1}H NMR spectra for **17A**.

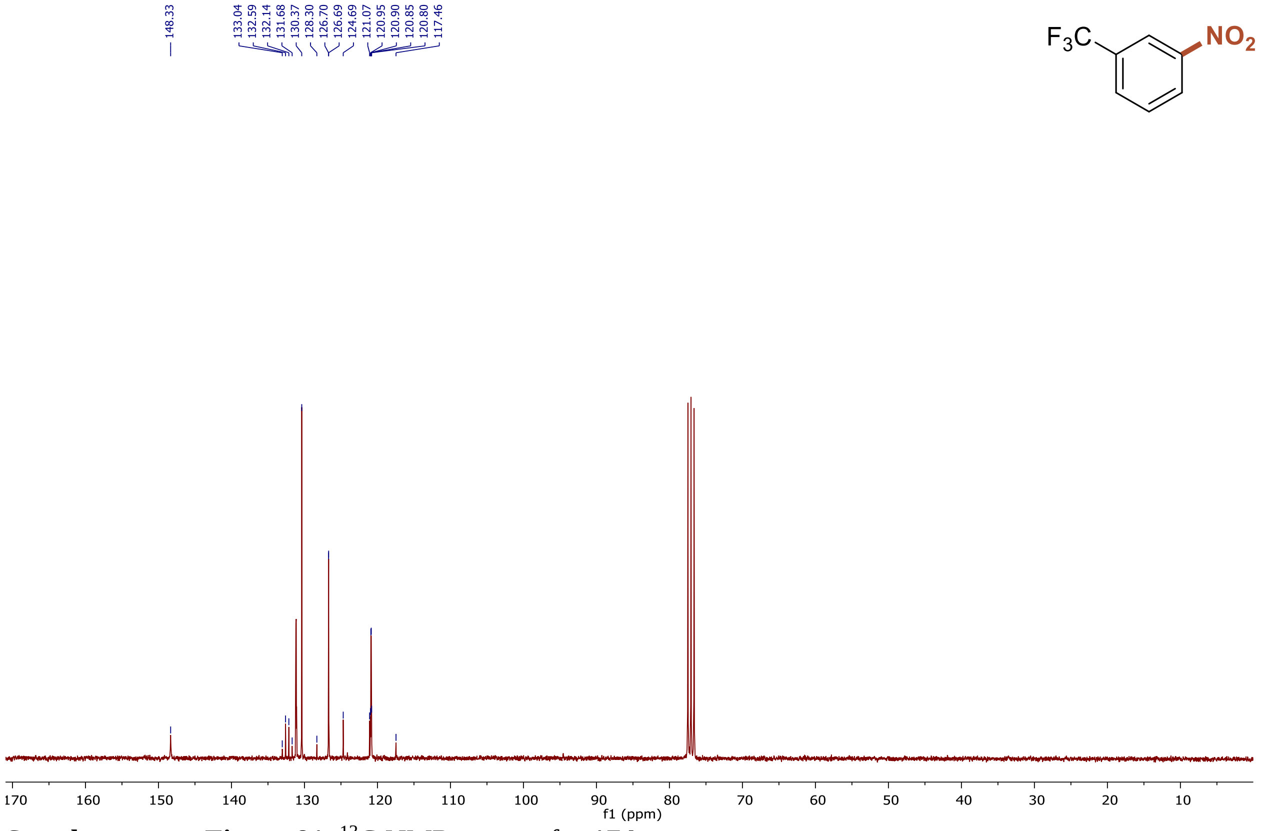

Supplementary Figure 81. ^{13}C NMR spectra for **17A**.

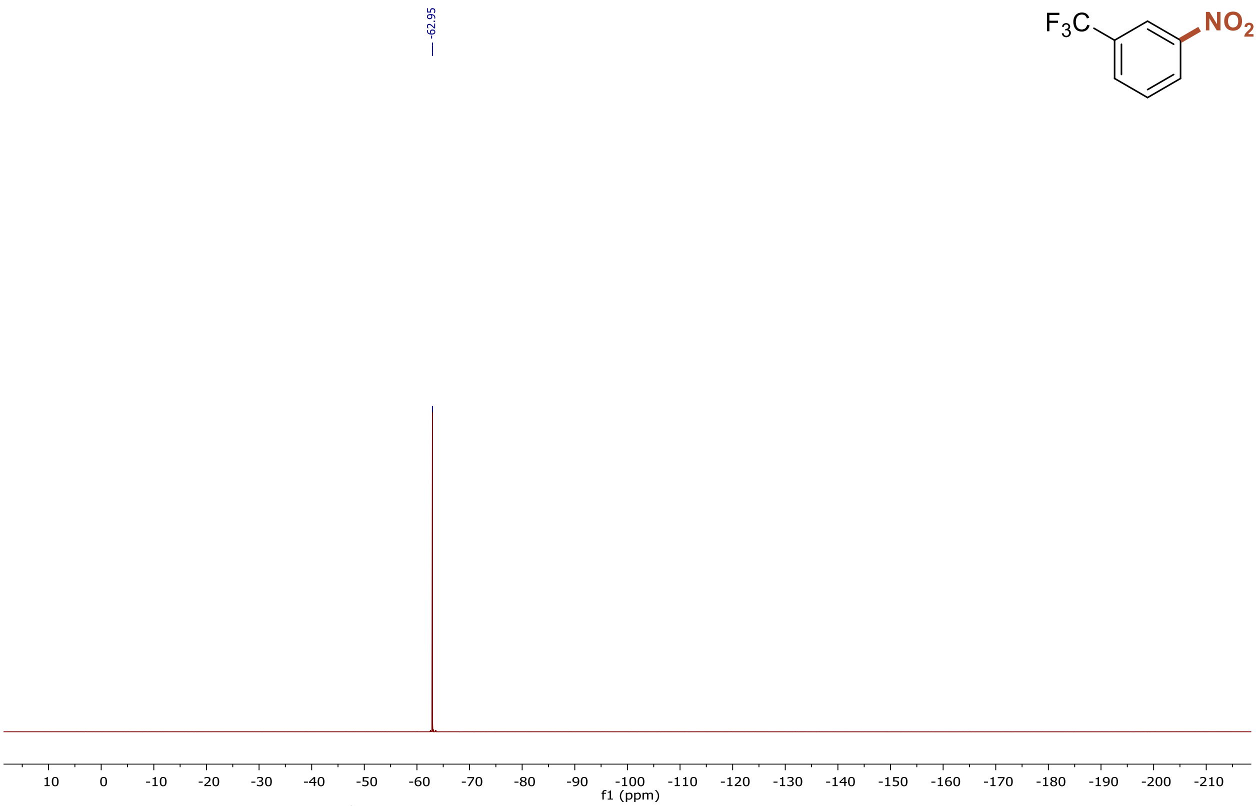

Supplementary Figure 82. ^{19}F NMR spectra for **17A**.

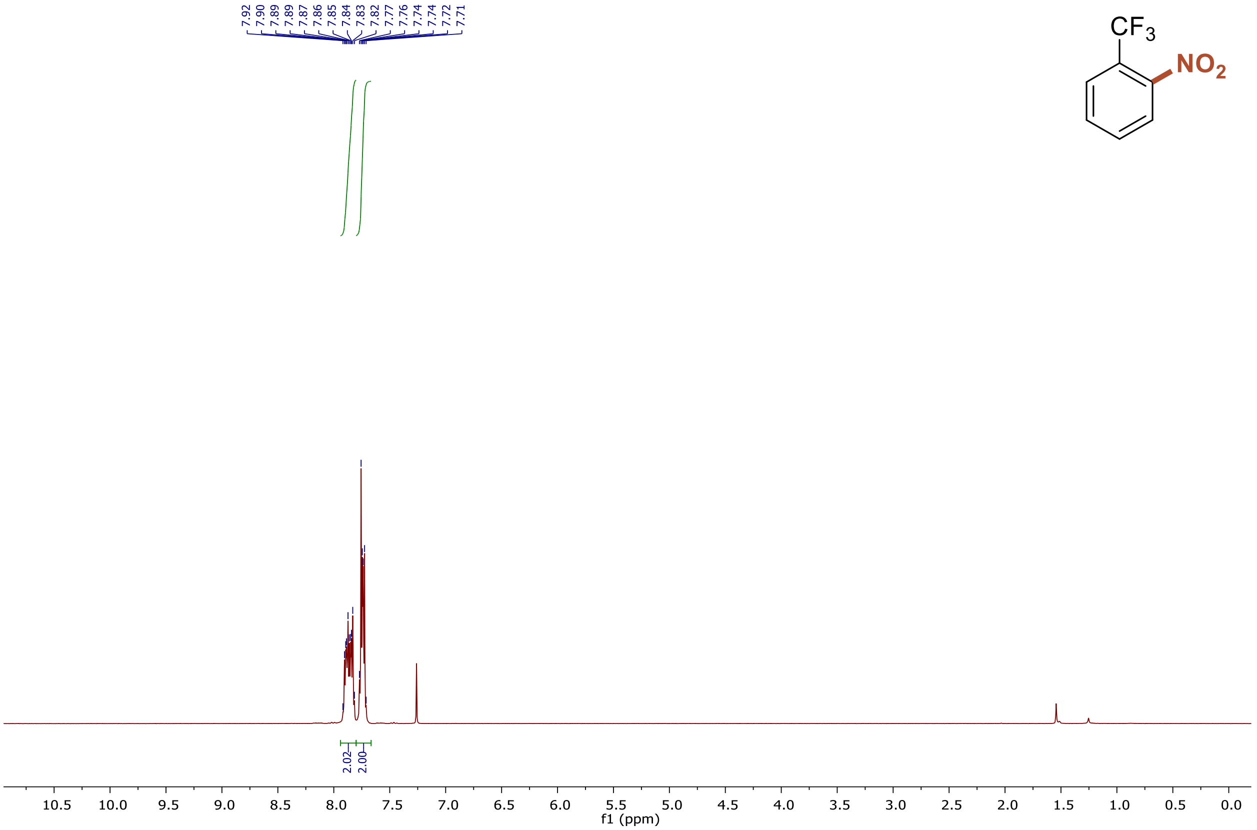

Supplementary Figure 83. ^{1}H NMR spectra for **17B**.

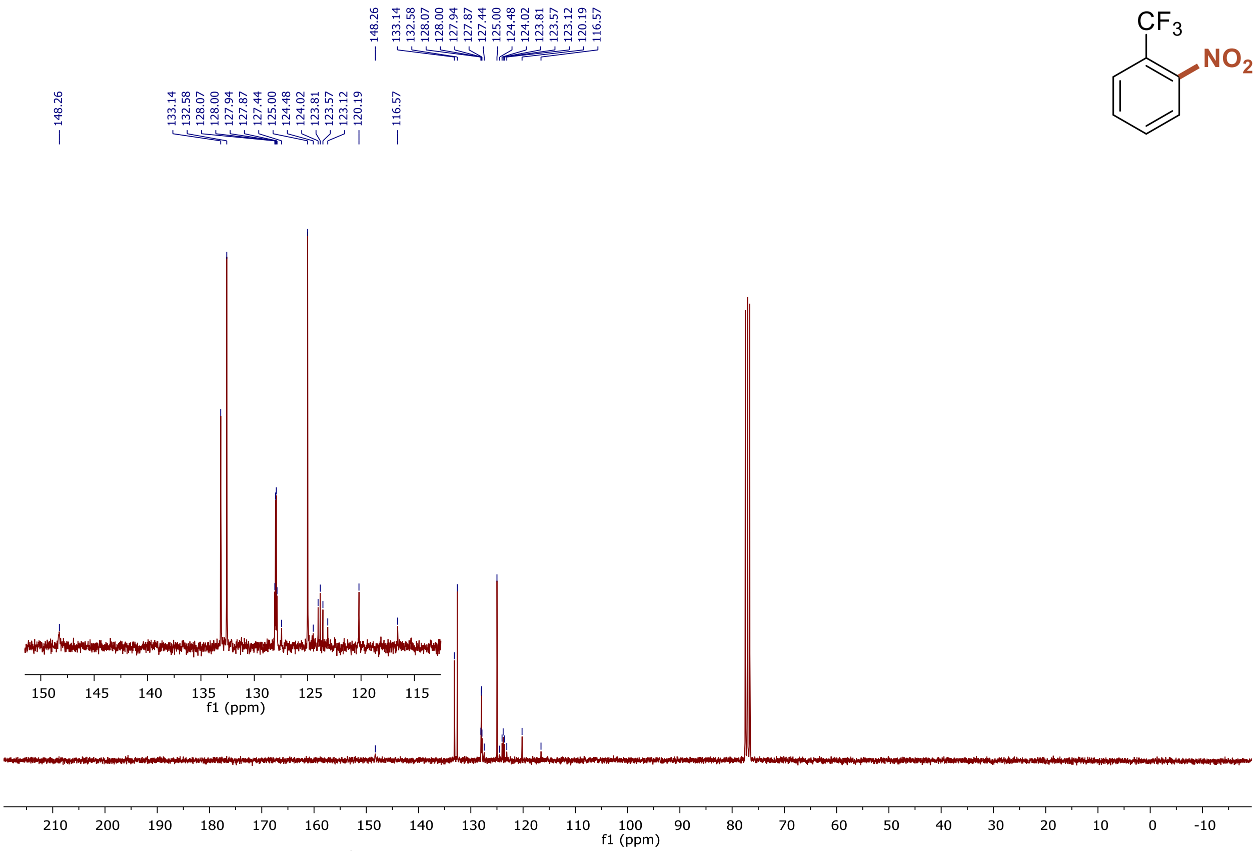

Supplementary Figure 84. ^{13}C NMR spectra for **17B**.

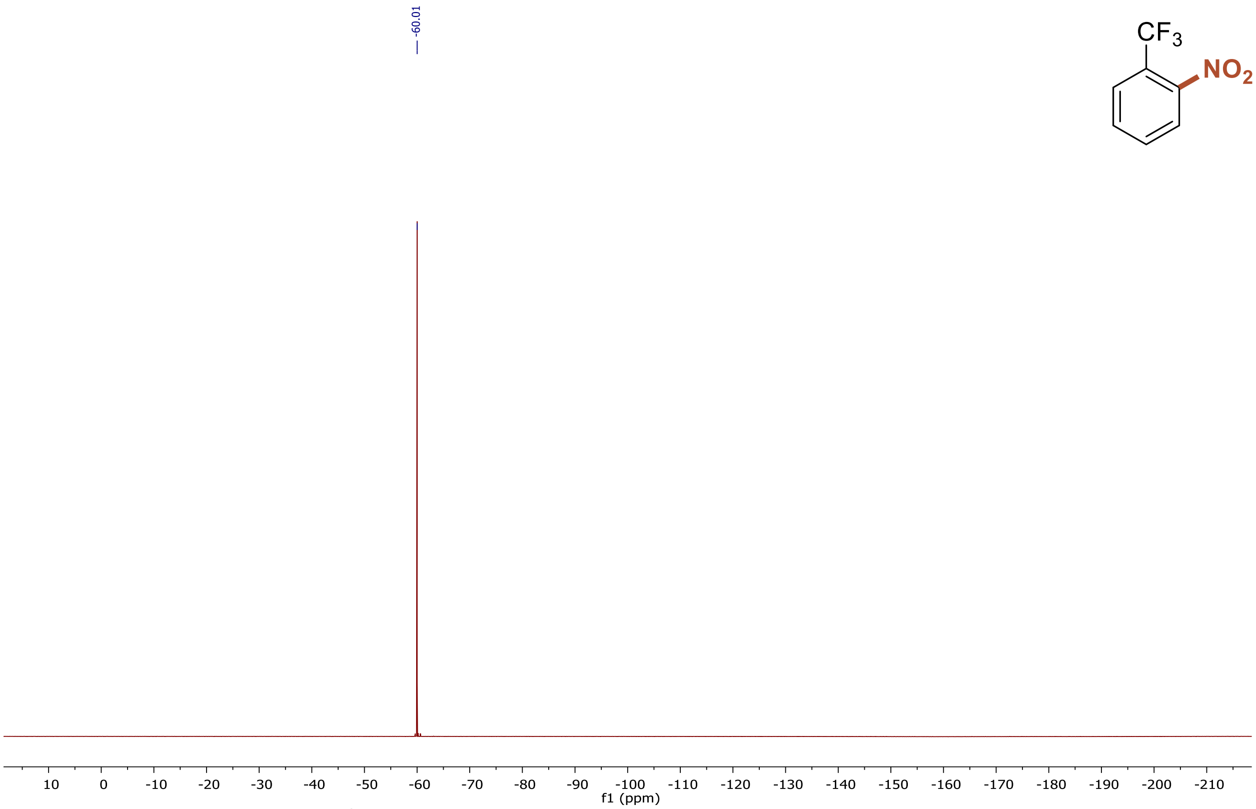

Supplementary Figure 85. ^{19}F NMR spectra for 17**B**.

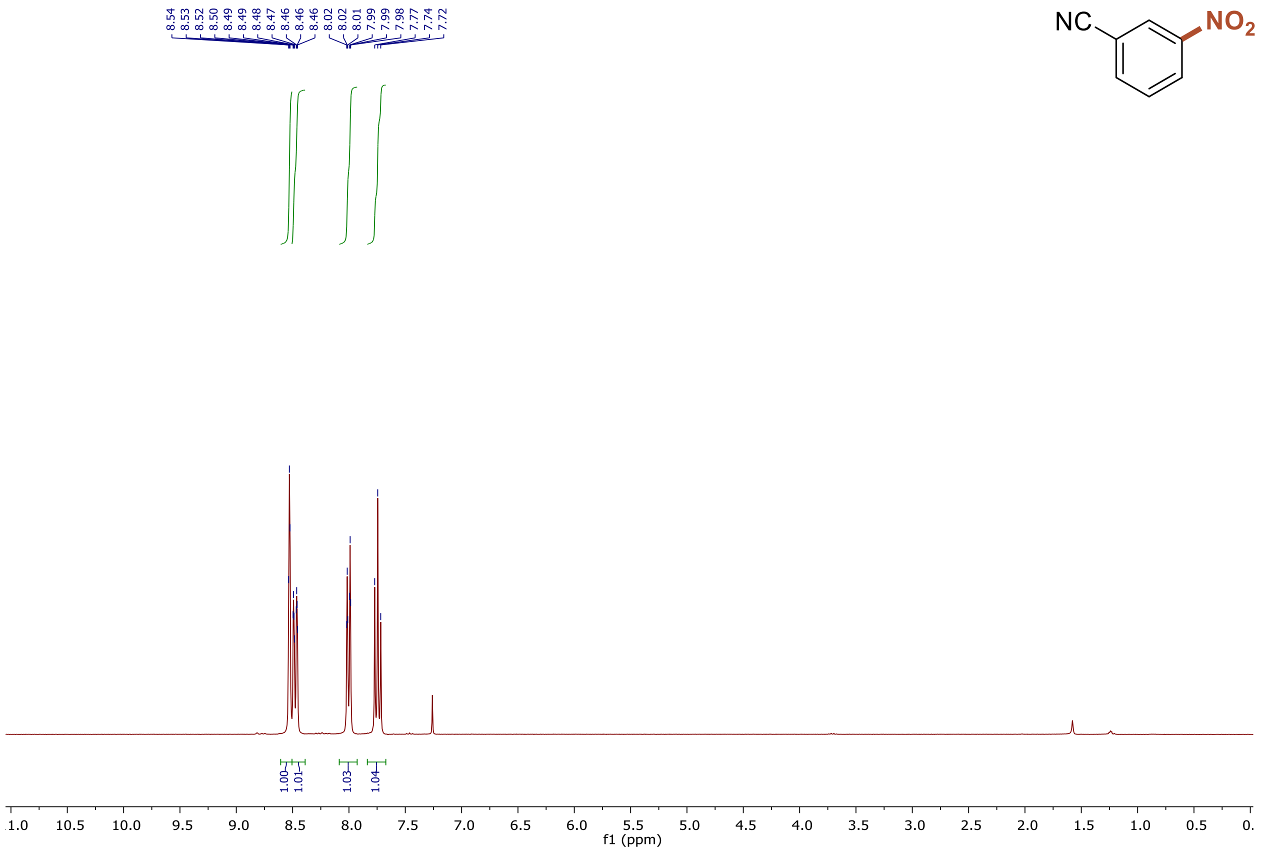

Supplementary Figure 86. ^{1}H NMR spectra for **18**.

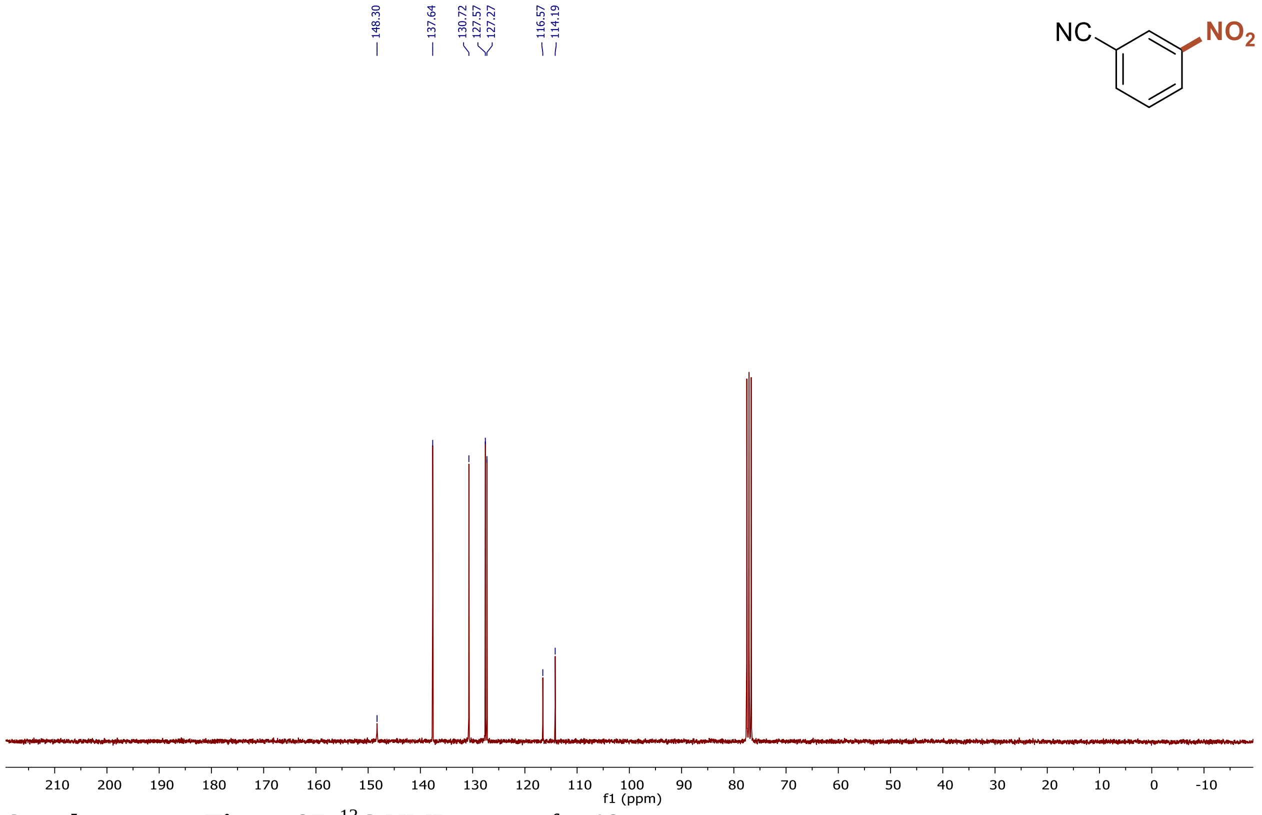

Supplementary Figure 87. ^{13}C NMR spectra for **18**.

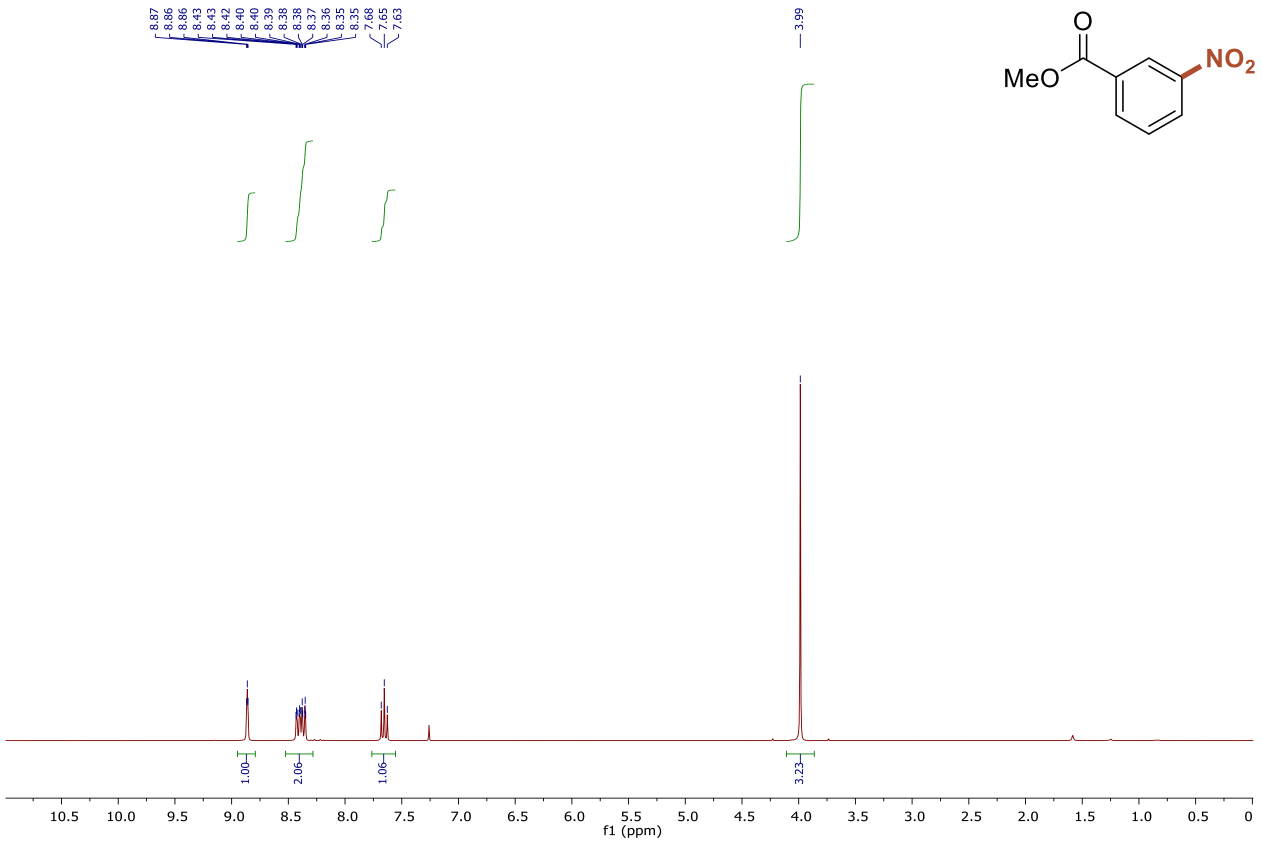

Supplementary Figure 88. ^{1}H NMR spectra for **19A**.

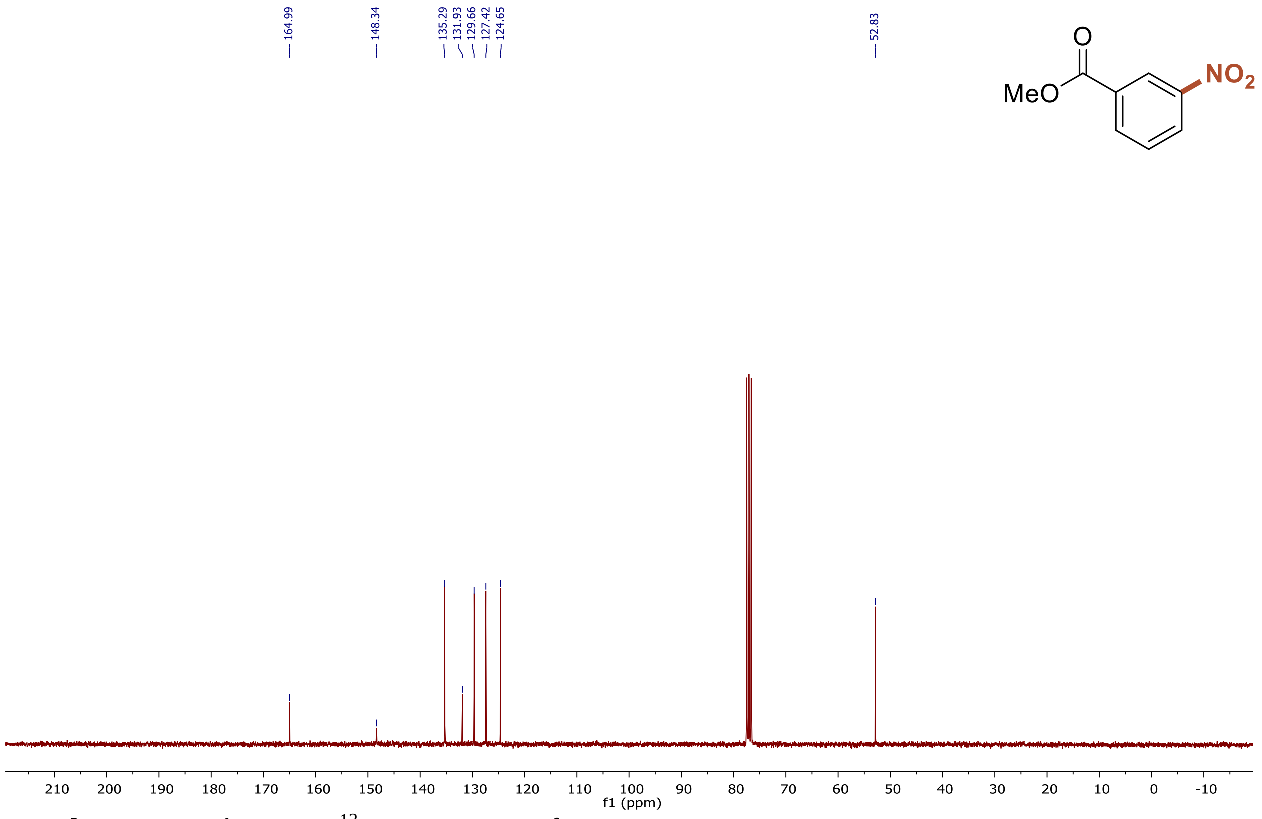

Supplementary Figure 89. ^{13}C NMR spectra for **19A**.

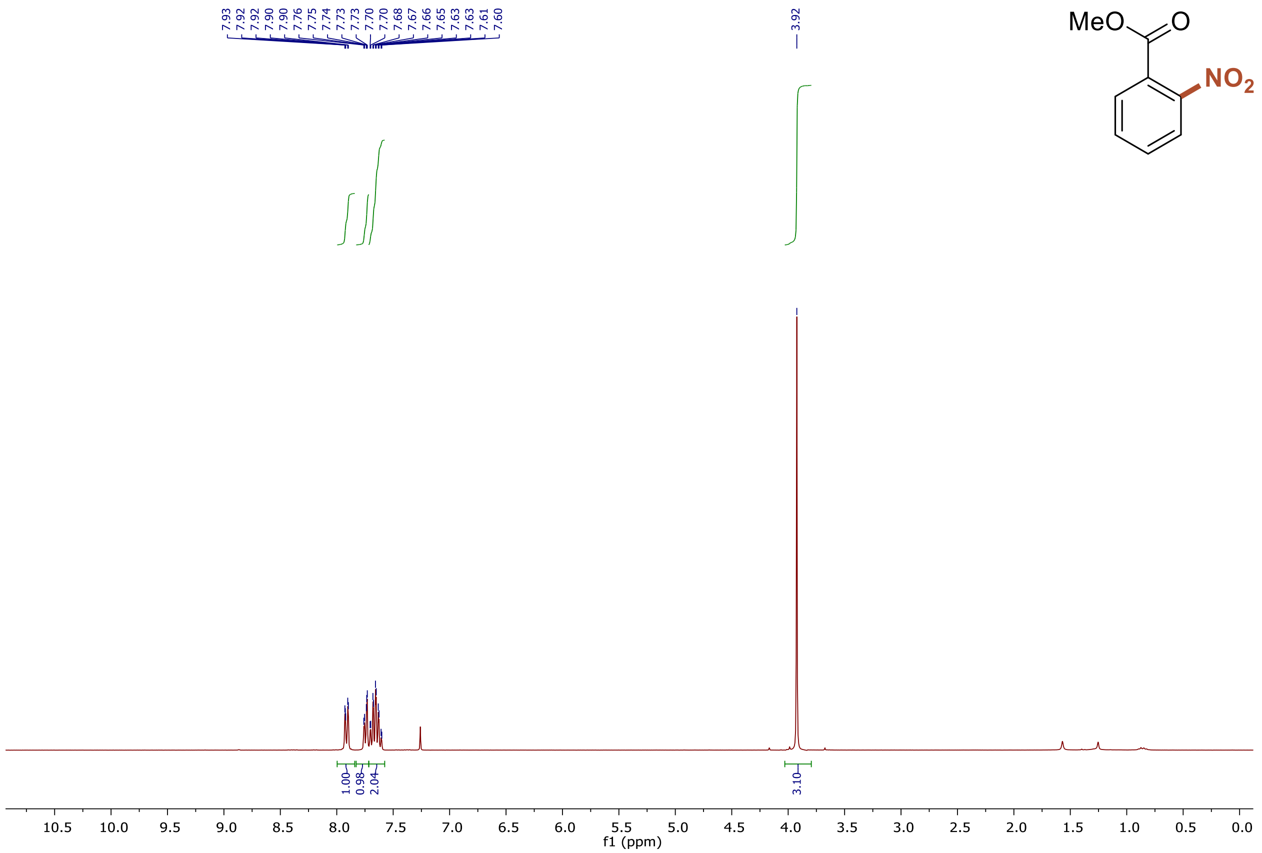

Supplementary Figure 90. ^{1}H NMR spectra for 1**9B**.

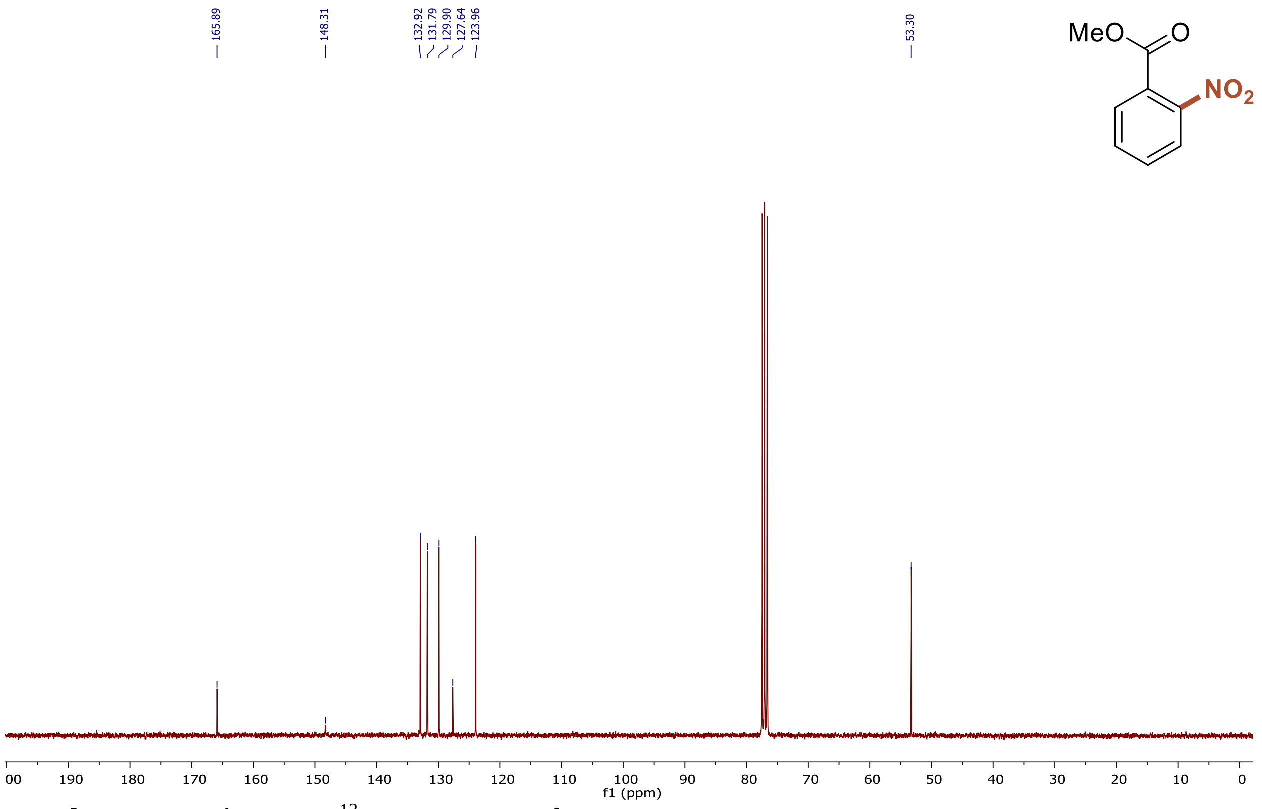

Supplementary Figure 91. ^{13}C NMR spectra for 1**9B**.

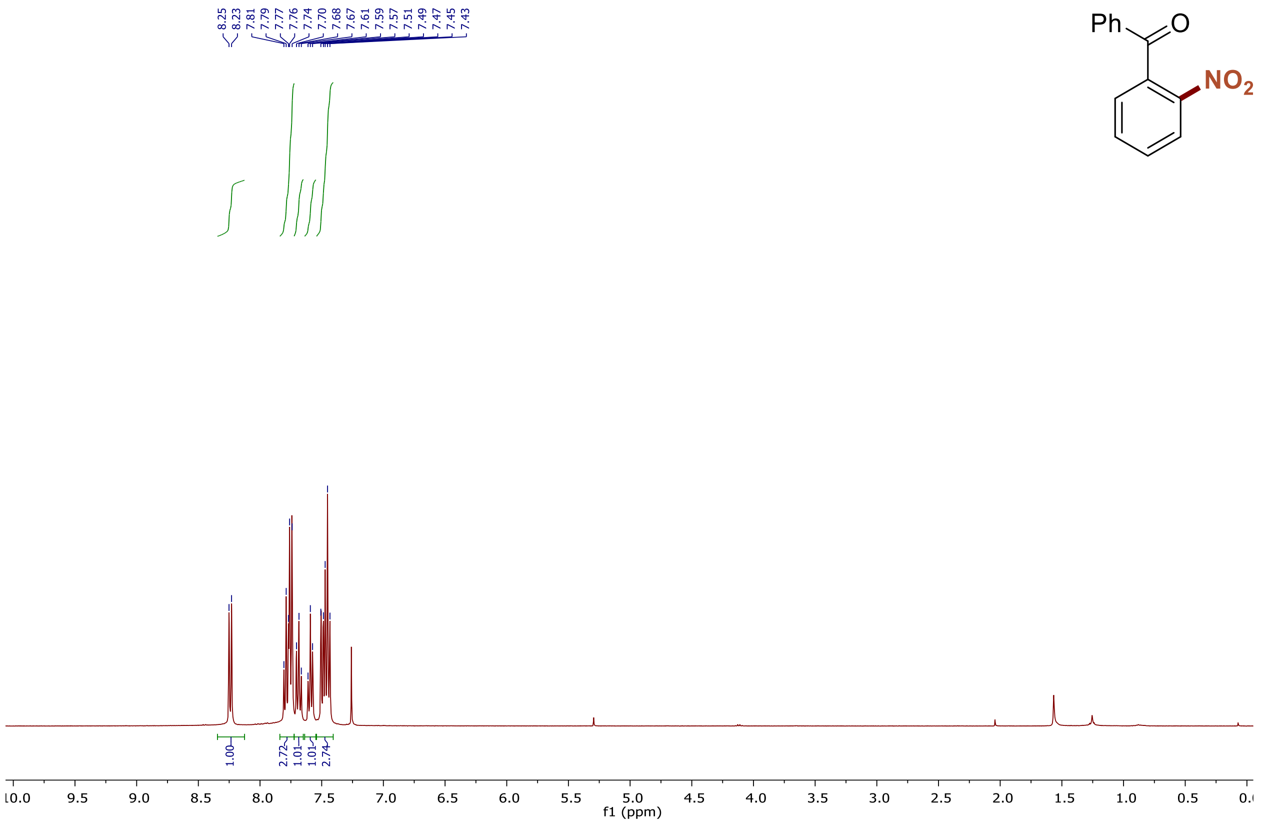

Supplementary Figure 92. ^{1}H NMR spectra for **20A**.

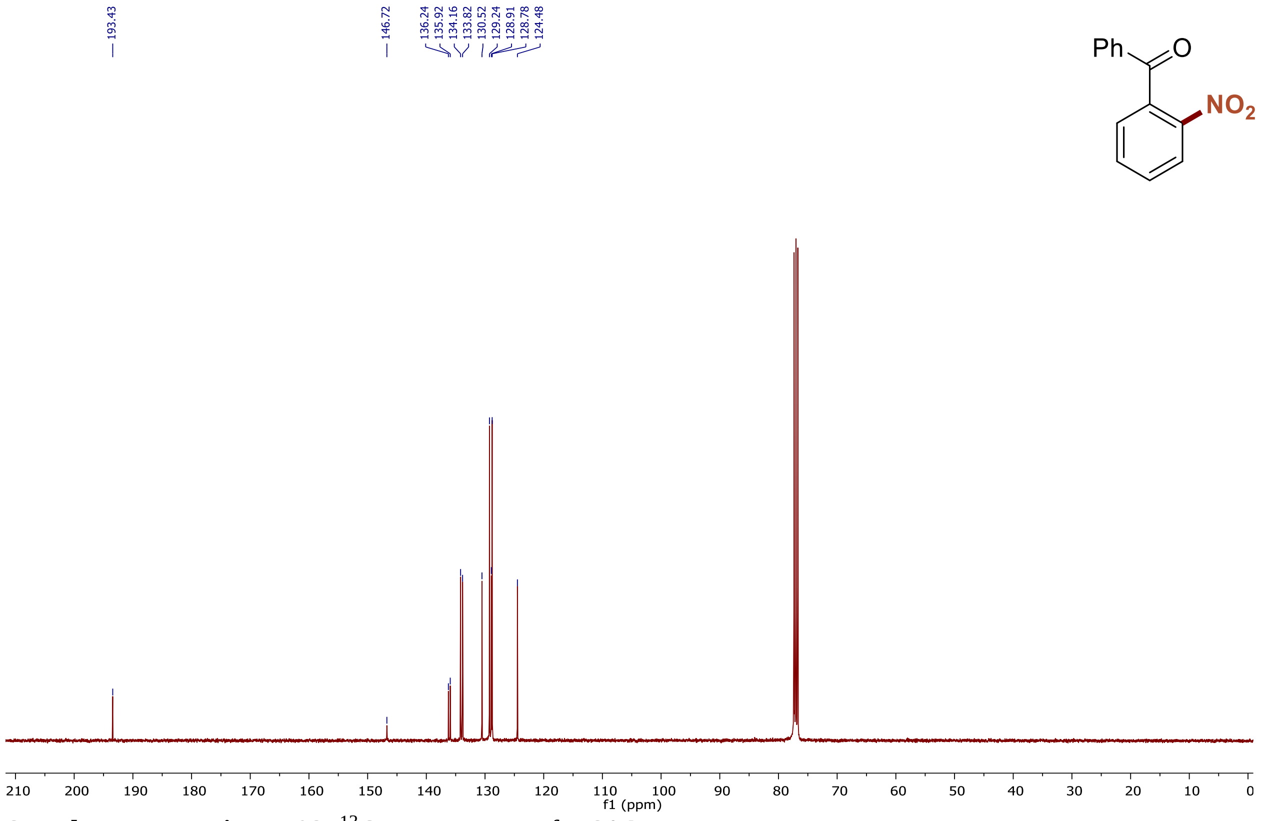

Supplementary Figure 93. ^{13}C NMR spectra for **20A**.

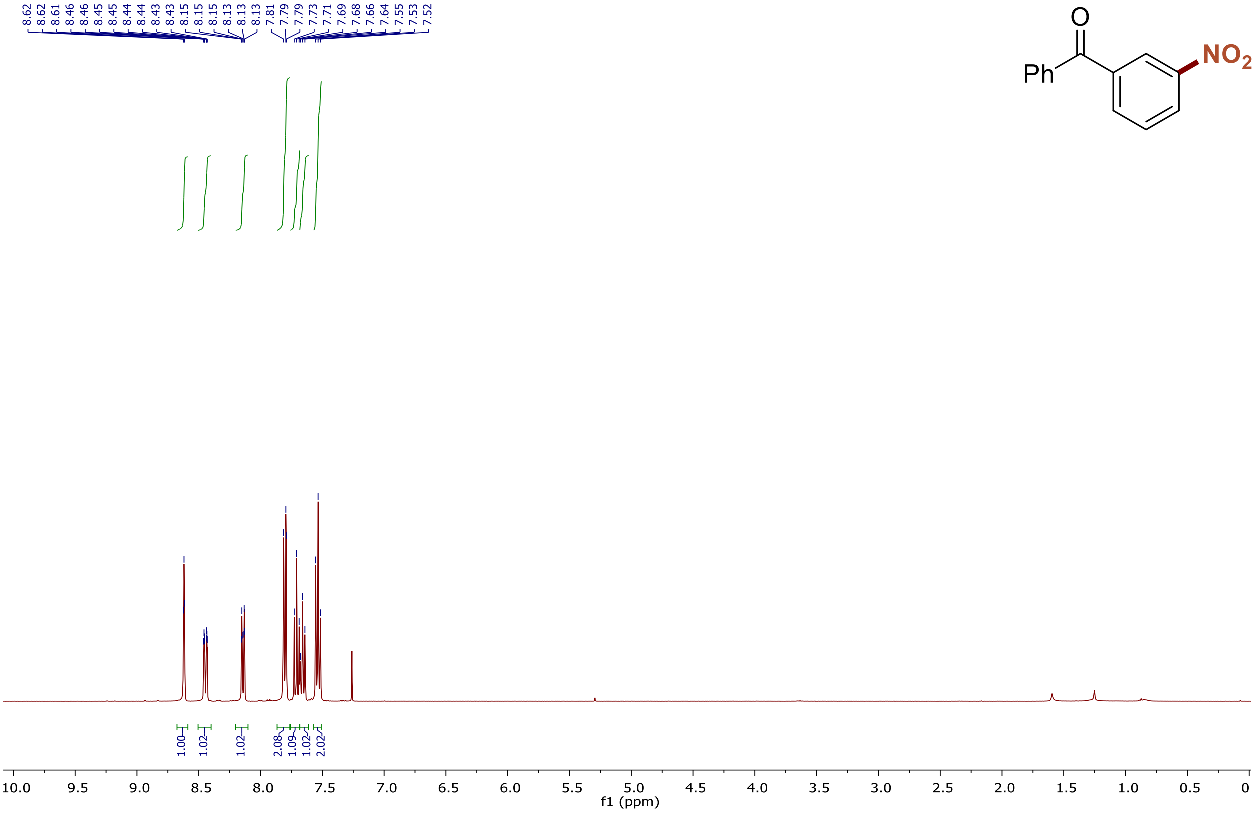

Supplementary Figure 94. ^{1}H NMR spectra for **20B**.

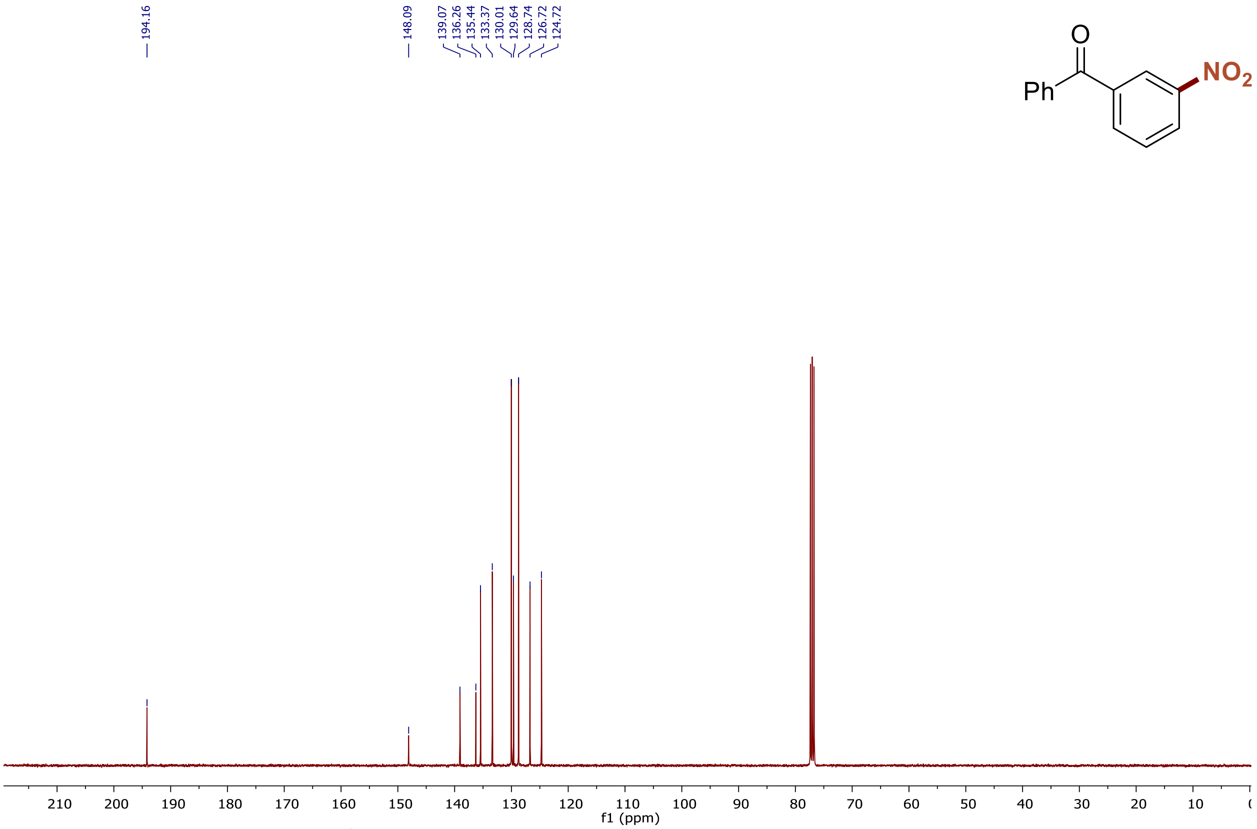

Supplementary Figure 95. ^{13}C NMR spectra for **20B**.

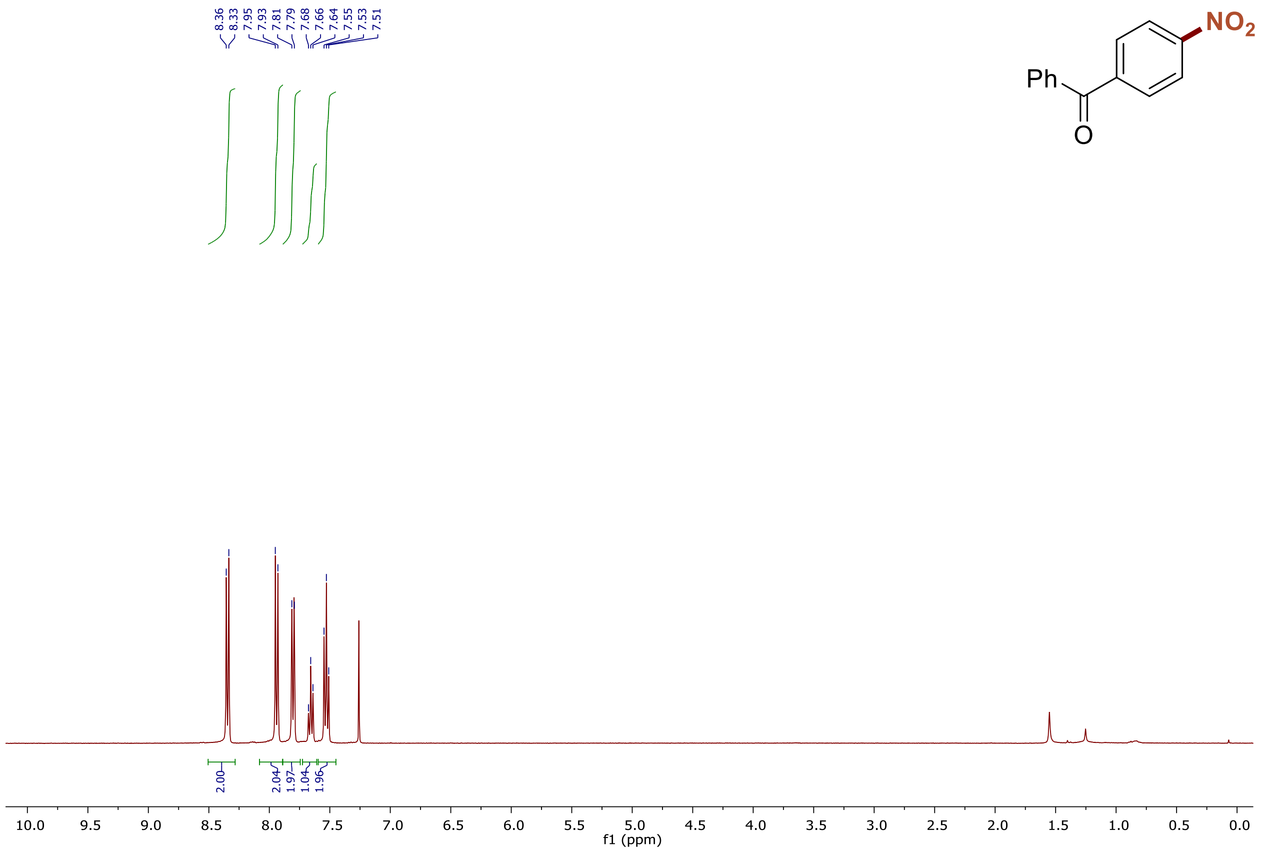

Supplementary Figure 96. ^{1}H NMR spectra for **20C**.

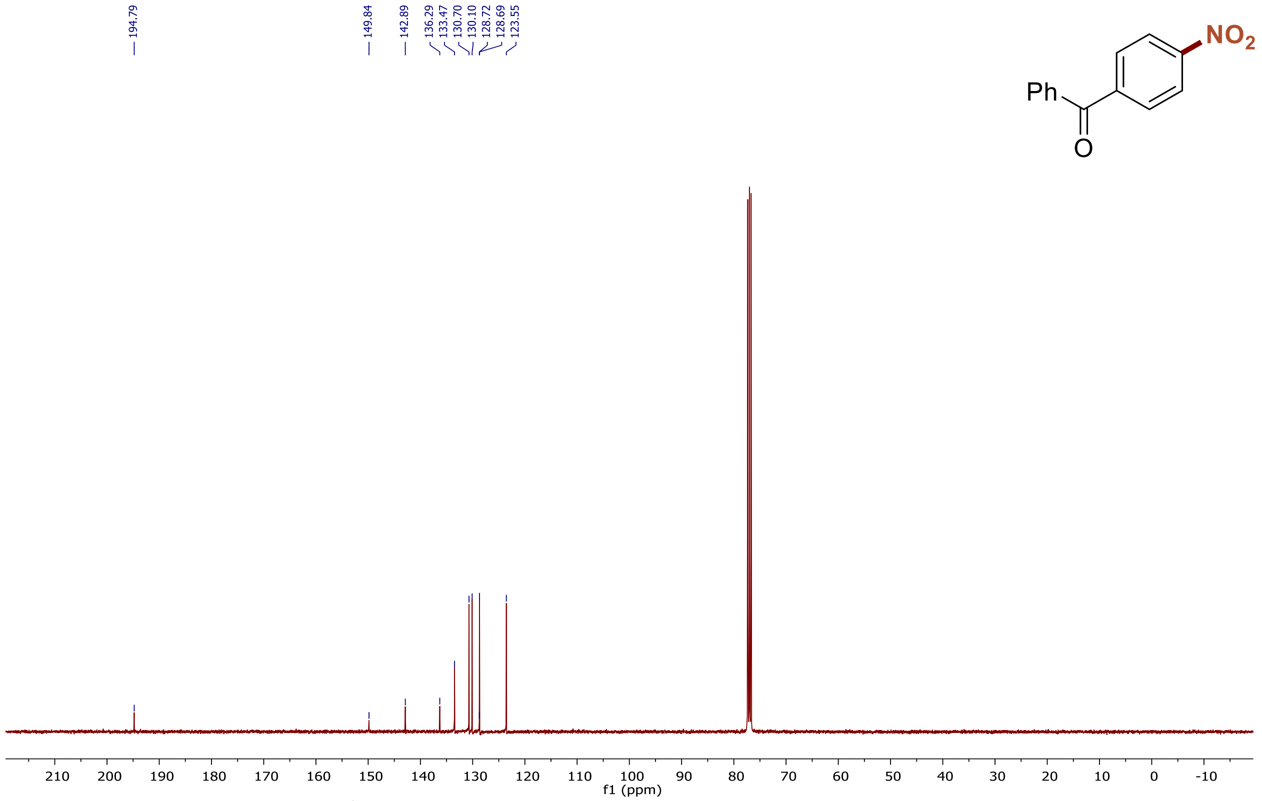

Supplementary Figure 97. ^{13}C NMR spectra for **20C**.

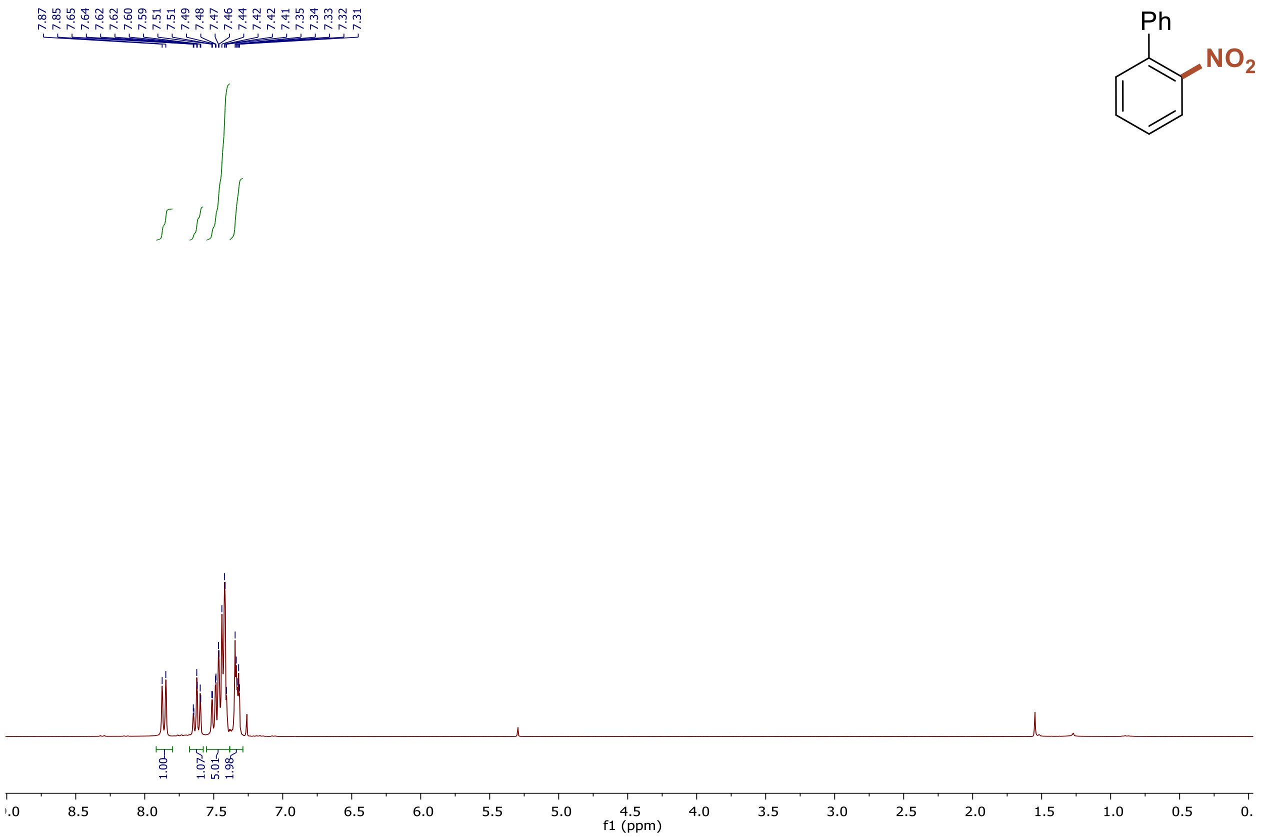

Supplementary Figure 98. ^{1}H NMR spectra for **21A**.

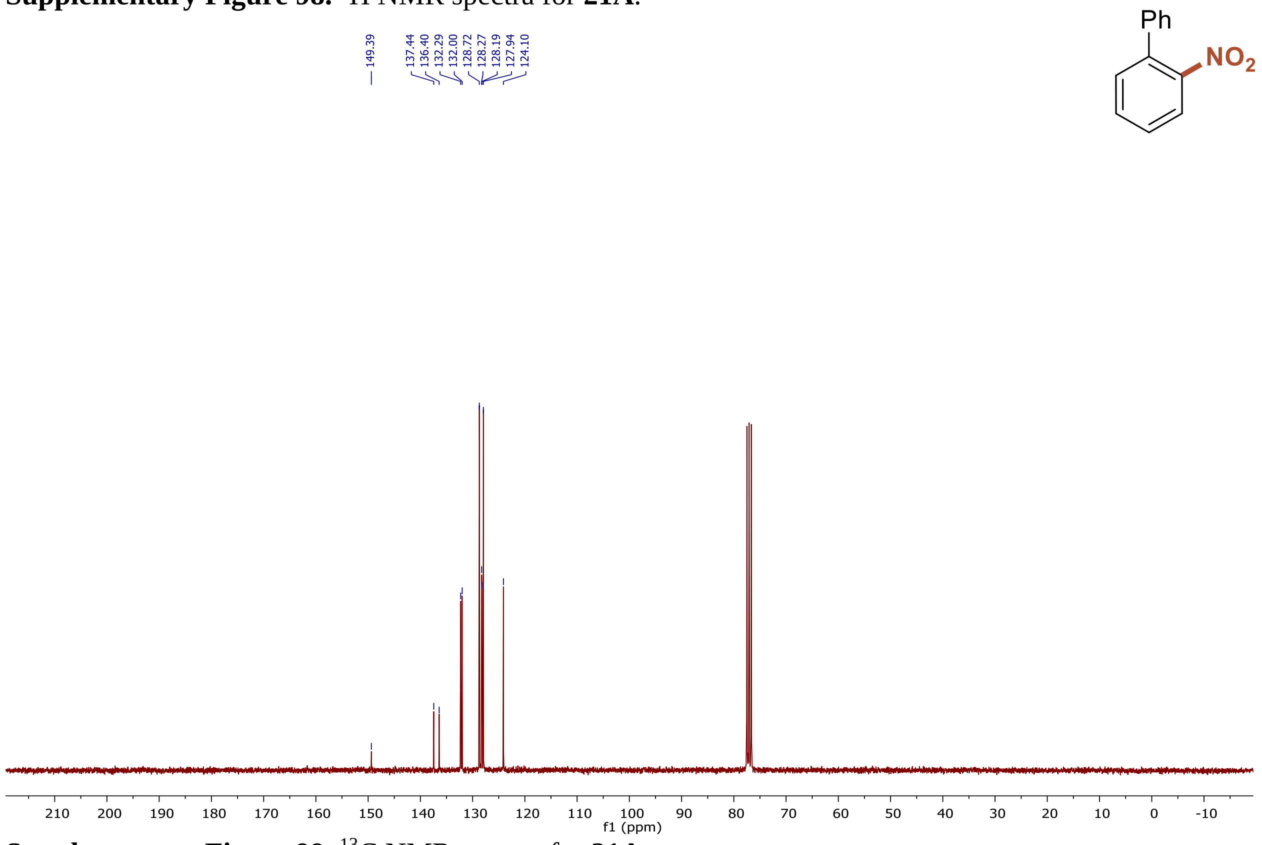

Supplementary Figure 99. ^{13}C NMR spectra for **21A**.

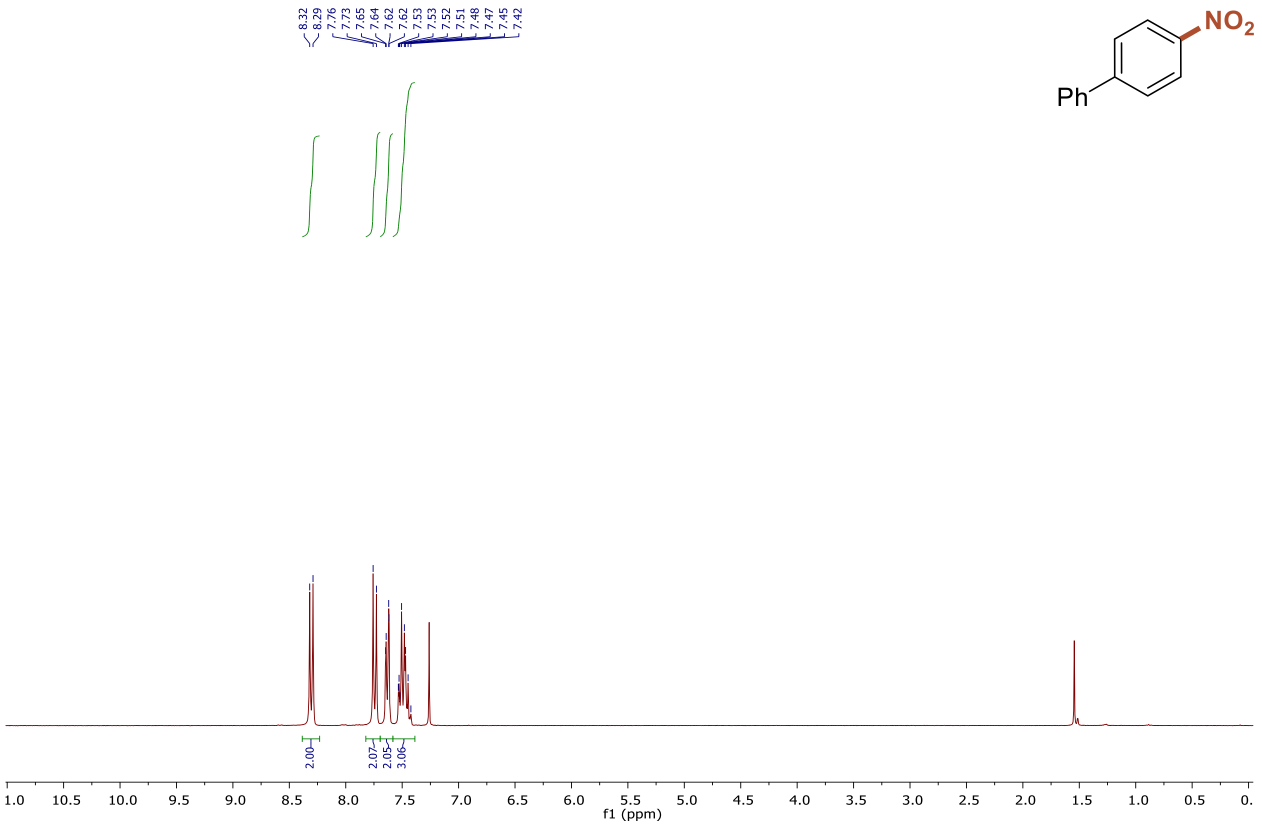

Supplementary Figure 100. ^{1}H NMR spectra for **21B**.

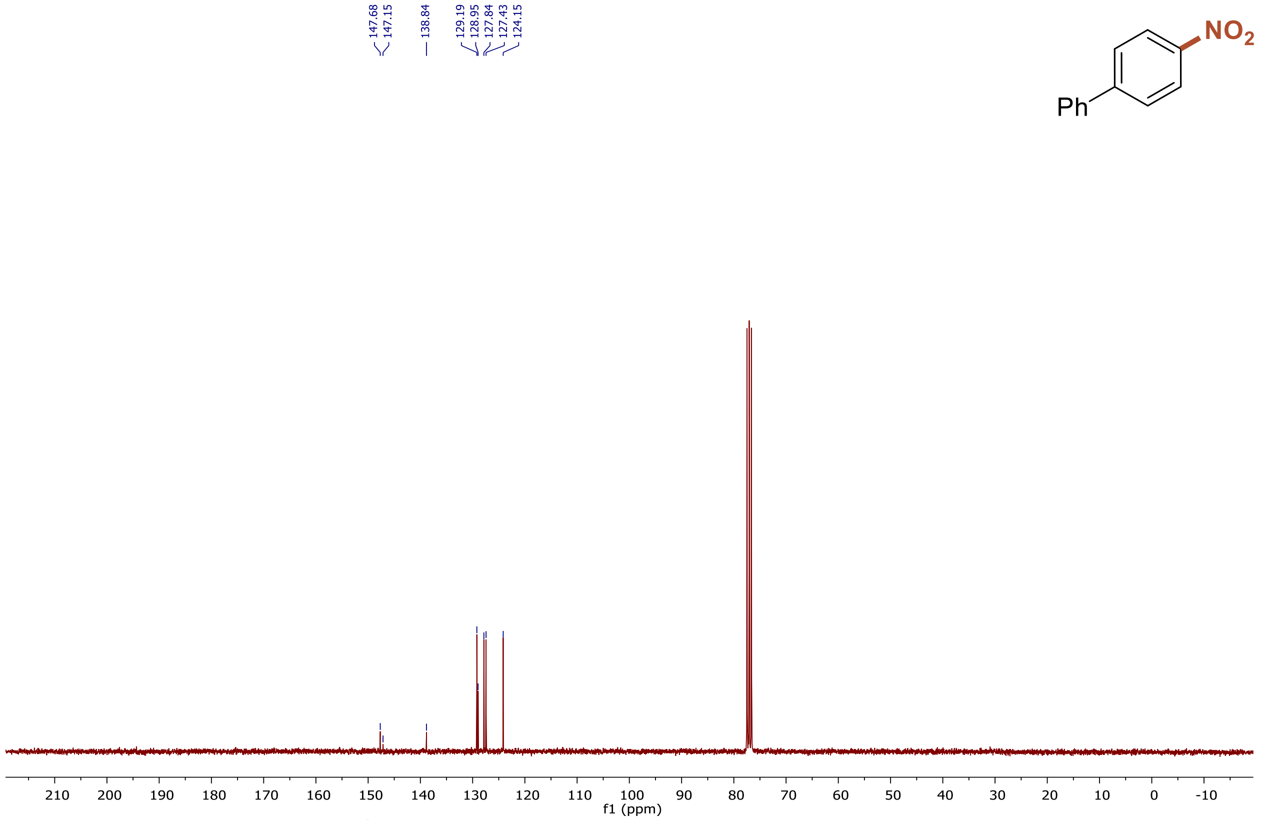

Supplementary Figure 101. ^{13}C NMR spectra for **21B**.

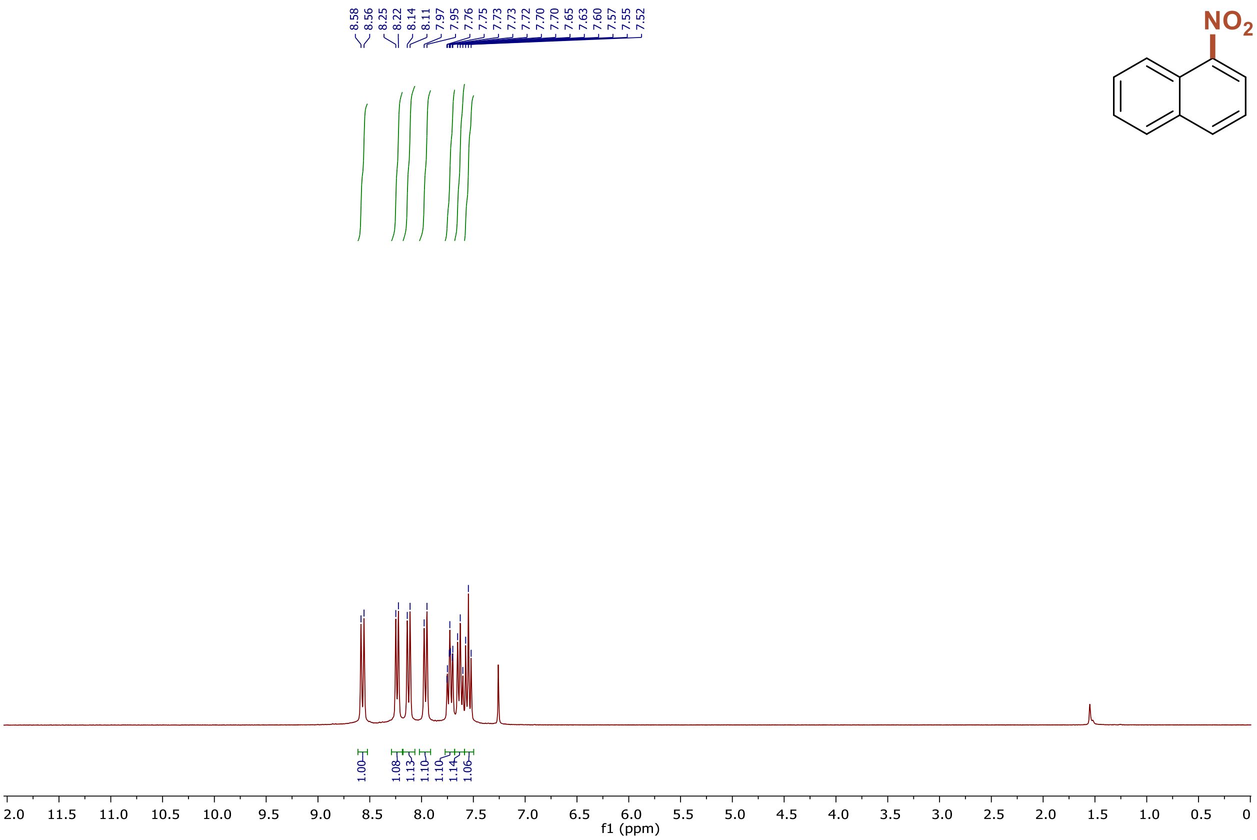

Supplementary Figure 102. ^{1}H NMR spectra for **22**.

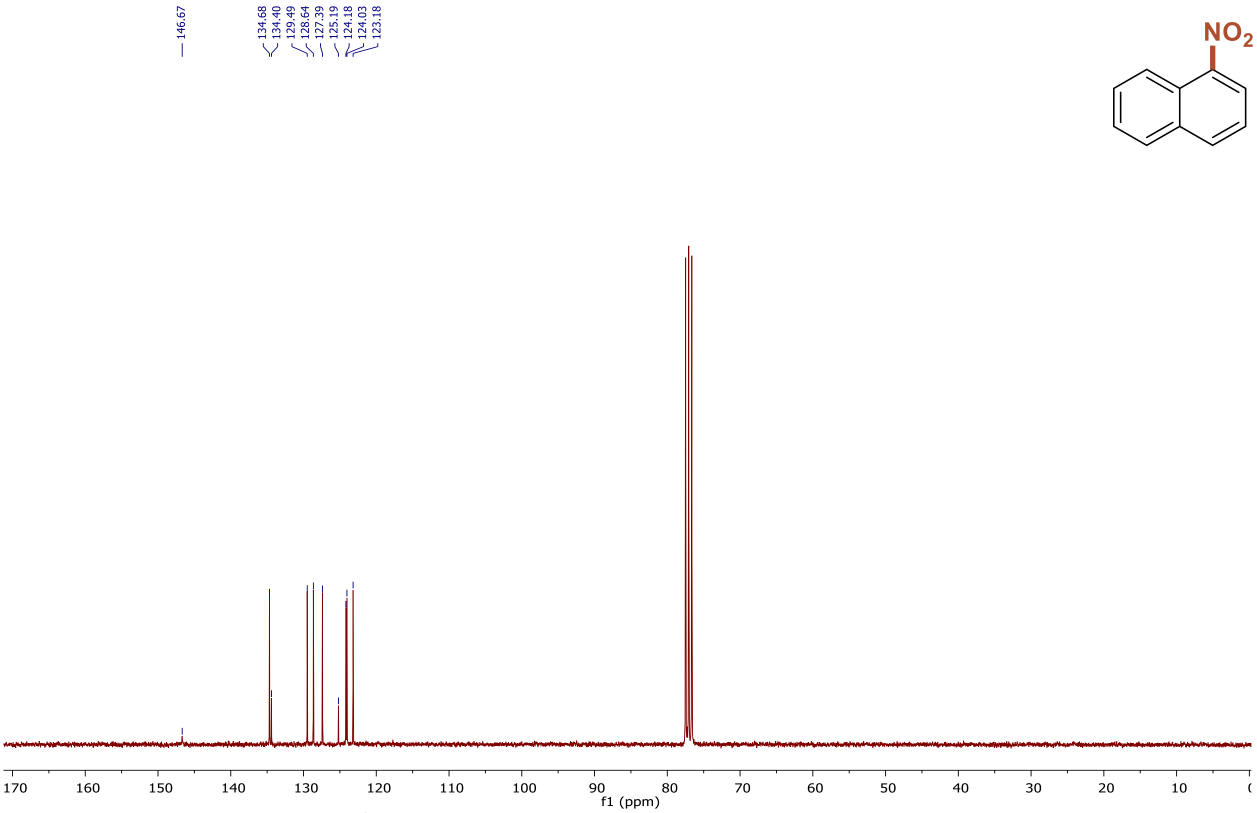

Supplementary Figure 103. ^{13}C NMR spectra for **22**.

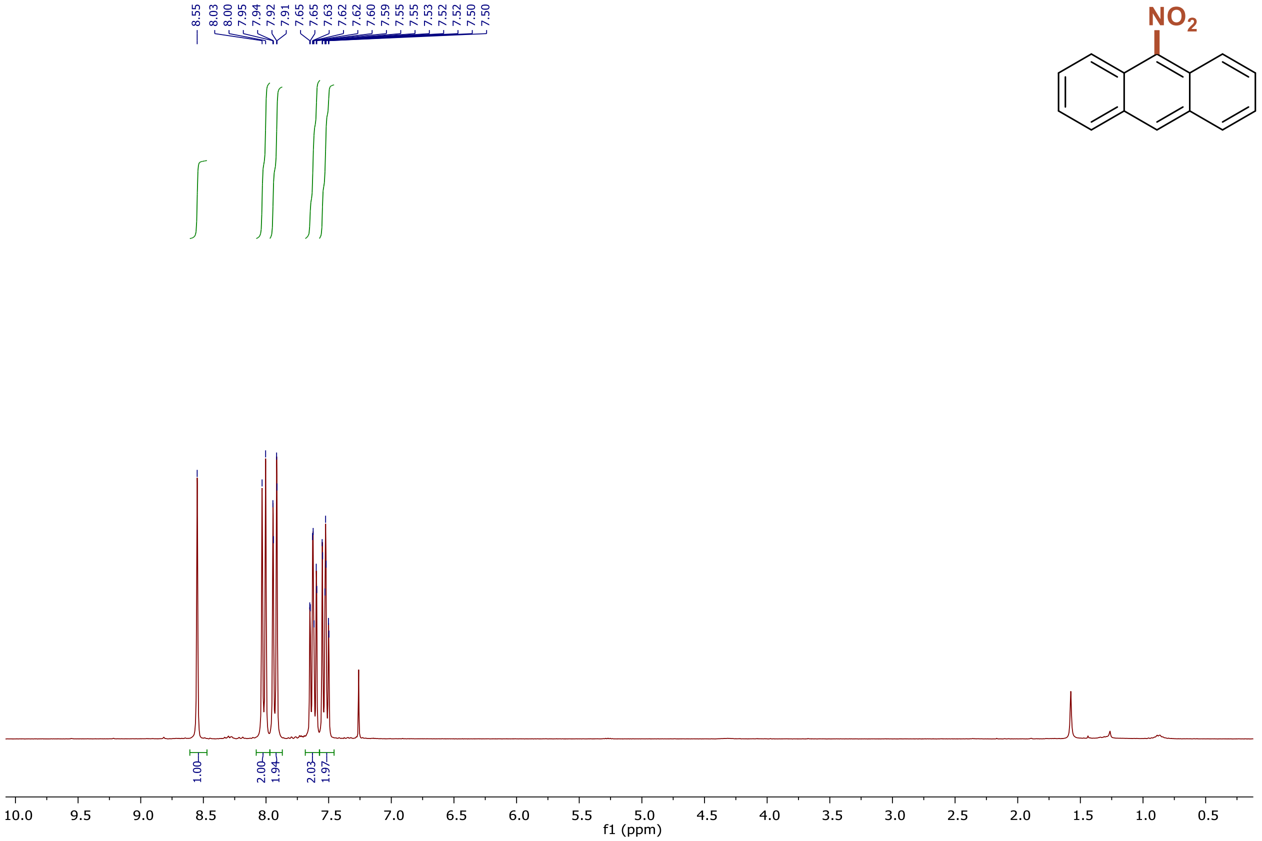

Supplementary Figure 104. ^{1}H NMR spectra for **23**.

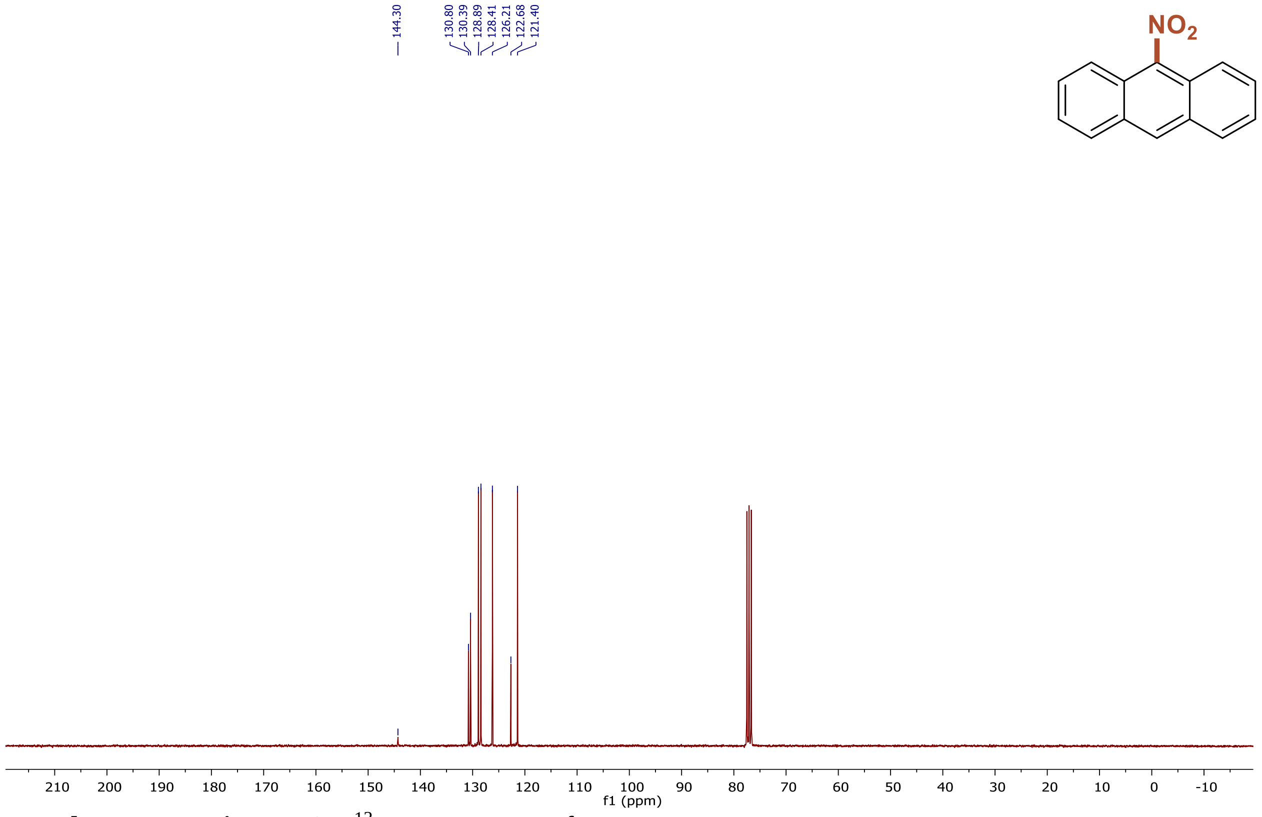

Supplementary Figure 105. ^{13}C NMR spectra for **23**.

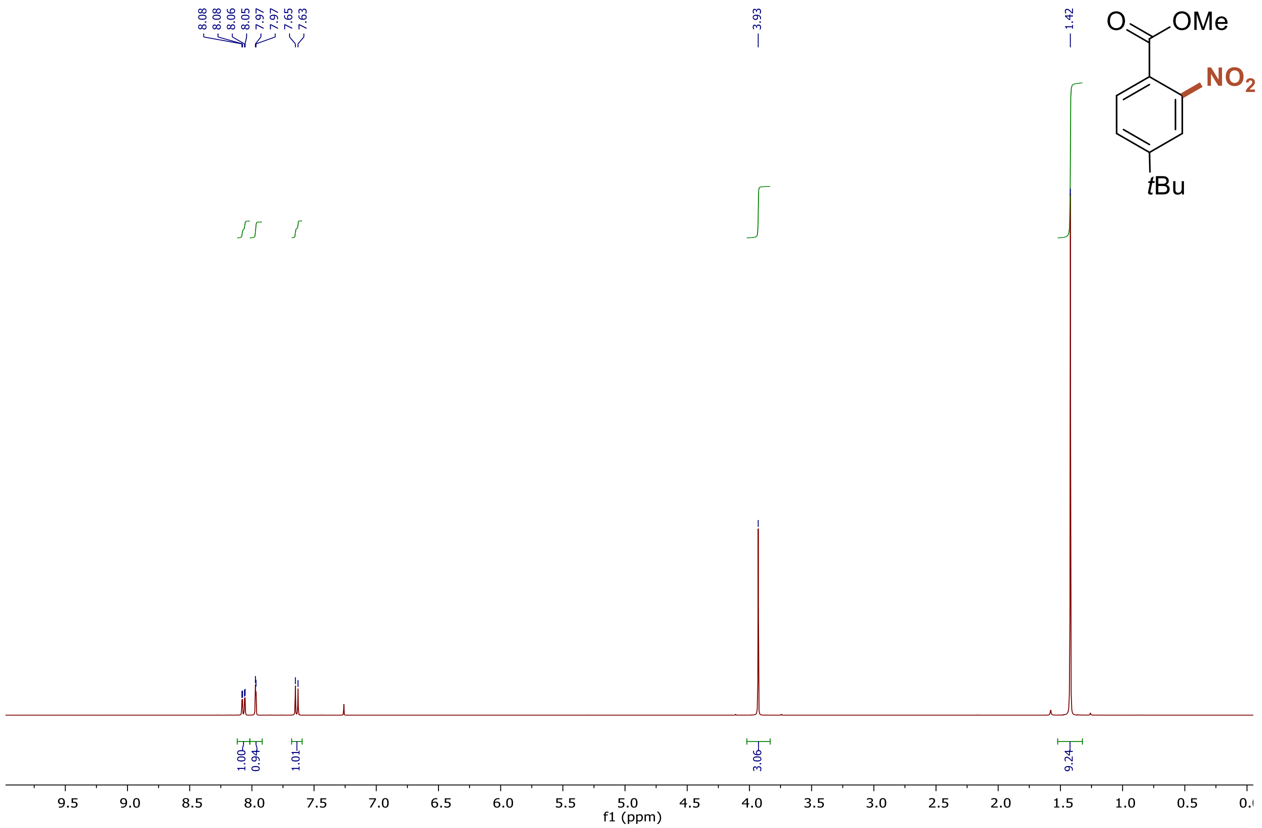

Supplementary Figure 106. ^{1}H NMR spectra for **24A**.

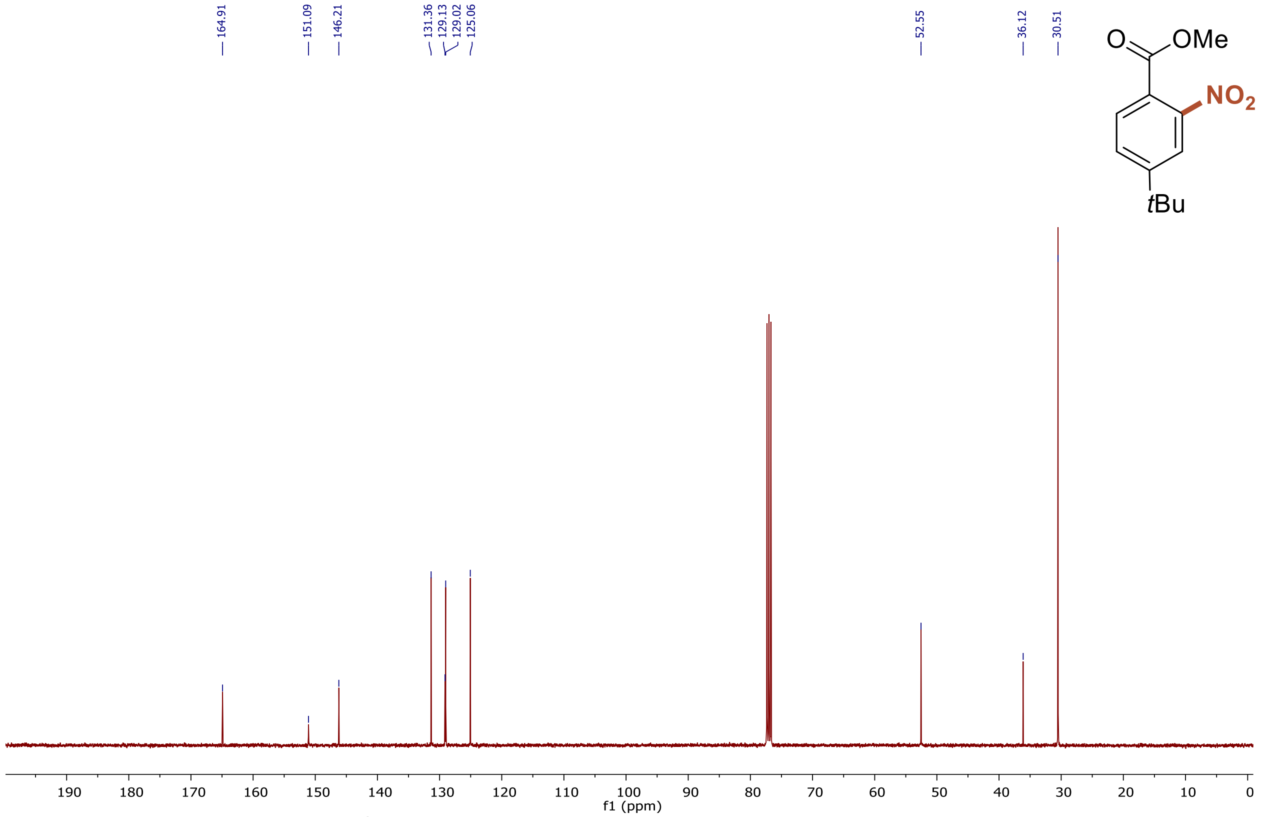

Supplementary Figure 107. ^{13}C NMR spectra for **24A**.

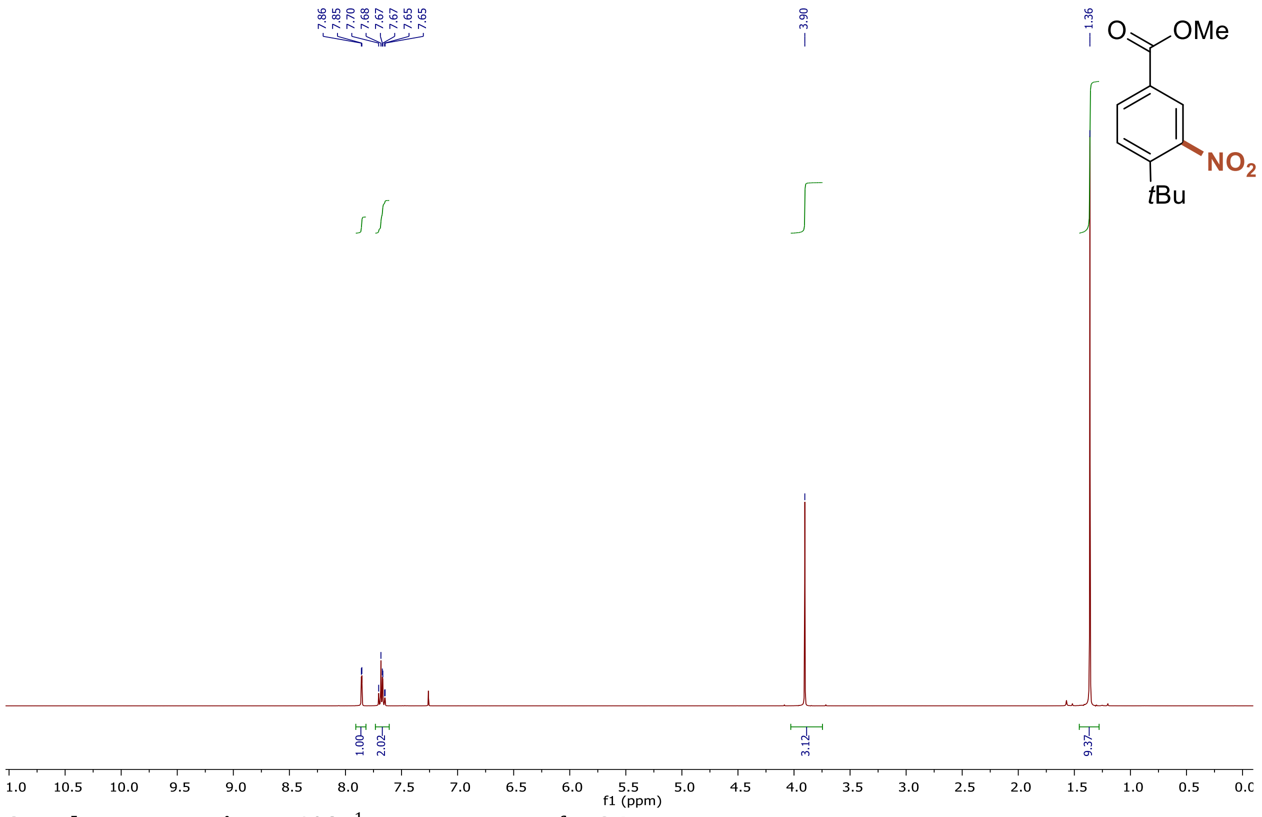

Supplementary Figure 108. ^{1}H NMR spectra for **24B**.

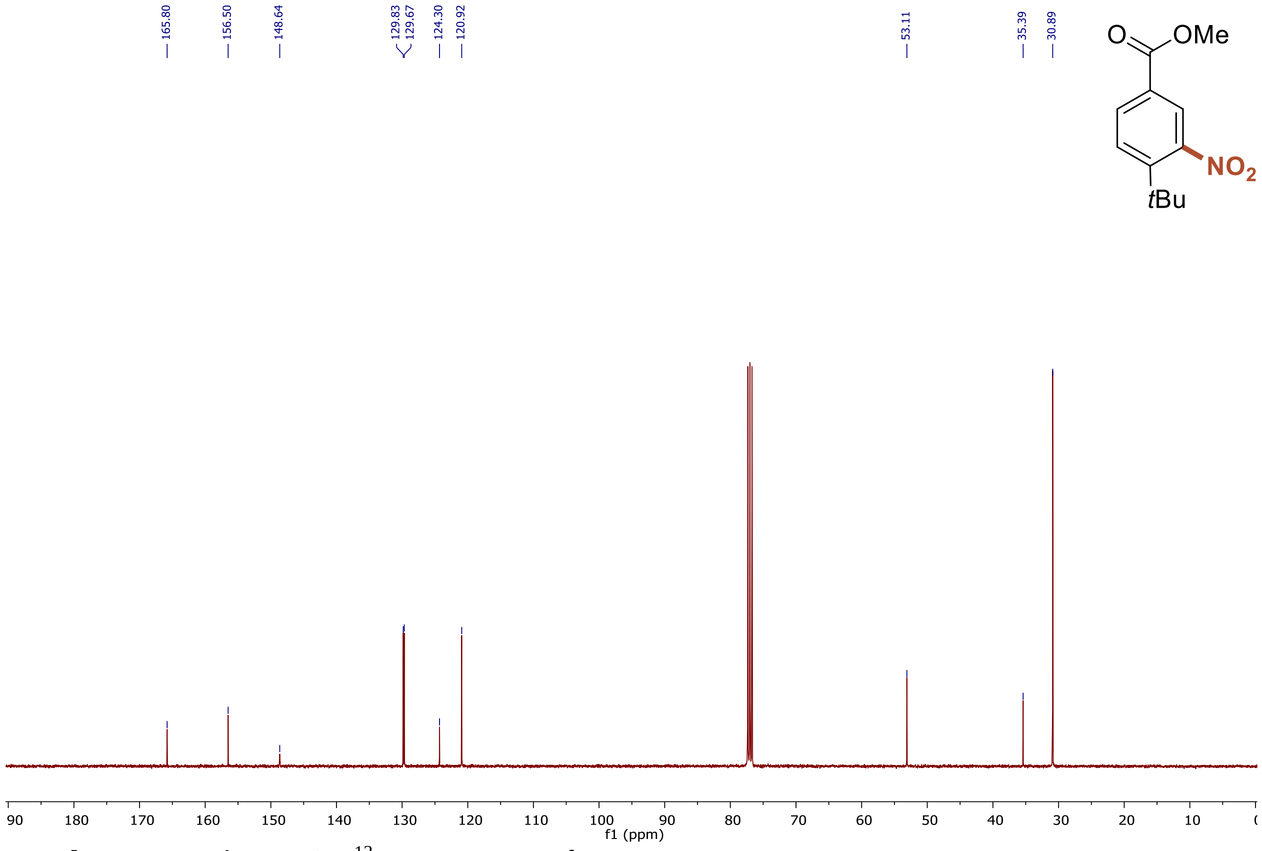

Supplementary Figure 109. ^{13}C NMR spectra for **24B**.

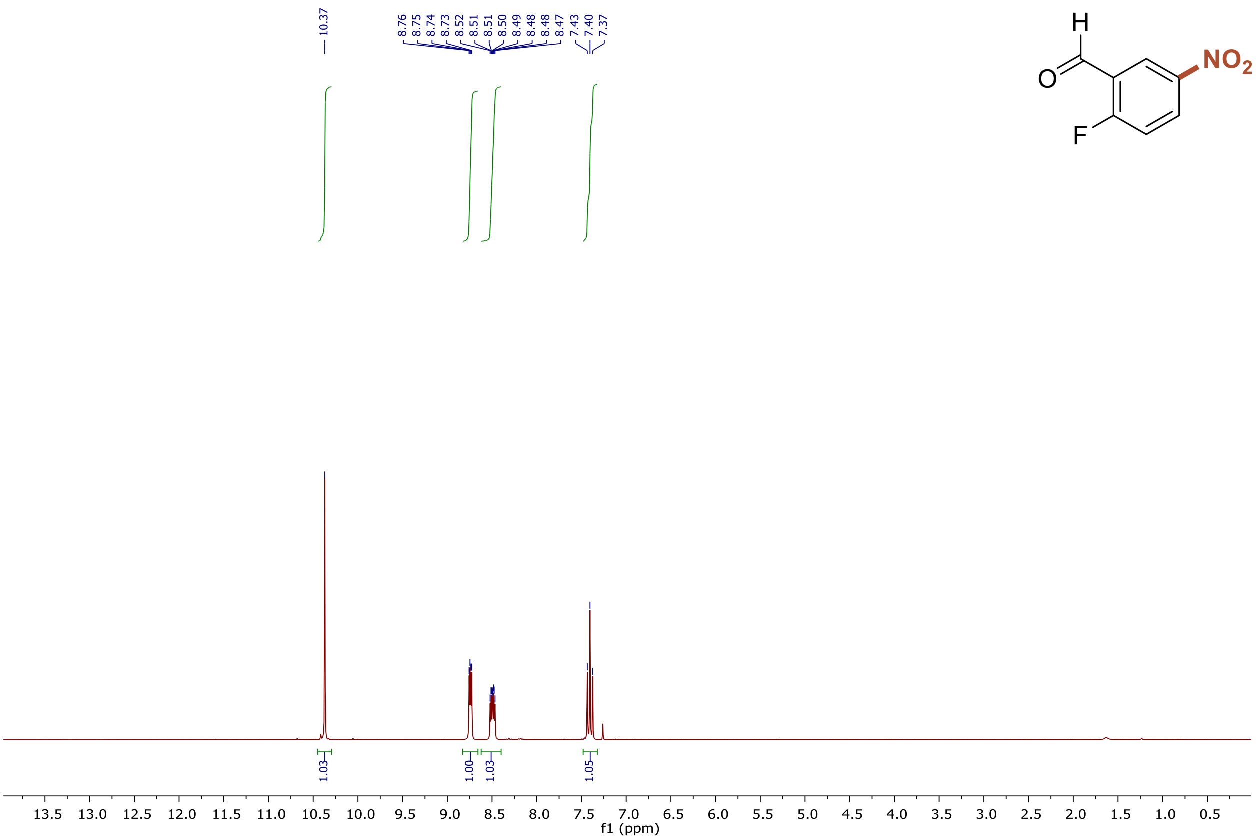

Supplementary Figure 110. ^{1}H NMR spectra for **25**.

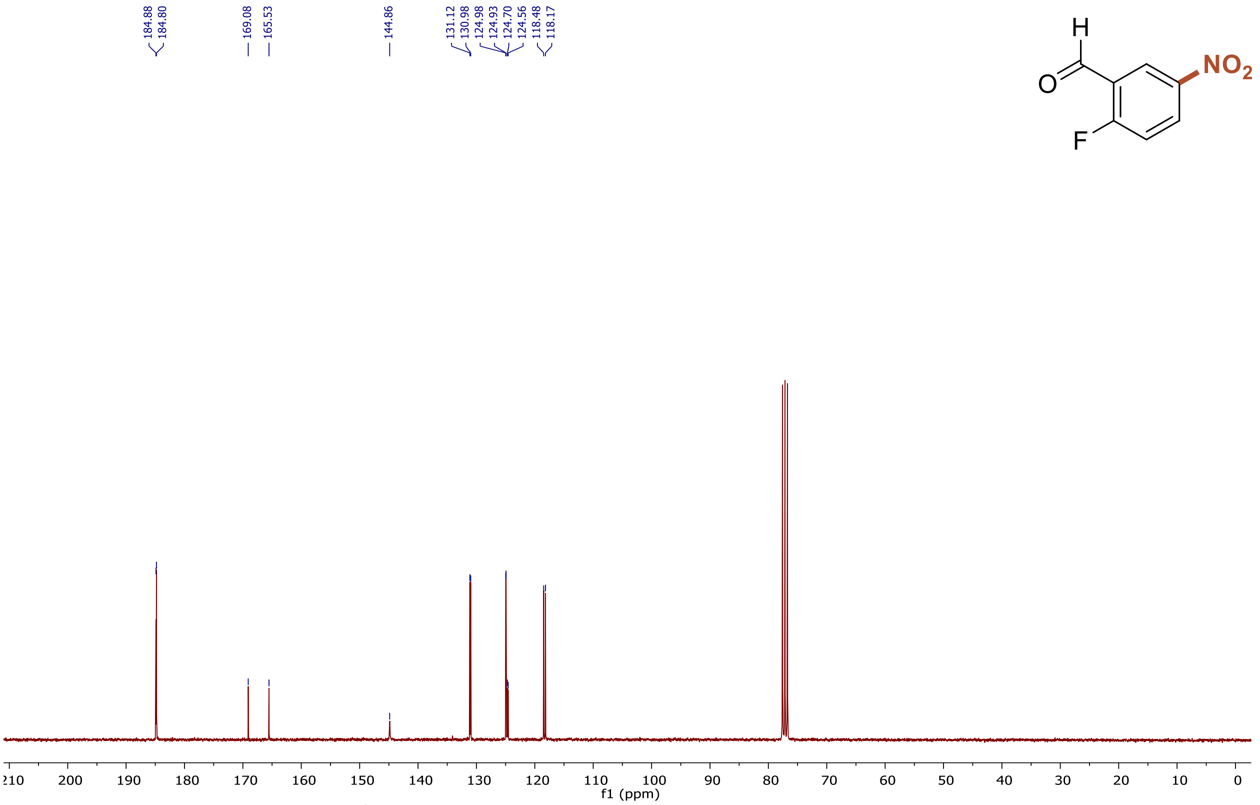

Supplementary Figure 111. ^{13}C NMR spectra for **25**.

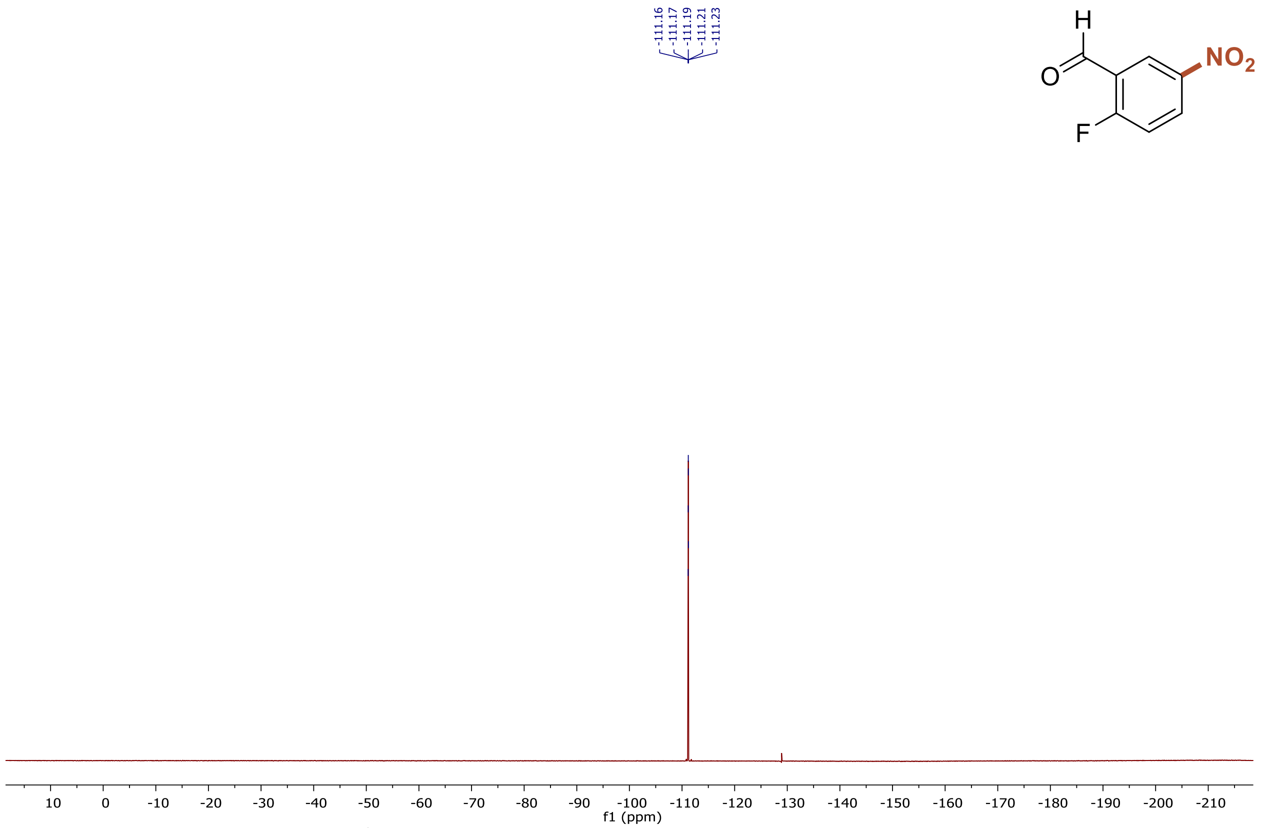

Supplementary Figure 112. ^{19}F NMR spectra for **25**.

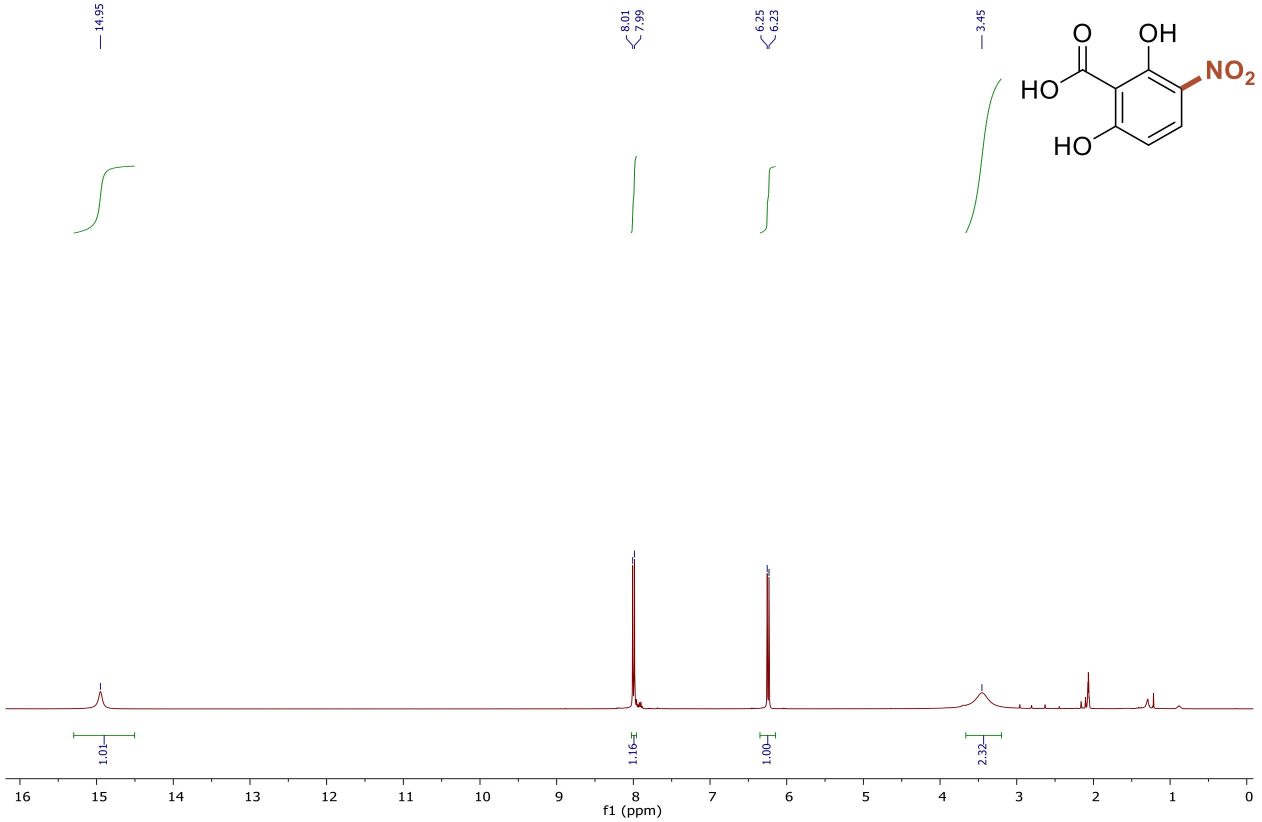

Supplementary Figure 113. ^{1}H NMR spectra for **26**.

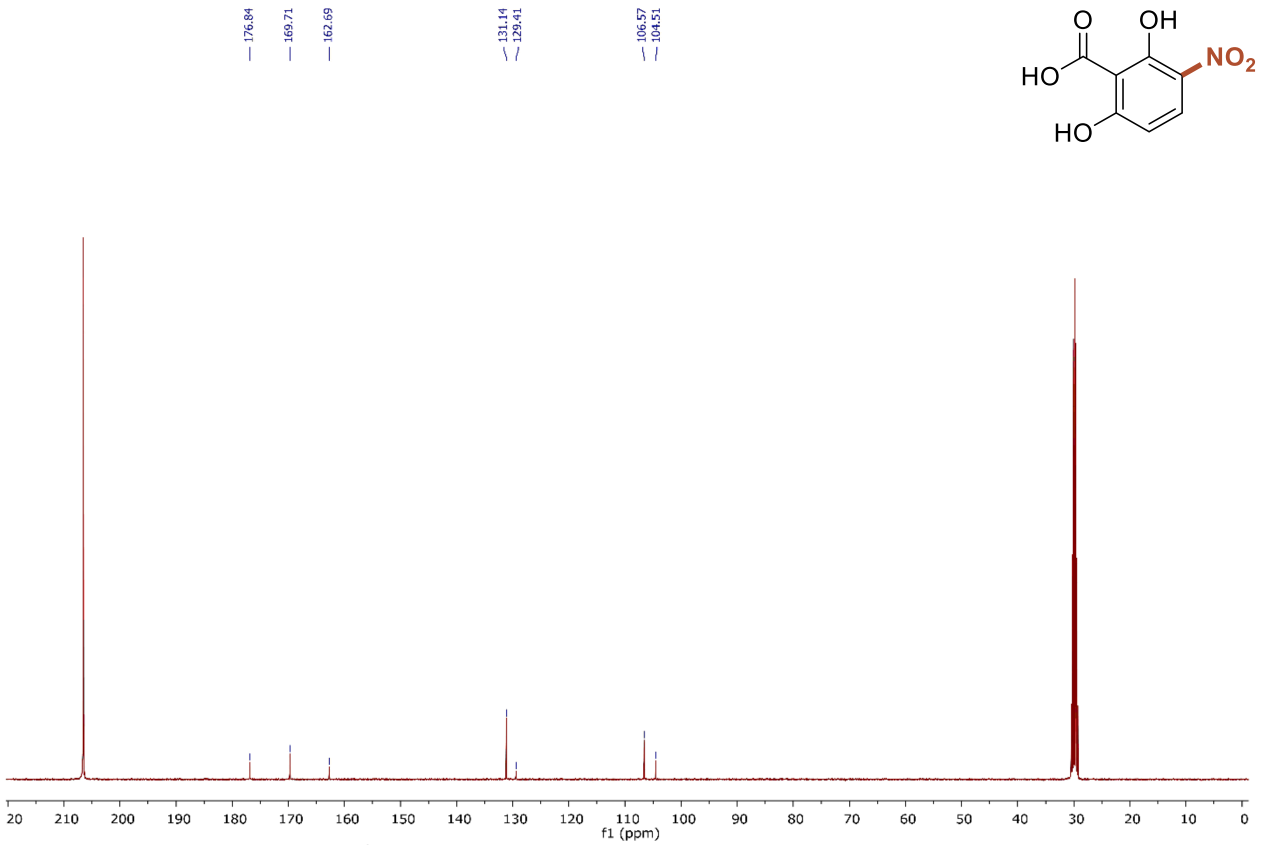

Supplementary Figure 114. ^{13}C NMR spectra for **26**.

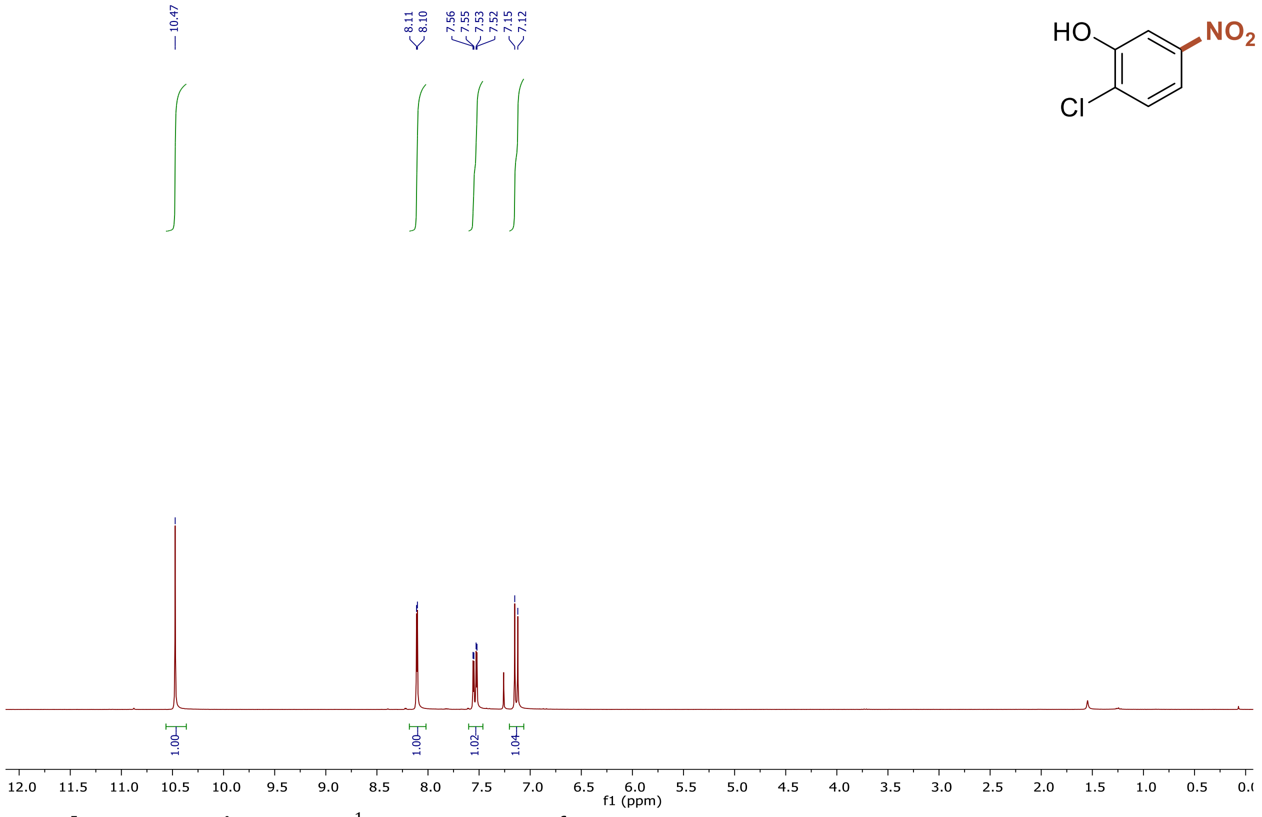

Supplementary Figure 115. ^{1}H NMR spectra for **27**.

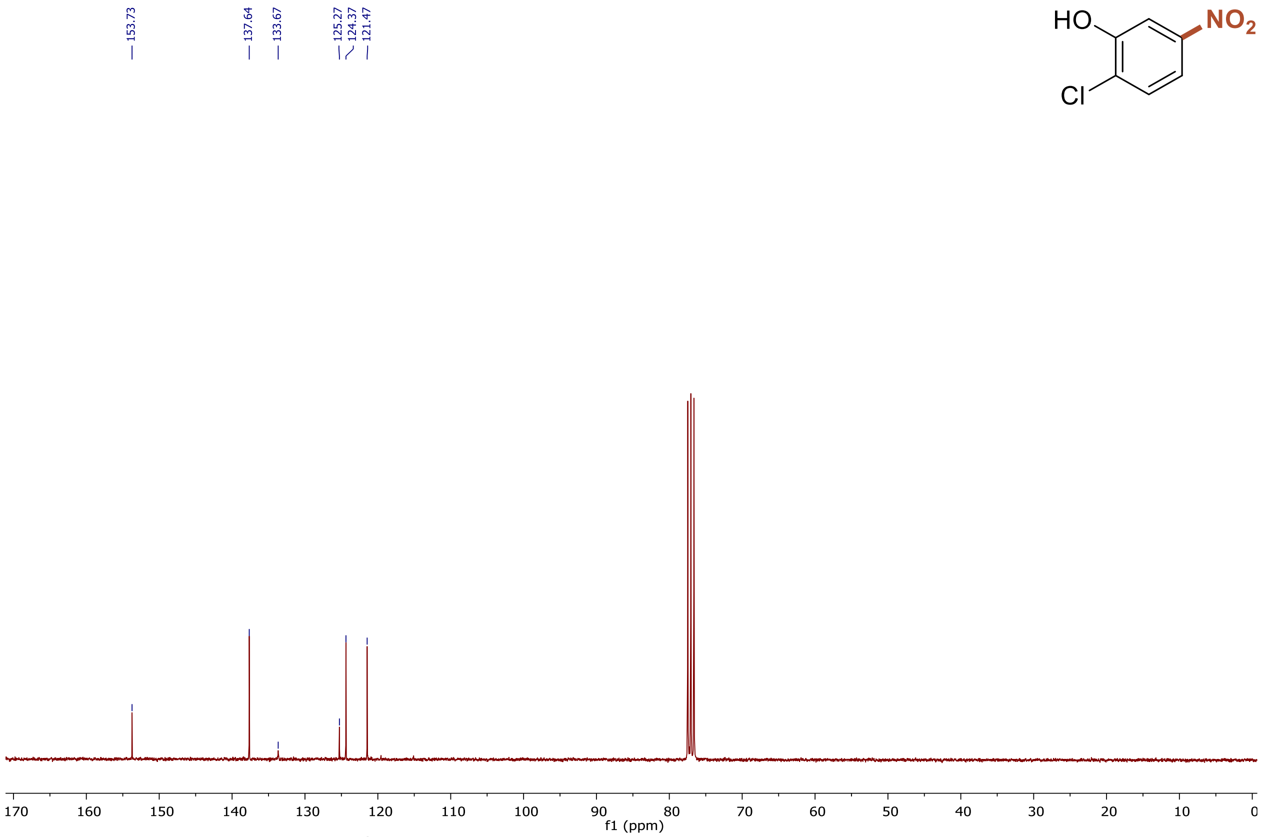

Supplementary Figure 116. ^{13}C NMR spectra for **27**.

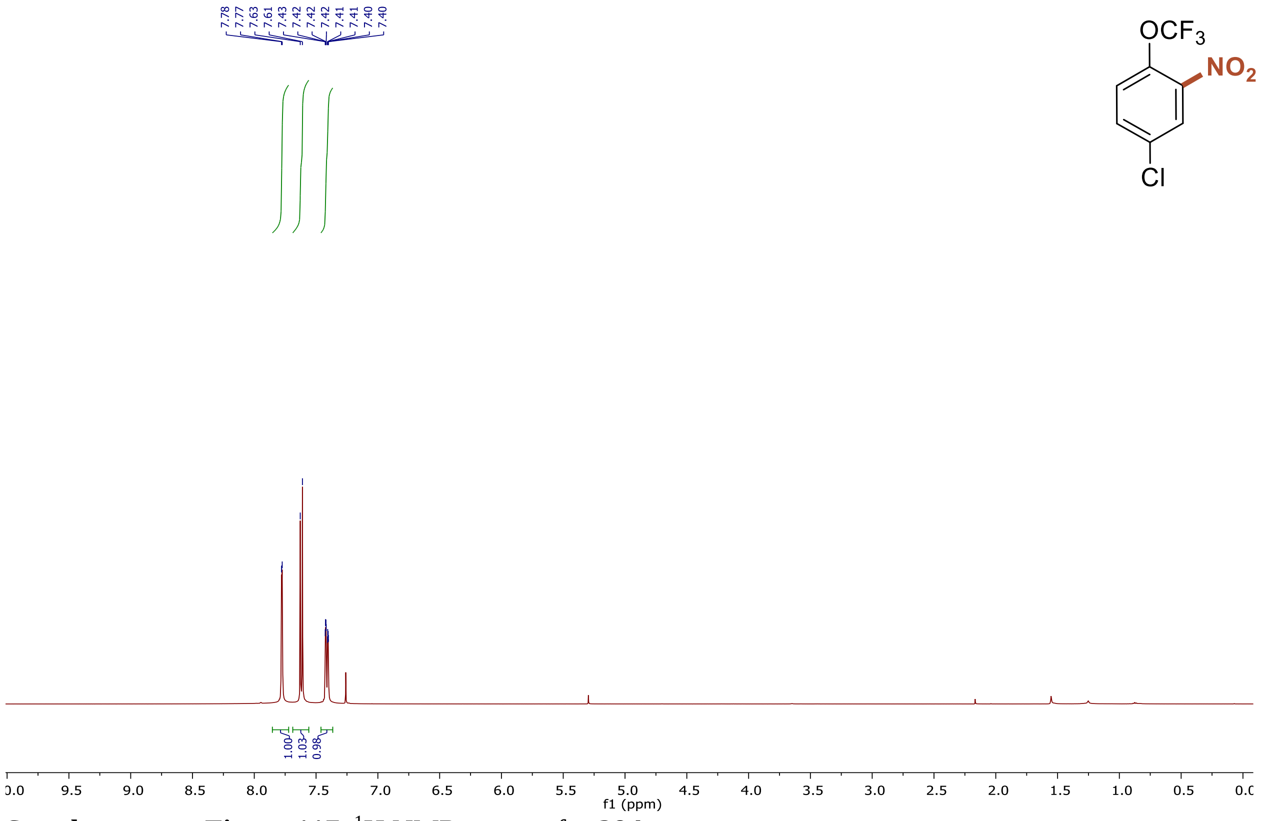

Supplementary Figure 117. ^{1}H NMR spectra for **28A**.

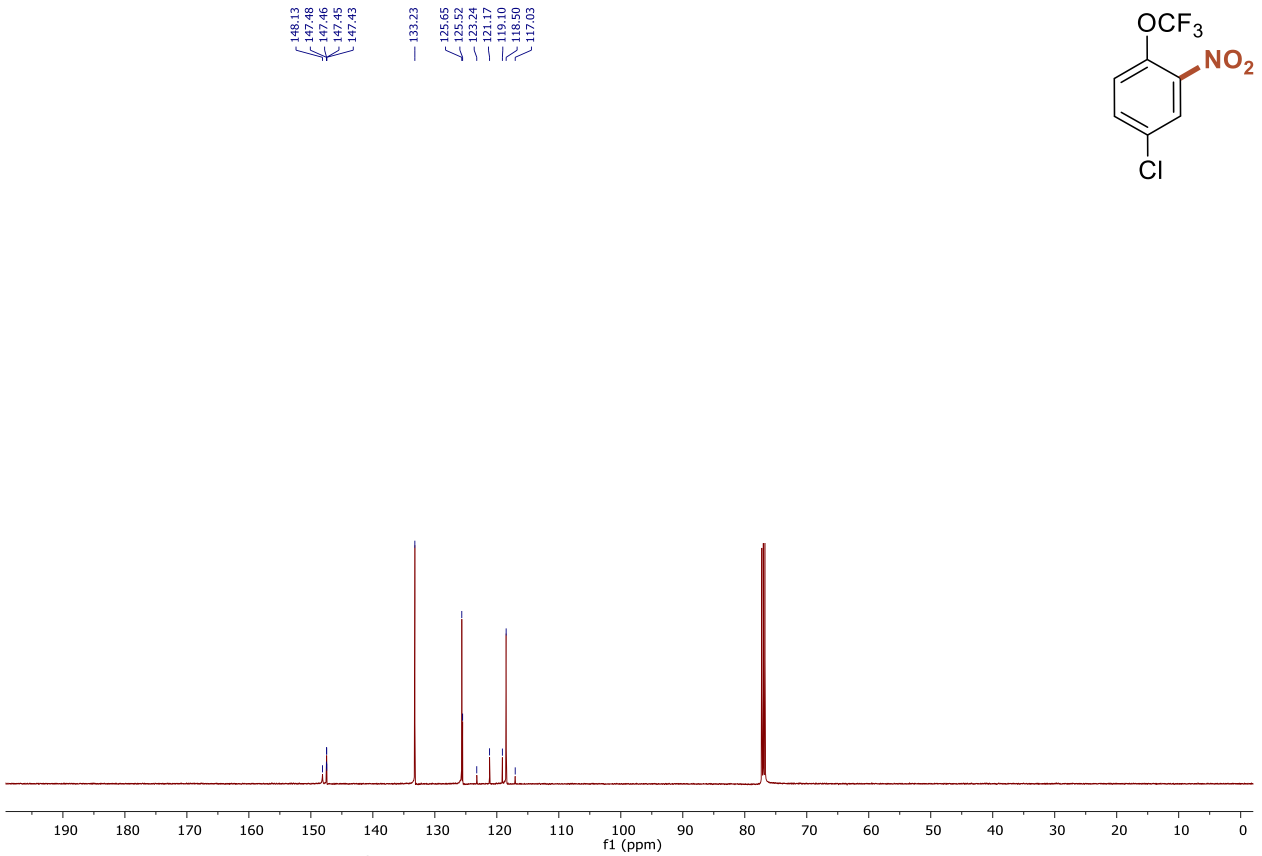

Supplementary Figure 118. ^{13}C NMR spectra for **28A**.

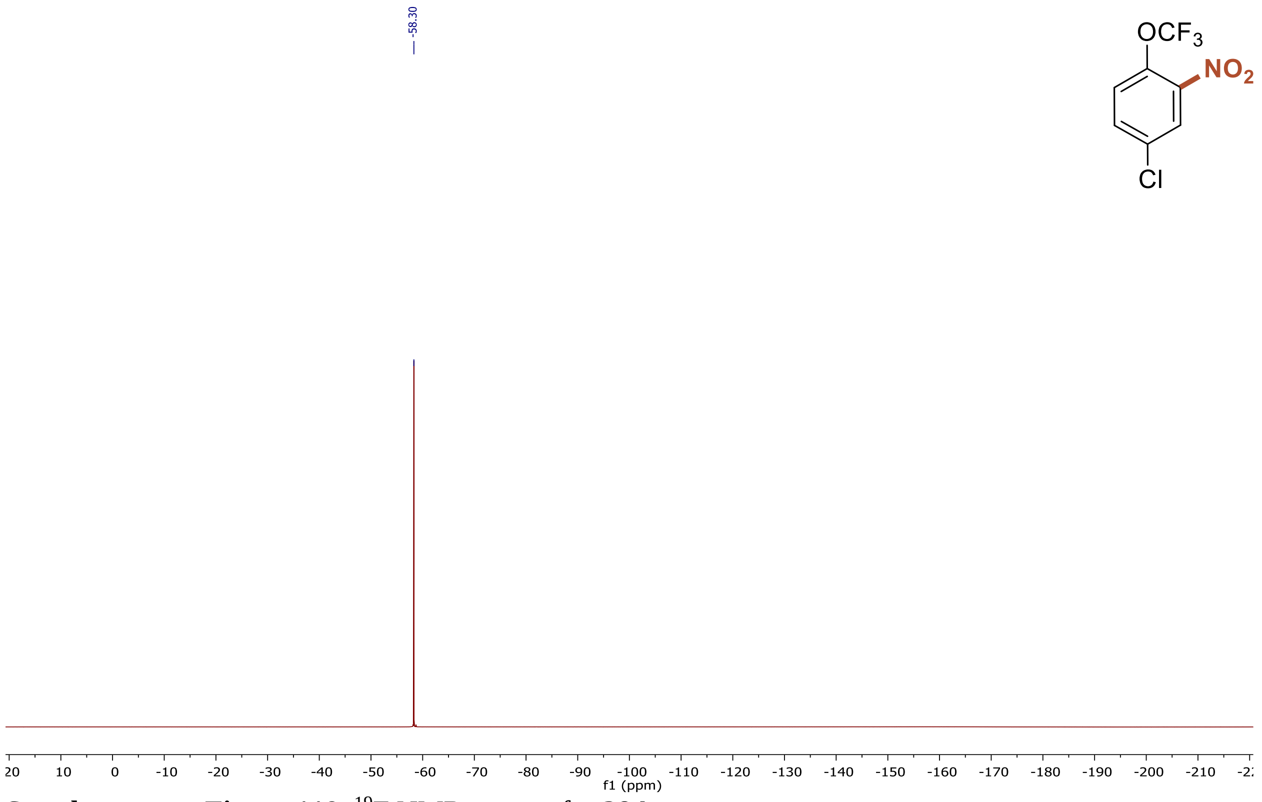

Supplementary Figure 119. ^{19}F NMR spectra for **28A**.

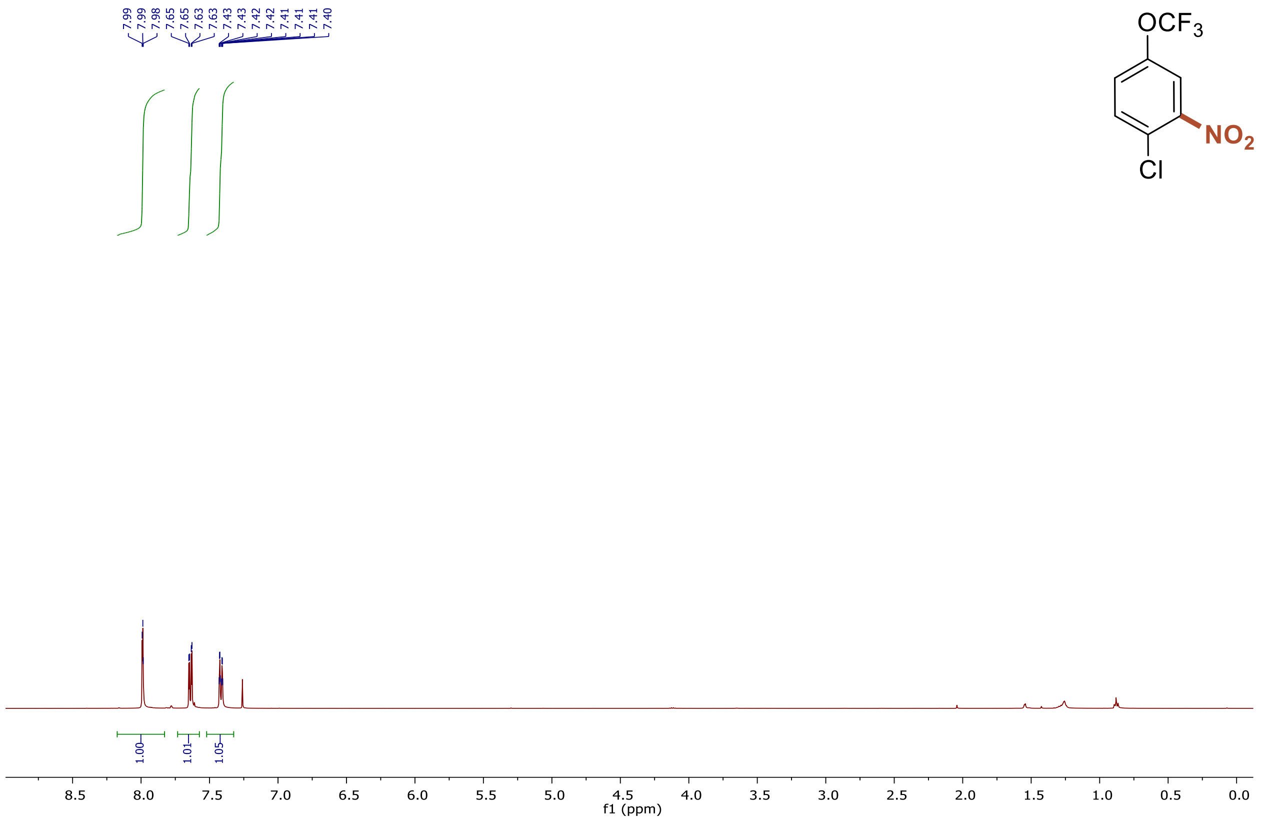

Supplementary Figure 120. ^{1}H NMR spectra for **28B**.

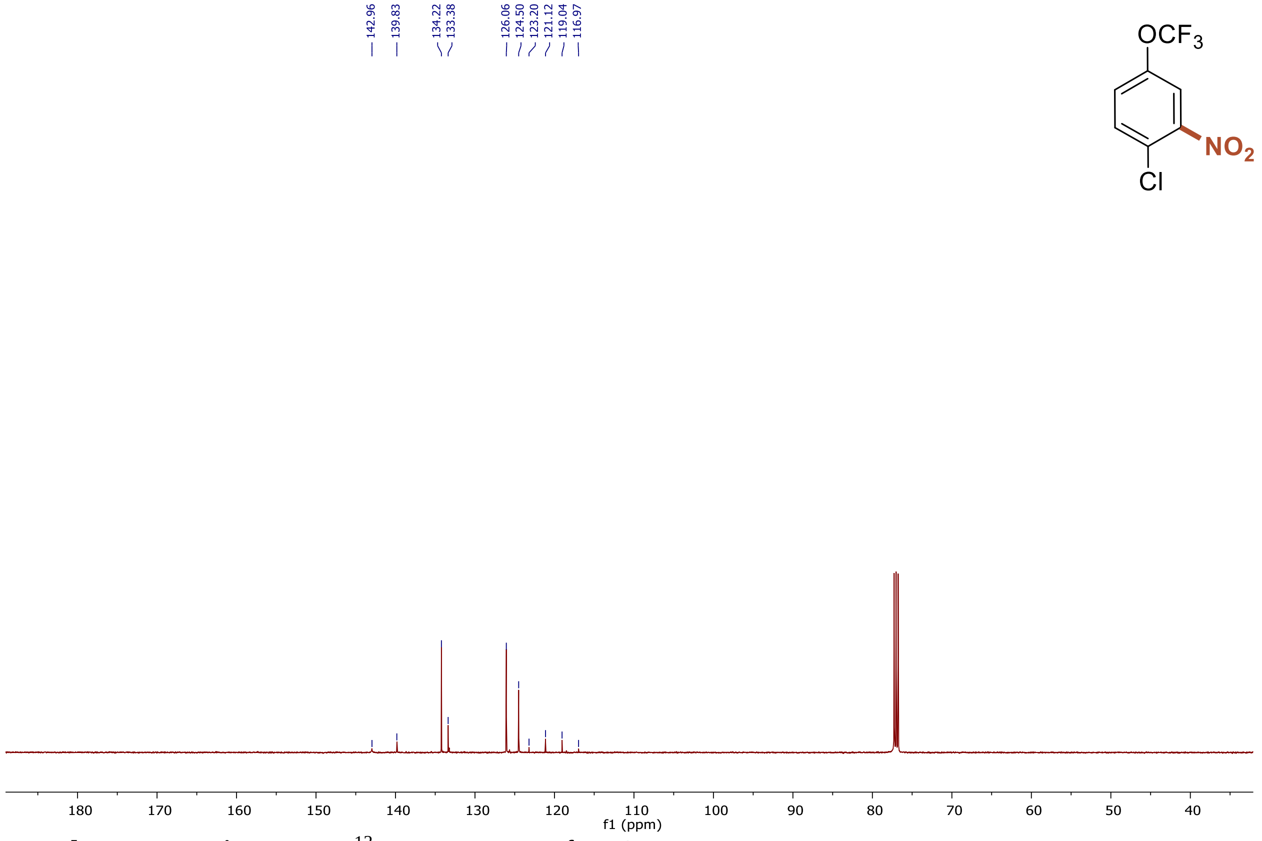

Supplementary Figure 121. ^{13}C NMR spectra for **28B**.

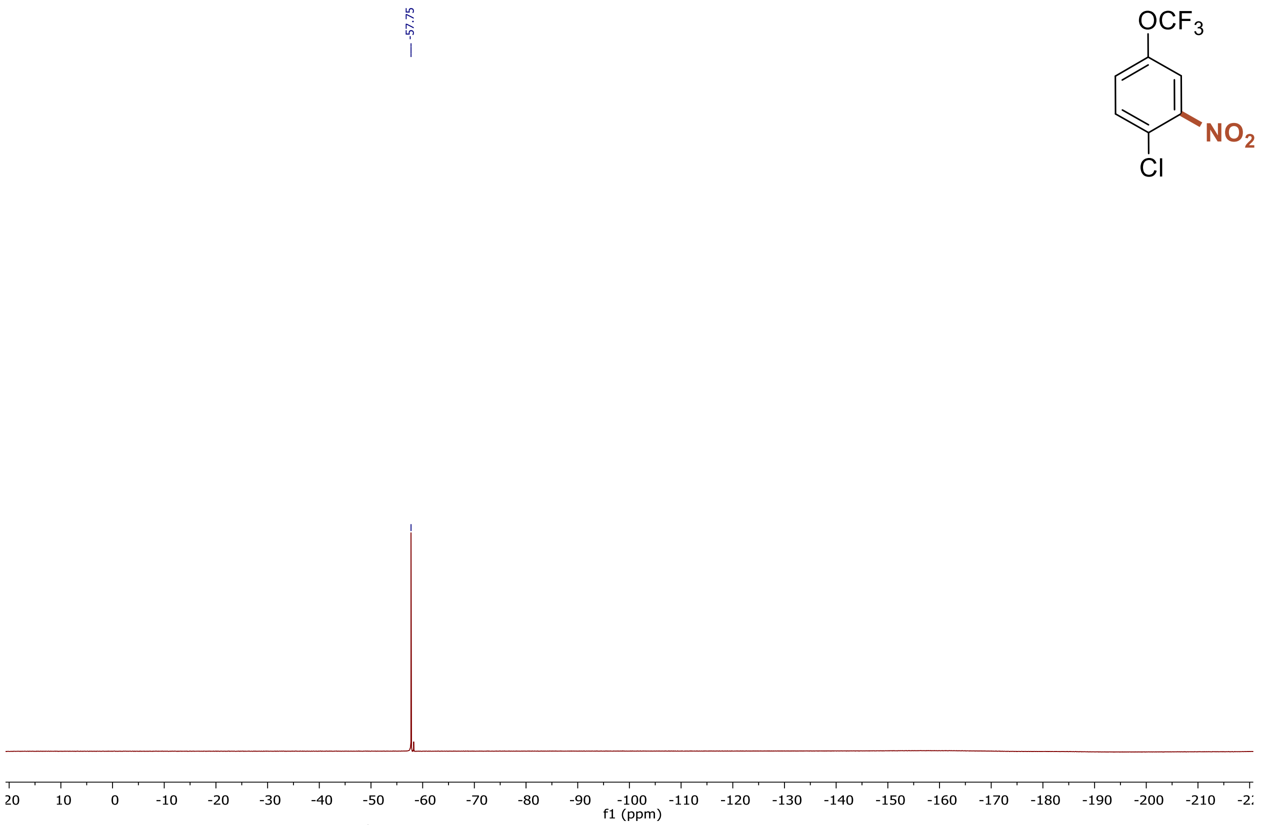

Supplementary Figure 122. ^{19}F NMR spectra for **28B**.

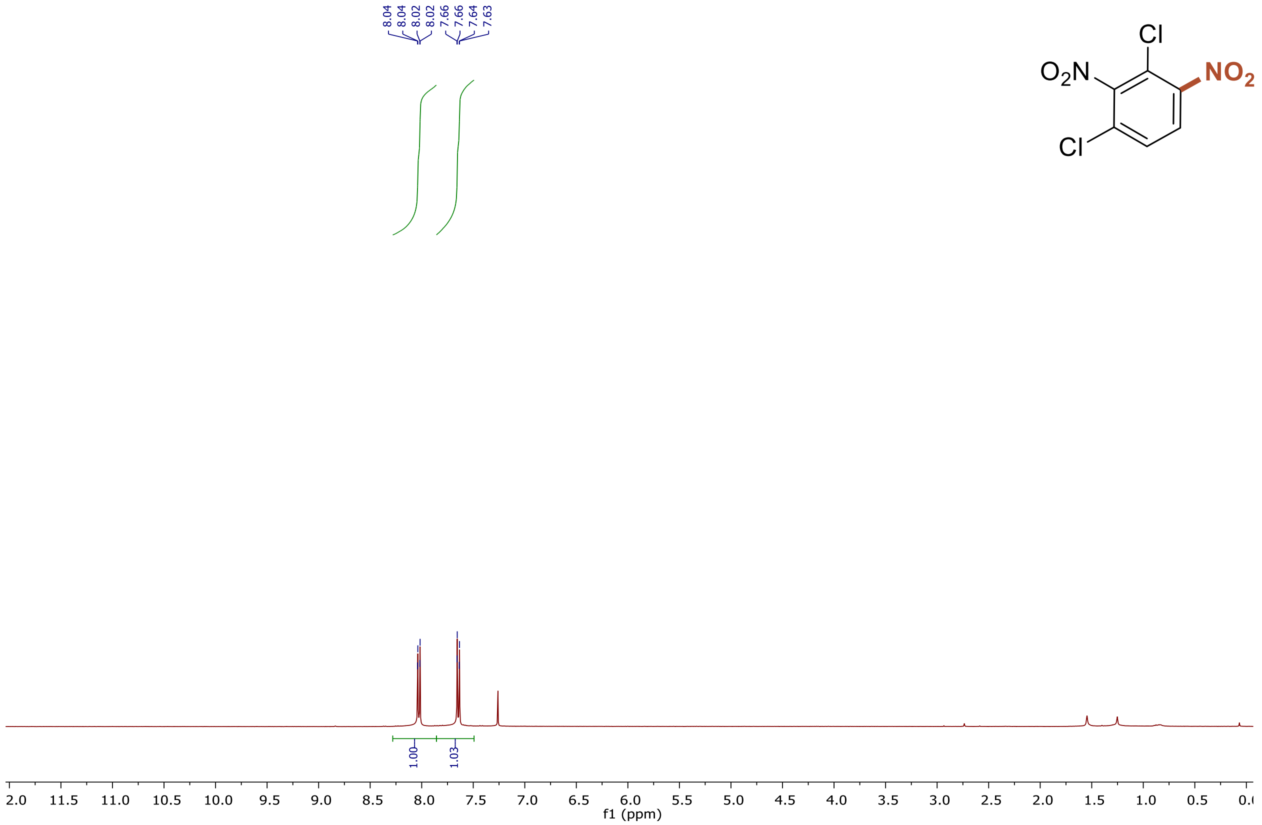

Supplementary Figure 123. ^{1}H NMR spectra for **29**.

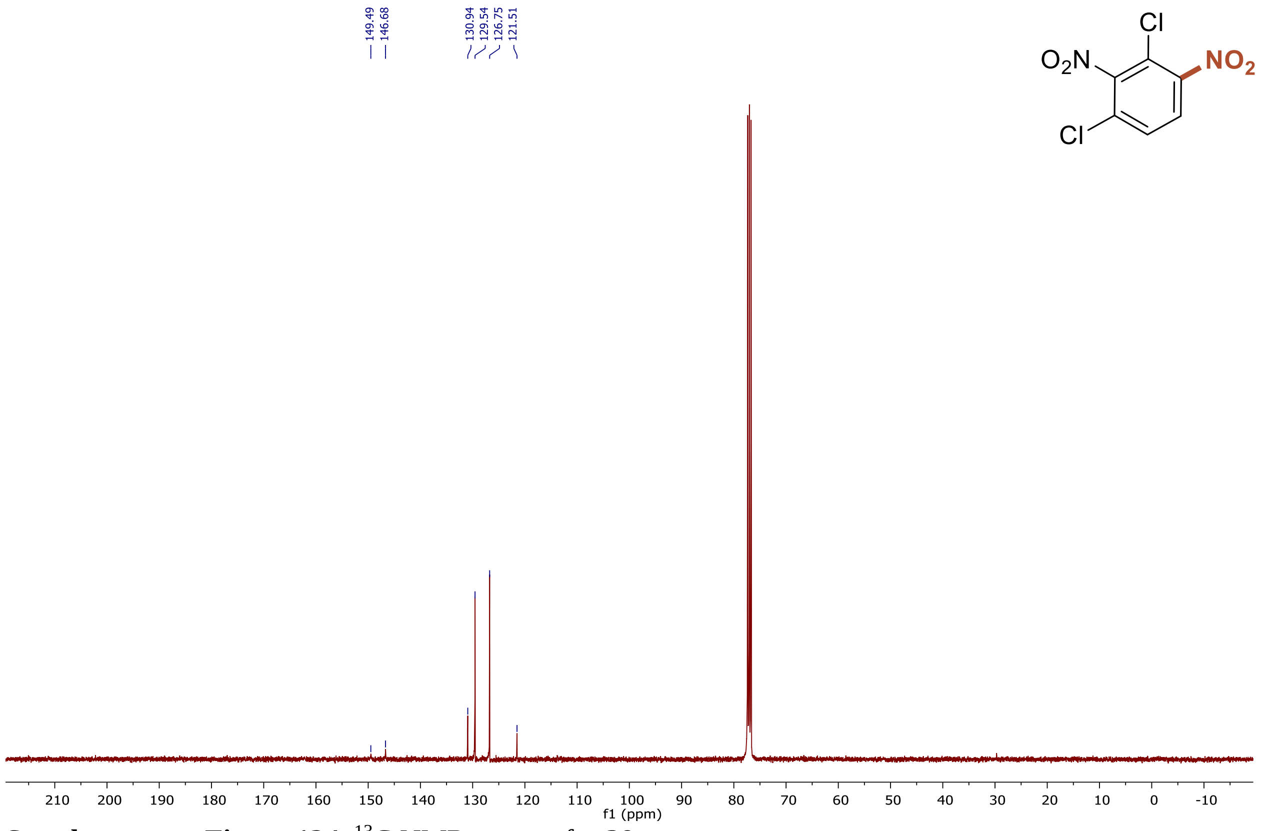

Supplementary Figure 124. ^{13}C NMR spectra for **29**.

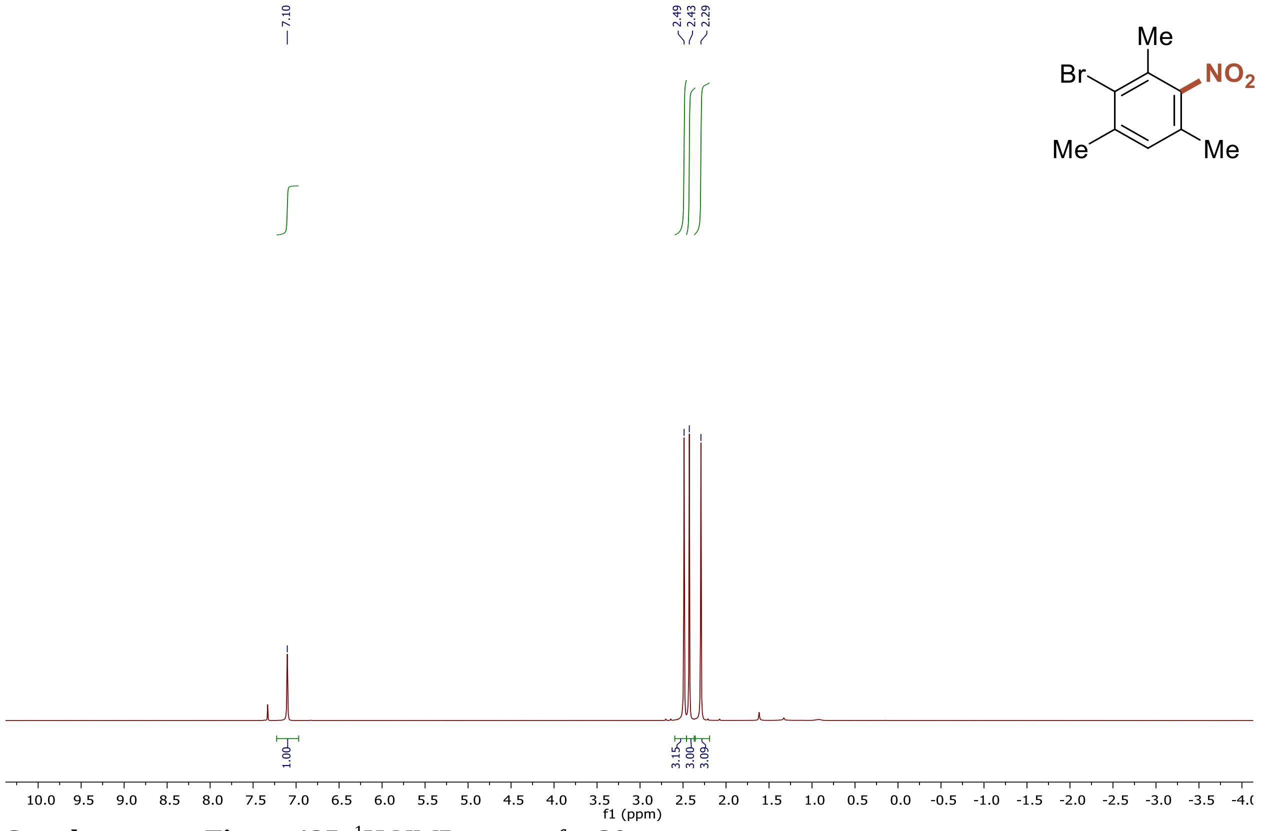

Supplementary Figure 125. ^{1}H NMR spectra for **30**.

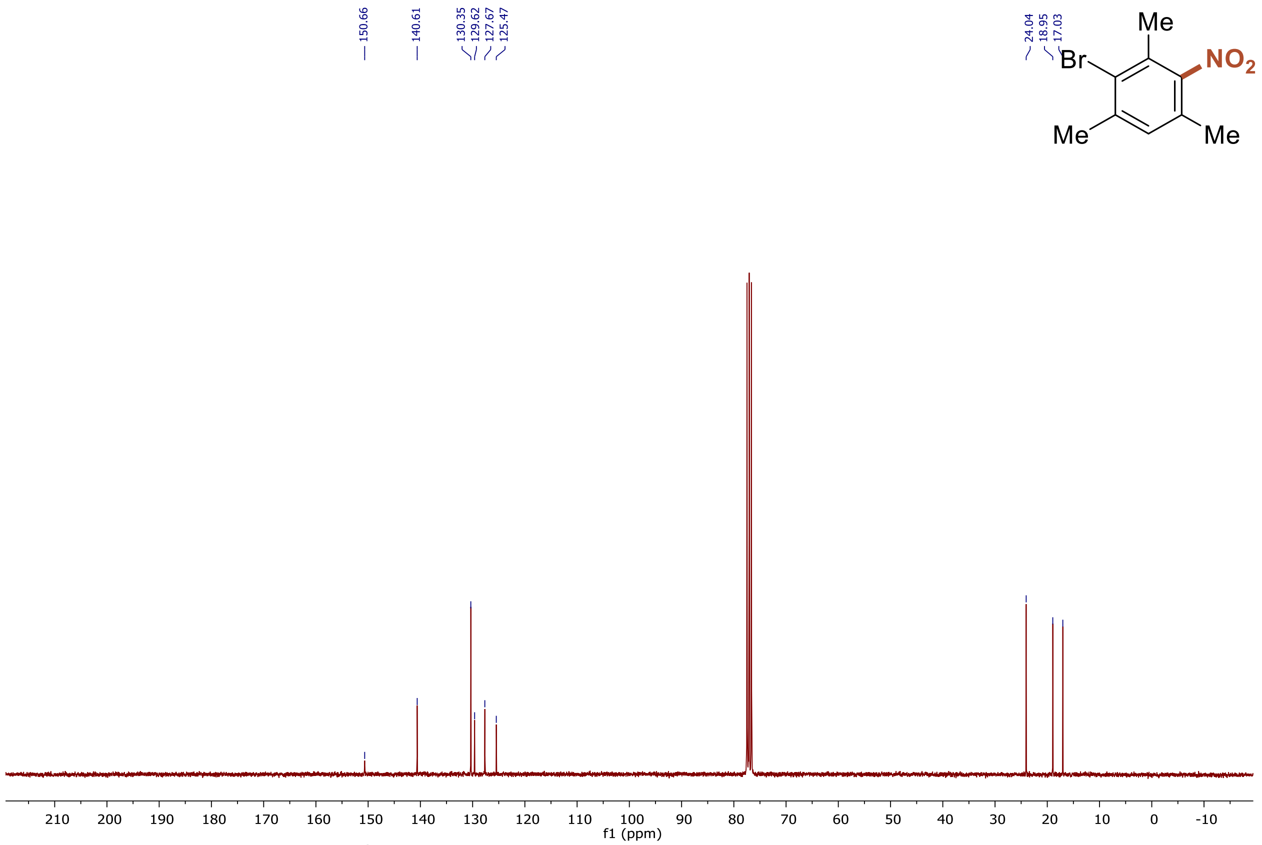

Supplementary Figure 126. ^{13}C NMR spectra for **30**.

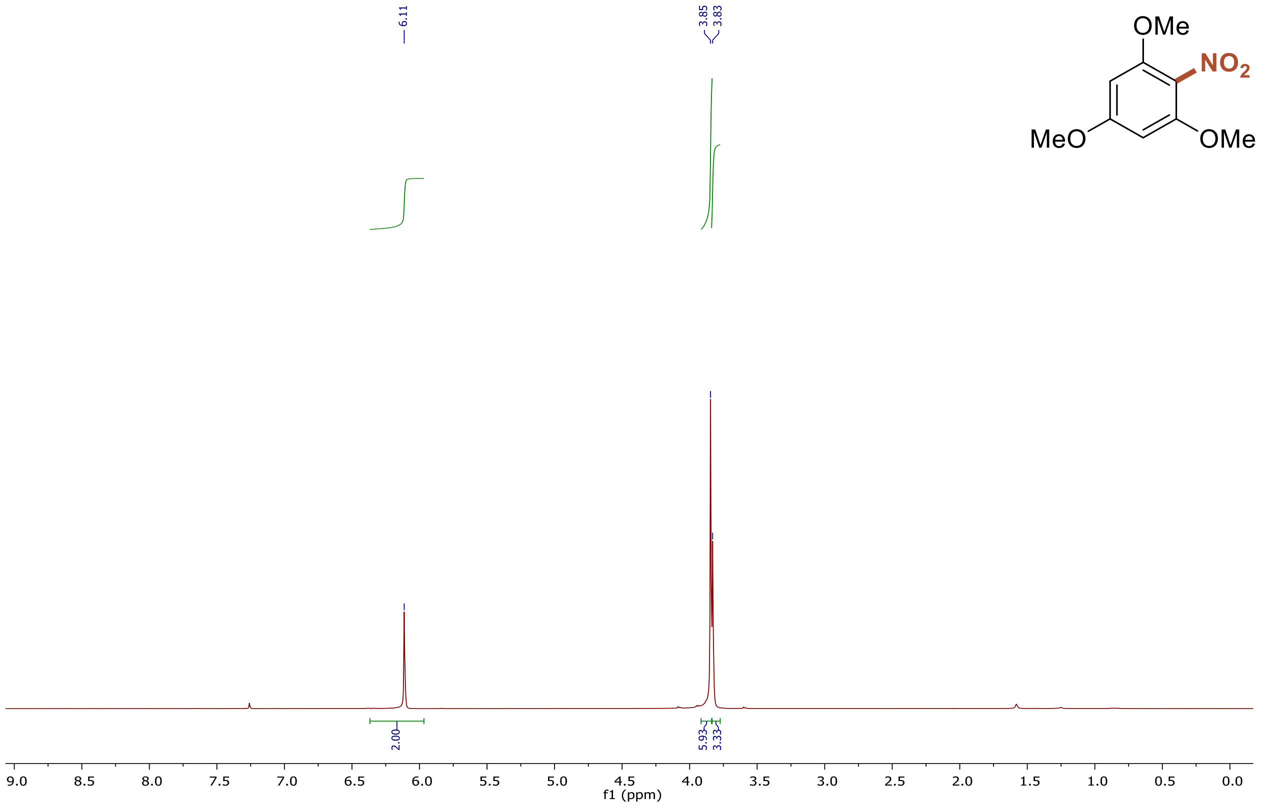

Supplementary Figure 127. ^{1}H NMR spectra for **31**.

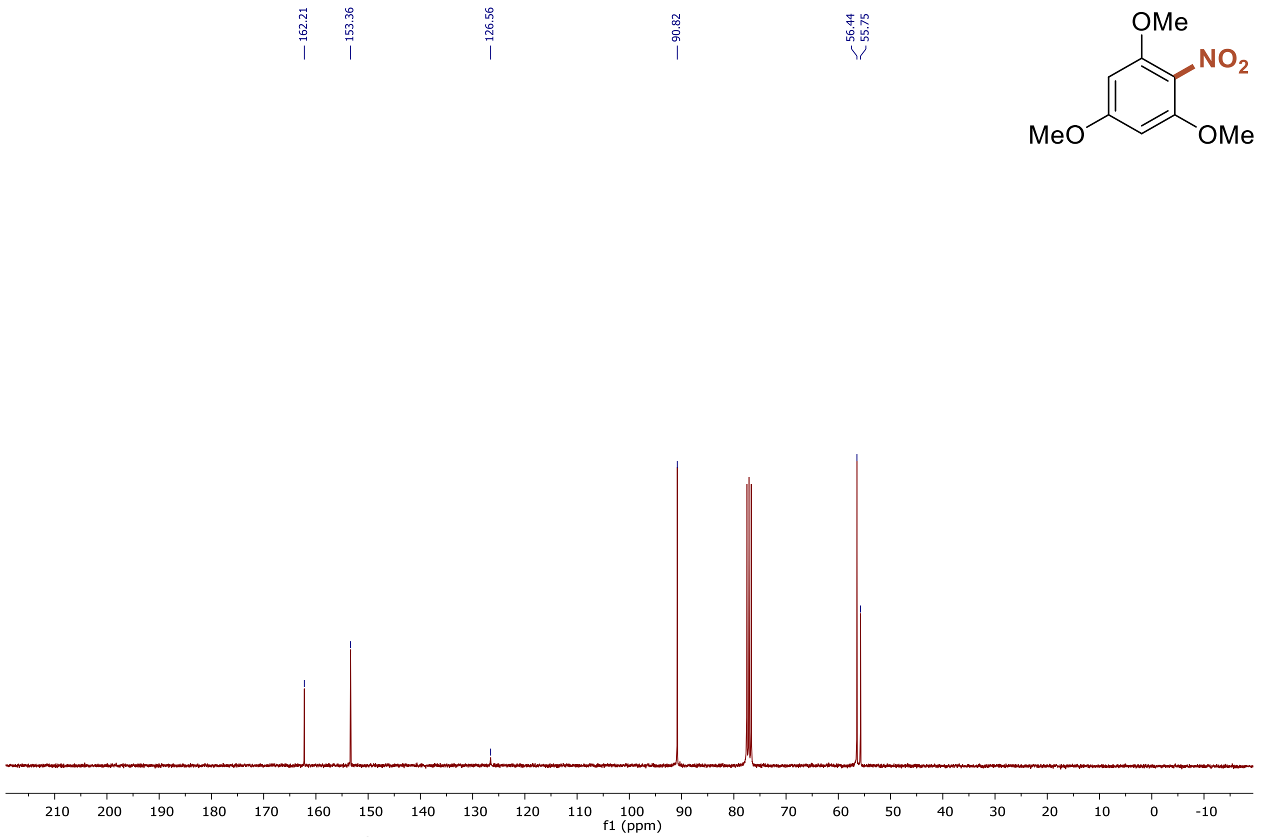

Supplementary Figure 128. ^{13}C NMR spectra for **31**.

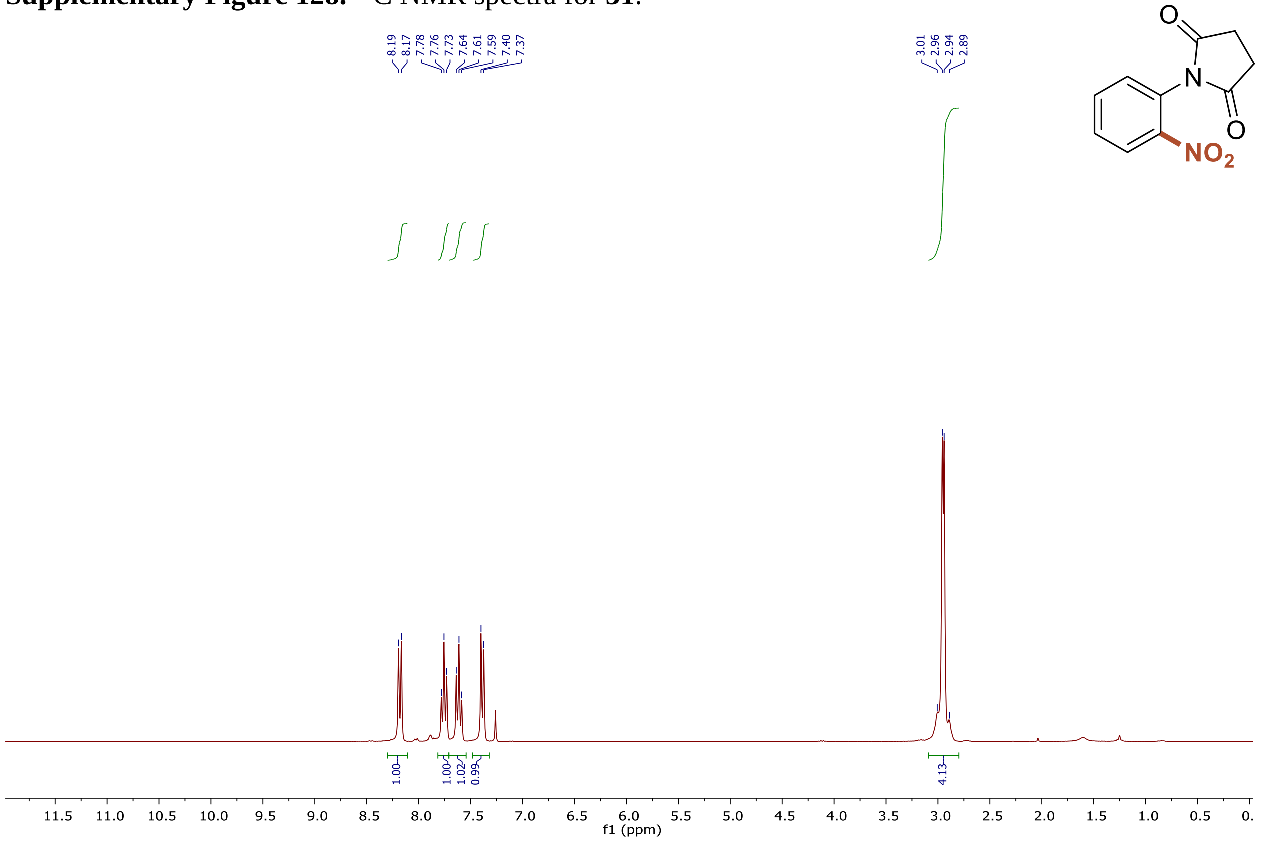

Supplementary Figure 129. ^{1}H NMR spectra for **32A**.

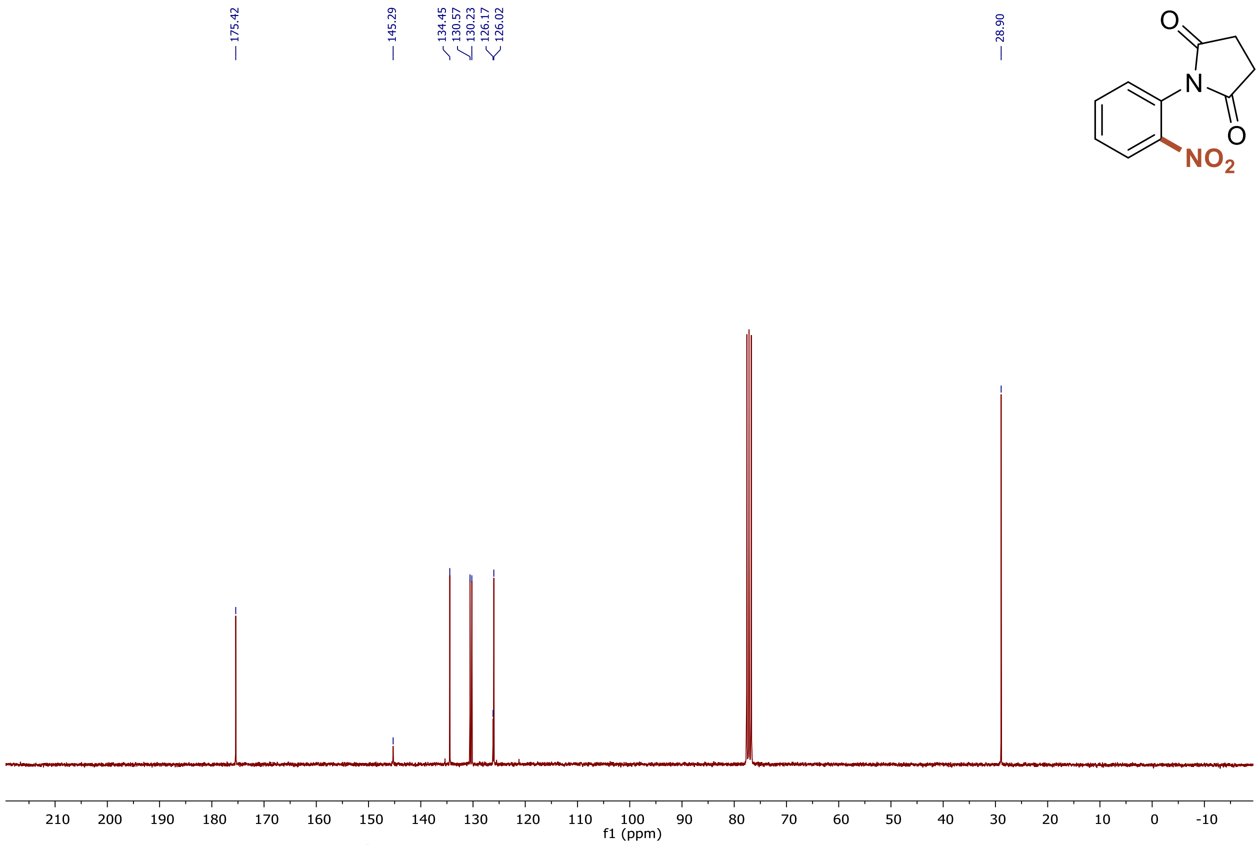

Supplementary Figure 130. ^{13}C NMR spectra for **32A**.

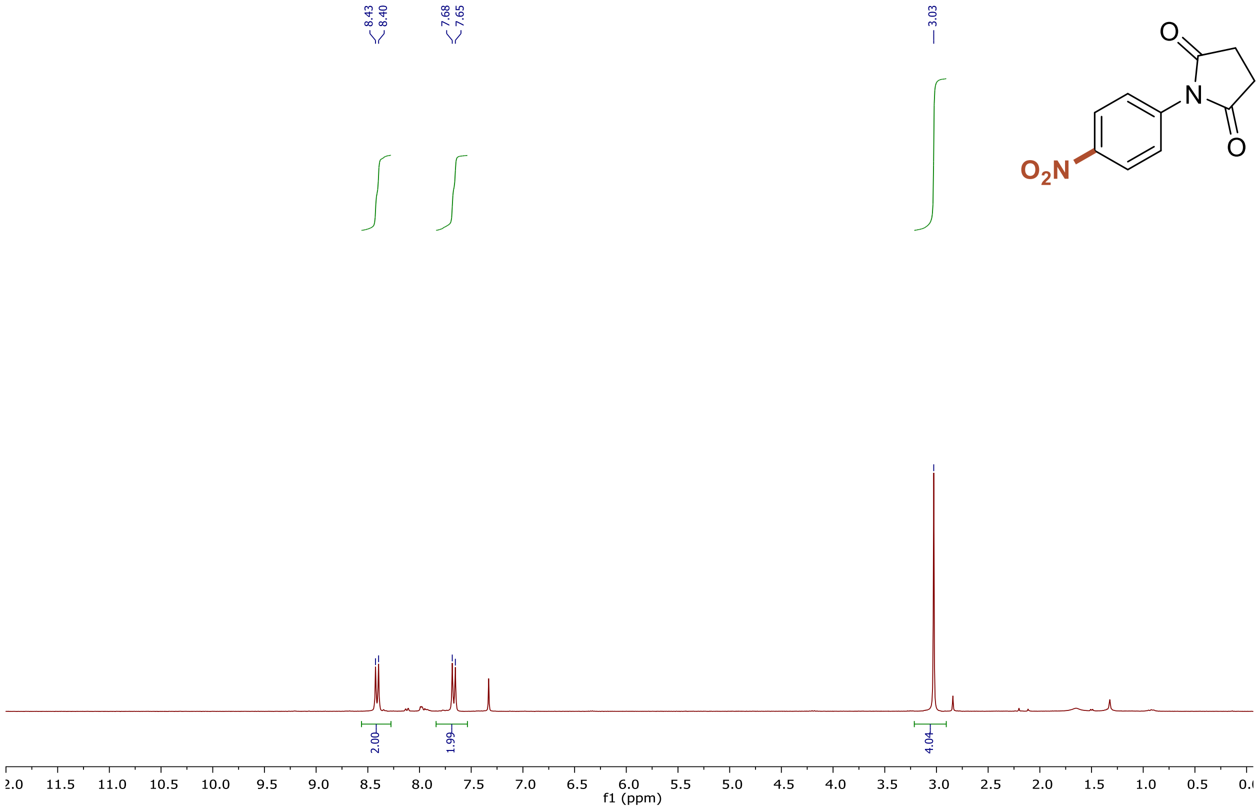

Supplementary Figure 131. ^{1}H NMR spectra for **32B**.

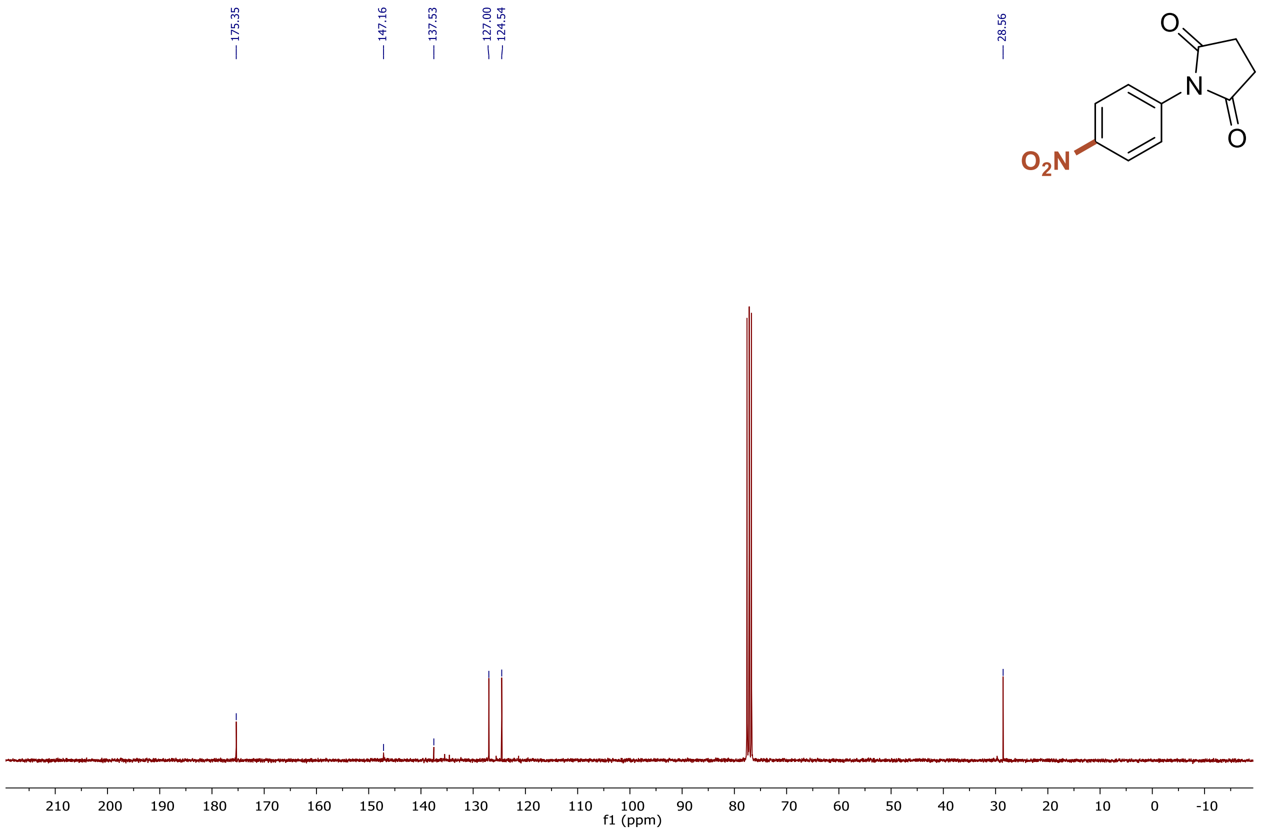

Supplementary Figure 132. ^{13}C NMR spectra for **32B**.

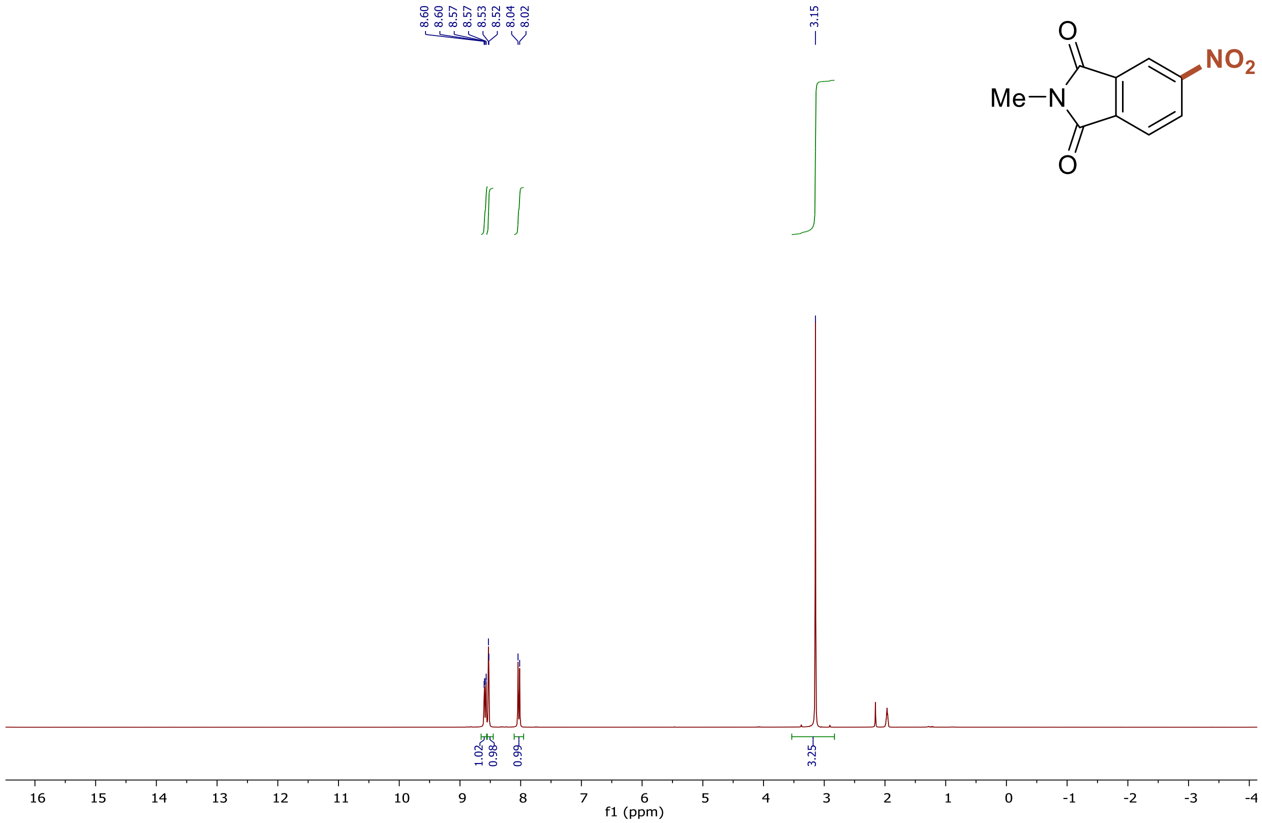

Supplementary Figure 133. ^{1}H NMR spectra for **33**.

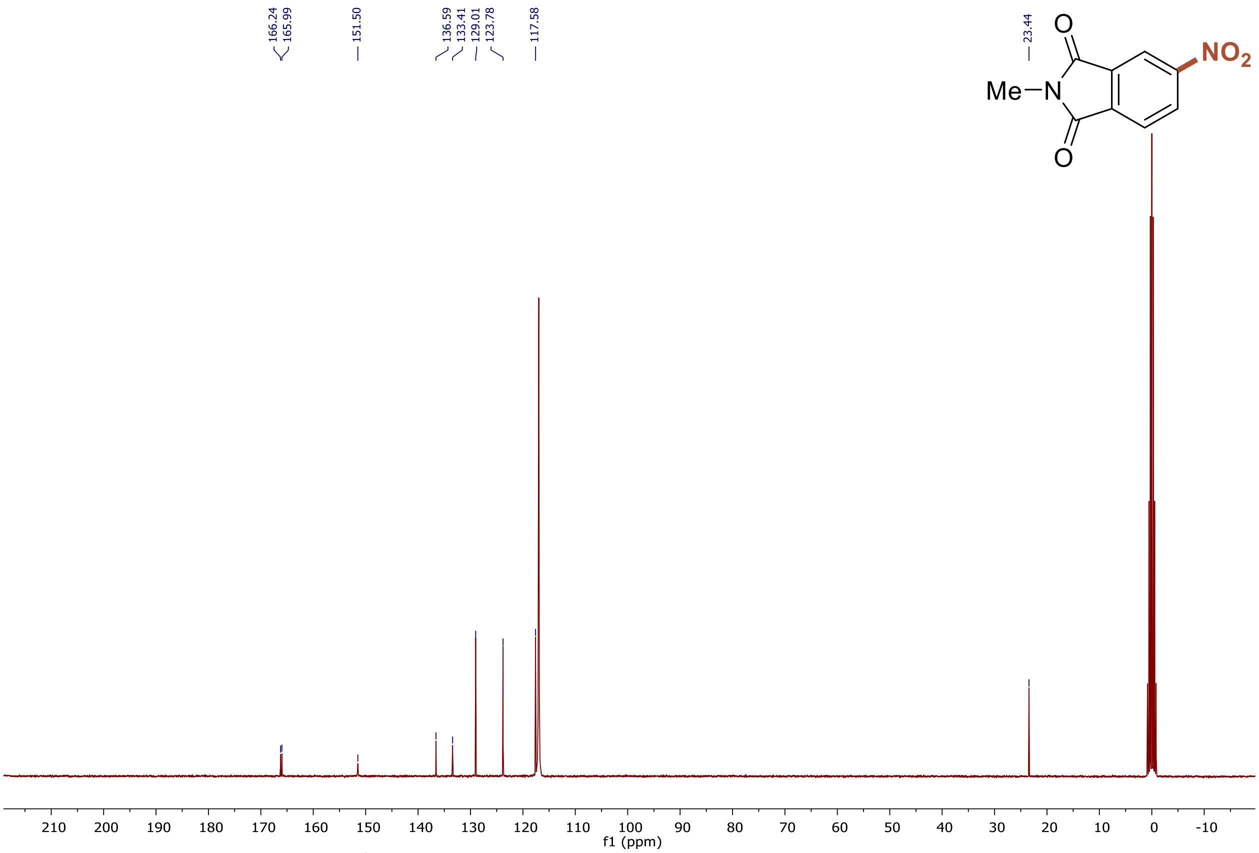

Supplementary Figure 134. ^{13}C NMR spectra for **33**.

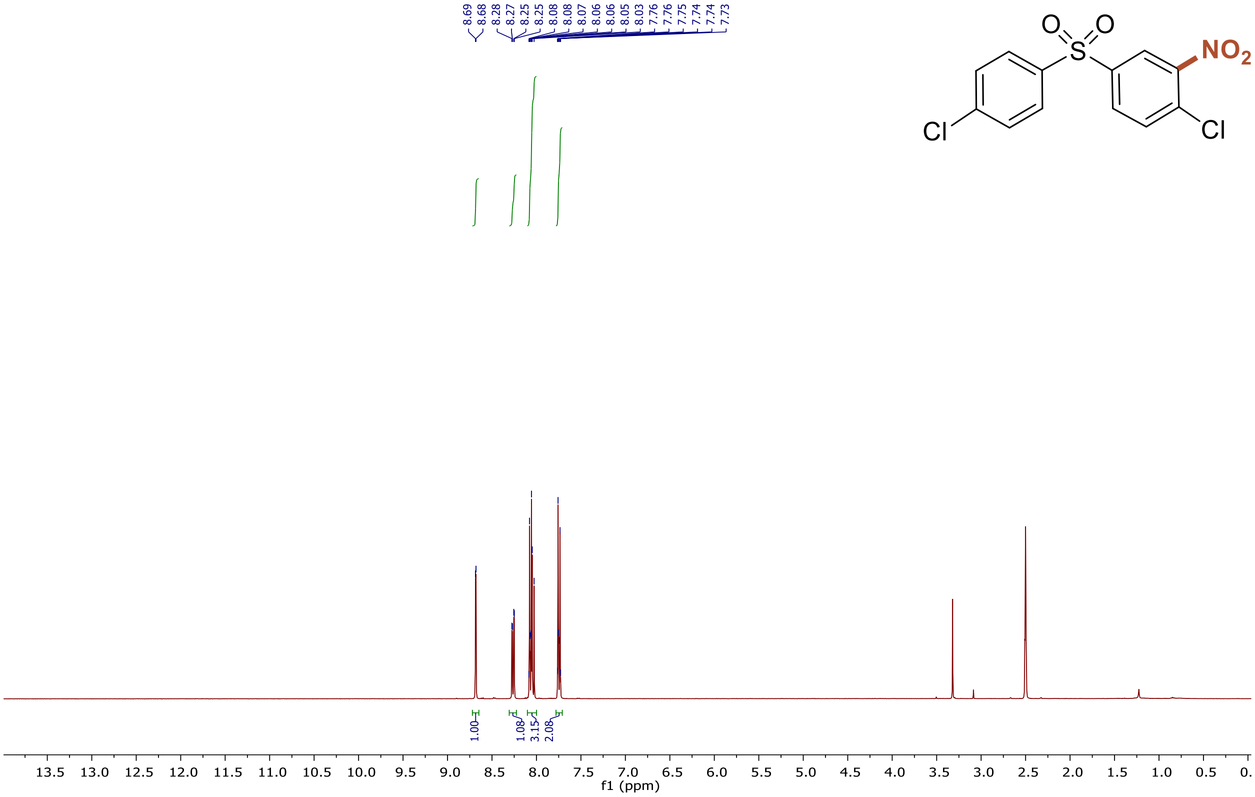

Supplementary Figure 135. ^{1}H NMR spectra for **34**.

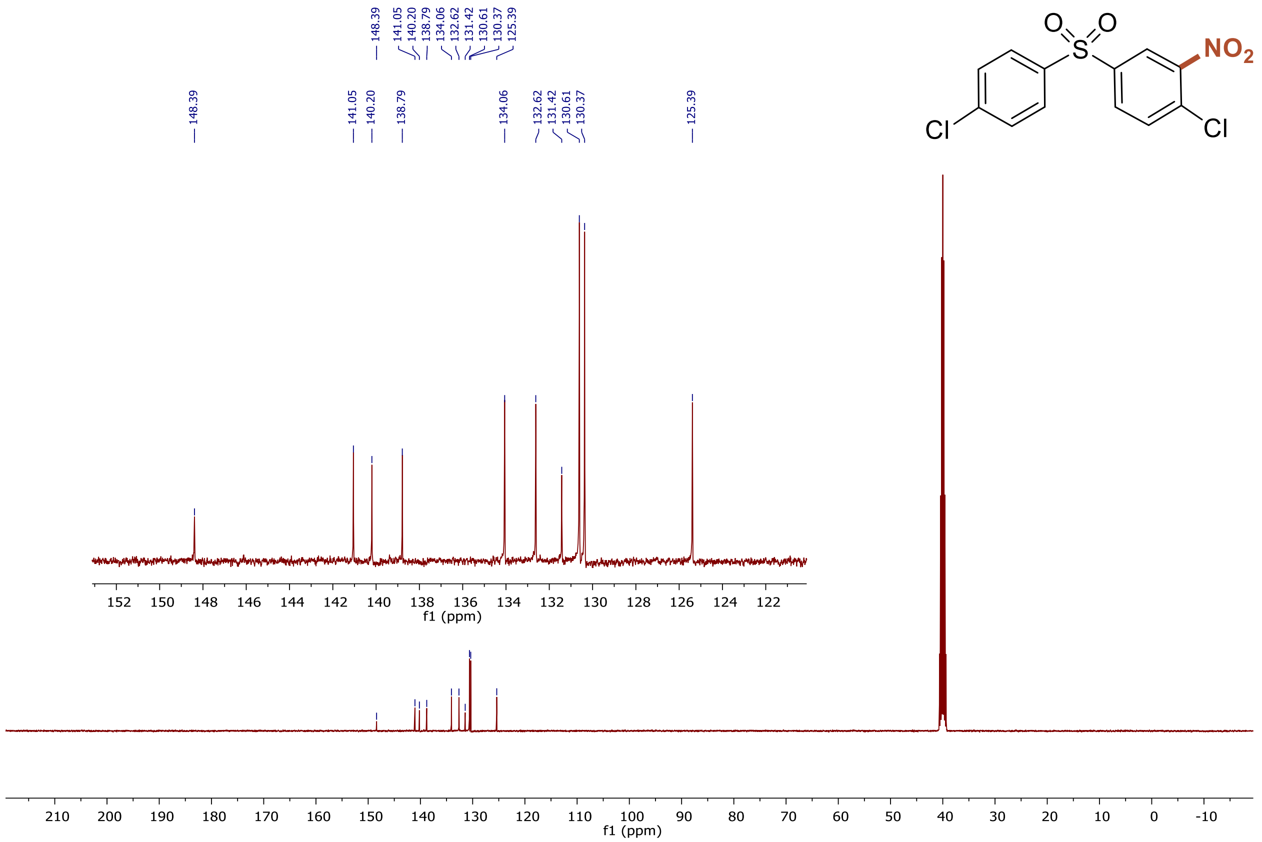

Supplementary Figure 136. ¹H NMR spectra for **34**.

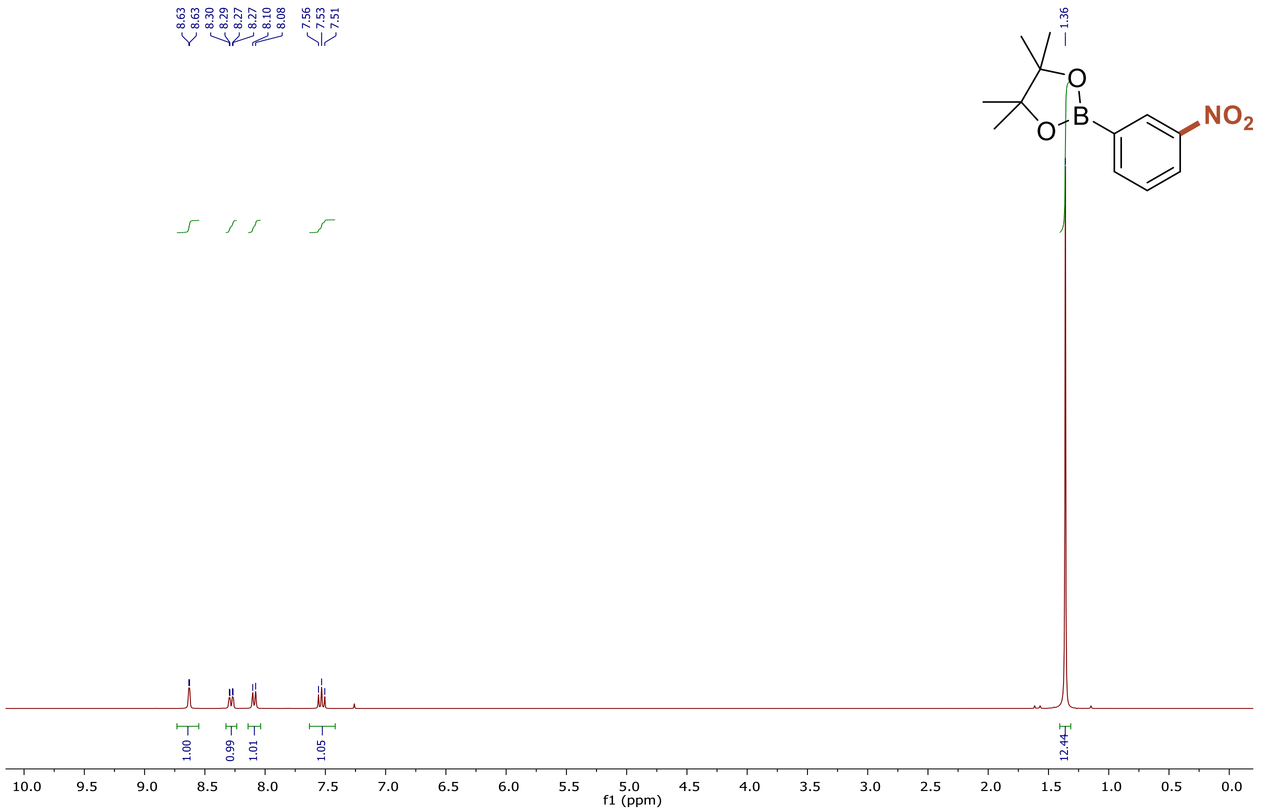

Supplementary Figure 137. ¹H NMR spectra for **35A**.

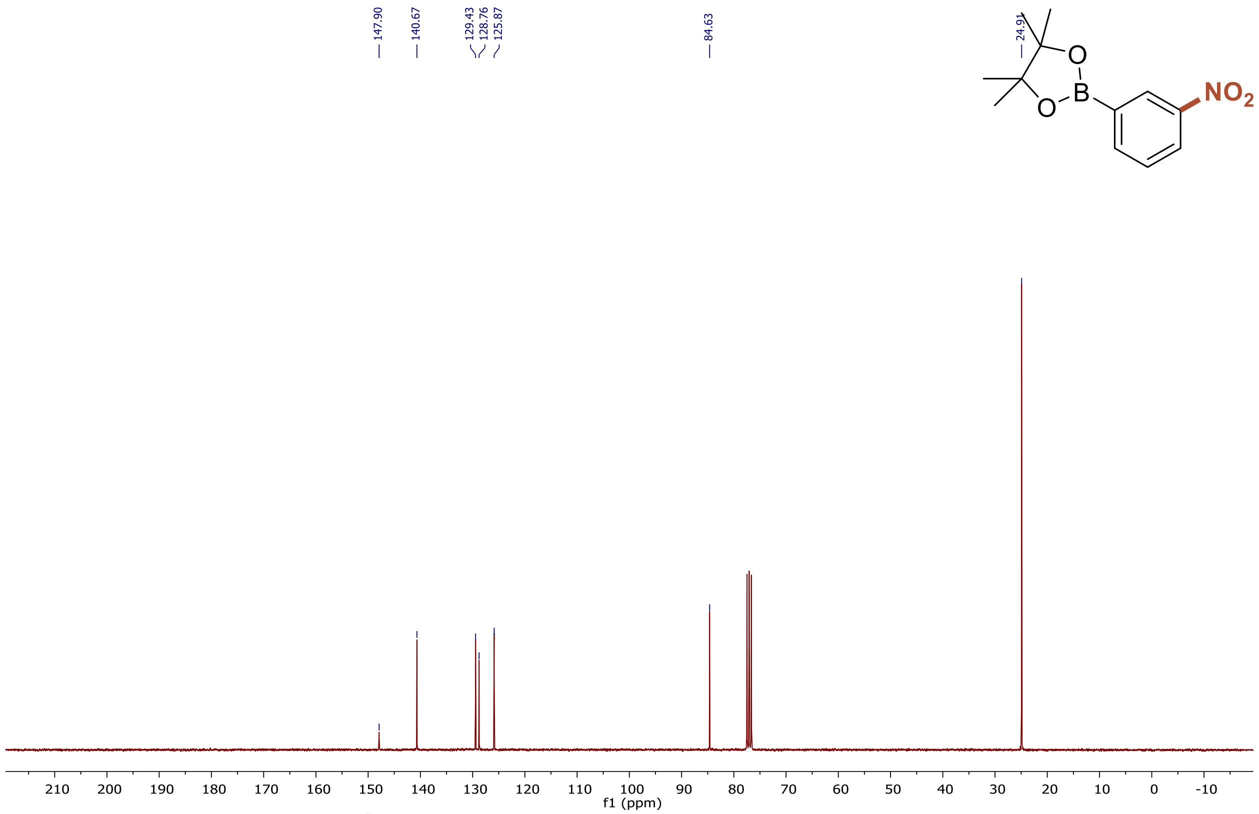

Supplementary Figure 138. ^{13}C NMR spectra for **35A**.

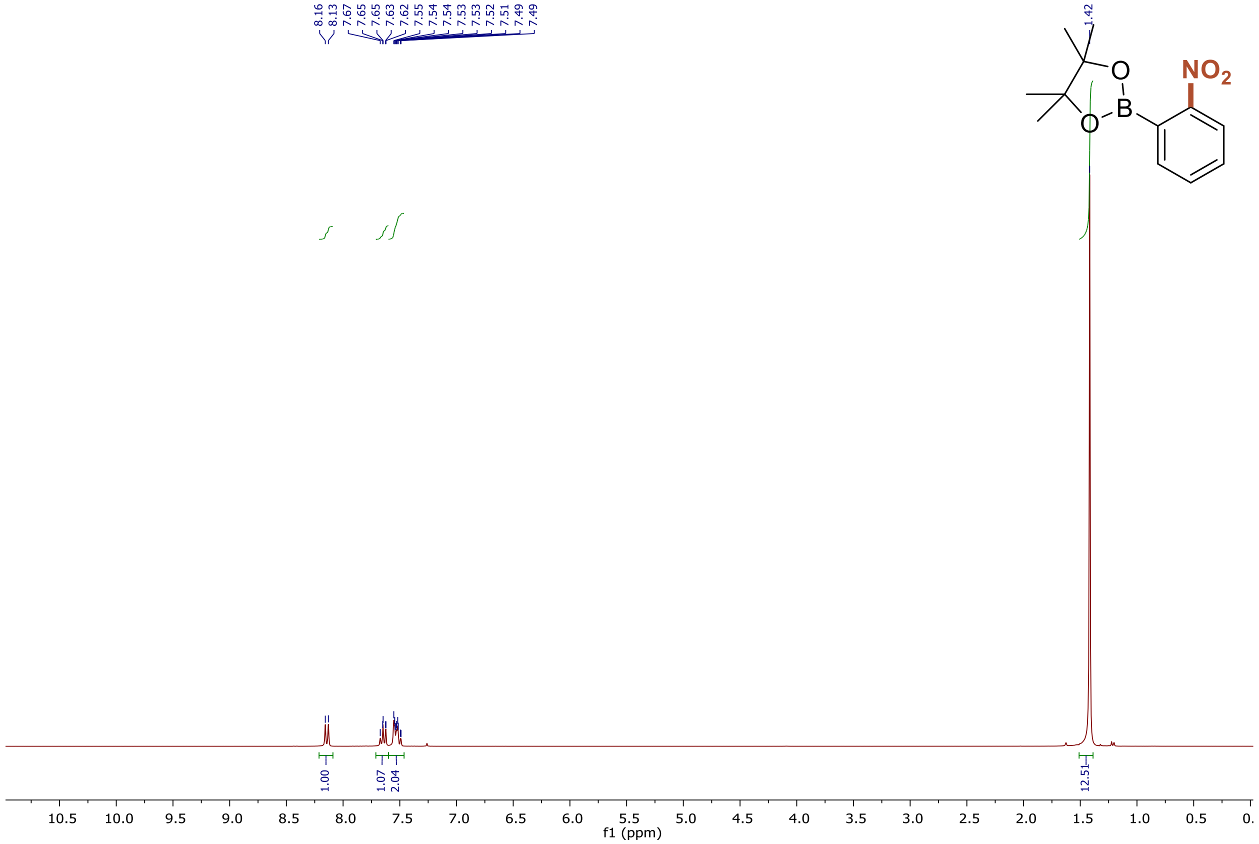

Supplementary Figure 139. ^{1}H NMR spectra for **35B**.

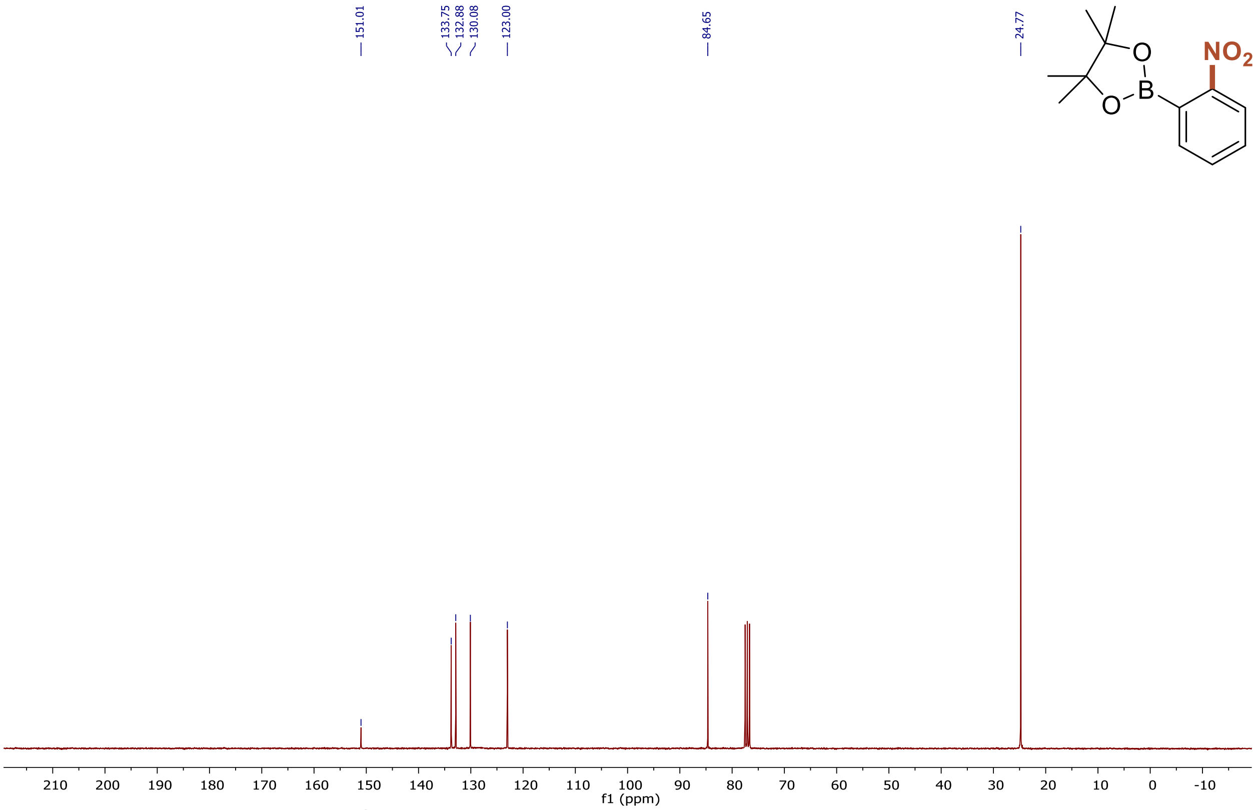

Supplementary Figure 140. ^{13}C NMR spectra for **35B**.

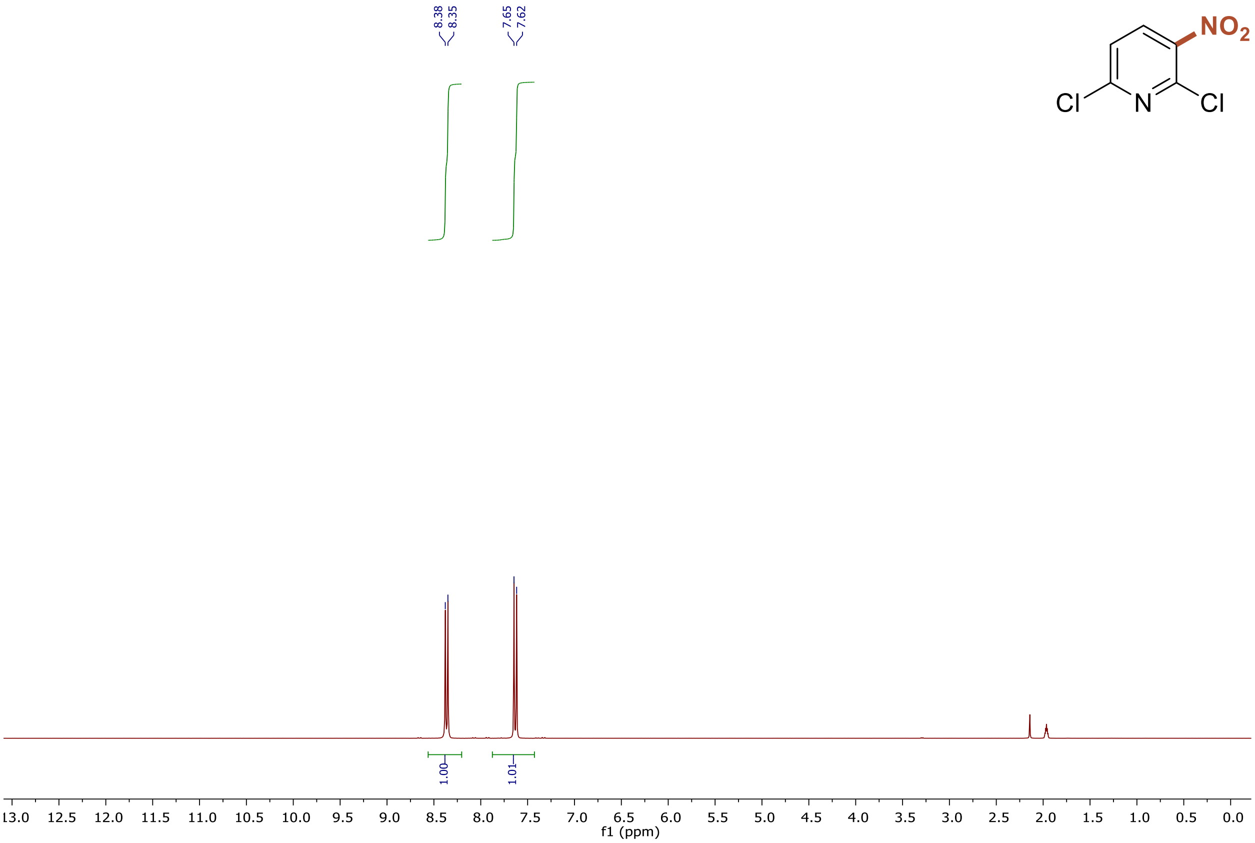

Supplementary Figure 141. ^{1}H NMR spectra for **36**.

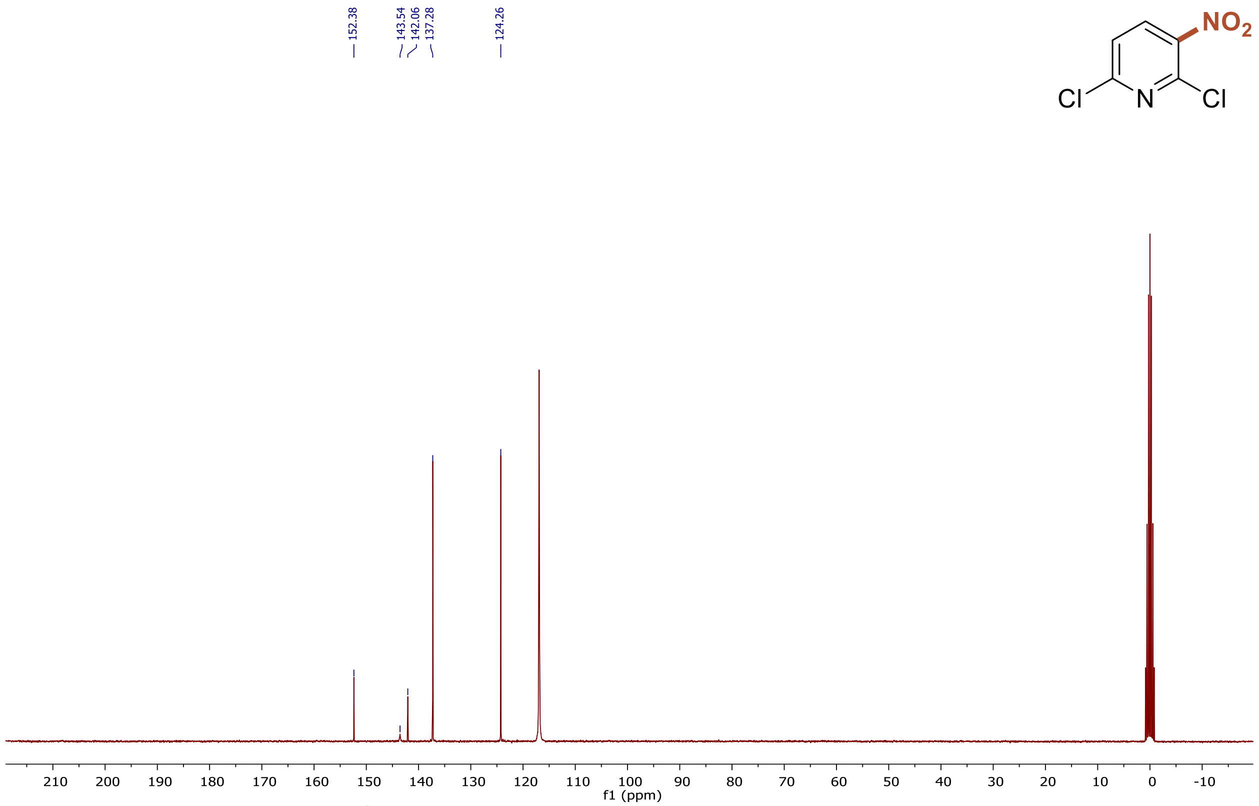

Supplementary Figure 142. ^{13}C NMR spectra for **36**.

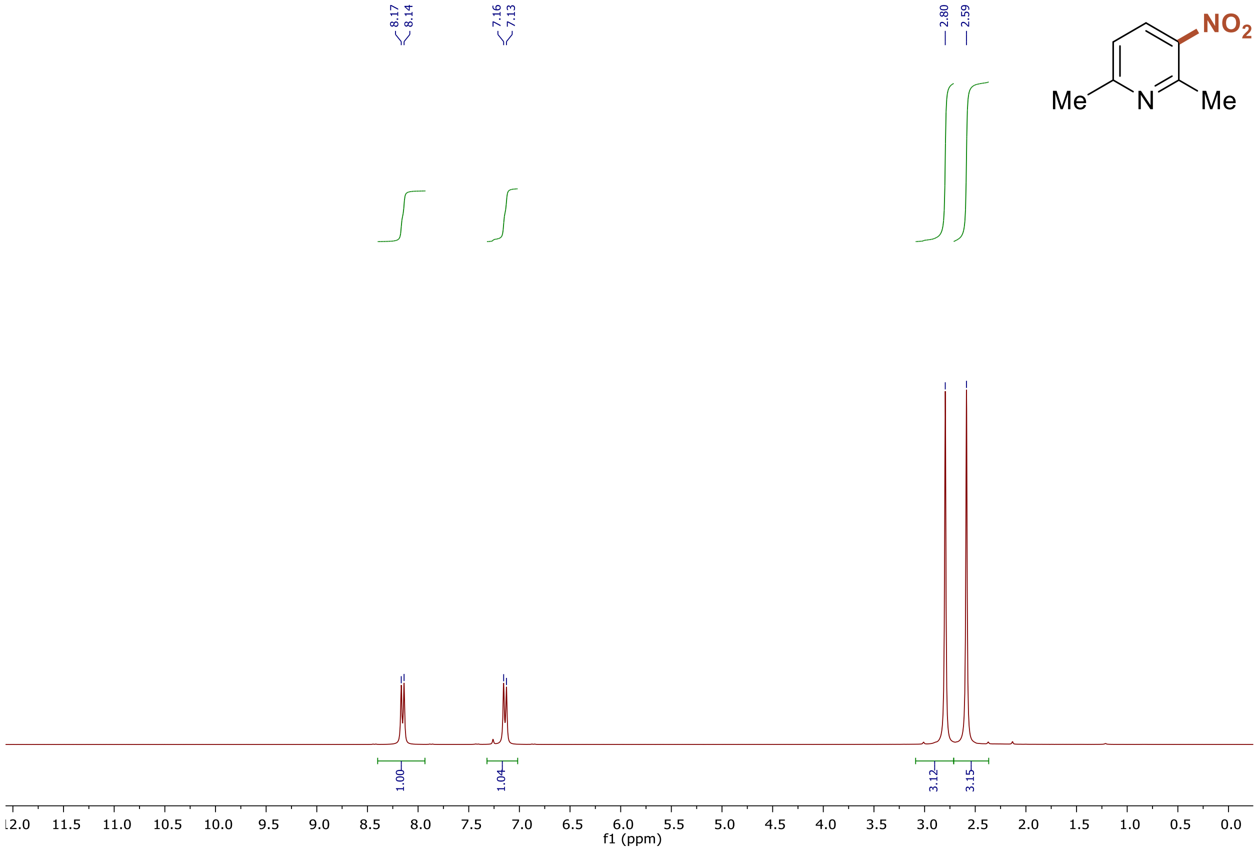

Supplementary Figure 143. ^{1}H NMR spectra for **37**.

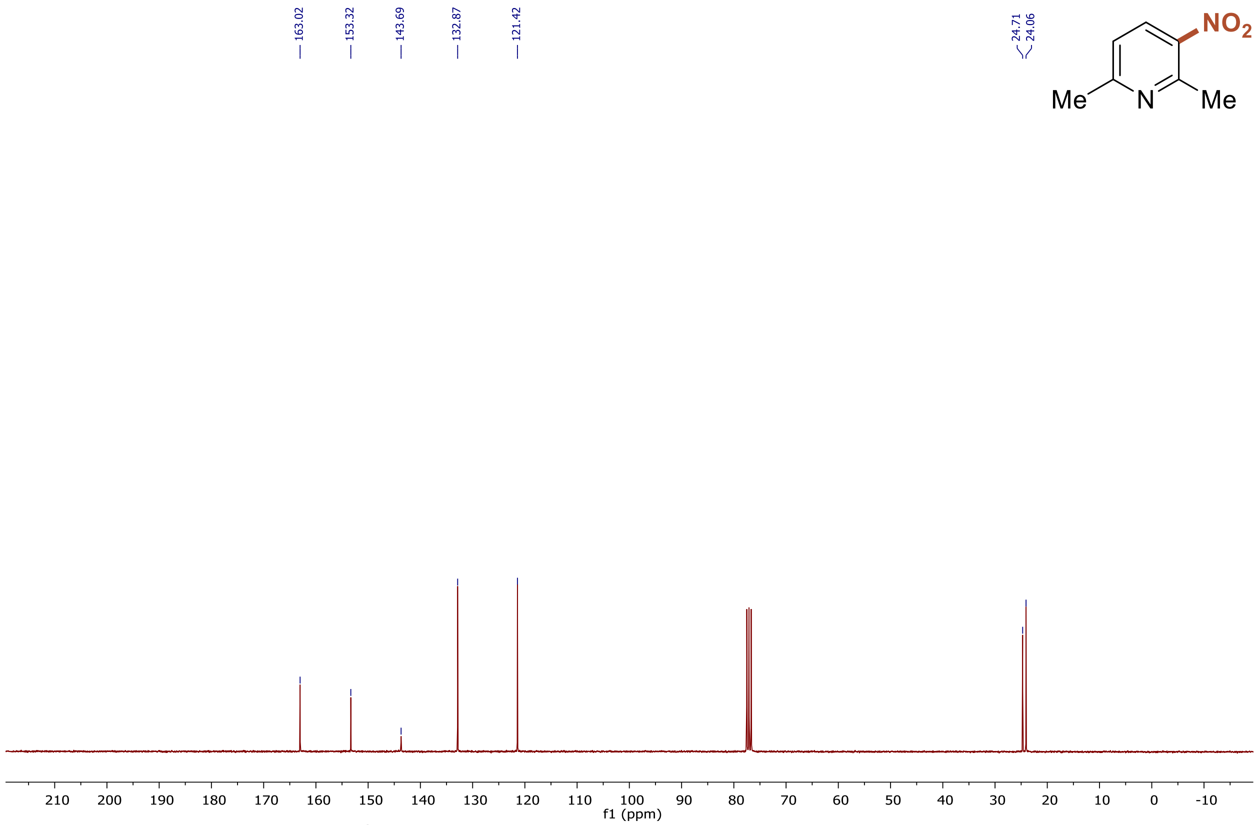

Supplementary Figure 144. ^{13}C NMR spectra for **37**.

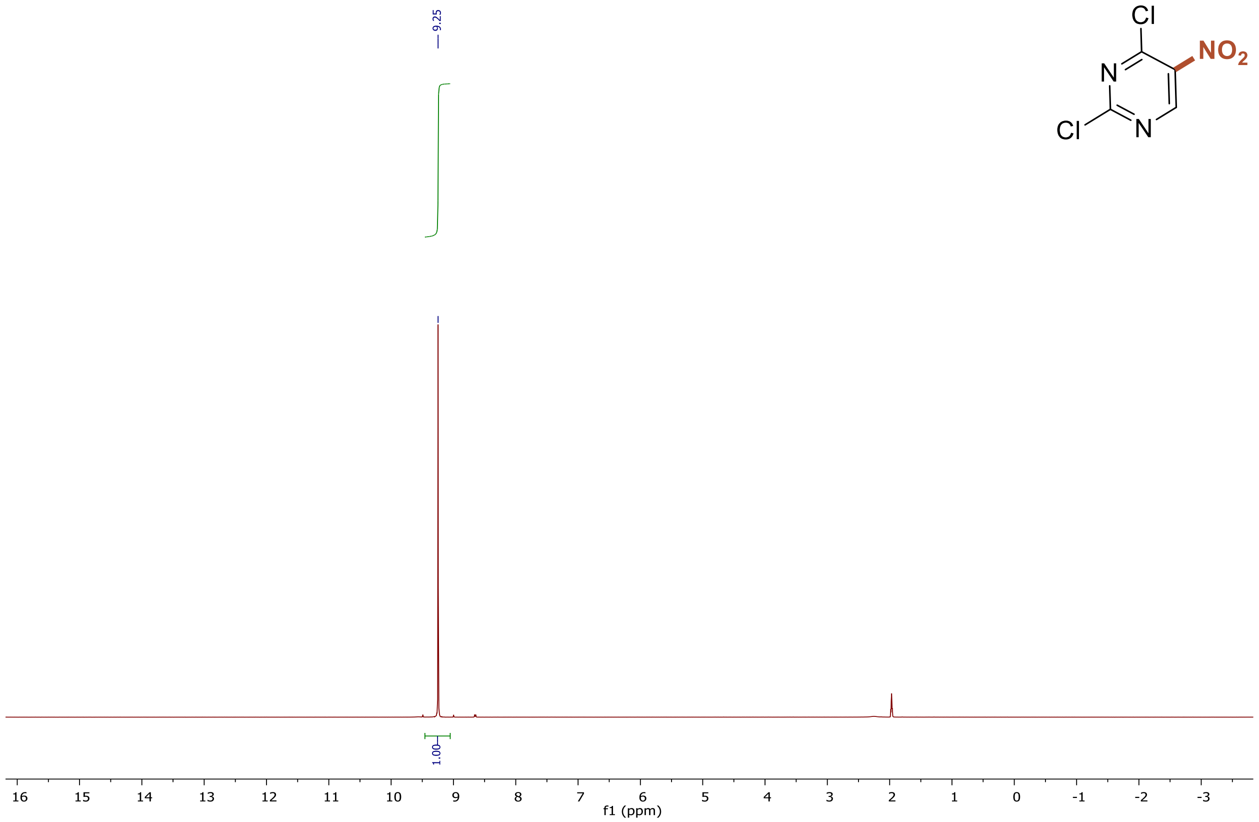

Supplementary Figure145. ^{1}H NMR spectra for **38**.

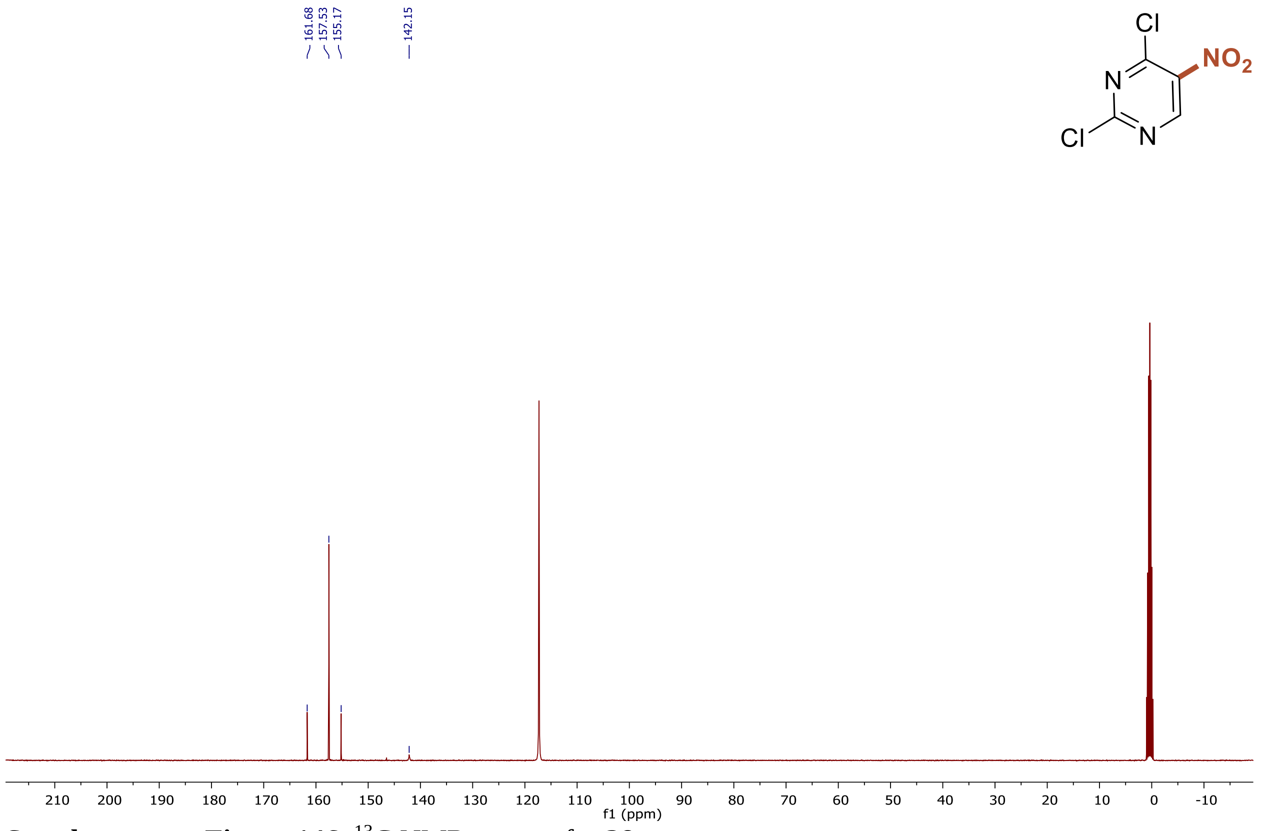

Supplementary Figure 146. ^{13}C NMR spectra for **38**.

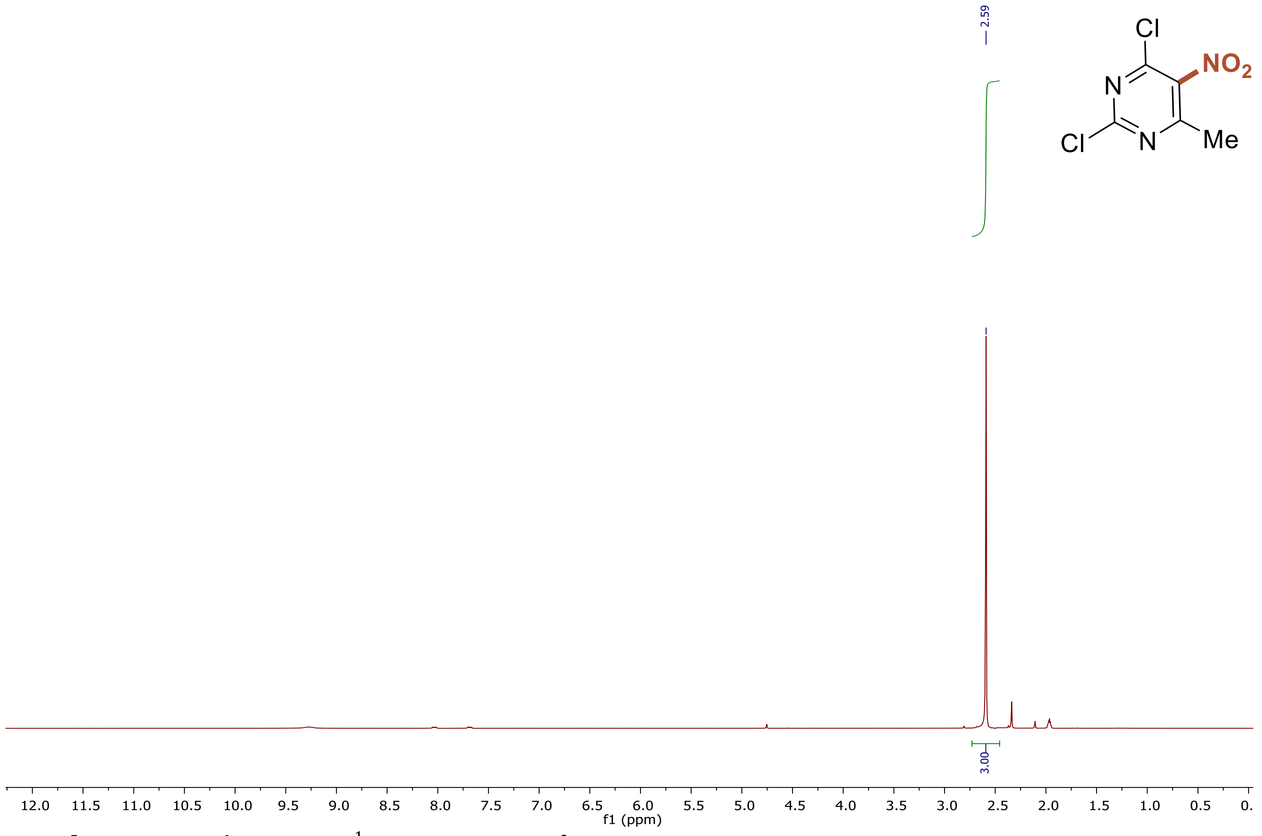

Supplementary Figure 147. ^{1}H NMR spectra for **39**.

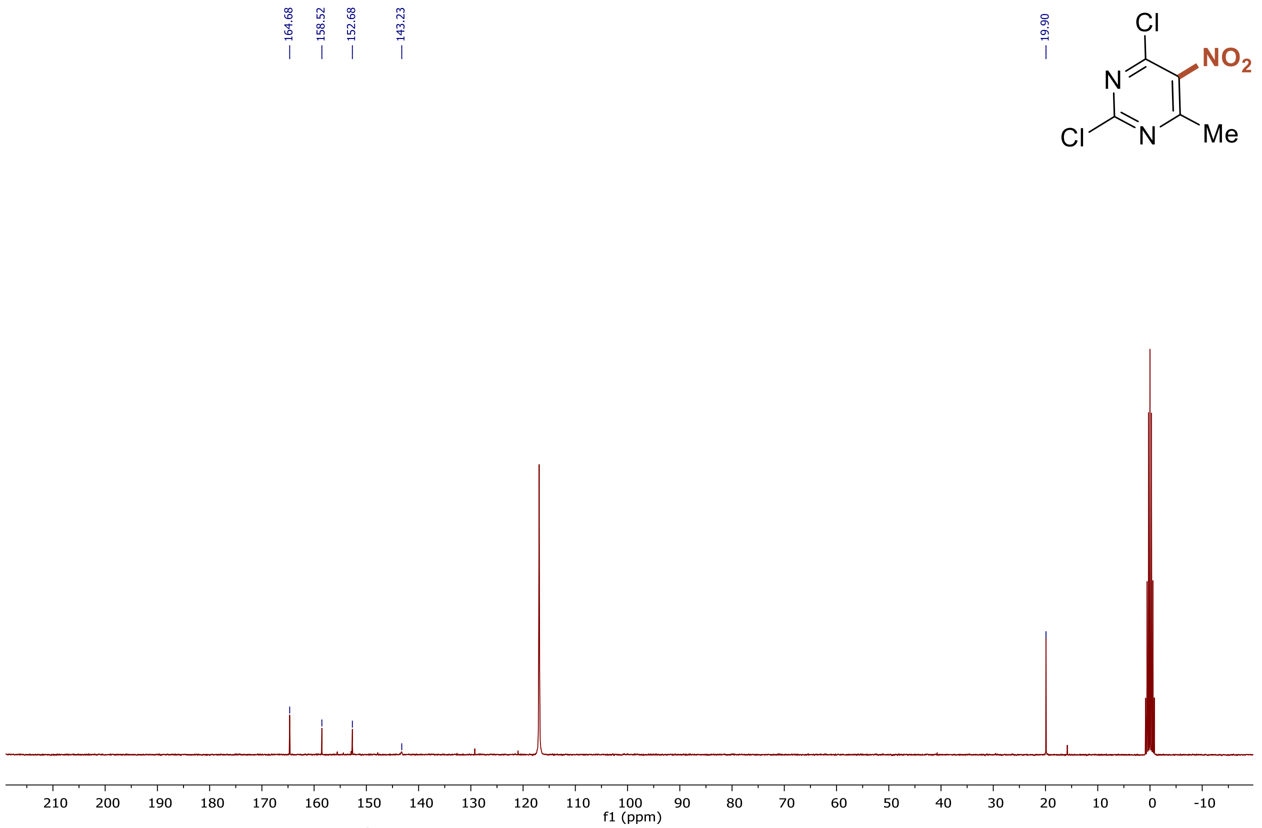

Supplementary Figure 148. ^{13}C NMR spectra for **39**.

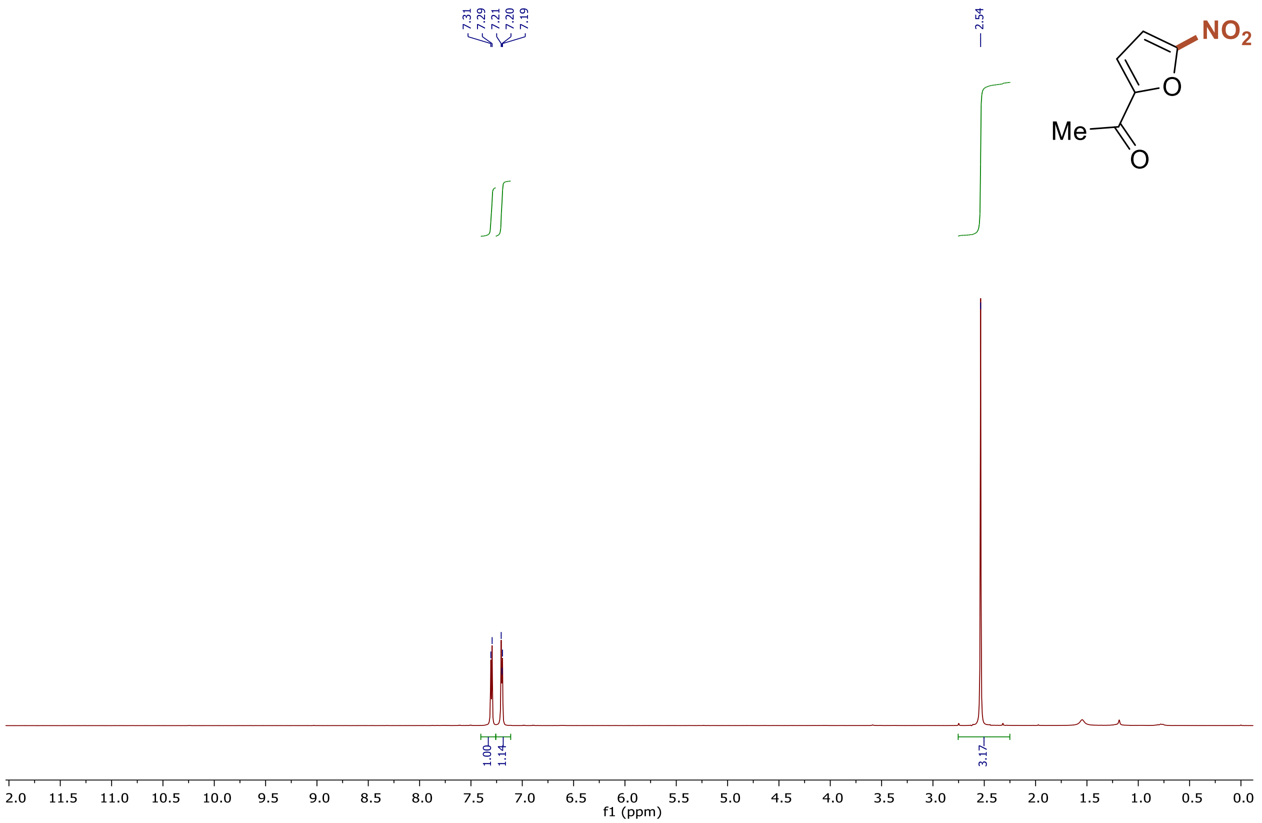

Supplementary Figure 149. ^{1}H NMR spectra for **40**.

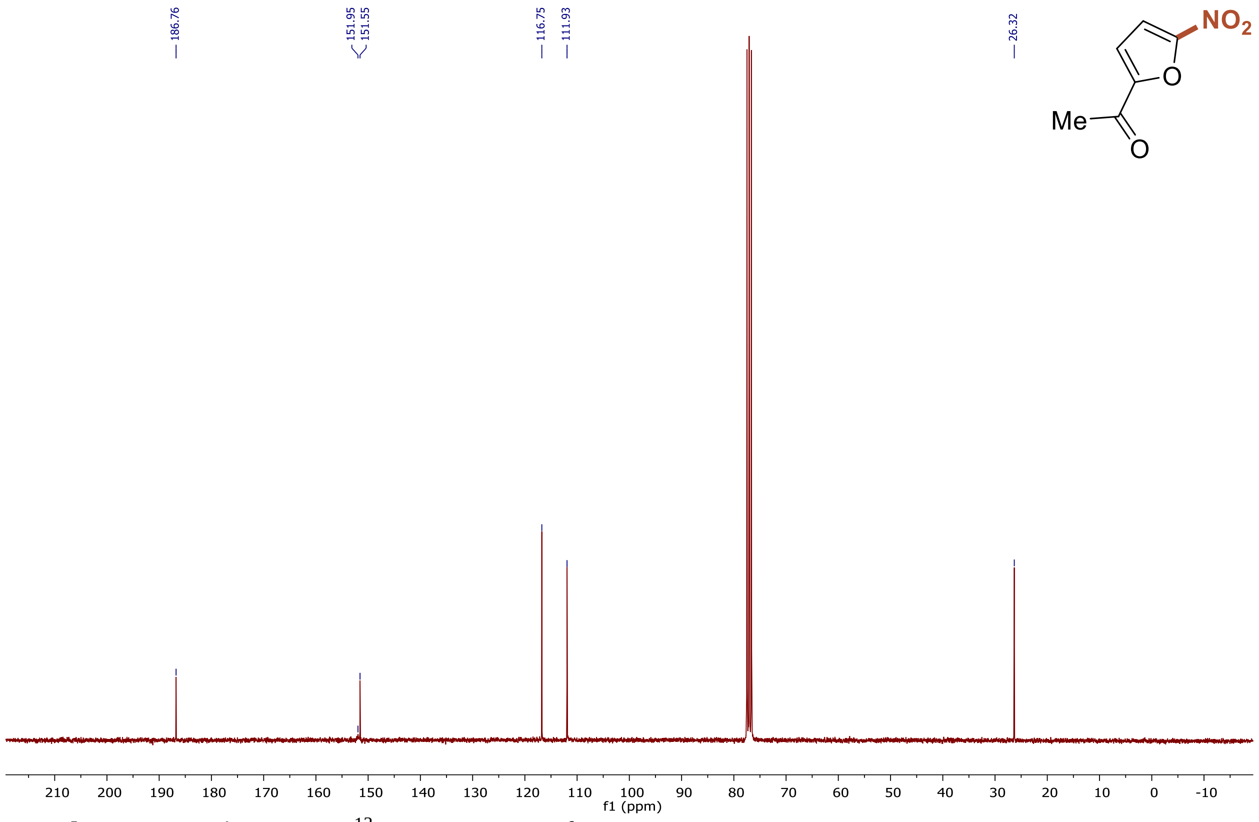

Supplementary Figure 150. ^{13}C NMR spectra for **40**

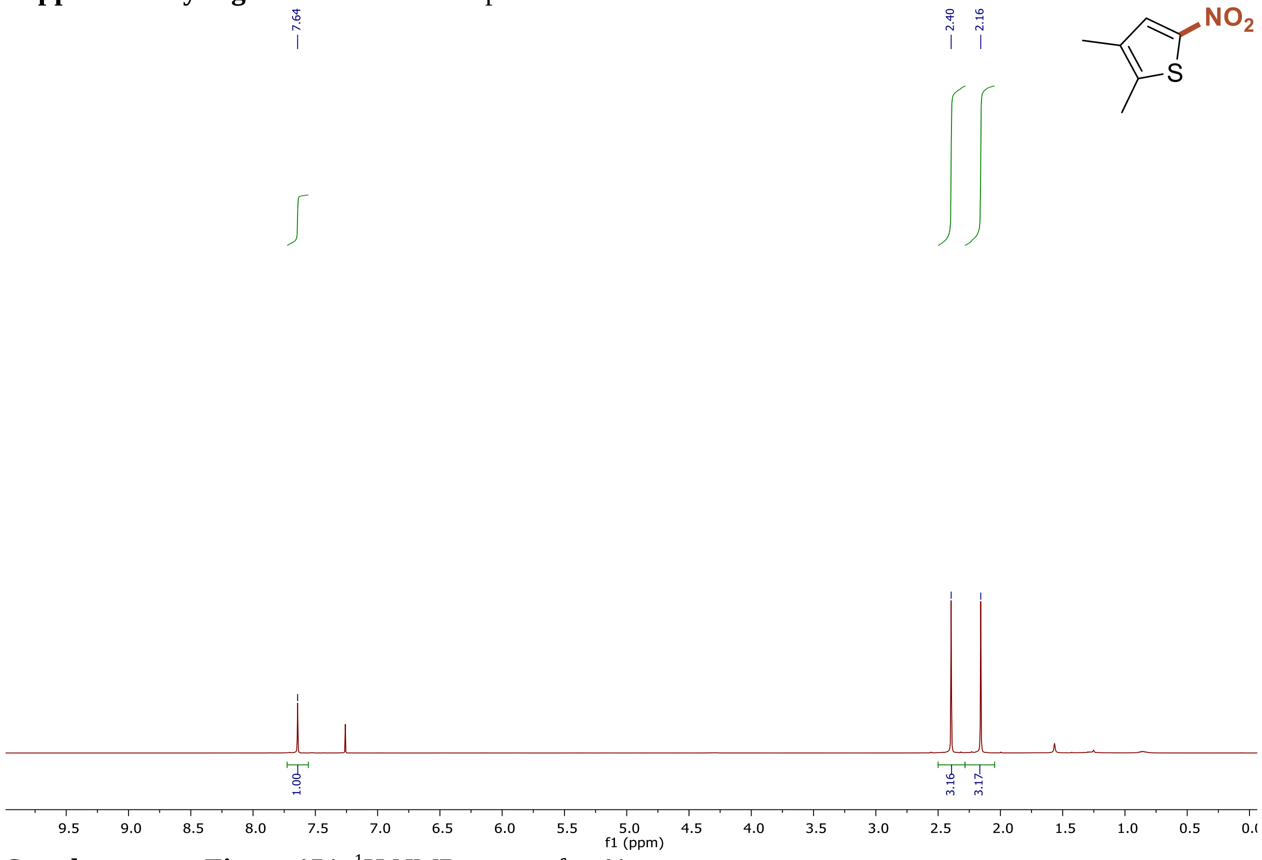

Supplementary Figure 151. ^{1}H NMR spectra for **41.**

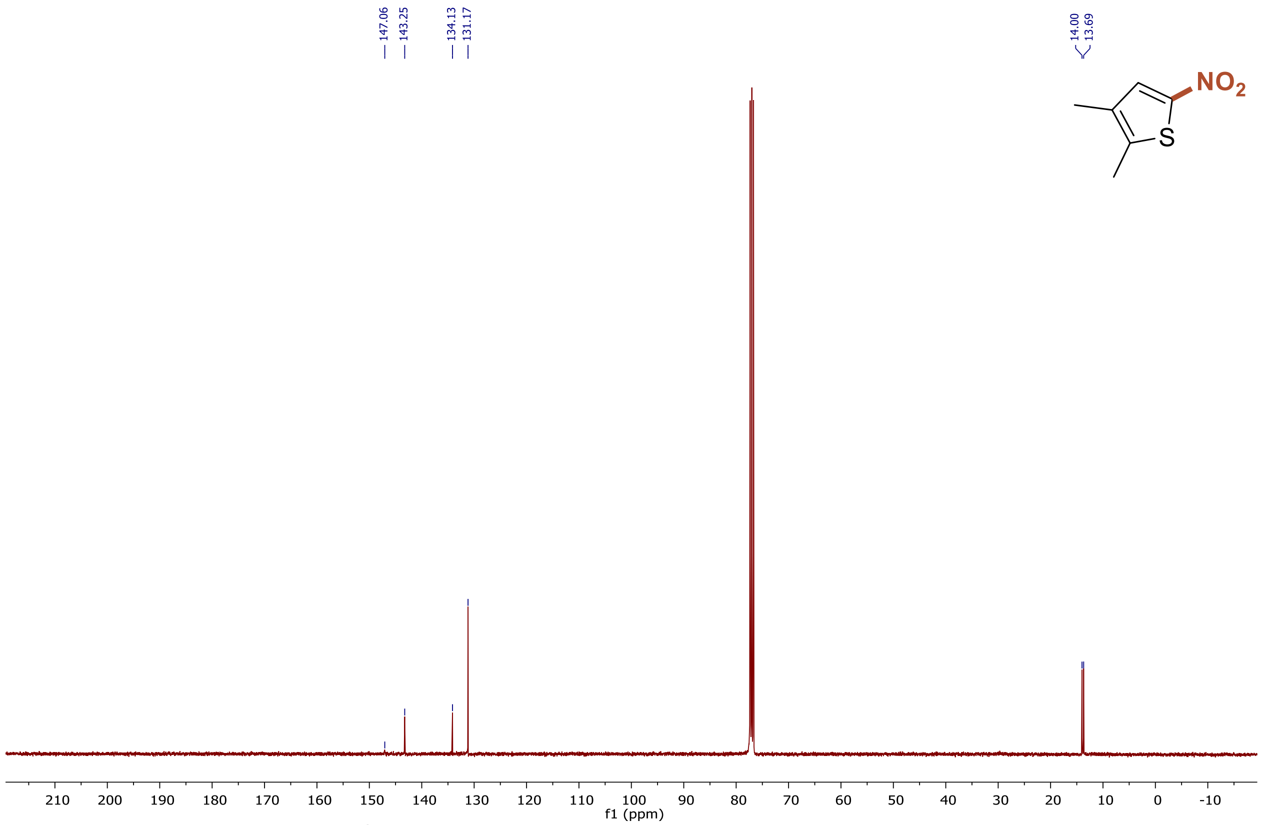

Supplementary Figure 152. ^{13}C NMR spectra for **41.**

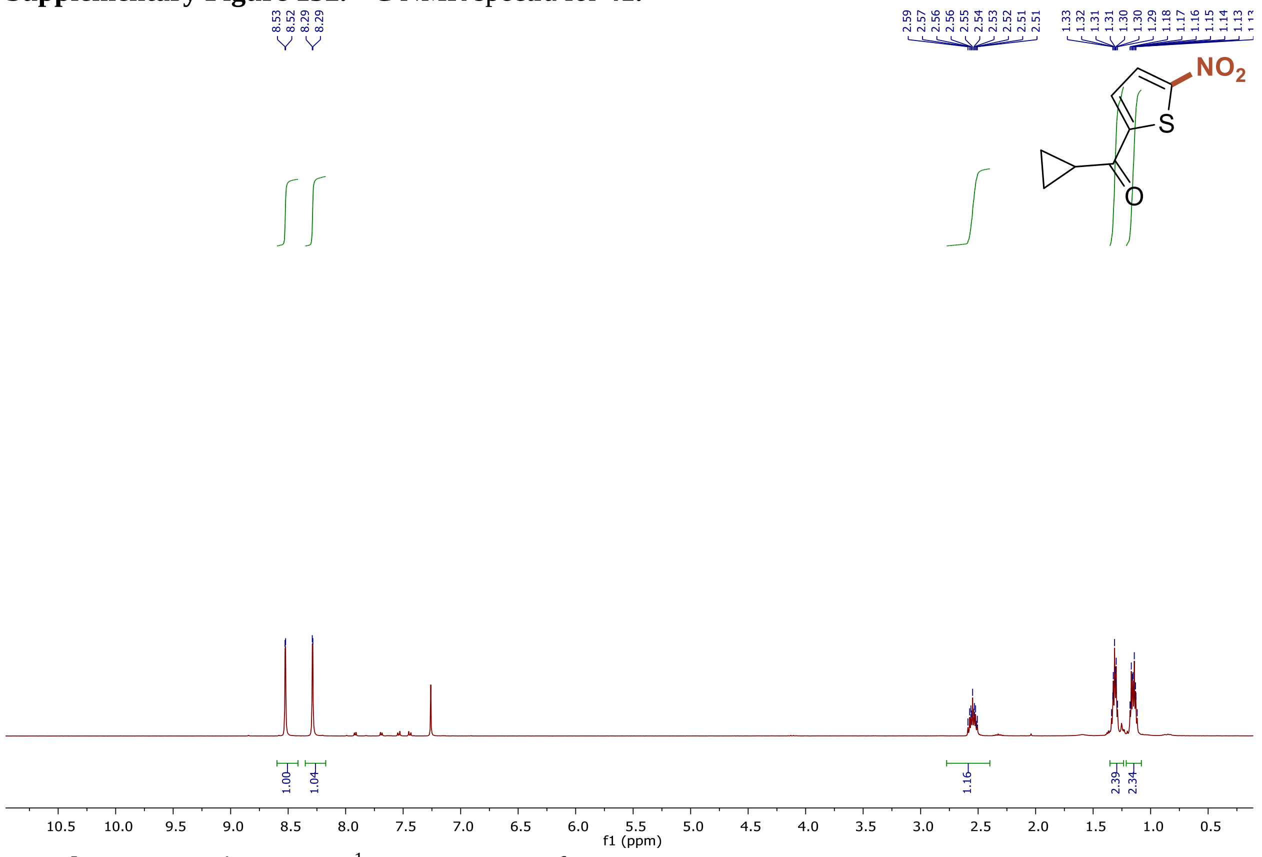

Supplementary Figure 153. ^{1}H NMR spectra for **42.**

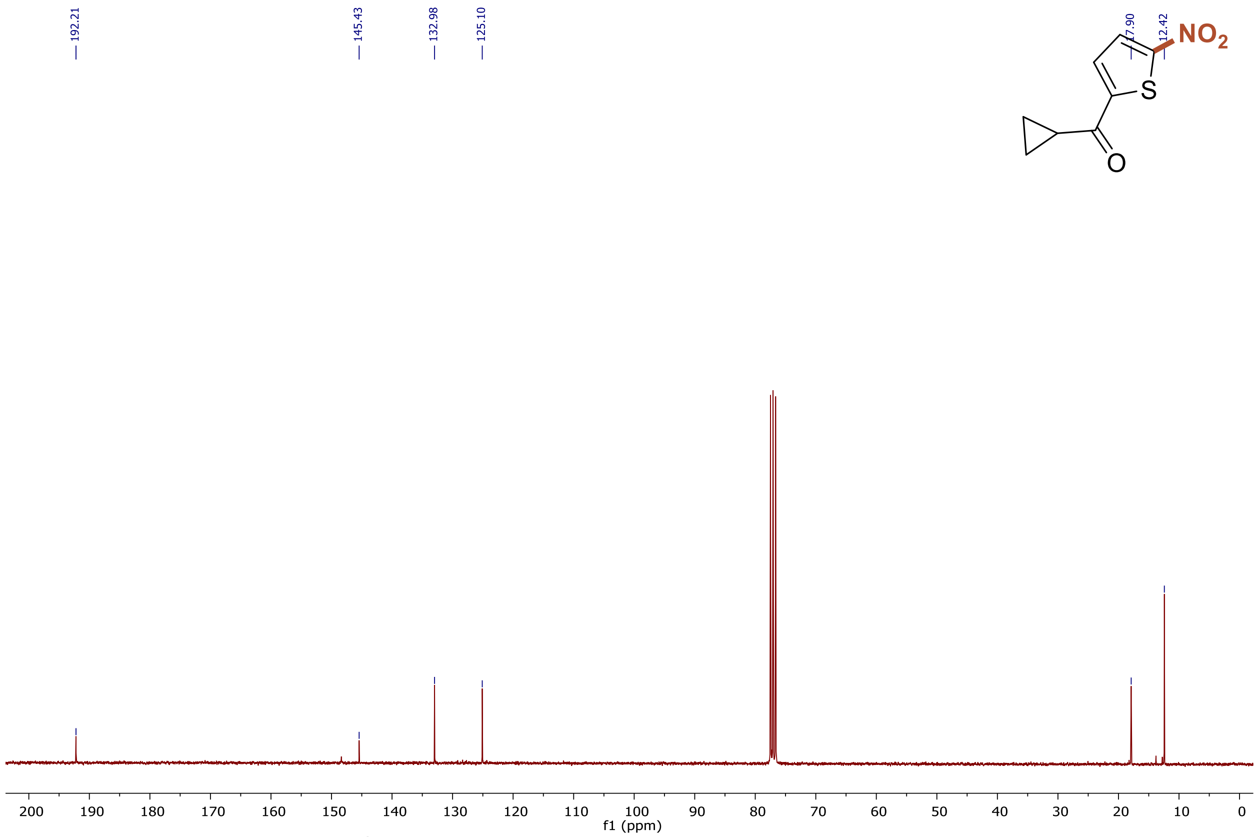

Supplementary Figure 154. ^{13}C NMR spectra for **42.**

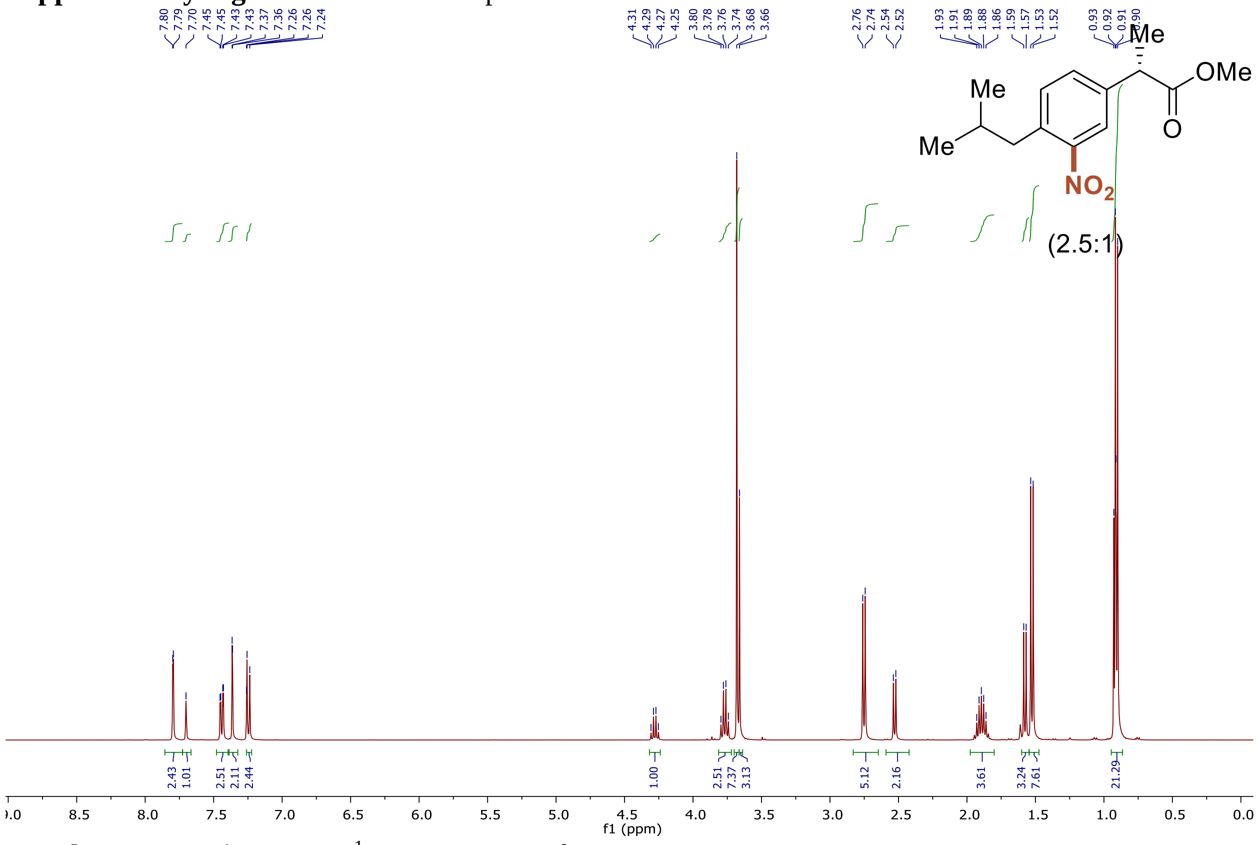

Supplementary Figure 155. ^{1}H NMR spectra for **43.**

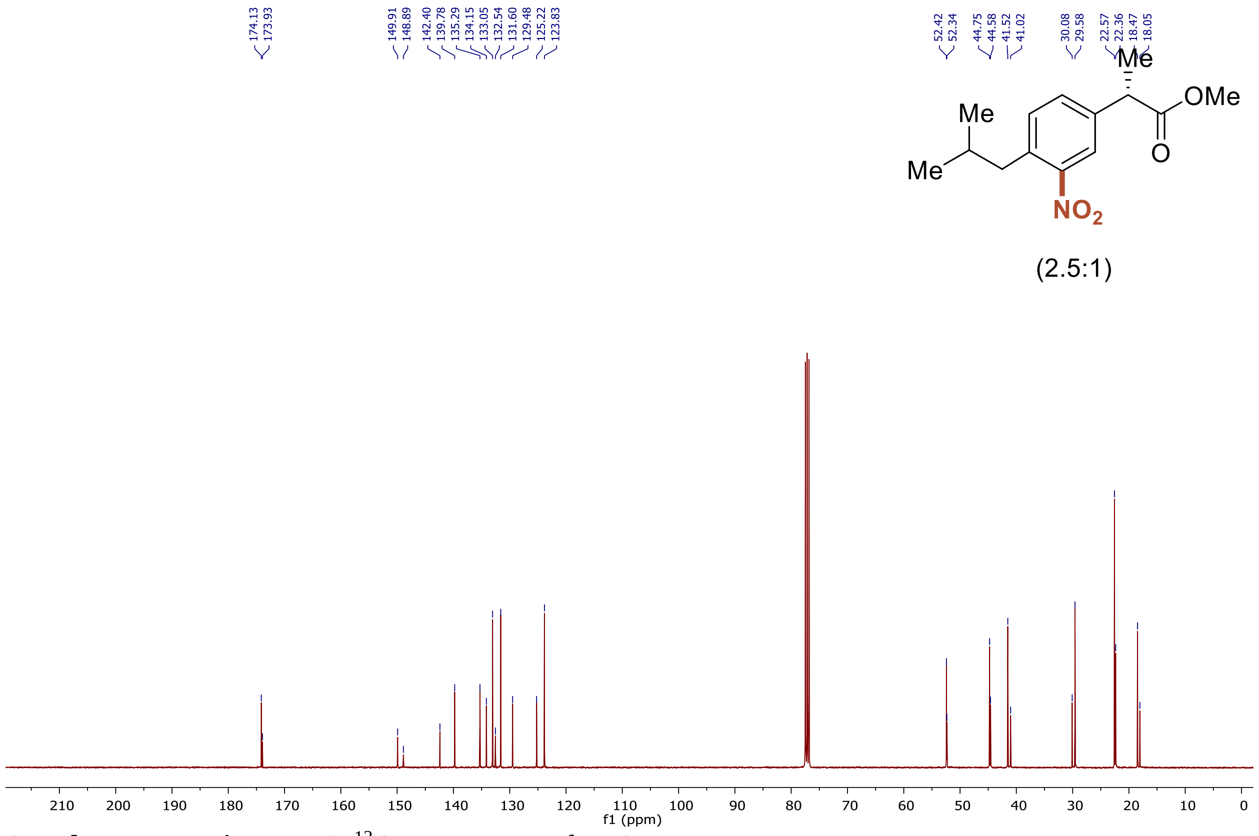

Supplementary Figure 156. ^{13}C NMR spectra for **43.**

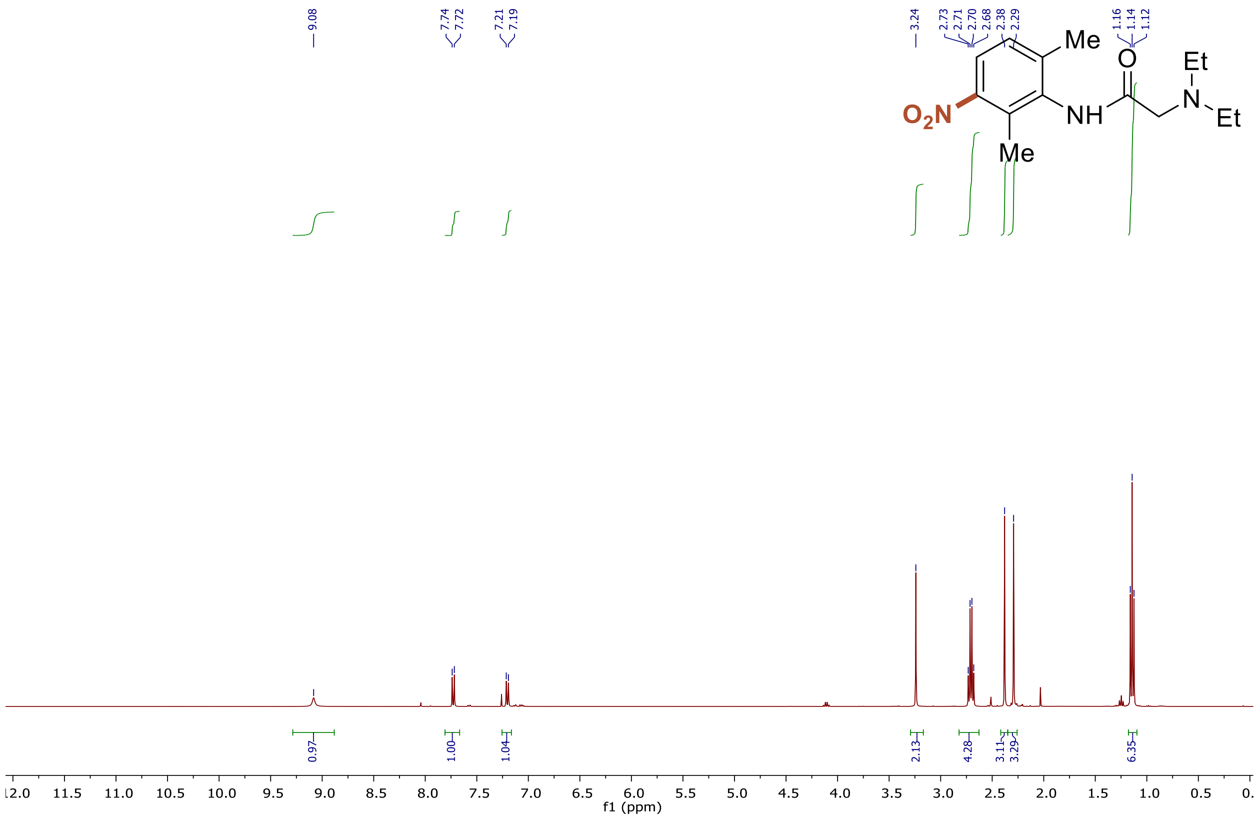

Supplementary Figure 157. ^{1}H NMR spectra for **44.**

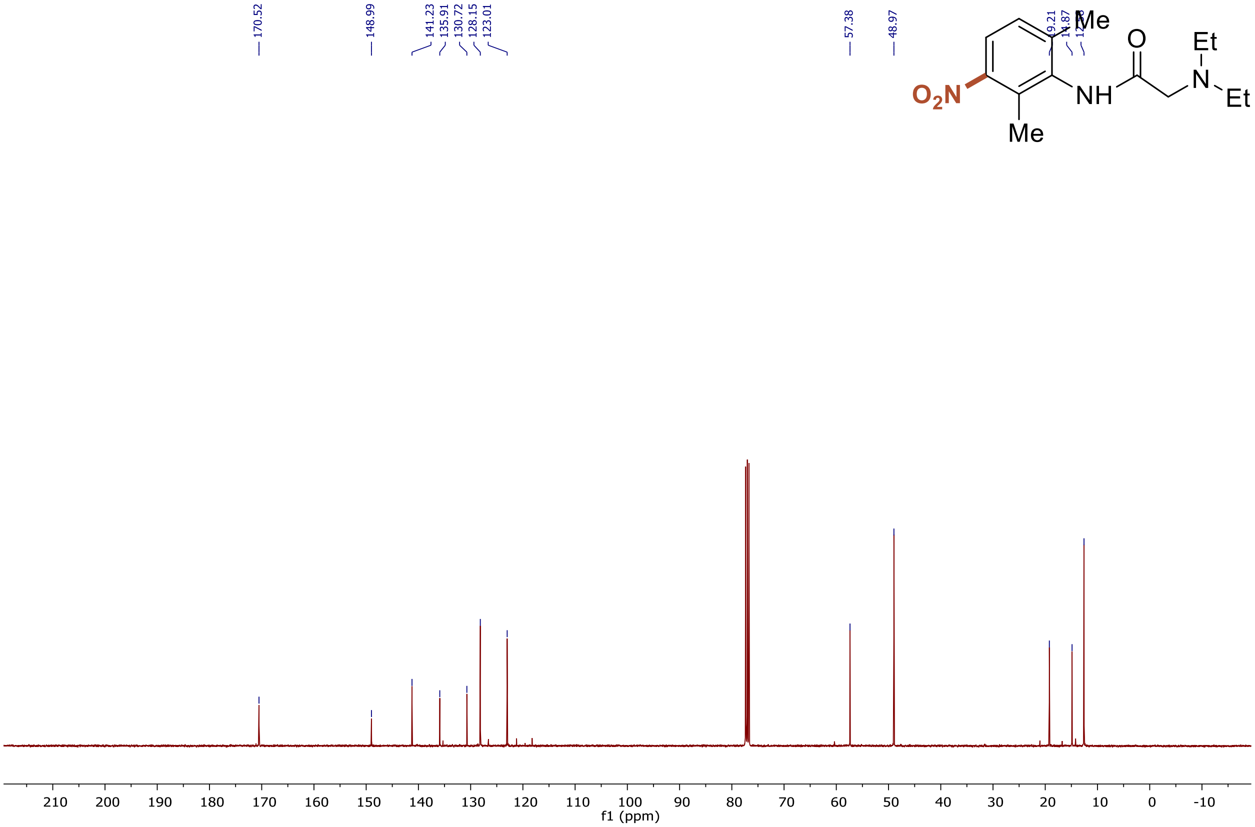

Supplementary Figure 158. ^{13}C NMR spectra for **44.**

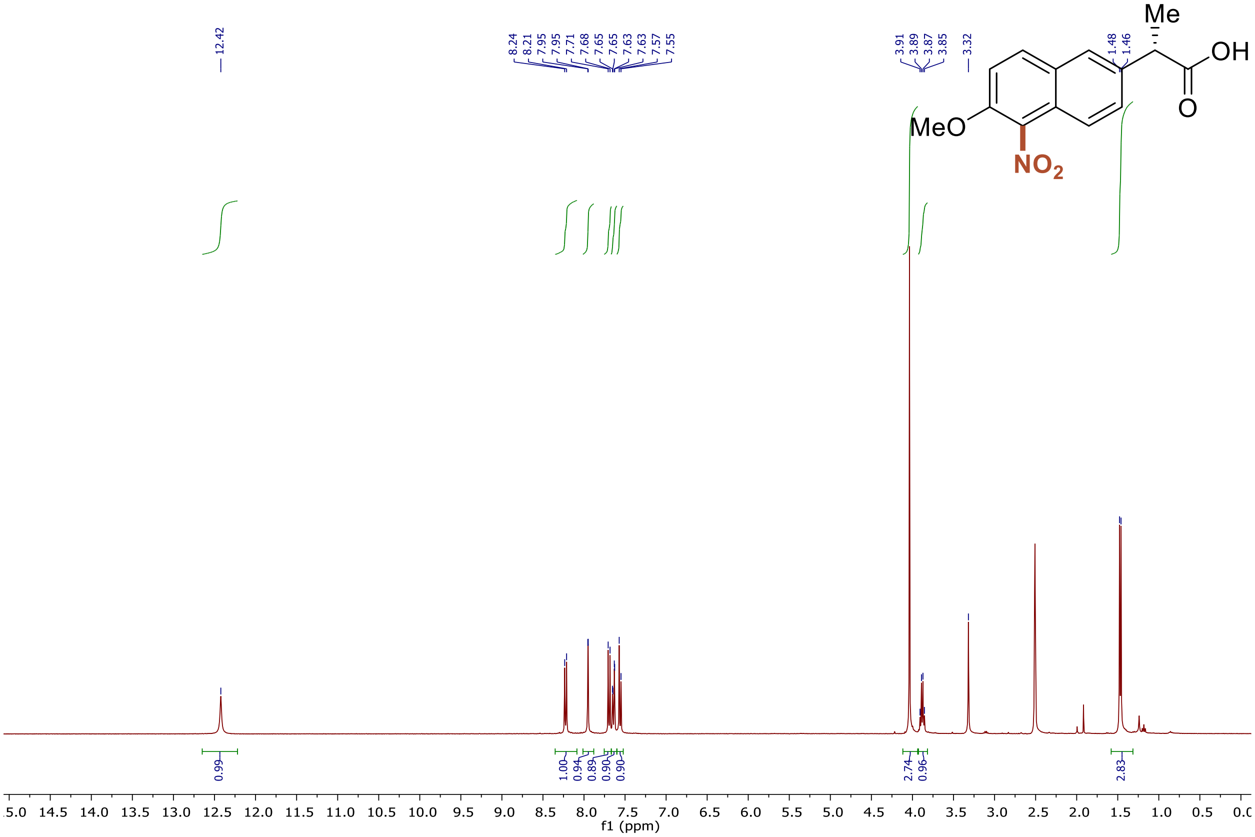

Supplementary Figure 159. ^{1}H NMR spectra for **45.**

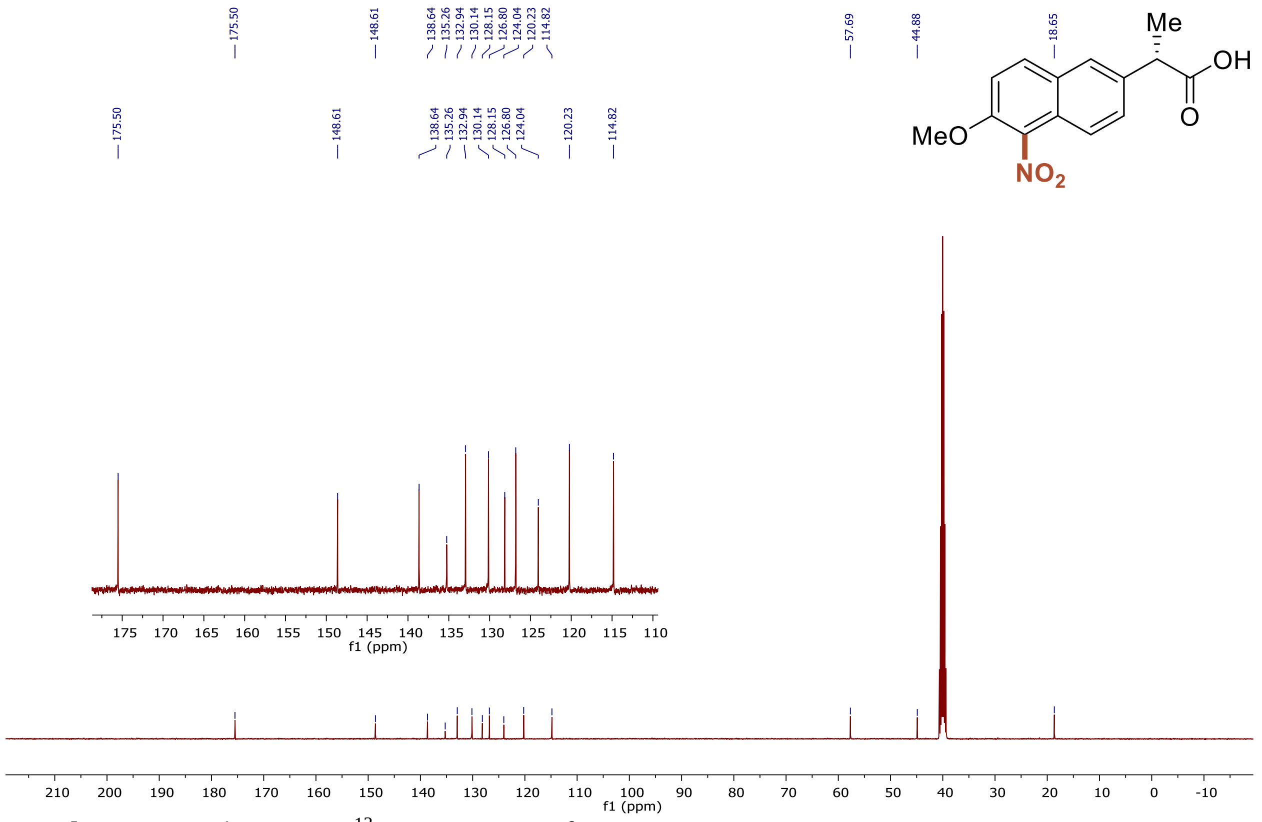

Supplementary Figure 160. ^{13}C NMR spectra for **45.**

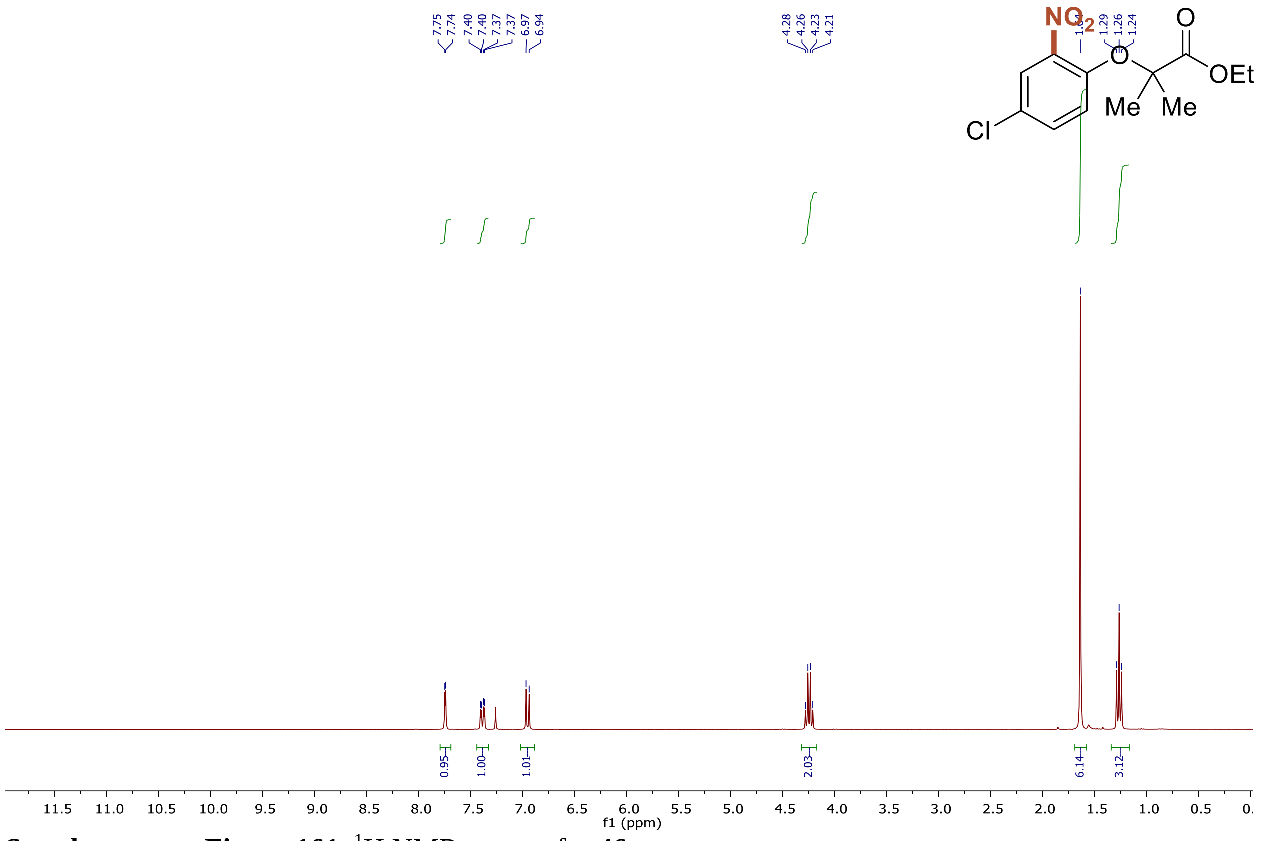

Supplementary Figure 161. ^{1}H NMR spectra for **46.**

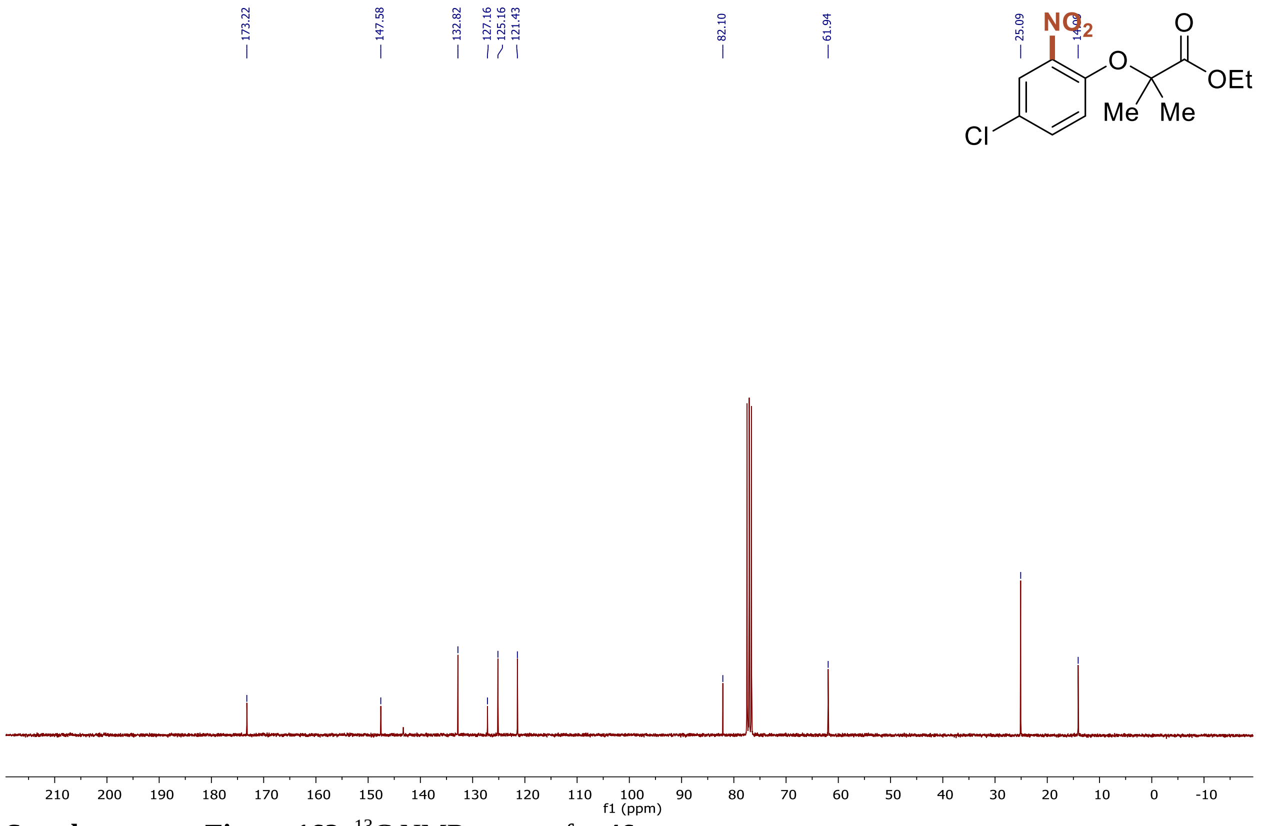

Supplementary Figure 162. ^{13}C NMR spectra for **46.**

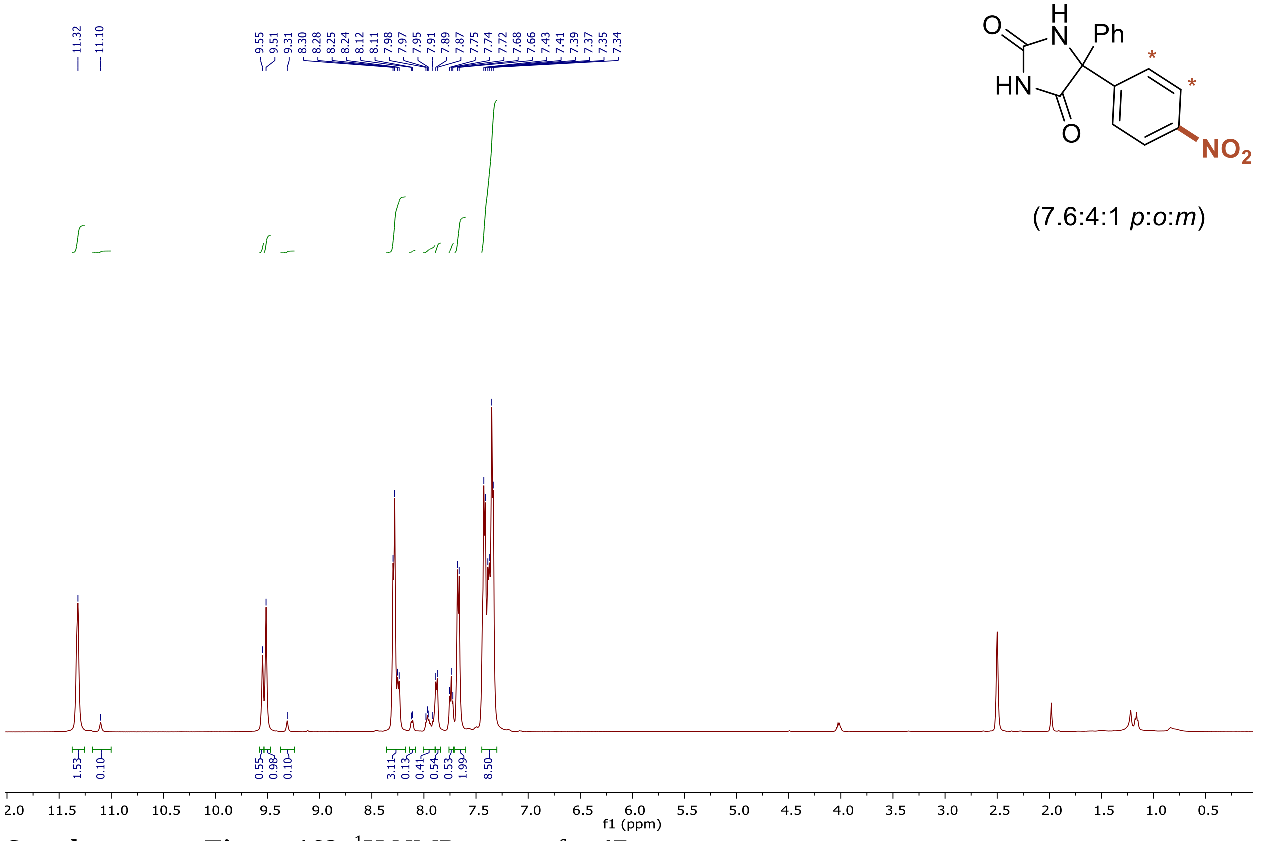

Supplementary Figure 163. ^{1}H NMR spectra for **47.**

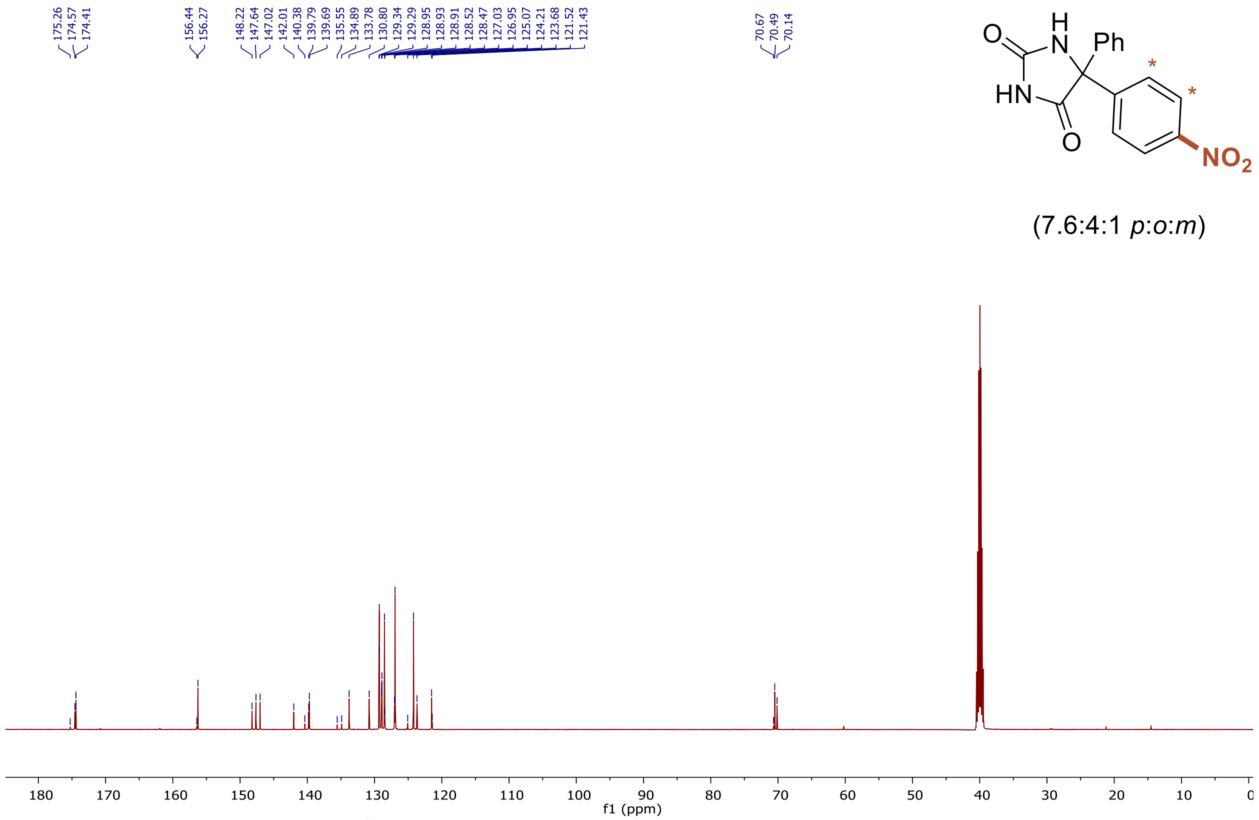

Supplementary Figure 164. ^{13}C NMR spectra for **47.**

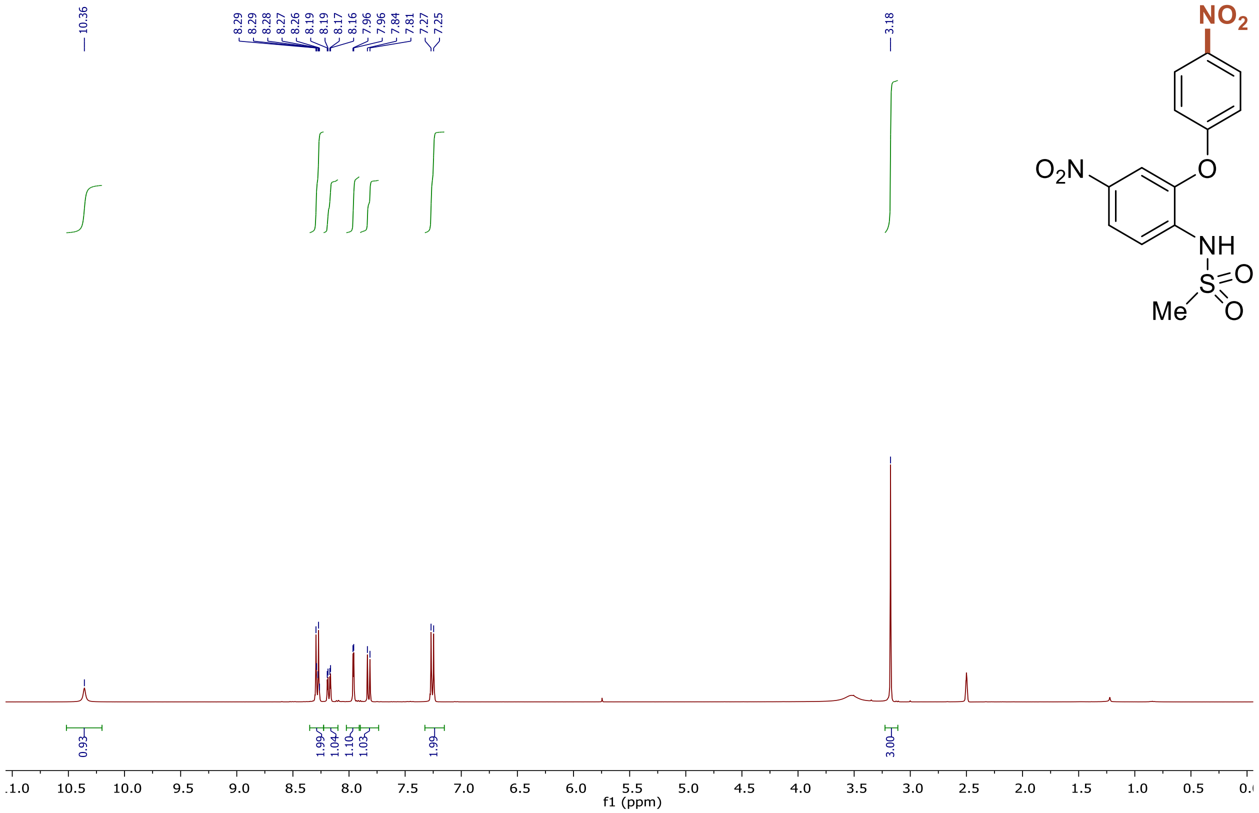

Supplementary Figure 165. ^{1}H NMR spectra for **48A.**

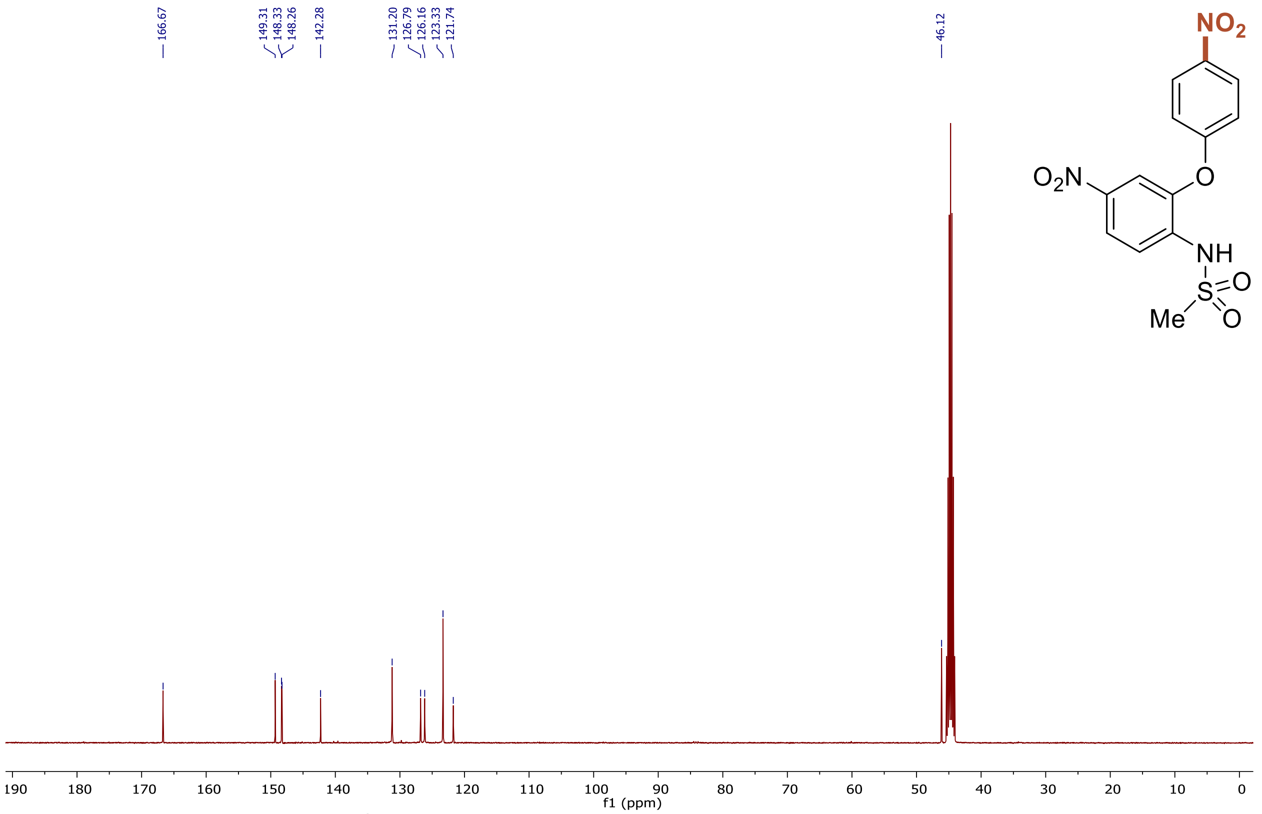

Supplementary Figure 166. ^{13}C NMR spectra for **48A.**

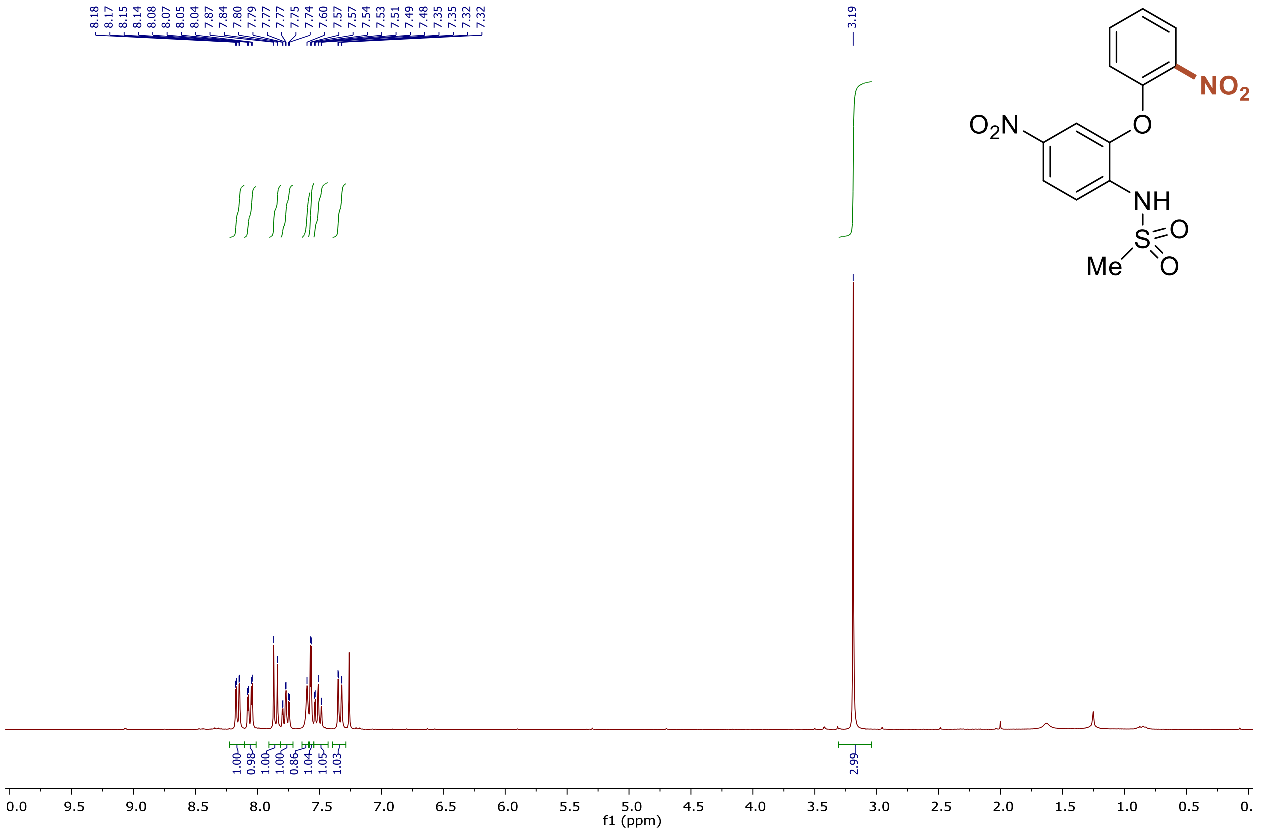

Supplementary Figure 167. ^{1}H NMR spectra for **48B.**

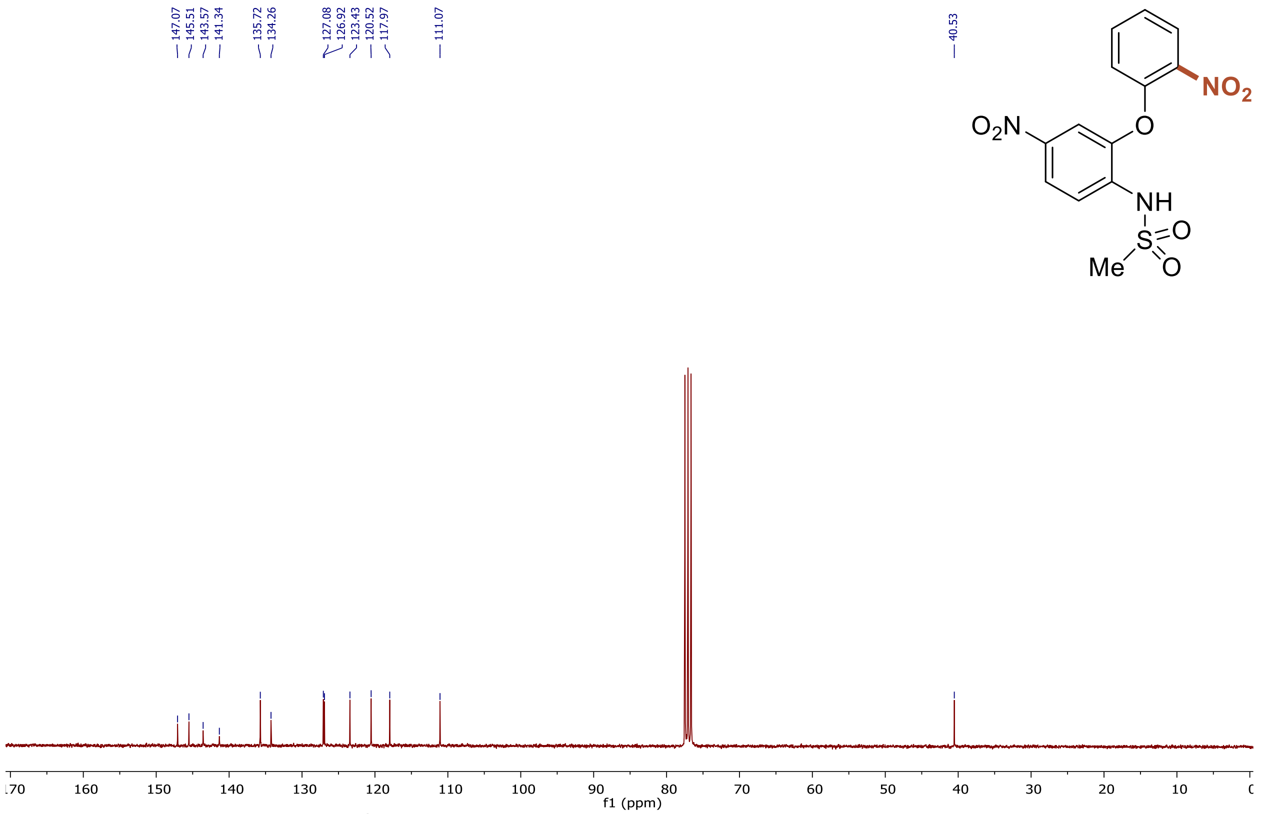

Supplementary Figure 168. ^{13}C NMR spectra for **48B.**

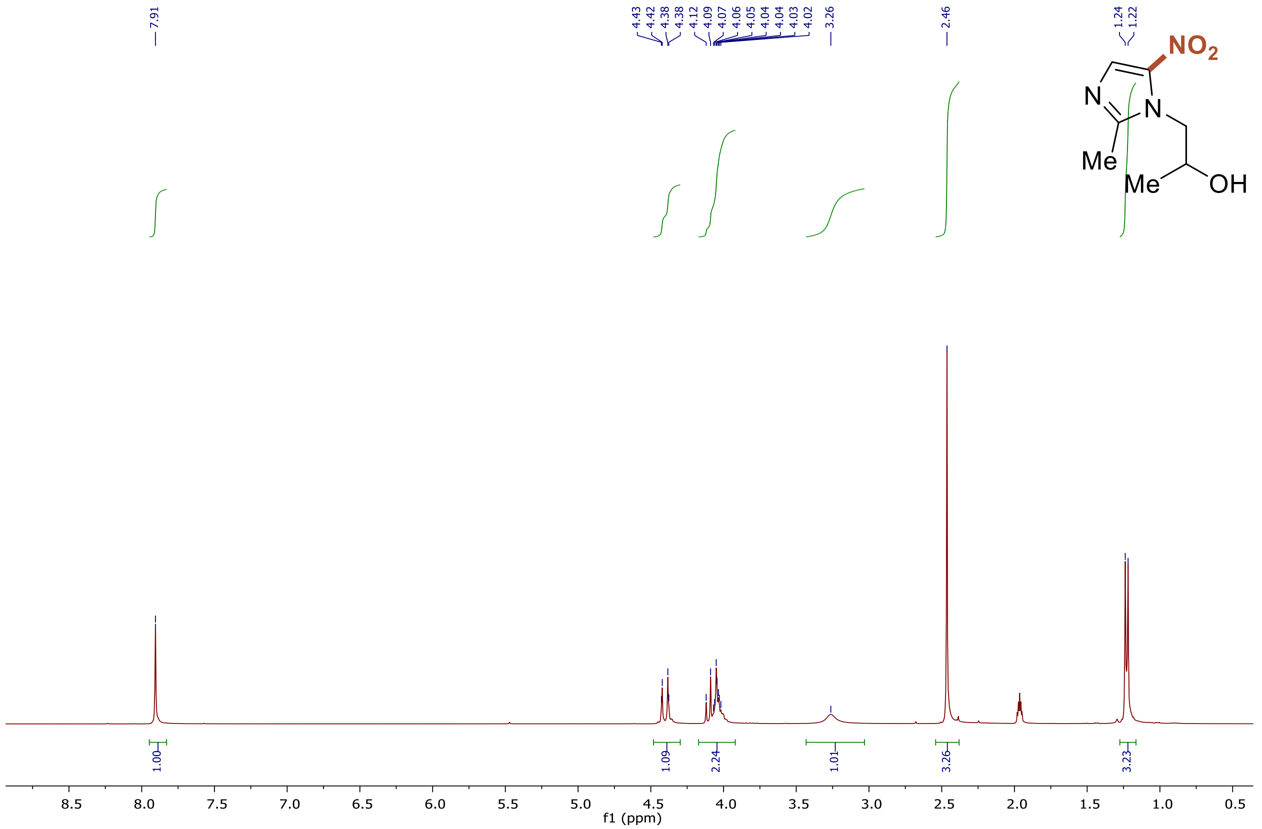

Supplementary Figure 169. ^{1}H NMR spectra for **49.**

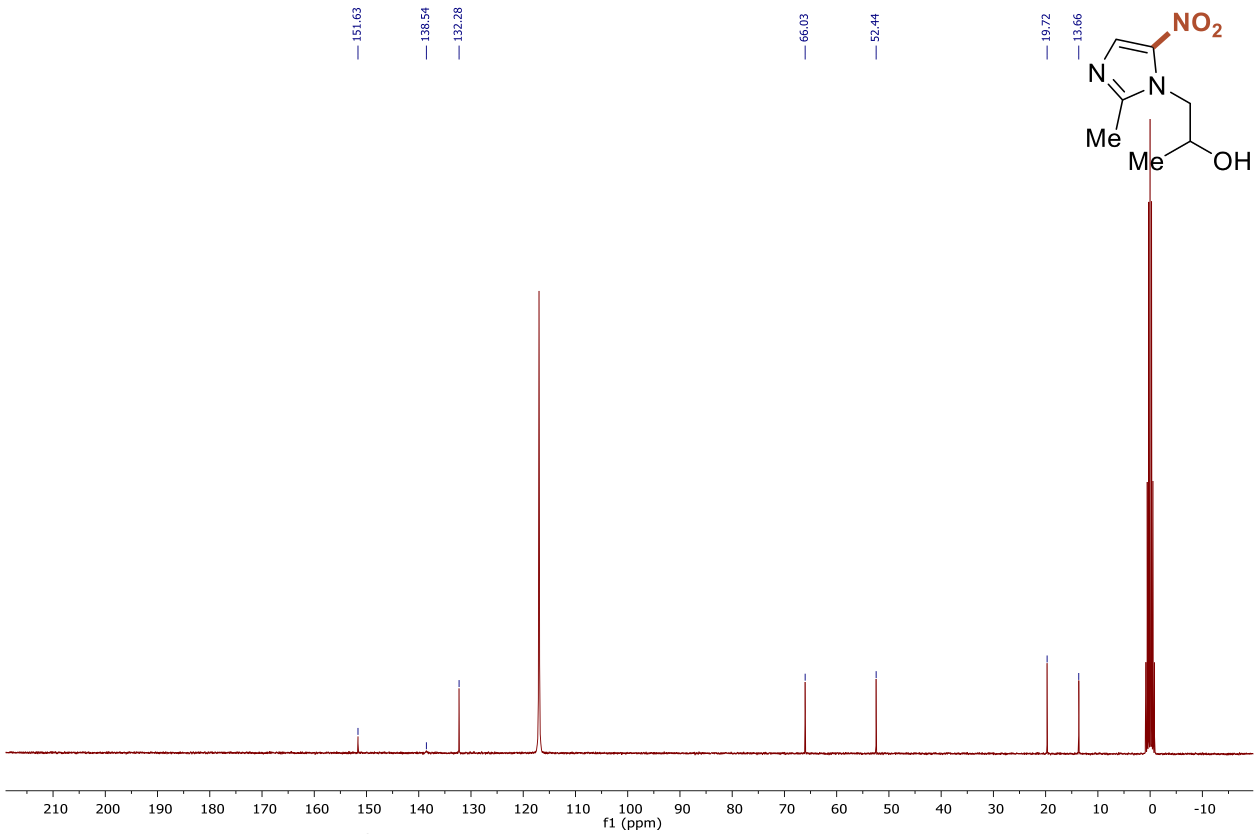

Supplementary Figure 170. ^{13}C NMR spectra for **49.**

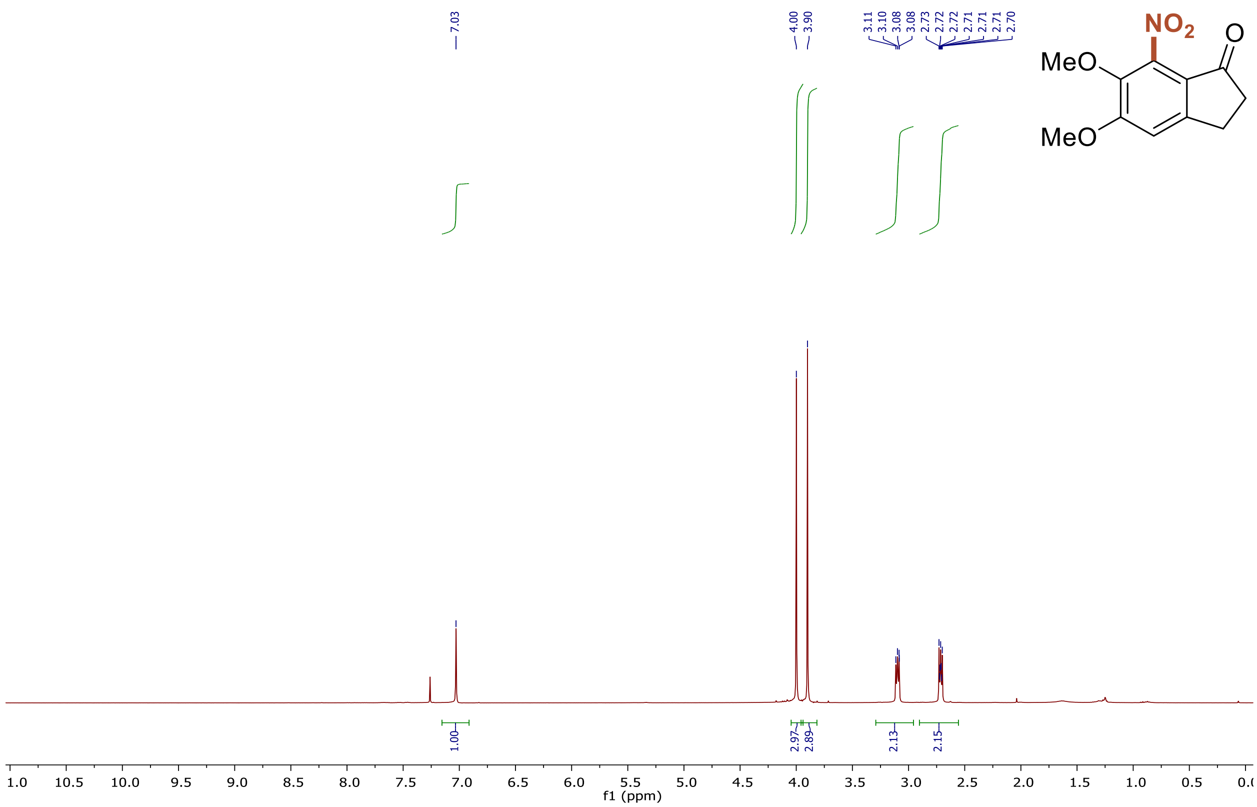

Supplementary Figure 171. ^{1}H NMR spectra for **50.**

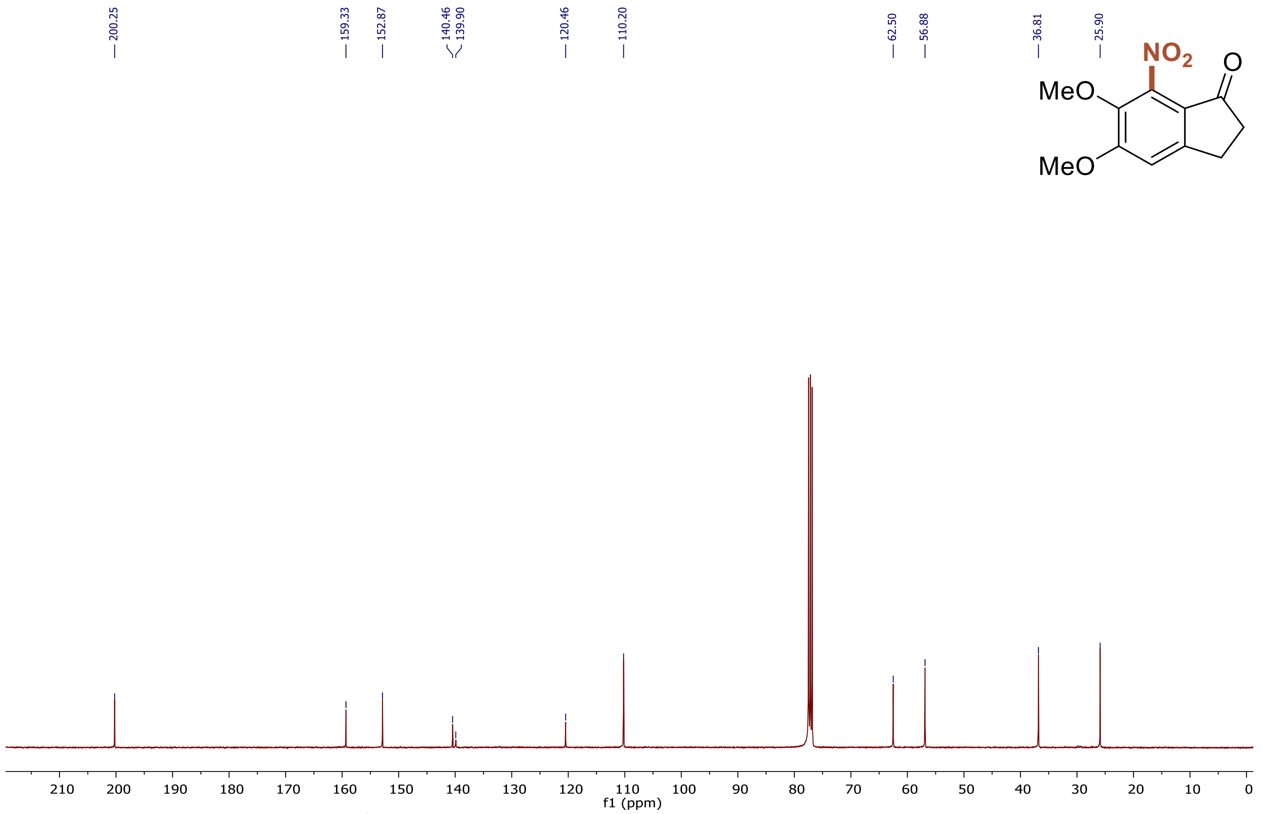

Supplementary Figure 172. ^{13}C NMR spectra for **50.**

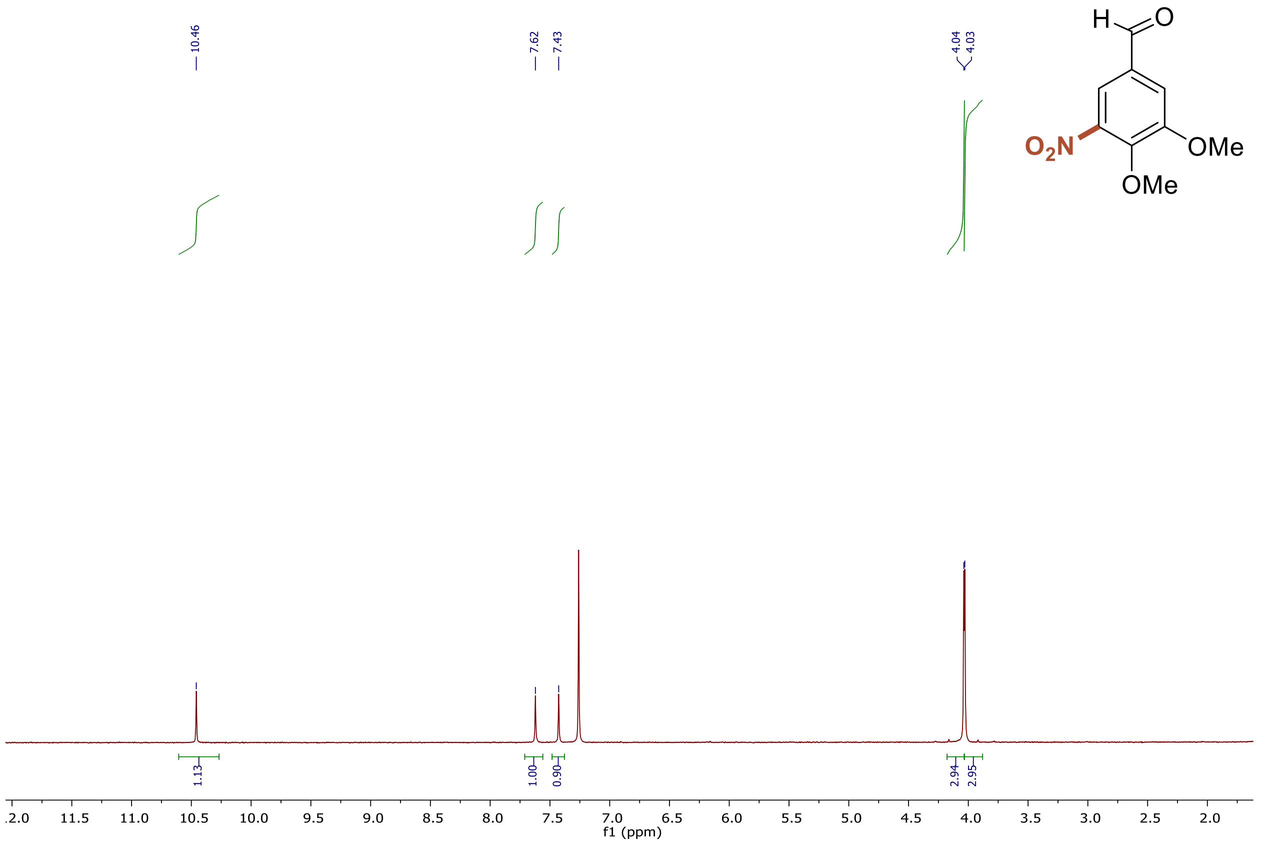

Supplementary Figure 173. ^{1}H NMR spectra for **51.**

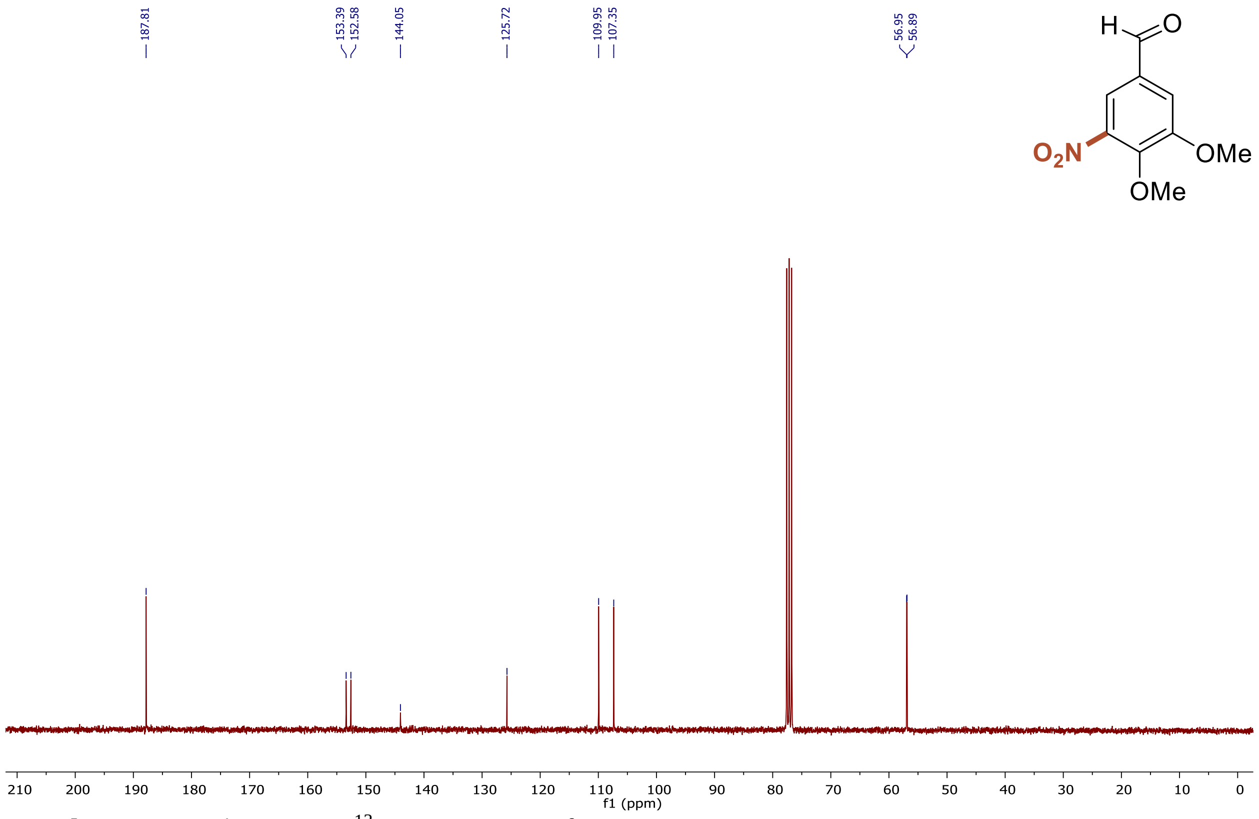

Supplementary Figure 174. ^{13}C NMR spectra for **51.**

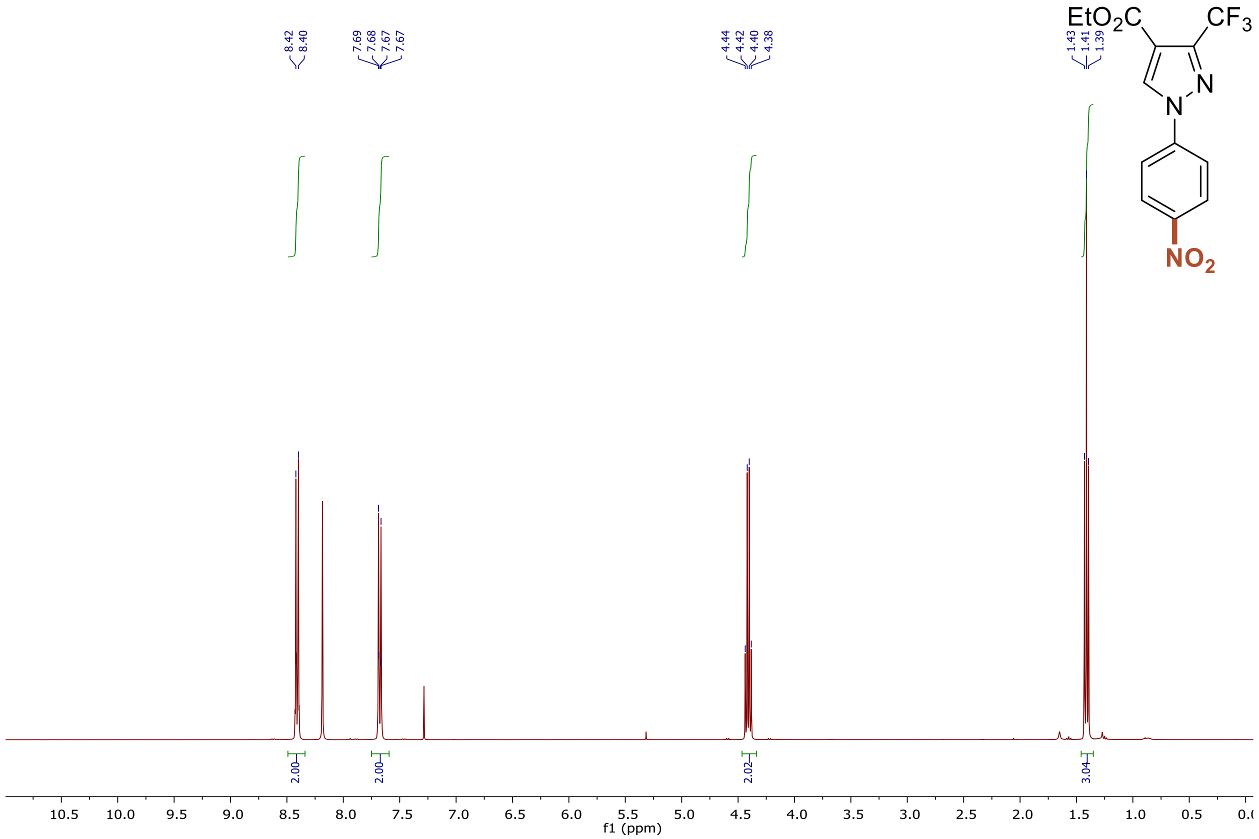

Supplementary Figure 175. ^{1}H NMR spectra for **52.**

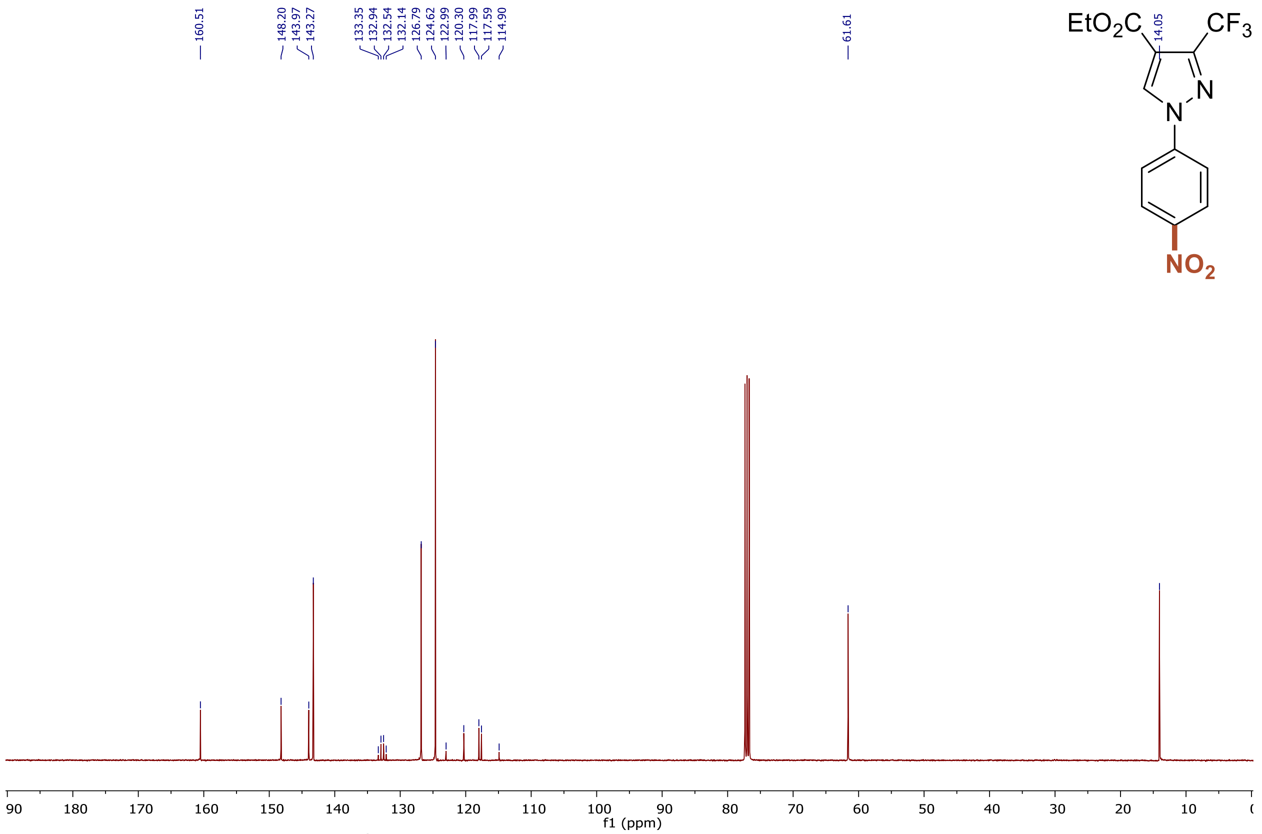

Supplementary Figure 176. ^{13}C NMR spectra for **52.**

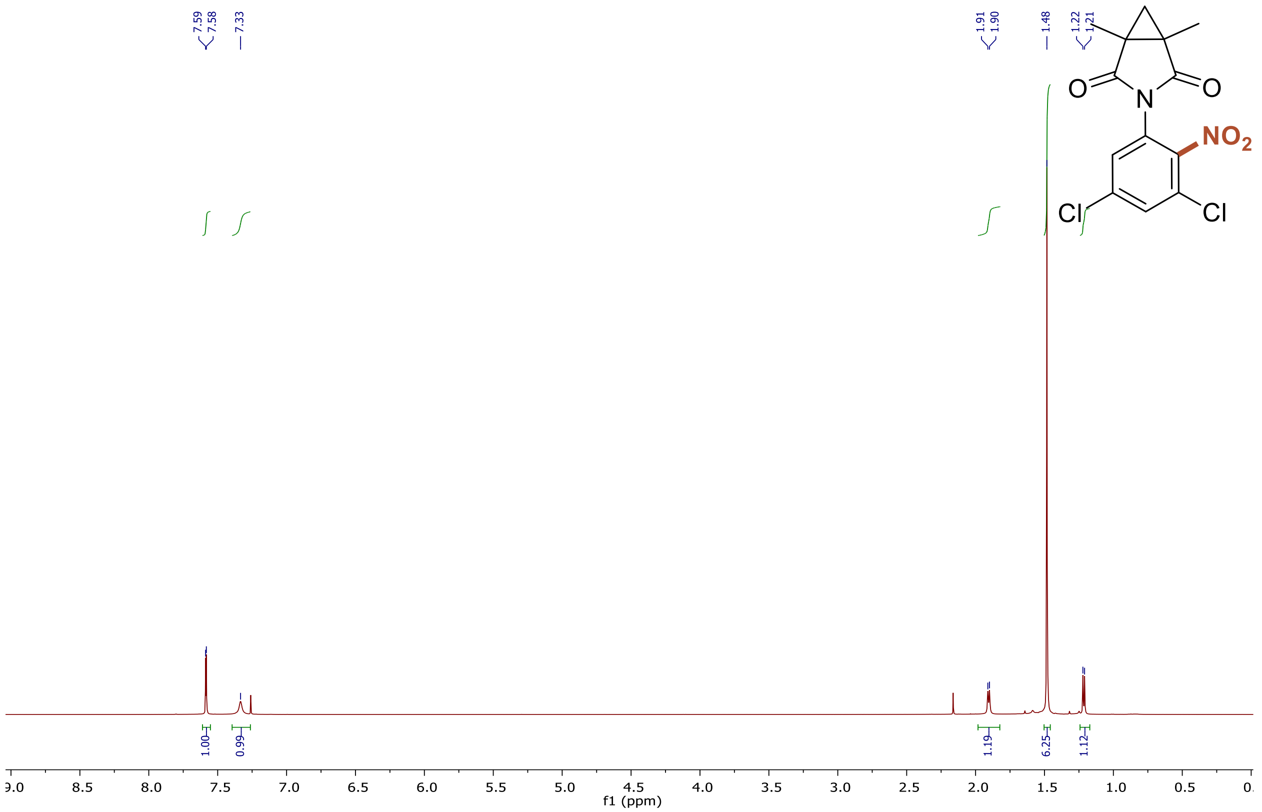

Supplementary Figure 177. ^{1}H NMR spectra for **53.**

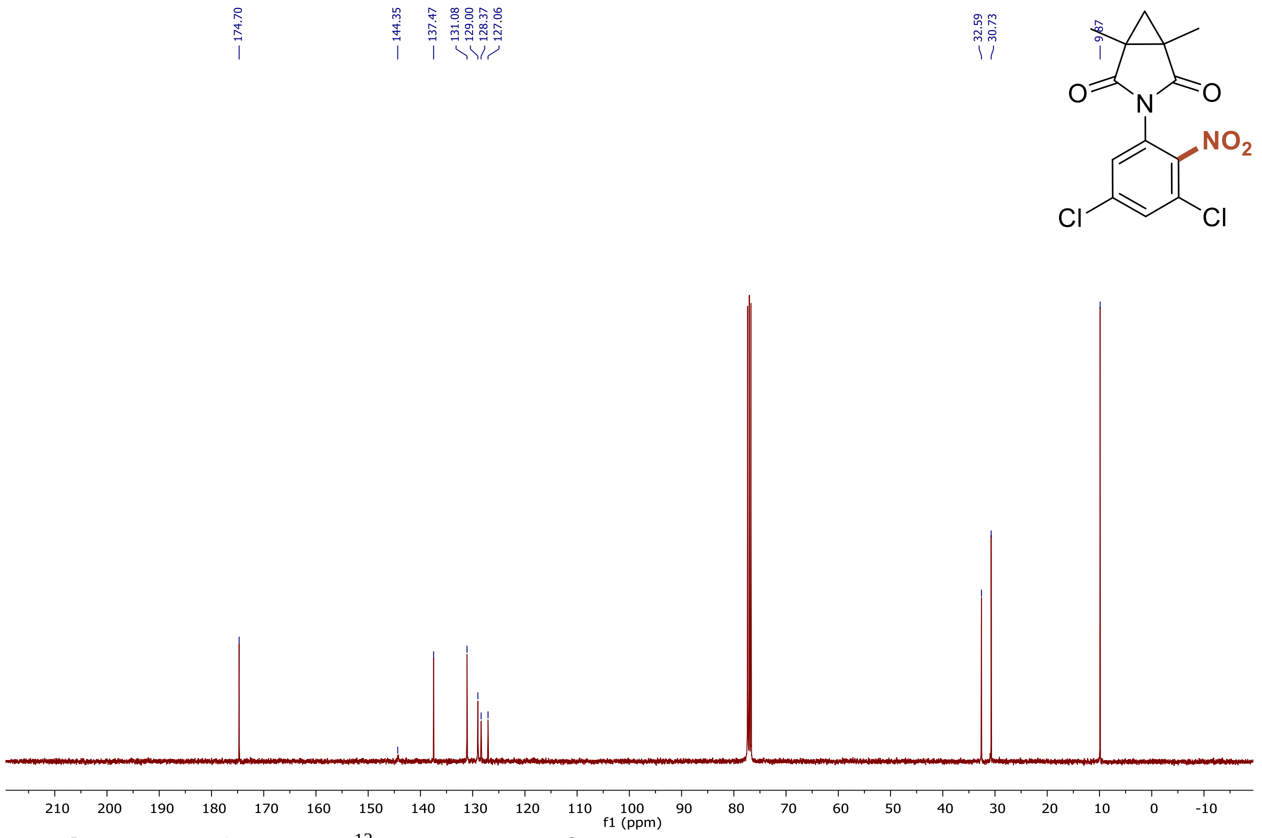

Supplementary Figure 178. ^{13}C NMR spectra for **53.**

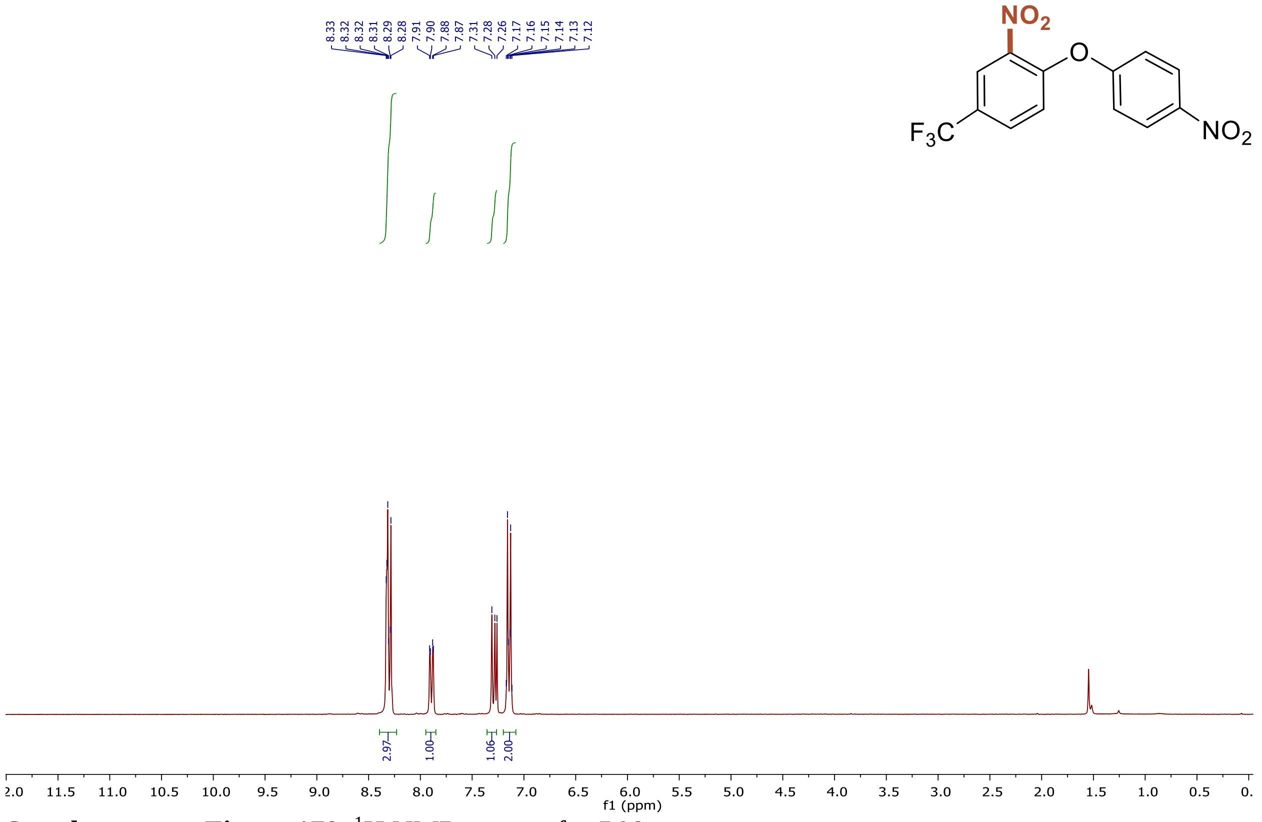

Supplementary Figure 179. ^{1}H NMR spectra for **54A.**

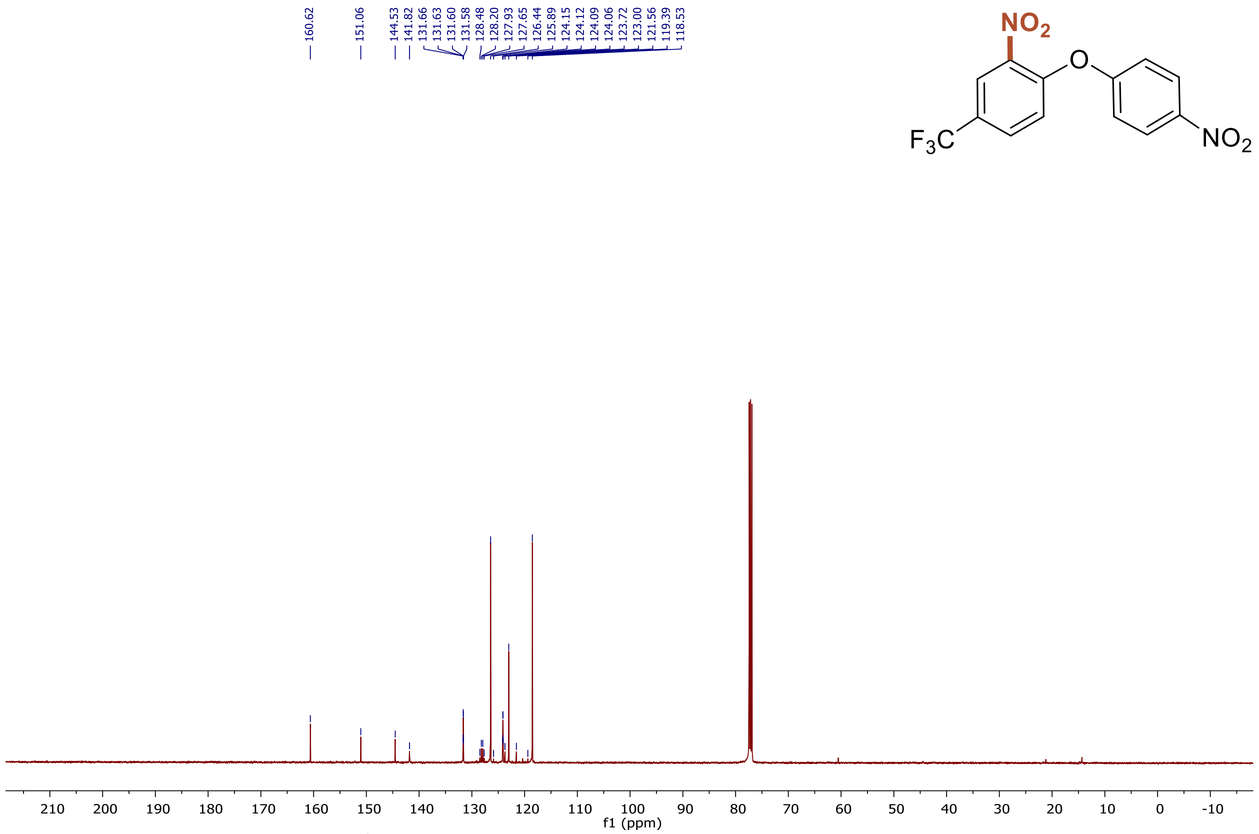

Supplementary Figure 180. ^{13}C NMR spectra for **54A.**

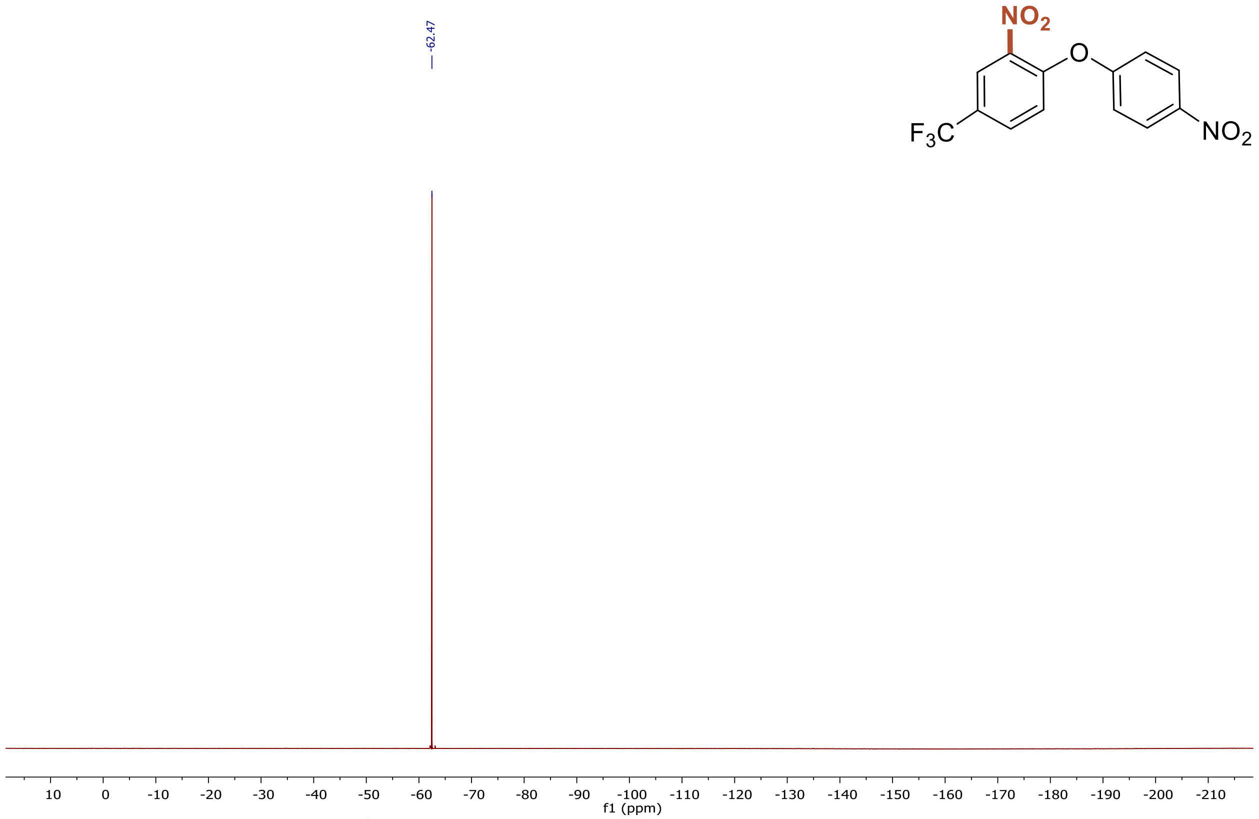

Supplementary Figure 181. ^{19}F NMR spectra for **54A.**

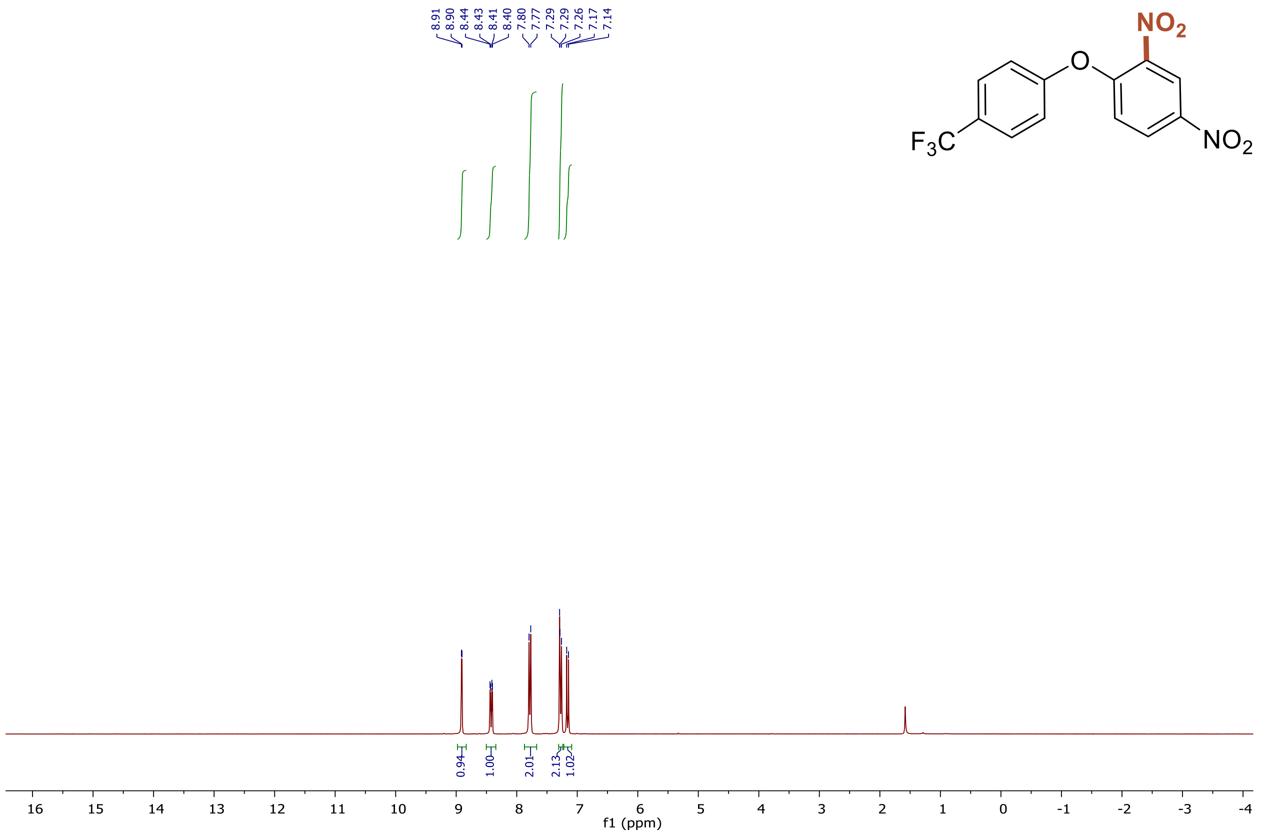

Supplementary Figure 182. ^{1}H NMR spectra for **54B.**

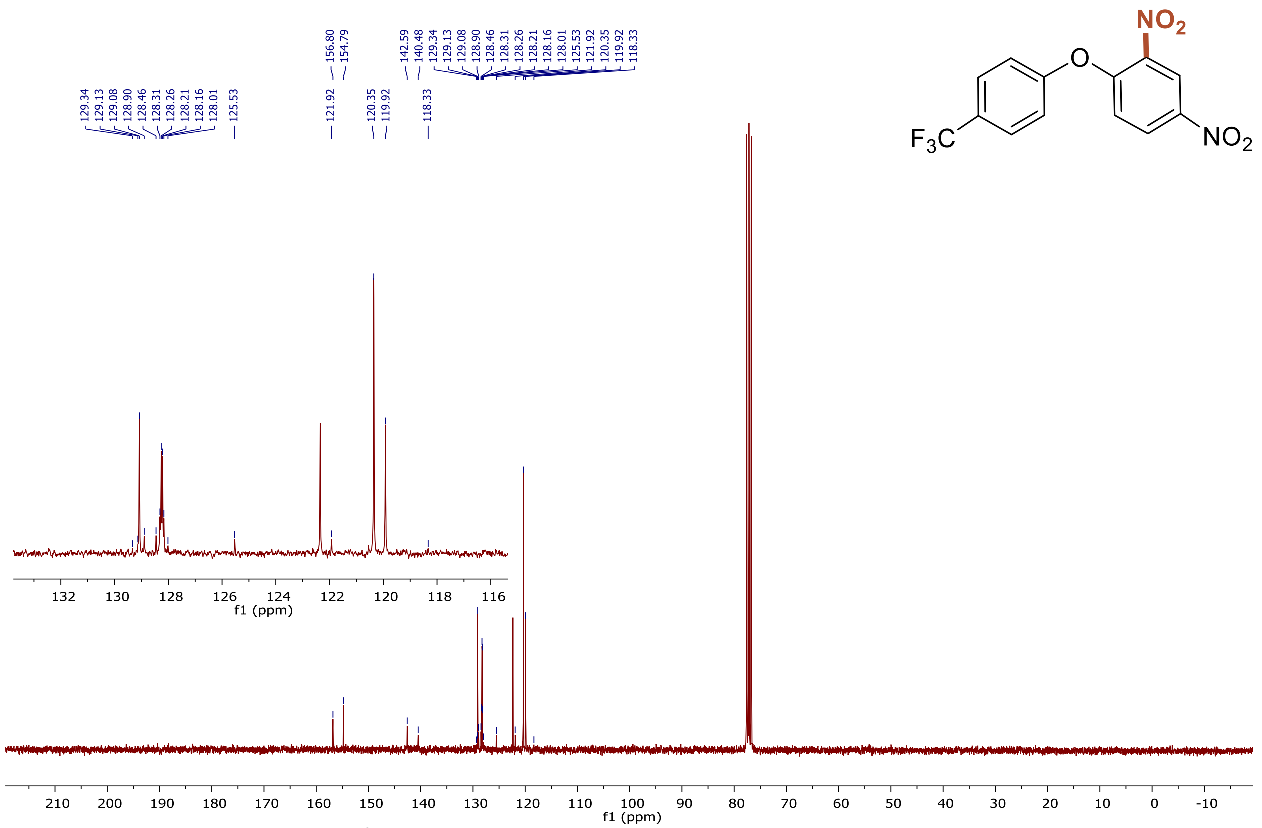

Supplementary Figure 183. ^{13}C NMR spectra for **54B.**

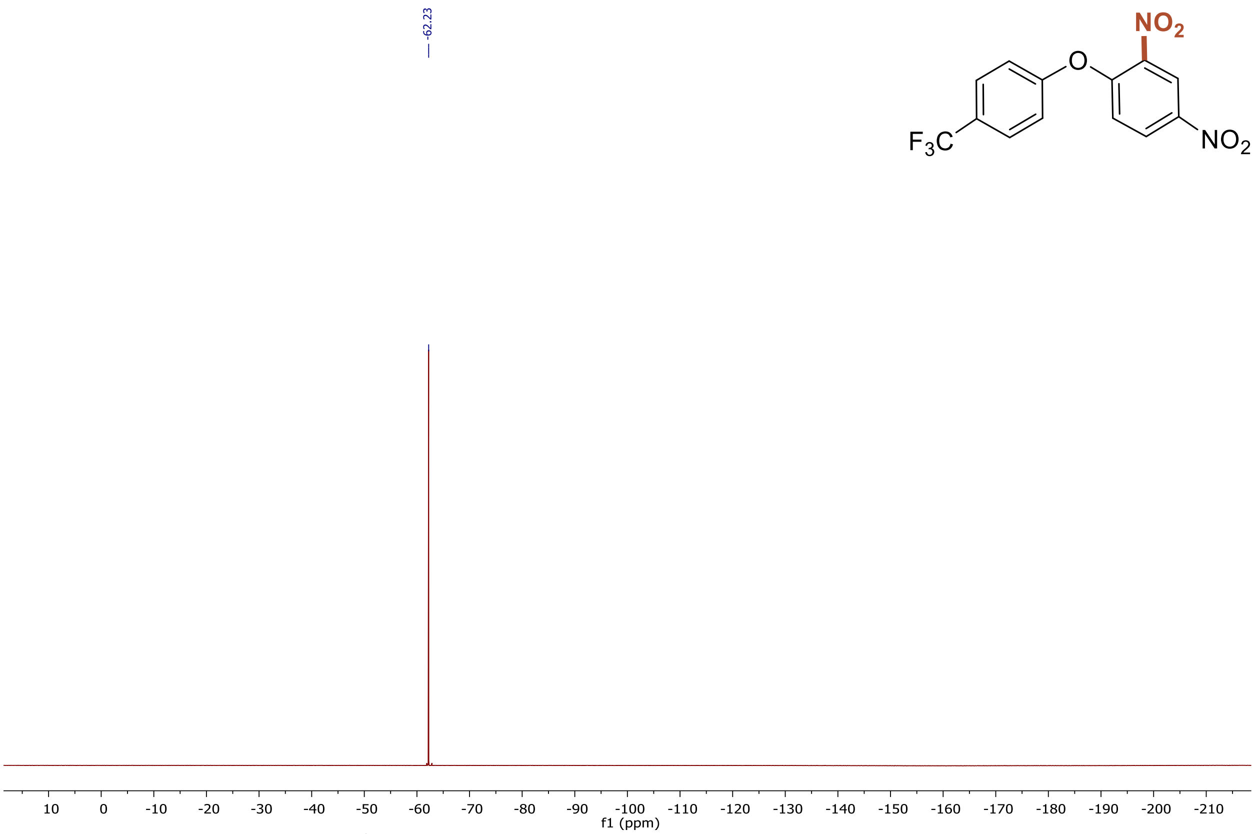

Supplementary Figure 184. ^{19}F NMR spectra for **54B.**

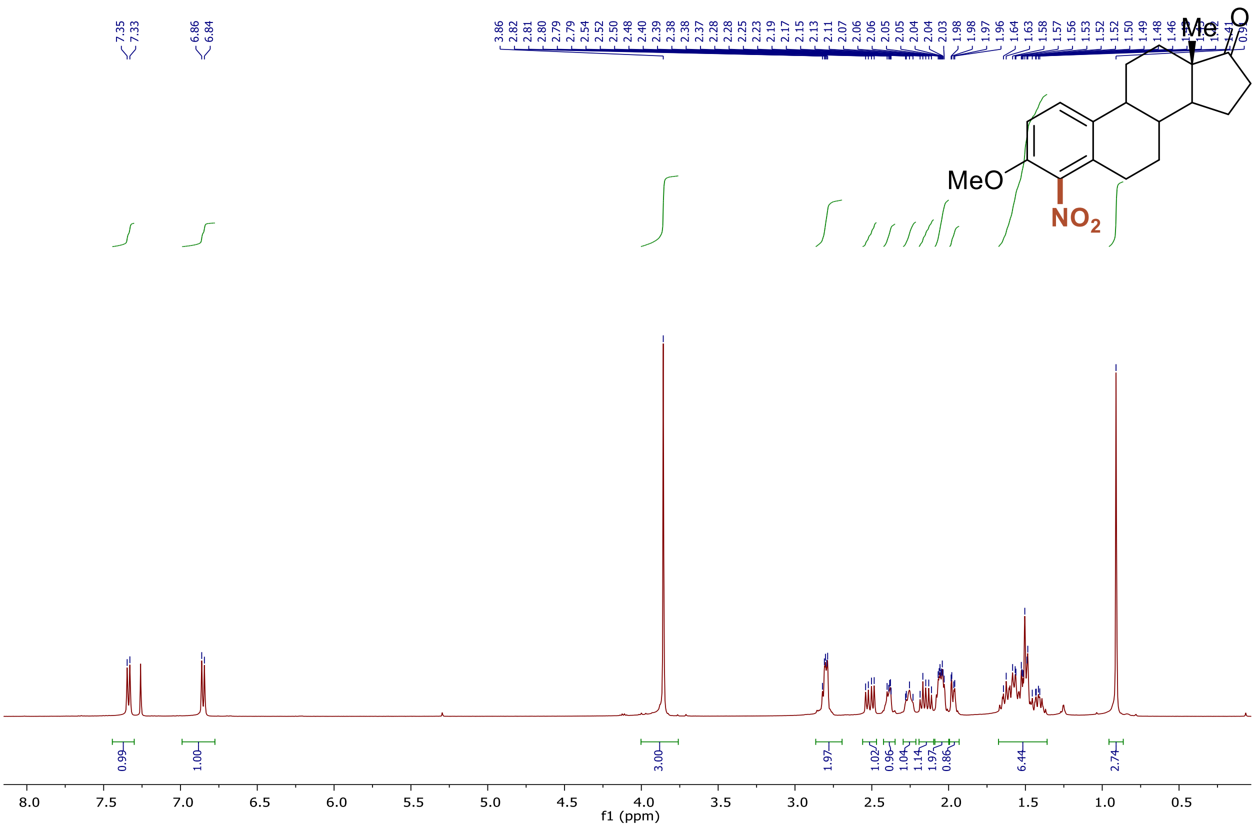

Supplementary Figure 185. ^{1}H NMR spectra for **55A.**

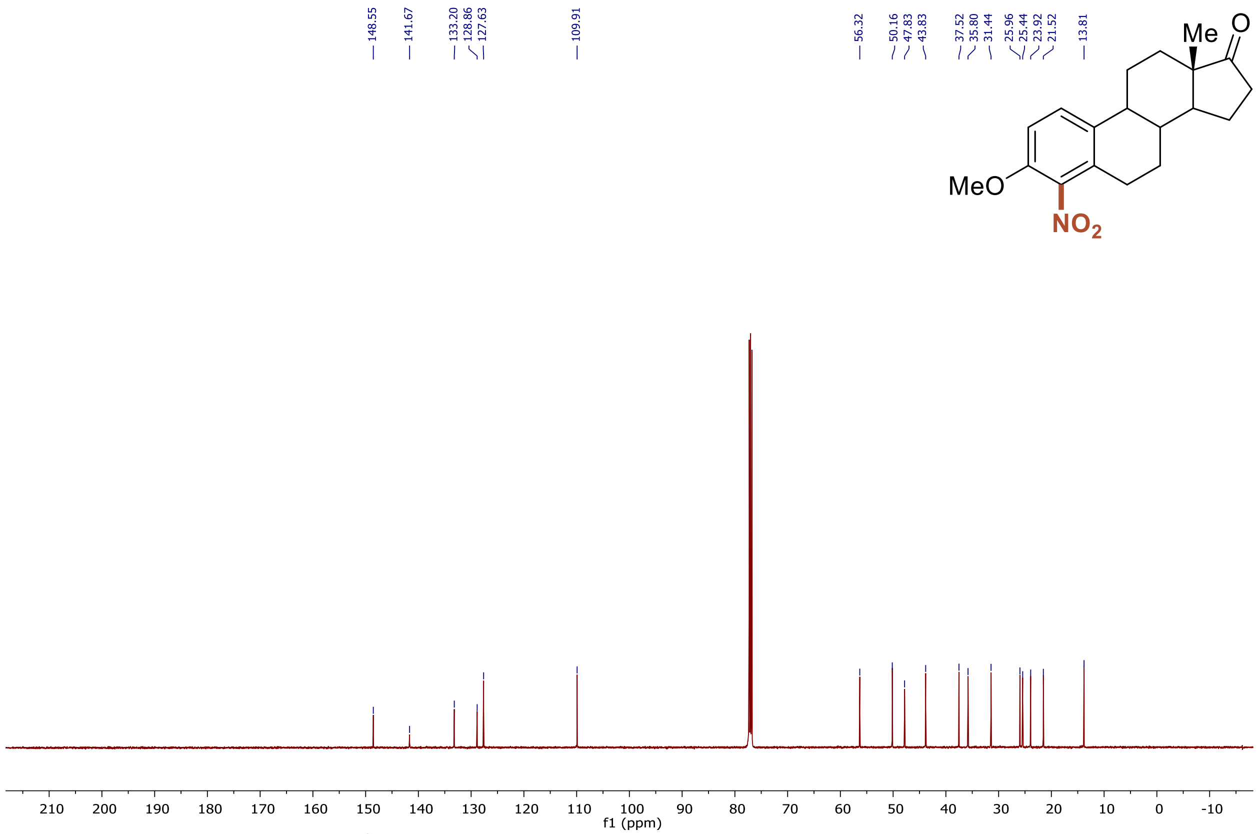

Supplementary Figure 186. ^{13}C NMR spectra for **55A.**

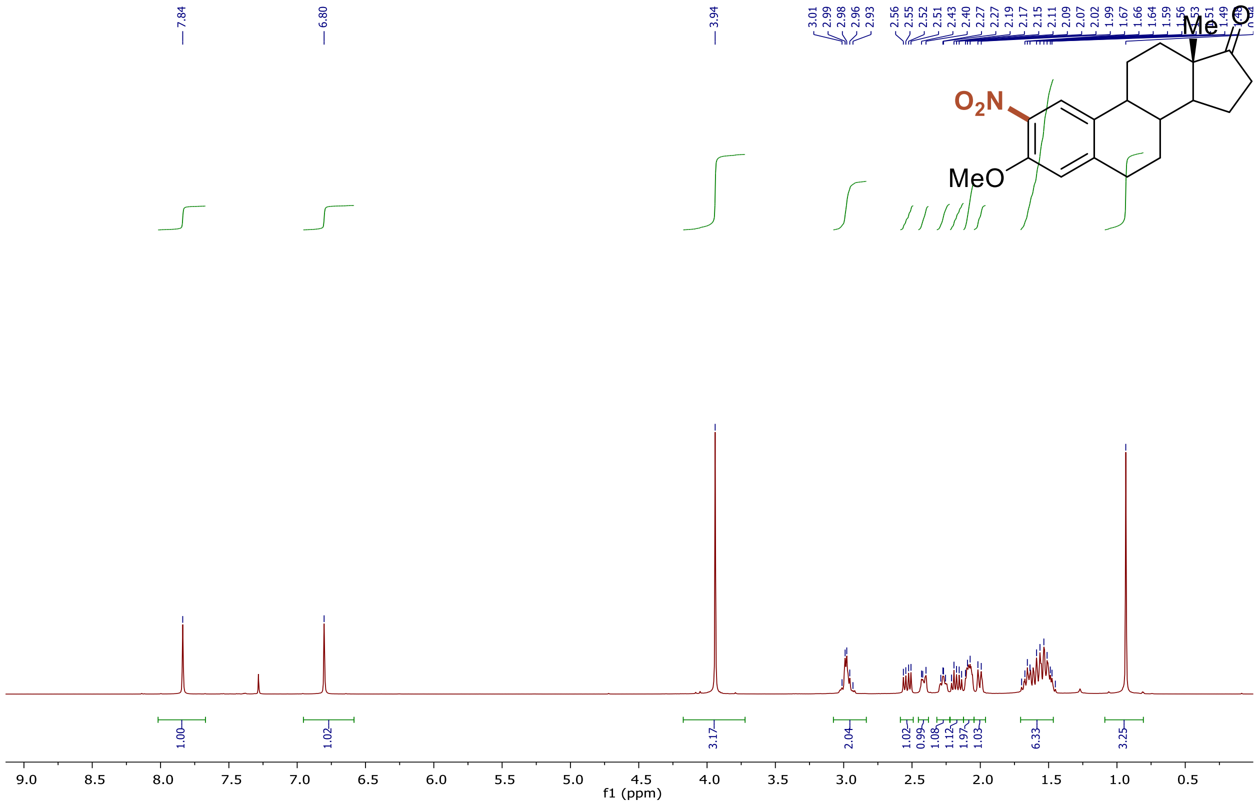

Supplementary Figure 187. ^{1}H NMR spectra for **55B.**

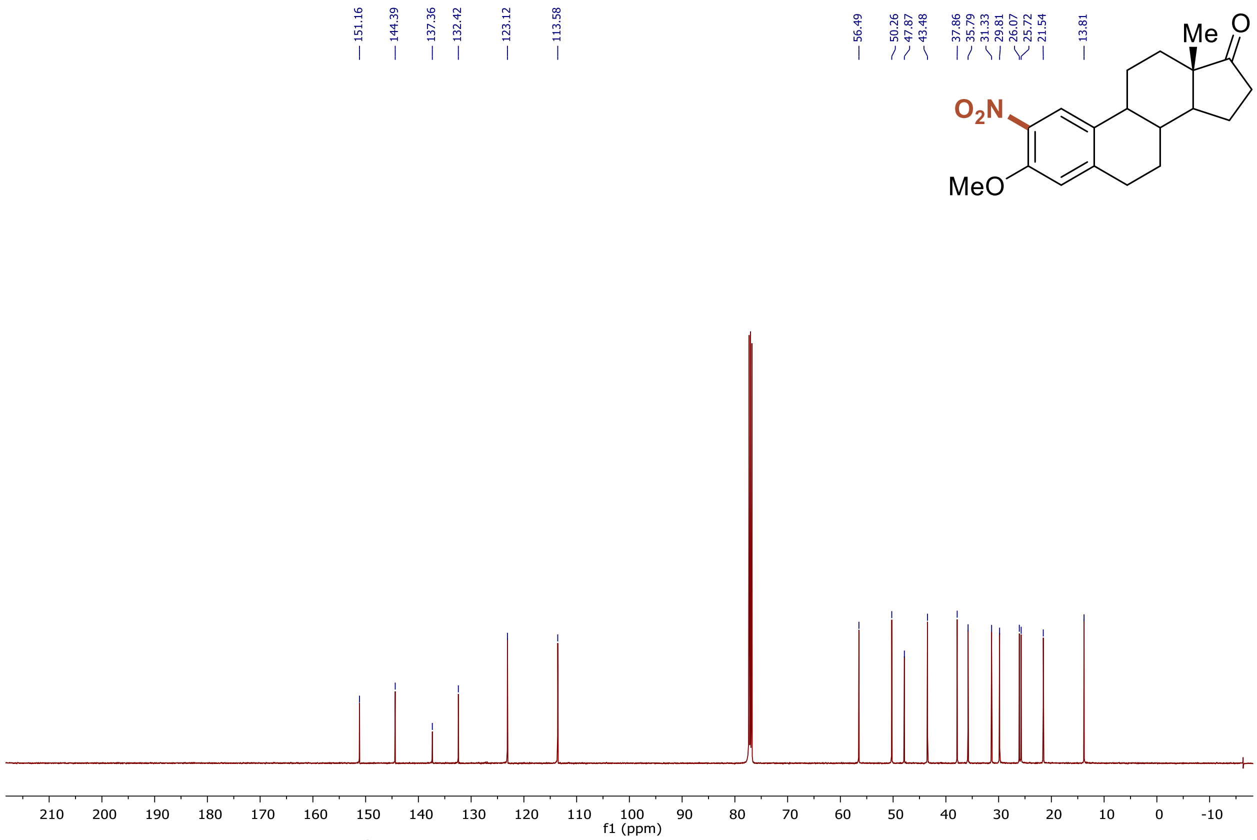

Supplementary Figure 188. ^{13}C NMR spectra for **55B.**

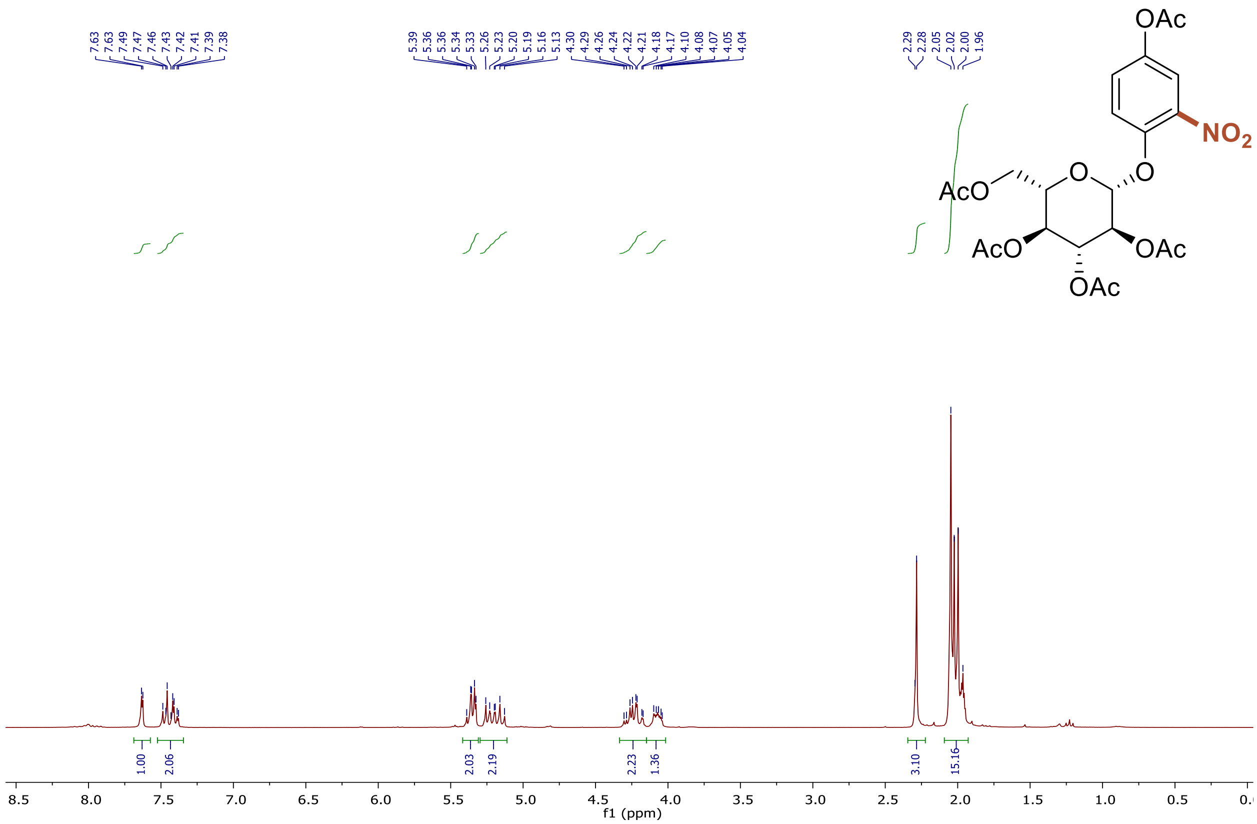

Supplementary Figure 189. ^{1}H NMR spectra for **56.**

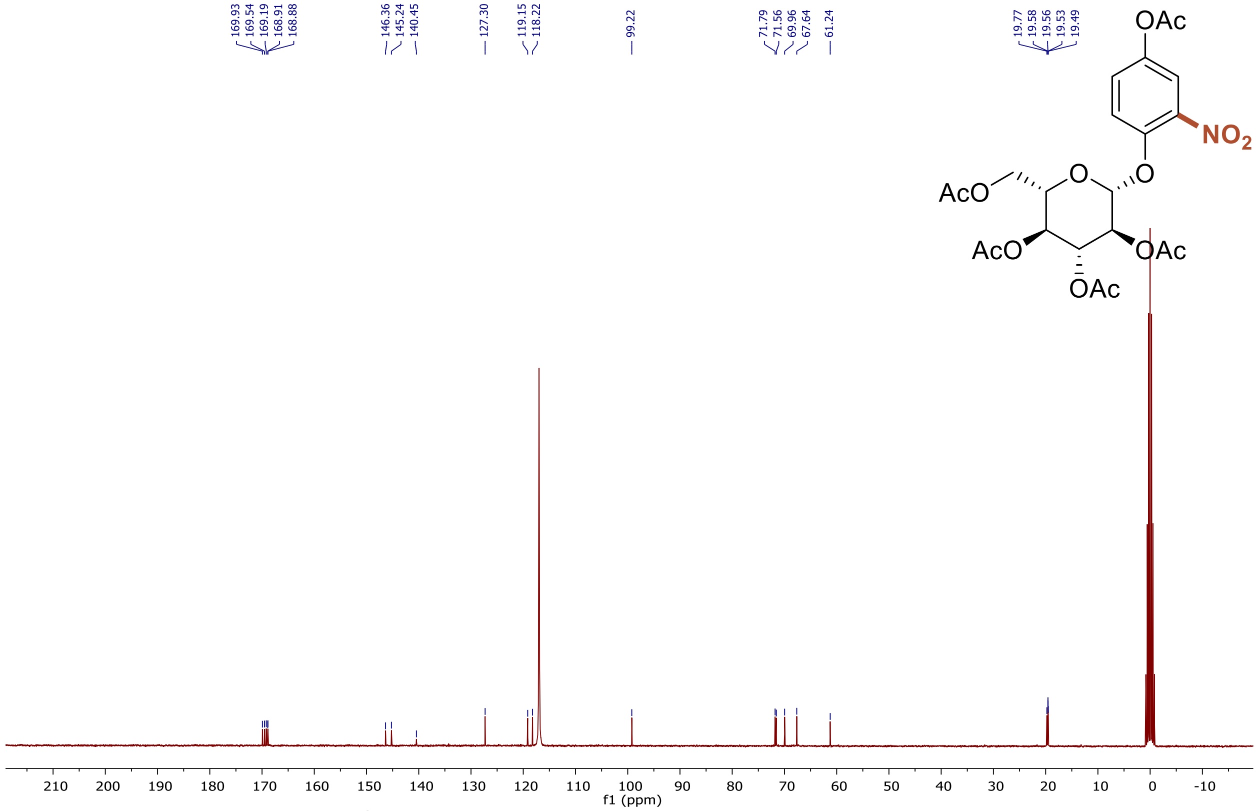

Supplementary Figure 190. ^{13}C NMR spectra for **56.**

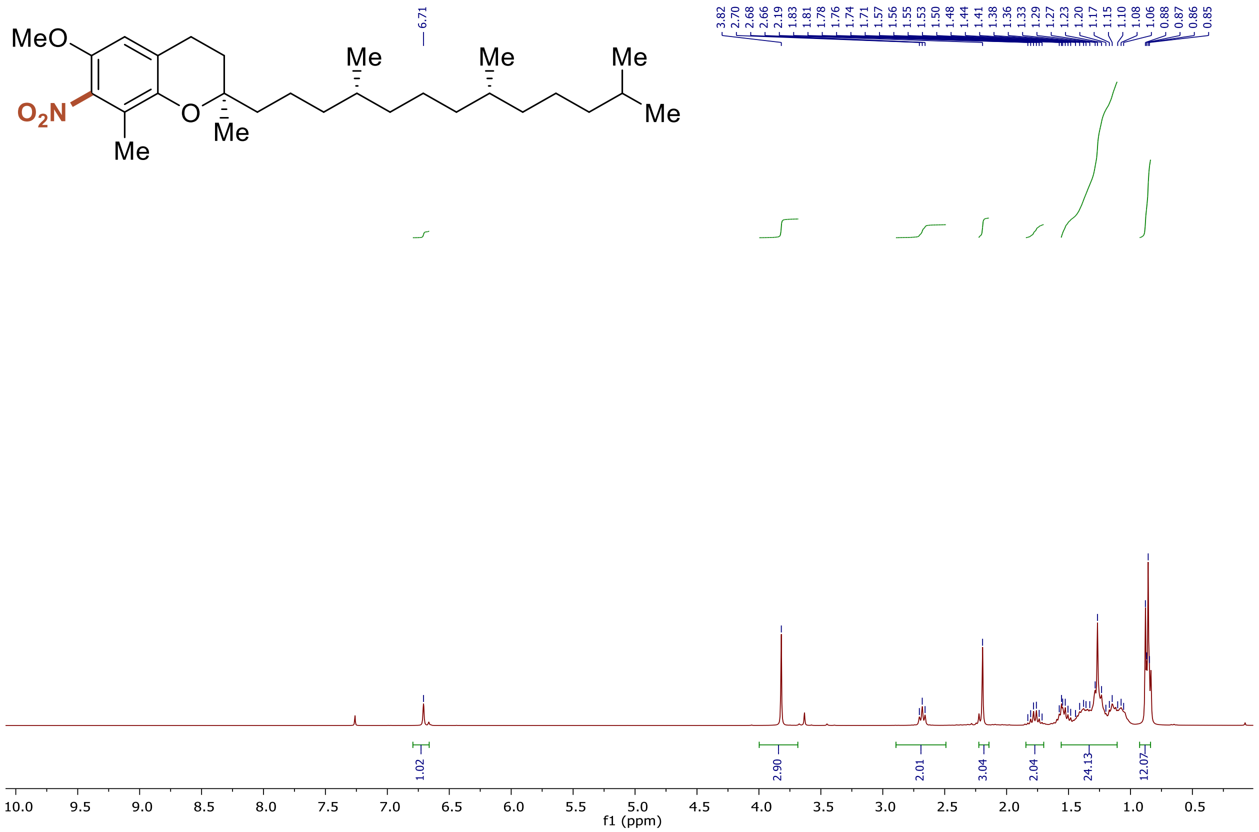

Supplementary Figure 191. ^{1}H NMR spectra for **57.**

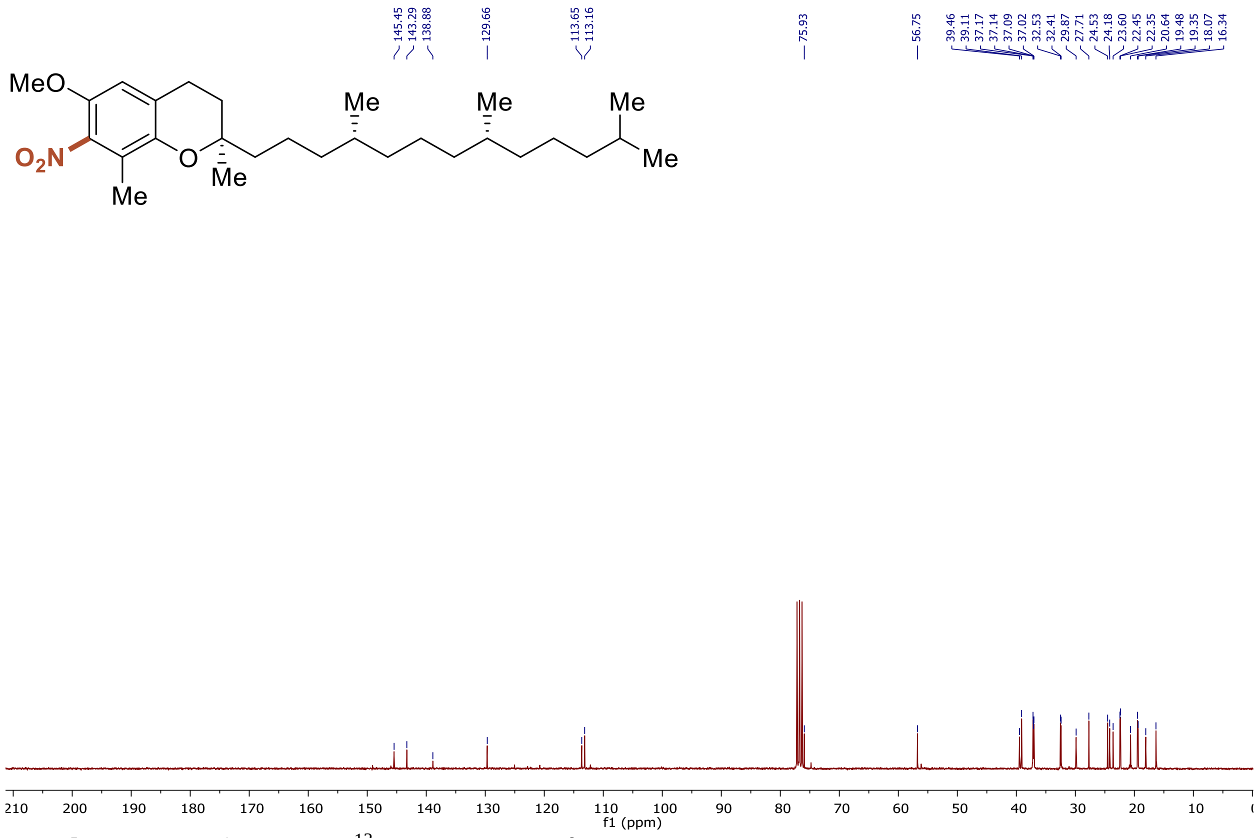

Supplementary Figure 192. ^{13}C NMR spectra for **57.**

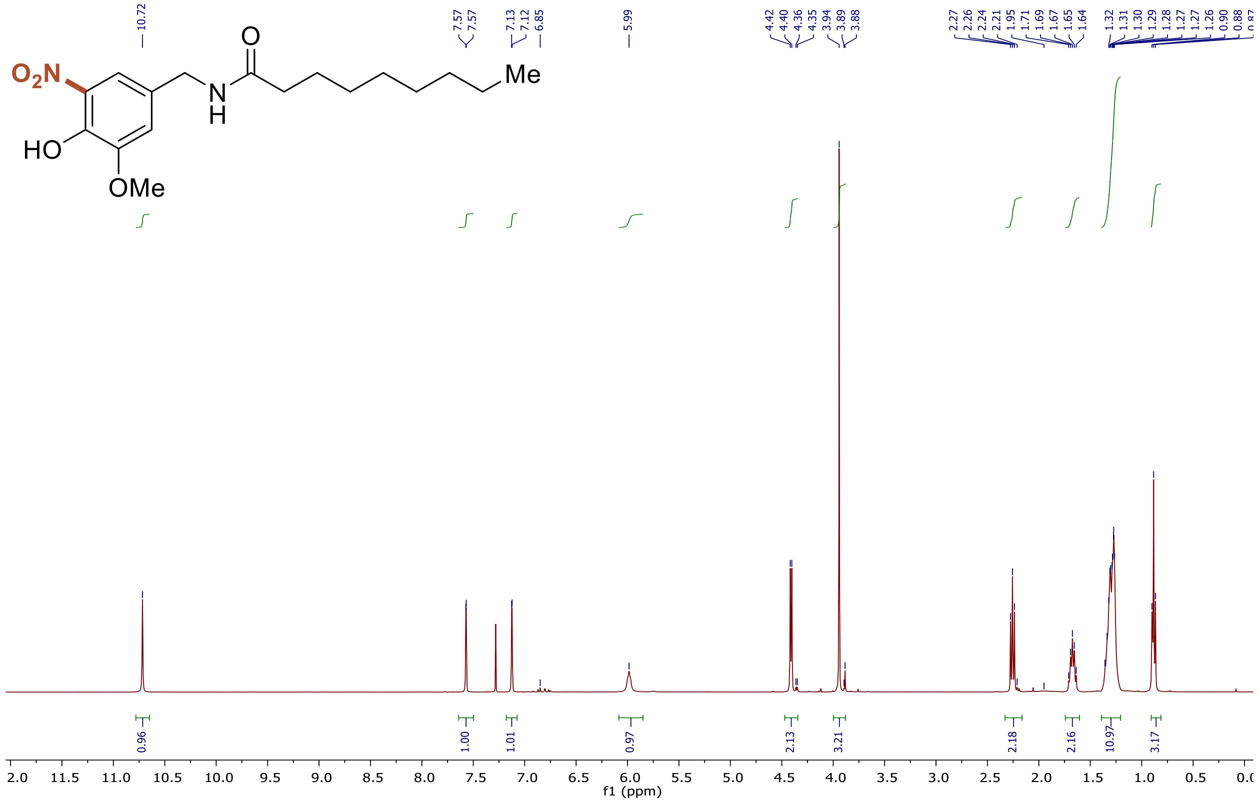

Supplementary Figure 193. ^{1}H NMR spectra for **58.**

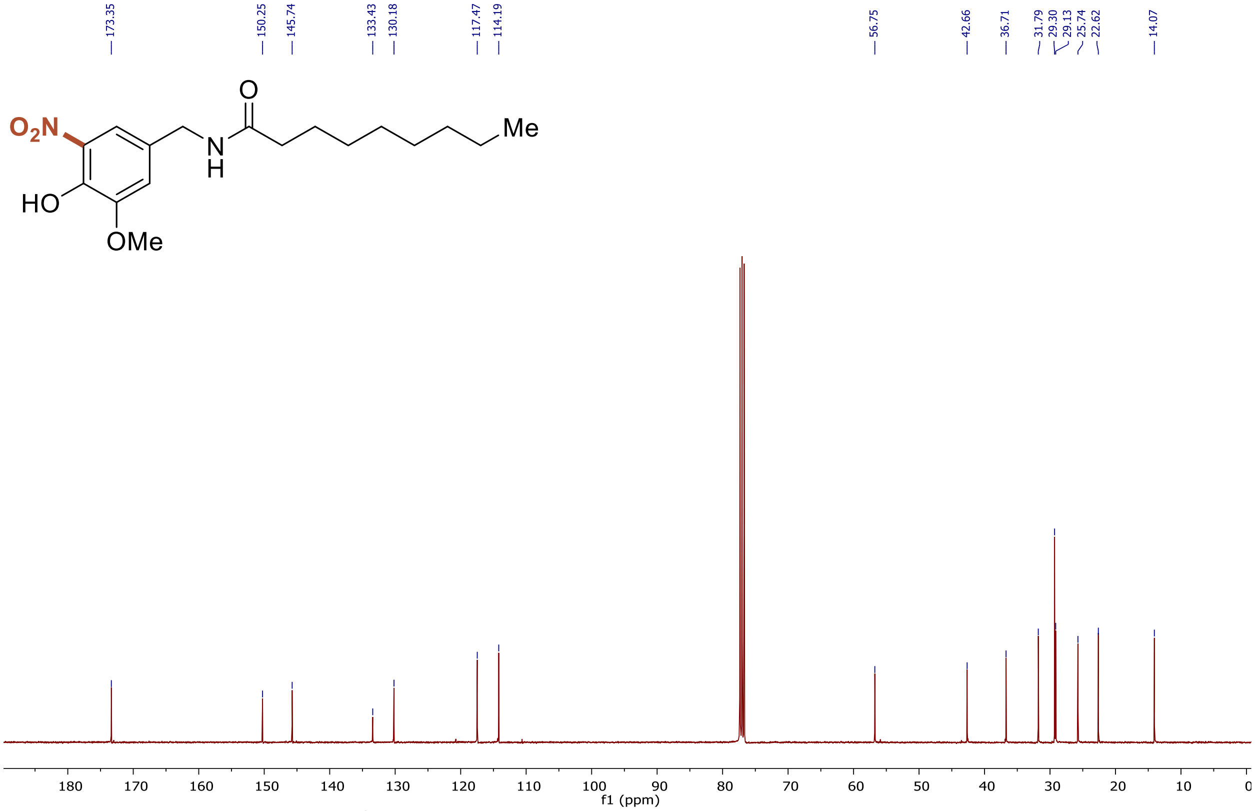

Supplementary Figure 194. ^{13}C NMR spectra for **58.**

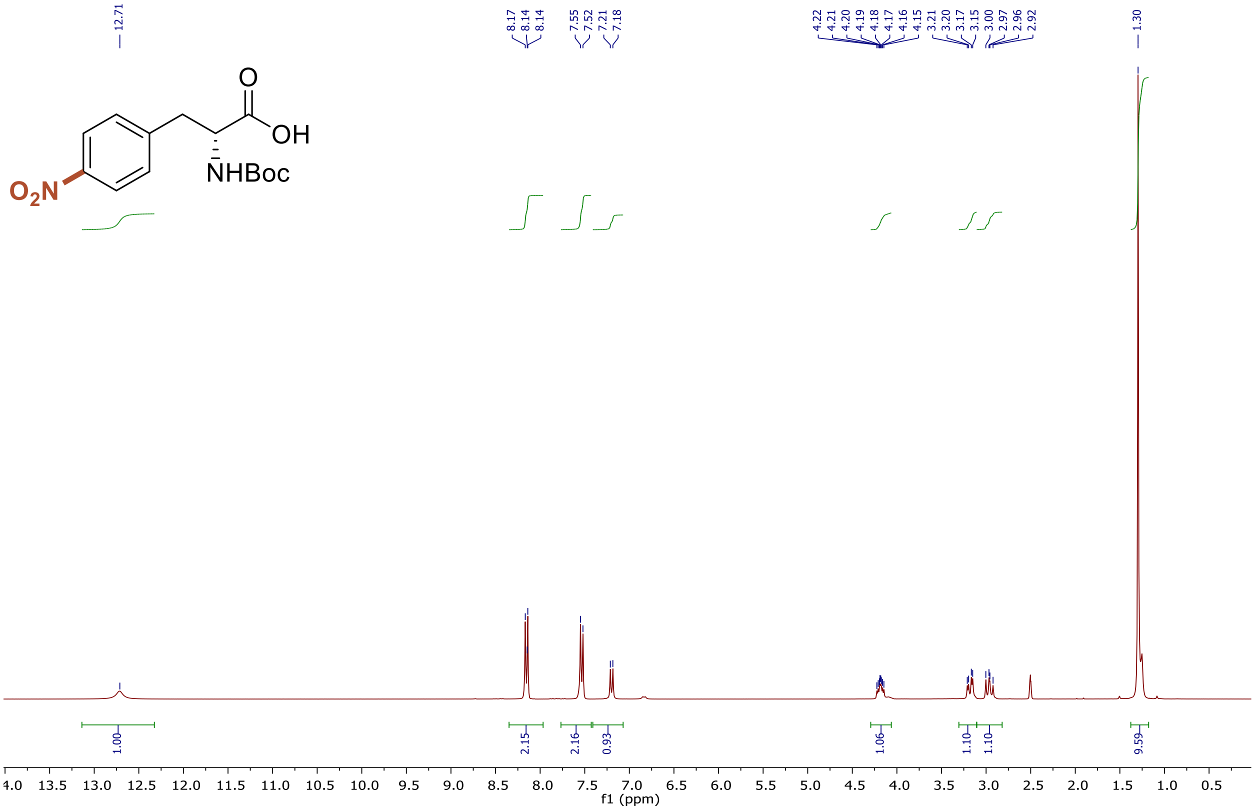

Supplementary Figure 195. ^{1}H NMR spectra for **59A.**

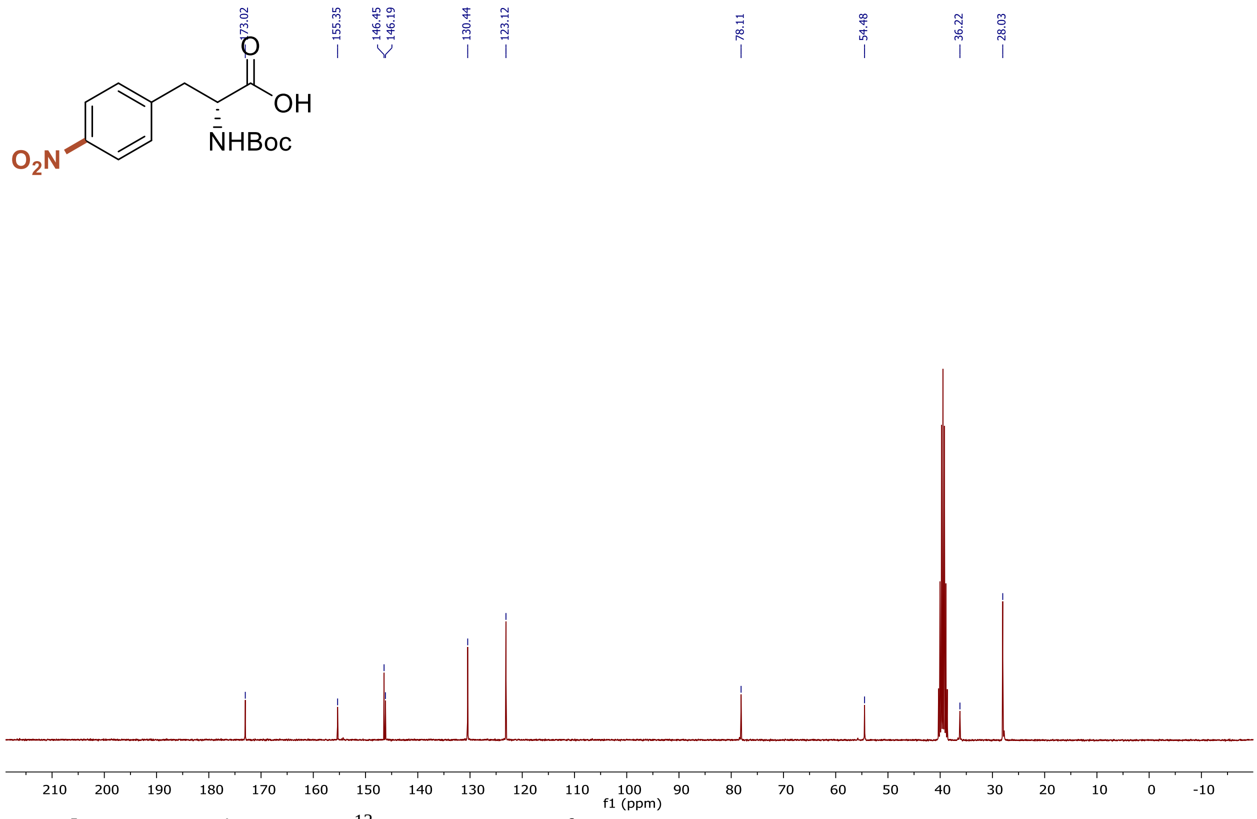

Supplementary Figure 196. ^{13}C NMR spectra for **59A.**

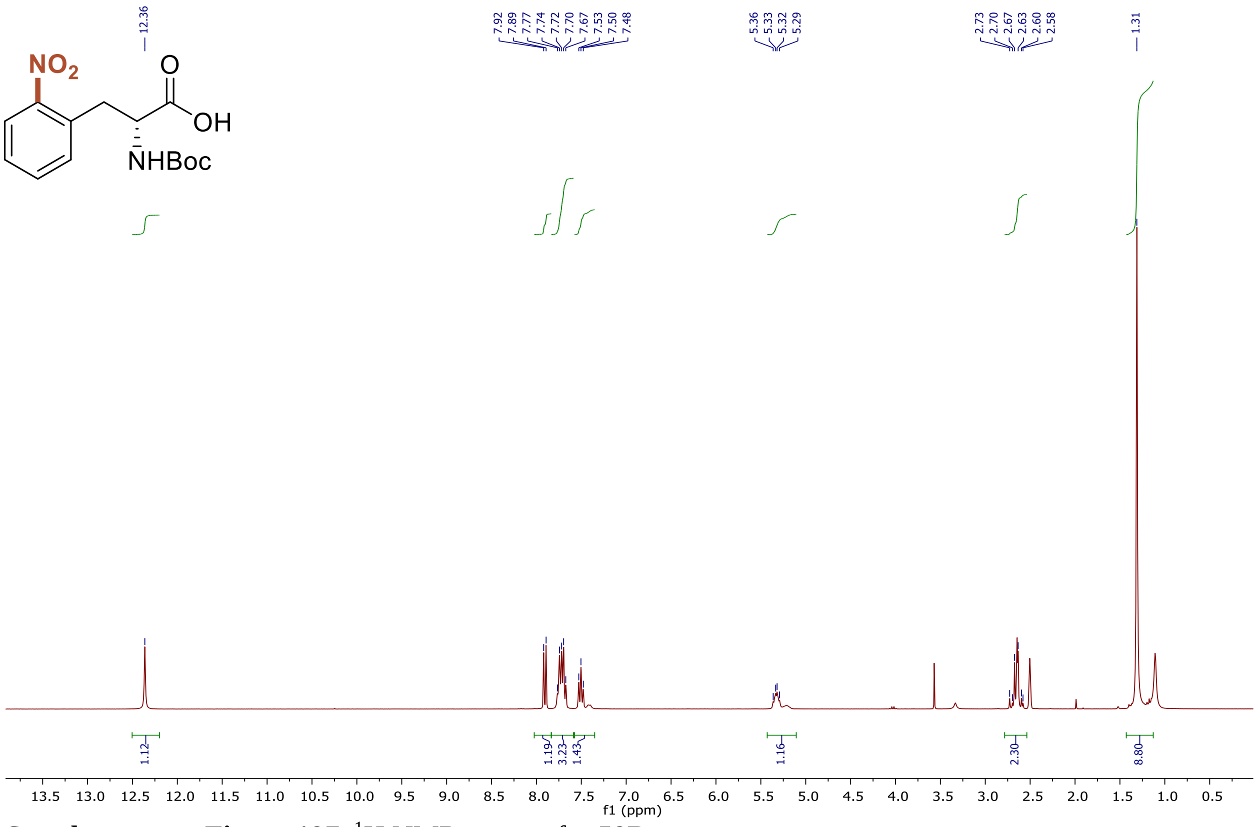

Supplementary Figure 197. ^{1}H NMR spectra for **59B.**

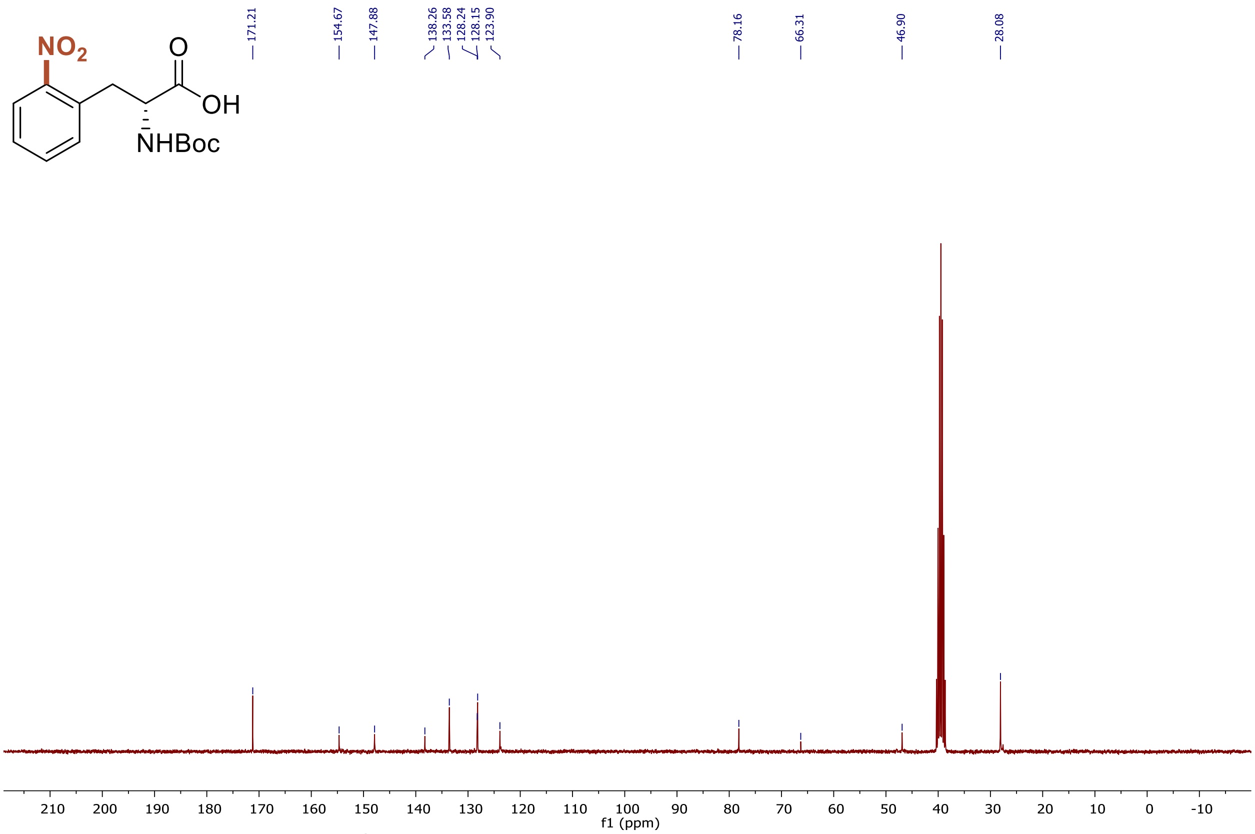

Supplementary Figure 198. ^{13}C NMR spectra for **59B.**

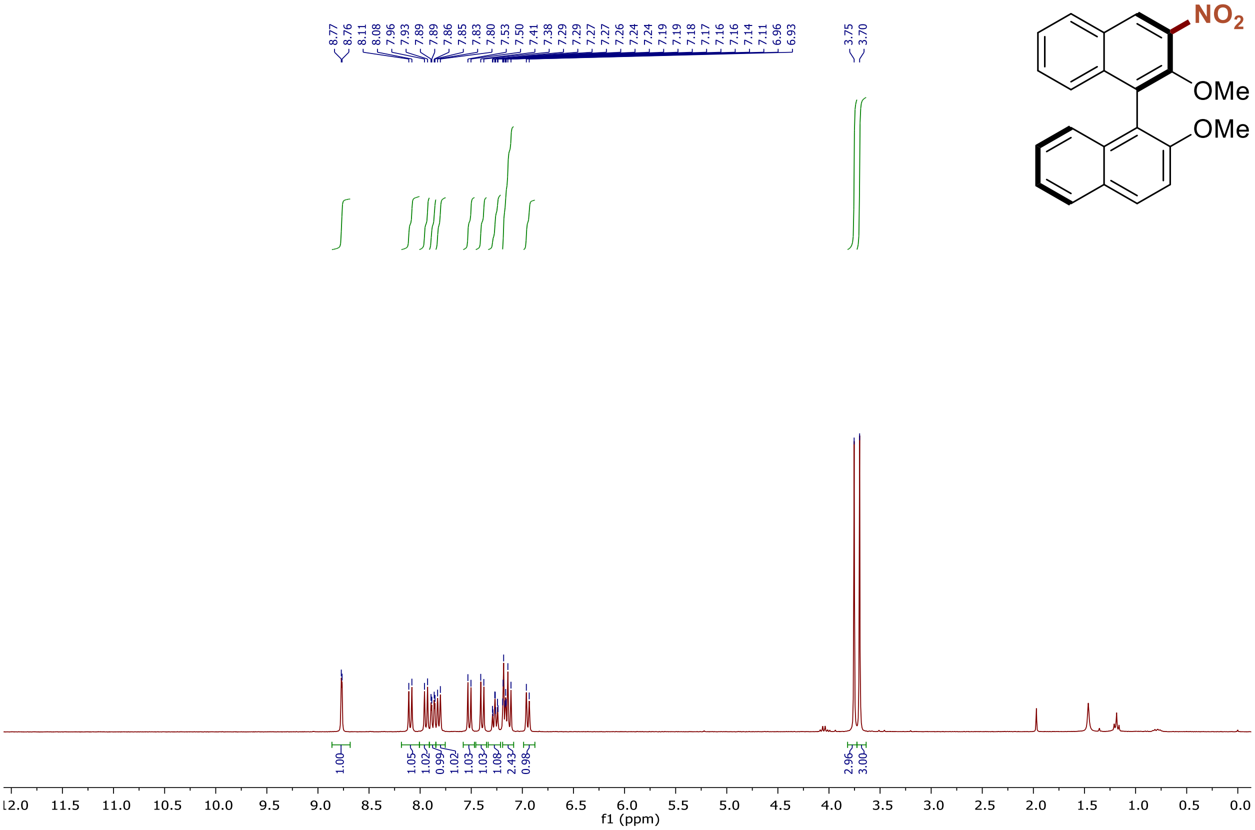

Supplementary Figure 199. ^{1}H NMR spectra for **60.**

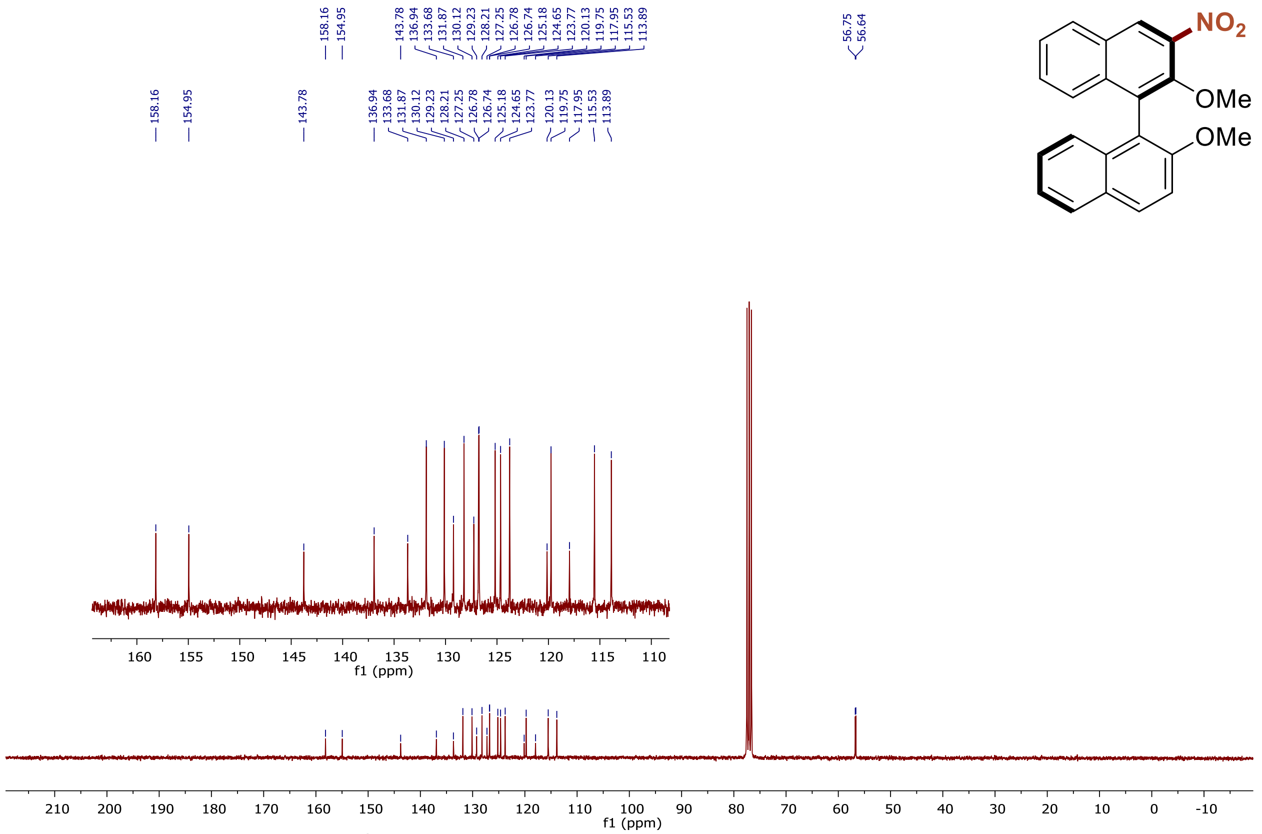

Supplementary Figure 200. ^{13}C NMR spectra for **60.**

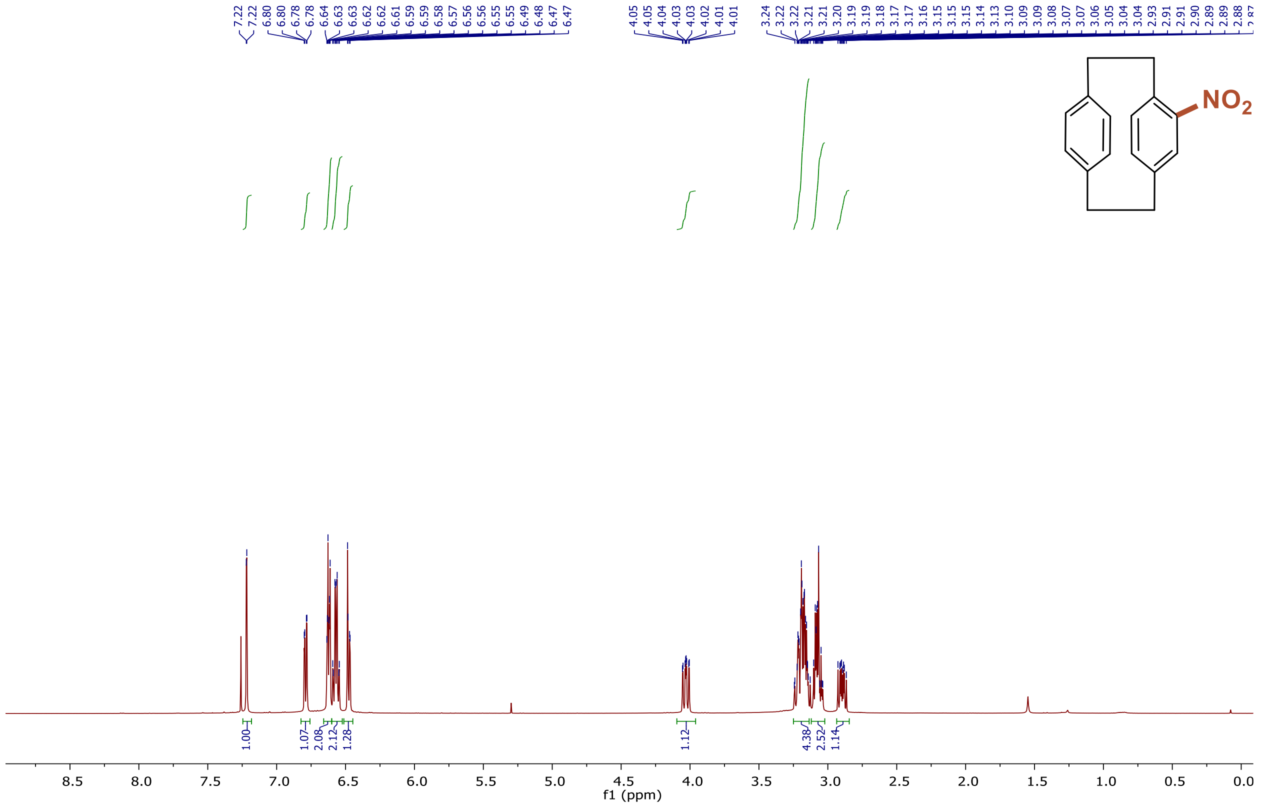

Supplementary Figure 201. ^{1}H NMR spectra for **61.**

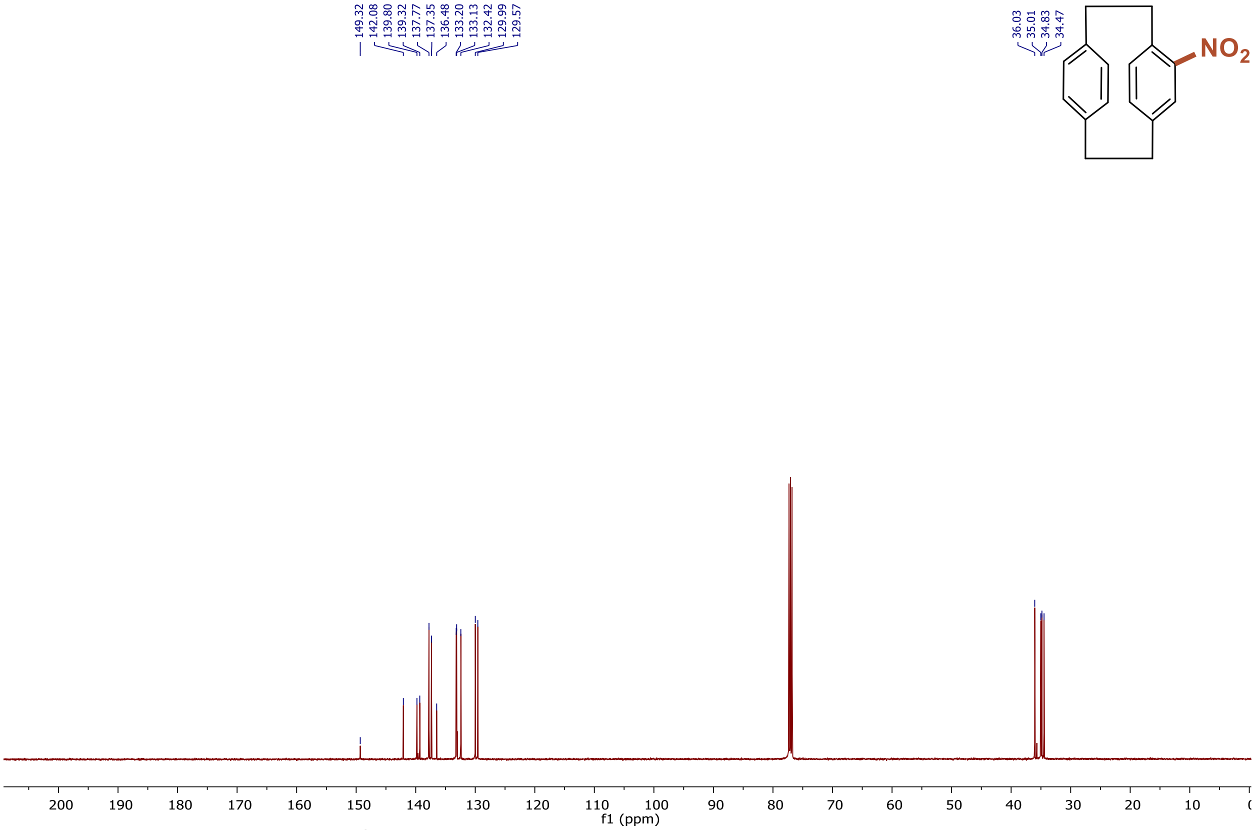

Supplementary Figure 202. ^{13}C NMR spectra for **61.**

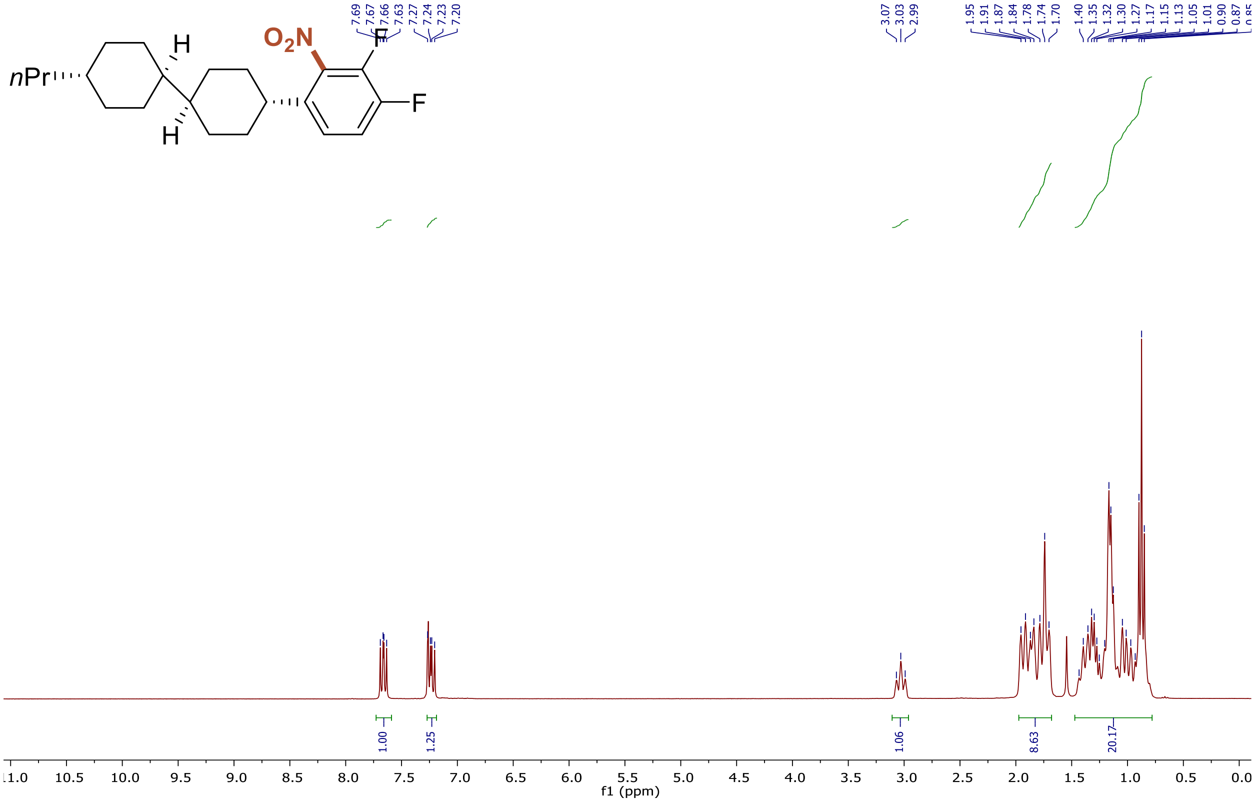

Supplementary Figure 203. ^{1}H NMR spectra for **62.**

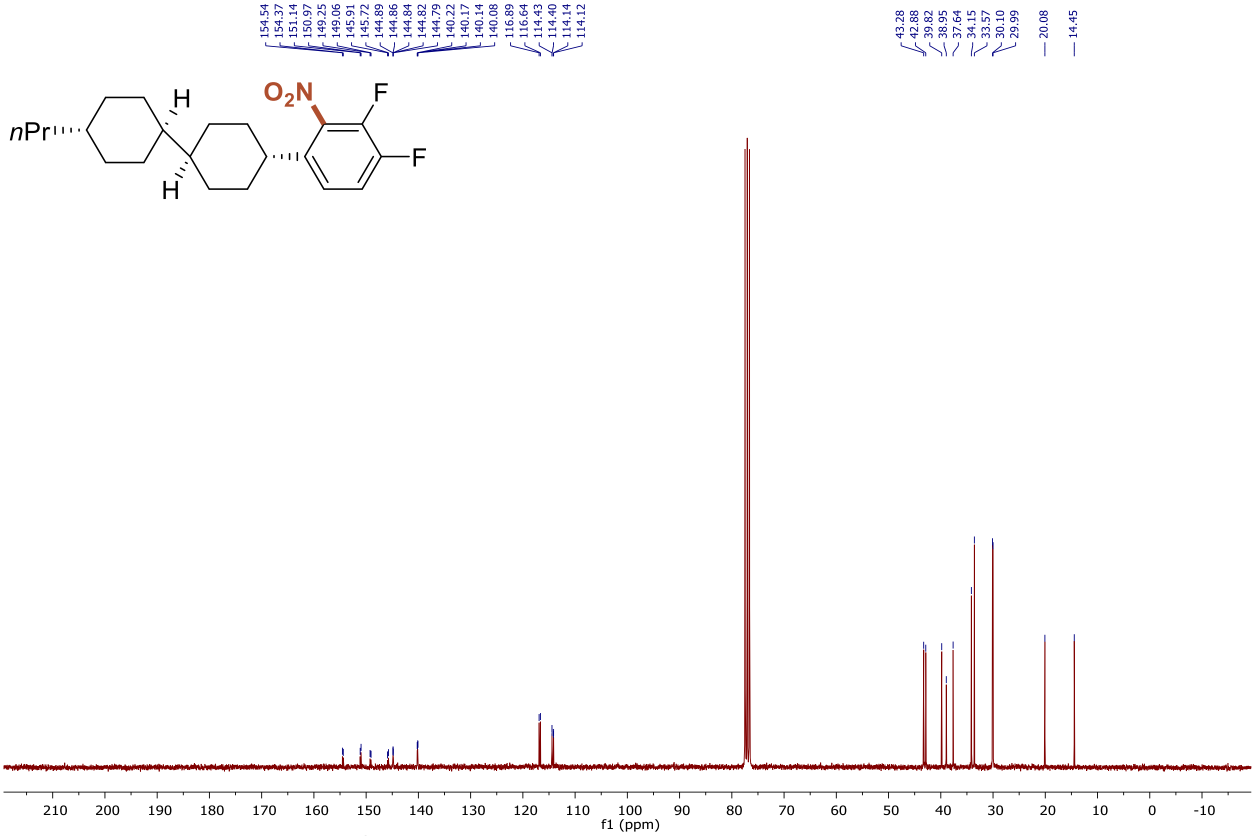

Supplementary Figure 204. ^{13}C NMR spectra for **62.**

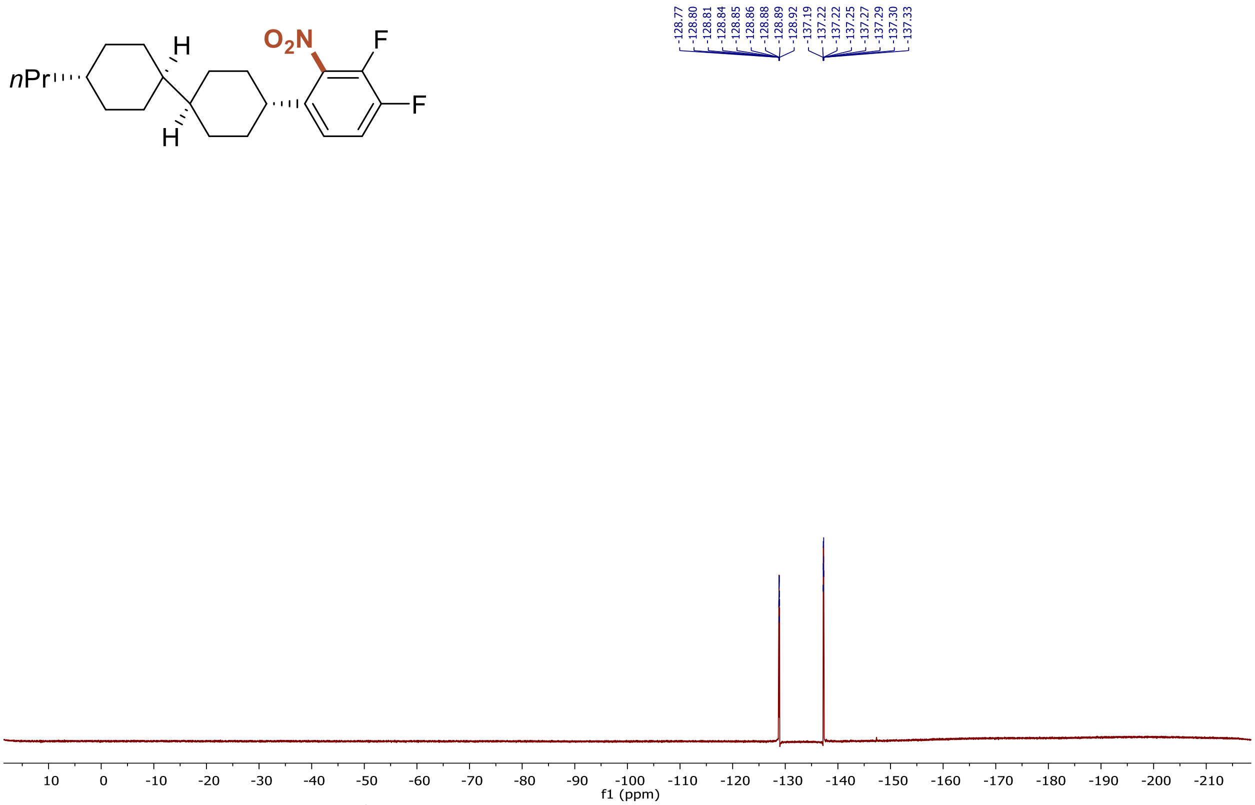

Supplementary Figure 205. ^{19}F NMR spectra for **62.**

Supplementary References

(1) Esteves, P. M., de M. Carberio, J. W., Cardoso, S. P., Barbosa, A. G. H., Laali, K. K., Prakash, G. K. S. & Olah, G. A. Unified mechanistic concept of electrophilic aromatic nitration: Convergence of computational results and experimental data (2003), *J. Am. Chem. Soc.* **125**, 4836-4849 (2003).

(2) Booth, G. in *Ullmann's Encyclopedia of Industrial Chemistry* (Wiley-VCH Verlag GmbH & Co. KGaA, Weinheim, Germany, 2000).

(3) N. Ono, *The Nitro group in organic synthesis* (John Wiley & Sons, Inc., New York, USA, 2001) *Wiley Series in Organic Nitro Chemistry*.

(4) Kulkarni, A. A. Continuous flow nitration in miniaturized devices. *Beilstein J. Org. Chem.* **10**, 405–424 (2014).

(5) Greish, A. Nitration of aromatic hydrocarbons over heterogenous catalysts. *Rus. Chem. J.* **48**, 92–104 (2004).

(6) Koskin, A. P., Mishakov, I. V. & Vedyagin, A. A. In search of efficient catalysts and appropriate reaction conditions for gas phase nitration of benzene. *Resour. Technol.* **2**, 118–125 (2016).

(7) Hughes, E. D., Ingold, C. K. & Reed, R. I. Kinetics of aromatic nitration : The Nitronium ion. *Nature* **158**, 448–449 (1946).

(8) Halberstadt, E. S., Hughes, E. D. & Ingold, C. K. Kinetics of aromatic nitration : The Nitracidium ion. *Nature* **158**, 514–514 (1946).

(9) Olah, G. A., Malhotra, R. & Narang, S. C. *Nitration: Methods and mechanisms* (VCH Publishers, Inc, New York, USA, 1989).

(10) Yan, G. & Yang, M. Recent advances in the synthesis of aromatic nitro compounds. *Org. Biomol. Chem.* **11**, 2554−2566 (2013).

(11) Prakash, G. K. S., Panja, C., Matthwer, T., Surampudi, V., Petasis, N. A. & Olah, G. A. Ipso-Nitration of arylboronic acids with chlorotrimethylsilane−nitrate salts. *Org. Lett.* **6**, 2205-2207 (2004).

(12) Fors, B. P. & Buchwald, S. L. Pd−Catalyzed conversion of aryl chlorides, triflates, and nonaflates to nitroaromatics. *J. Am. Chem. Soc.* **131**, 12898–12899 (2009).

(13) Song, L.-R., Fan, Z. & Zhang, A. Recent advances in transition metal-catalyzed C(sp^2)–H nitration. *Org. Biomol. Chem.* **17**, 1351–1361 (2019).

(14) Rozen, S. & Carmeli, M. From azides to nitro compounds in a few seconds using $HOF \cdot CH_3CN$. *J. Am. Chem. Soc.* **125**, 8118-8119 (2003).

(15) Reddy, K. R., Maheswari, C. U., Venkateshwar, M. & Kantam, M. L. Selective oxidation of aromatic amines to nitro derivatives using potassium iodide-tert-butyl hydroperoxide catalytic system. *Adv. Synth. Catal.* **351**, 93–96 (2009).

(16) Kilpatrick, B., Heller, M. & Arns, S. Chemoselective nitration of aromatic sulfonamides with tert-butyl nitrite. *Chem. Commun.* **49**, 514–516 (2013).

(17) Wu, X.-F., Schranck, J., Neumann, H. & Beller, M. Convenient and mild synthesis of nitroarenes by metal-free nitration of arylboronic acids. *Chem. Commun.* **47**, 12462 (2011).

(18) Olah, G. A., Narang, S. C., Olah, J. A. & Lammertsma, K. Recent aspects of nitration: New preparative methods and mechanistic studies. *Proc. Natl. Acad. Sci.* **79**, 4487–4494 (2006).

(19) Coburn, M. D. & Ungnade, H. E. Synthesis and structure of the N-nitropyrrolidinones. *J. Heterocycl. Chem.* **2**, 308–309 (1965).

(20) Kauffman, H. F. & Burger, A. N-Nitrosuccinimide and N-nitrophthalimide. *J. Org. Chem.* **19**, 1662–1670 (1954).

(21) Calvert, J., Eberson, L., Hartshorn, M. P. & Svensson, J. O. Succinimidylation and nitration of aromatic compounds by photolysis with N-nitrosuccinimide. *J. Chem. Soc. Perkin Trans. 2* 645–652 (1995).

(22) Kozlova, I. K., Luk'yanov, O. A. & Tartakovskii, V. A. Chemistry of nitro imides. *Bull. Acad. Sci. USSR Div. Chem. Sci.* **30**, 1712–1714 (1981).

(23) Runge, J. & Treibs, W. Die Darstellung von N-Nitroacylalkylamiden und -diacylimiden mit Hilfe von Distickstoffpentoxyd. *J. für Prakt. Chemie* **15**, 223–227 (1962).

(24) Fulkrod, J. E. A laboratory study of strike and inductive effects. *J. Chem. Educ.* **51**, 115 (1974).

(25) Nieves-Quinones, Y. & Singleton, D. A. Dynamics and the regiochemistry of nitration of toluene. *J. Am. Chem. Soc.* **138**, 15167–15176 (2016).

(26) Hammond, G. S., Douglas, K. J., Hammond, G. S. & Douglas, K. J. Aromatic nitration. III. The ortho to para ratio in nitration of benzonitrile. *J. Am. Chem. Soc.* **81**, 1184–1187 (1959).

(27) Cummings, W. M., Cox, C. H. & Snyder, H. R. Arylboronic Acids. A Medium-Size Ring Containing Boronic Ester Groups. *J. Org. Chem.* **34,** 1669–1674 (1969).

(28) Ainley, A. D. & Challenger, F. CCLXXX.—Studies of the boron–carbon linkage. Part I. The oxidation and nitration of phenylboric acid. *J. Chem. Soc. Perkin Trans. 1* 2171–2180 (1930).

(29) Steinmetz, G. & Thiele, K. 2,6-Dichloro-3-nitro-pyridine. patent, US3809695 (1968).

(30) Zohuri, G. H., Seyedi, S. M., Sandaroos, R., Damavandi, S. & Mohammadi, A. Novel late transition metal catalysts based on iron: Synthesis, structures and ethylene polymerization. *Catal. Letters* **140**, 160–166 (2010).

(31) Ju, K.-S. & Parales, R. E. Nitroaromatic compounds, from synthesis to biodegradation. *Microbiol. Mol. Biol. Rev.* **74**, 250–72 (2010).

(32) Nepali, K., Lee, H.-Y. & Liou, J.-P. Nitro-group-containing drugs. *J. Med. Chem.* **62**, 2851–2893 (2019).

(33) Gillis, J. C. & Wiseman, L. R. Secnidazole. A review of its antimicrobial activity, pharmacokinetic properties and therapeutic use in the management of protozoal infections and bacterial vaginosis. *Drugs* **51**, 621–638 (1996).

(34) Hirai, K. in *Peroxidizing Herbicides* (Springer, Berlin, Germany, 1999), pp. 15–72.

(35) Galabov, B., Nalbantova, D., von Ragué Schleyer, P. & Schaefer, H. F. Electrophilic aromatic substitution: New insights into an old class of reactions. *Acc. Chem. Res.* **49**, 1191–1199 (2016).

(36) Koleva, G., Galabov, B., Hadjieva, B., Schaefer, H. F. & von Ragué Schleyer, P. An Experimentally established key intermediate in benzene nitration with mixed acid. *Angew. Chem. Int. Ed.* **127**, 14329–14333 (2015).

(37) Lu, L., Liu, H. & Hua, R. HNO_3/HFIP: A Nitrating system for arenes with direct observation of π-complex intermediates. *Org. Lett.* **20**, 3197–3201 (2018).

(38) Smith, M. & March, J. *March's advanced organic chemistry: reactions, mechanisms, and structure* (Wiley, New York, ed. 6th, 2006).

(39) Colomer, I., Chamberlain, A. E. R., Haughey, M. B. & Donohoe, T. J. Hexafluoroisopropanol as a highly versatile solvent. *Nat. Rev. Chem.* **1,** 88 (2017).

(40) D'Amato, E. M., Börgel, J. & Ritter, T. Aromatic C–H amination in hexafluoroisopropanol. *Chem. Sci.* **10,** 2424–2428 (2019).

(41) Börgel, J., Tanwar, L., Berger, F. & Ritter, T. Late-Stage Aromatic C–H Oxygenation. *J. Am. Chem. Soc.* **140,** 16026–16031 (2018).

(42) Galabov, B., Koleva, G., Simova, S., Hadjieva, B., Shaefer III, H. F. & von Ragué Schleyer, P. Arenium ions are not obligatory intermediates in electrophilic aromatic substitution. *Proc. Natl. Acad. Sci. U. S. A.* **111**, 10067–10072 (2014).

(43) Torra, N., Urpi, F. & Vilarrasa, J. N. N-Nitrosation and N-nitration of lactams. From macrolactams to macrolactones. *Tetrahedron* **45**, 863−868 (1989).

(44) Romea, P., Aragones, M., Garcia, J. & Vilarrasa, J. Improved methods for the N-nitration of amides. *J. Org. Chem*. **56**, 7038−7042 (1991).

(45) Adams, C. M., Sharts, C. M. & Shackelford, S. A. Electrophilic tetraalkylammonium nitrate nitration. I. Convenient new anhydrous nitronium triflate synthesis and in-situ heterocyclic N-nitration. *Tetrahedron Lett*. **34**, 6669−6672 (1993).

(46) Bartoli, G., Babiuch, K., Bosco, M., Carlone, A., Galzerano, P., Melchiorre, P. & Sambri, L. Magnesium perchlorate as efficient Lewis acid: A Simple and convenient route to 1,4-dihydropyridines. *SYNLETT* **18**, 2897−2901 (2007).

(47) El-Awad, A. M., Gabr, R. M. & Girgis, M. M. Kinetics and mechanism of thermal decomposition of magnesium perchlorate catalysed by metal metavanadate additives. *Therm. Acta* **184**, 205−212 (1991).

(48) Yau, H. M., Croft, A. K. & Harper, J. B. 'One-pot' Hammett plots: A general method for the rapid acquisition of relative rate data. *Chem. Commun*. **2012**, 8937–8939 (2012).

(49) Yau, H. M., Haines, R. S. & Harper, J. B. A robust, "one-pot" method for acquiring kinetic data for Hammett plots used to demonstrate transmission of substituent effects in reactions of aromatic ethyl esters. *J. Chem. Educ*. **92**, 538–542 (2015).

(50) Frisch, M. J., Trucks, G. W., Schlegel, H. B., Scuseria, G. E., Robb, M. A.. Cheeseman, J. R., Scalmani, G., Barone, V., Mennucci, B., Petersson, G. A., Nakatsuji, H., Caricato, M., Li, X., Hratchian, H. P., Izmaylov, A. F., Bloino, J., Zheng, G., Sonnenberg, J. L., Hada, M., Ehara, M., Toyota, K., Fukuda, R., Hasegawa, J., Ishida, M., Nakajima, T., Honda, Y., Kitao, O., Nakai, H., Vreven, T., Montgomery, Jr., J. A., Peralta, J. E., Ogliaro, F., Bearpark, M., Heyd, J. J., Brothers, E., Kudin, K. N., Staroverov, V. N., Kobayashi, R., Normand, J., Raghavachari, K., Rendell, A., Burant, J. C., Iyengar, S. S., Tomasi, J., Cossi, M., Rega, N., Millam, N. J., Klene, M., Knox, J. E., Cross, J. B., Bakken, V., Adamo, C., Jaramillo, J., Gomperts, R., Stratmann, R. E., Yazyev, O., Austin, A. J., Cammi, R., Pomelli, C., Ochterski, J. W., Martin, R. L., Morokuma, K., Zakrzewski, V. G., Voth, G. A., Salvador, P., Dannenberg, J. J., Dapprich, S., Daniels, A. D., Farkas, Ö., Foresman, J. B., Ortiz, J. V., Cioslowski, J. & Fox, D. J. Gaussian 09, Revision D.01, Gaussian, Inc., Wallingford CT., **2009**.

(51) Zhao, Y. & Truhlar, D. G. The M06 suite of density functionals for main group thermochemistry, thermochemical kinetics, noncovalent interactions, excited states, and transition elements: two new functionals and systematic testing of four M06-class functionals and 12 other functionals. *Theor. Chem. Acc.* **120**, 215–241 (2008).

(52) Krishnan, R., Binkley, J. S., Seeger, R. & Pople, J. A. Self-consistent molecular orbital methods. XX. A basis set for correlated wave functions. *J. Chem. Phys.* **72**, 650−654 (1980).

(53) McLean, A. D. & Chandler, G. S. Contracted Gaussian basis sets for molecular calculations. I. Second row atoms, *Z*=11–18. *J. Chem. Phys.* **72**, 5639–5648 (1980).

(54) Tomasi, J., Mennucci, B. & Cammi, R. Quantum Mechanical Continuum Solvation Models. *Chem. Rev.* **105**, 2999–3094 (2005).

(55) Cossi, M., Scalmani, G., Rega, N. & Barone, V. New developments in the polarizable continuum model for quantum mechanical and classical calculations on molecules in solution. *J. Chem. Phys.* **117**, 43–54 (2002).

(56) Lide, D. R. *CRC Handbook of Chemistry and Physics*, 90th Edition, CRC Press, **2009**.

(57) Li, G.-X., Morales-Rivera, C. A., Gao, F., Wang, Y., He, G., Liu, P. & Chen, G. A unified photoredox-catalysis strategy for C(sp3)−H hydroxylation and amidation using hypervalent uodine. *Chem. Sci.* **8**, 7180–7185 (2017).

(58) Qi, C., Hasenmaile, F., Gandon, V. & Leboeuf, D. Calcium(II)-catalyzed intra- and intermolecular hydroamidation of unactivated alkenes in hexafluoroisopropanol. *ACS Catal.* **8**, 1734−1739 (2018).

(59) Koleva, G., Galabov, B., Kong, J., Schaefer III, H. F. & von Ragué Schleyer, P. Addition-elimination versus direct substitution mechanisms for arene chlorination. *Eur. J. Org. Chem.* **31**, 6918−6024 (2014).

(60) Kong, J., Galabov, B., Koleva, G., Zou, J. J., Schaefer III, H. F. & von Ragué Schleyer, P. The inherent competition between addition and substitution reactions of Br_2 with benzene and arenes. *Angew. Chem. Int. Ed.* **50**, 6809−6813 (2011).
