# Supplementary material for: Facile access to nitroarenes and nitroheteroarenes using N-nitrosaccharin
Source: Nat Commun. 2019 Jul 30;10:3410. doi: 10.1038/s41467-019-11419-y (PMC6667458; doi:10.1038/s41467-019-11419-y)
Supplement: Supplementary file 3 — Description of Additional Supplementary Data Files [file 41467_2019_11419_MOESM3_ESM.pdf]

Description of Additional Supplementary Files
File Name: Supplementary Data 1
Description: Crystallographic information file for compound **4a**.

File Name: Supplementary Data 2
Description: Crystallographic information file for compound **4b**.

File Name: Supplementary Data 3
Description: Crystallographic information file for compound **54**.

File Name: Supplementary Data 4
Description: Crystallographic information file for compound **61**.
