# Supplementary material for: Facile access to nitroarenes and nitroheteroarenes using N-nitrosaccharin
Source: Nat Commun. 2019 Jul 30;10:3410. doi: 10.1038/s41467-019-11419-y (PMC6667458; doi:10.1038/s41467-019-11419-y)
Supplement: Supplementary file 2 — Peer Review File [file 41467_2019_11419_MOESM2_ESM.pdf]

Editorial Note: This manuscript has been previously reviewed at another journal that is not operating a transparent peer review scheme. This document only contains reviewer comments and rebuttal letters for versions considered at *Nature Communications*.

REVIEWERS' COMMENTS:

Reviewer #1 (Remarks to the Author):

The revised manuscript has addressed the questions/comments in the previous reviewer's report. In brief, the new reagent reported in this manuscript offers a mild and practical method for the nitration of a diverse range of arenes and heteroarenes, with excellent functional group tolerance. The protocol is also effective for late stage nitration of complex aromatics bearing sensitive functional groups, demonstrating its great potentials for the preparation of functionally important materials. The manuscript is suitable for publication in Nature Communications.

Reviewer #2 (Remarks to the Author):

The quality of this revised manuscript has been improved by addressing the questions from the reviewers. I recommend it publication as it is.
